# Disease-related miRNA mutations are associated with mature miRNA secondary structure changes

**DOI:** 10.1016/j.bpj.2025.09.049

**Published:** 2025-10-03

**Authors:** Javor K. Novev, Sebastian E. Ahnert

**Affiliations:** 1 Department of Chemical Engineering and Biotechnology, University of Cambridge, Cambridge, United Kingdom; 2 Institute of Genetics and Cancer, The University of Edinburgh, Western General Hospital, Edinburgh, United Kingdom; 3 The Alan Turing Institute, London, United Kingdom

## Abstract

MicroRNAs (miRNAs) are ubiquitous short RNAs regulating gene expression in many organisms, including humans. How the secondary structure (SS) of a mature miRNA affects its regulatory function remains an open question. Here, we investigate this question through computational SS predictions of miRNA point mutants. We explore the mutational neighborhoods of miRNAs with association to human diseases, including cancer. We focus on possible SS changes independent of target-site complementarity by leaving the seed region unchanged. We formulate metrics of the SS differences between such mutants and their wild types (WTs) and test whether disease-associated mutations tend to differ from others in terms of these metrics by comparing our results with the miRNASNP-v3 database. We find that disease-related mutants tend to have a higher probability of being fully unfolded than their WT; this and other SS-related measures are statistically significant at the database level. This is confirmed when we restrict the analysis to the better-validated miRNAs encoded by genes that appear in the manually curated MiRGeneDB database. With the same approach, we identify a subset of individual miRNAs for which SS changes are most likely to be related to disease. These are hsa-miR-1269b, hsa-miR-4537, hsa-miR-4477b, hsa-miR-4641, and hsa-miR-6821-3p; when focusing on the higher-confidence MiRGeneDB miRNAs, we find that hsa-miR-485-5p and hsa-miR-1908-3p are the ones for which SS changes are most likely to be linked to disease. In addition, we show that there are pairs of known miRNA WTs differing only by disease-related point mutations outside the seed region that exhibit very different SS. These pairs include hsa-miR-1269a—hsa-miR-1269b and hsa-miR-3689a-3p—hsa-miR-3689b-3p.

## Significance

microRNAs regulate the expression of large numbers of genes, including many disease-associated ones, through binding to mRNAs as part of the RNA-induced silencing complex (RISC). Their mature form, which is found in RISC, is canonically thought not to have secondary structure, and the folding of mature microRNAs has received little attention in the literature. We use in silico tools to predict the folding of microRNA point mutants and then check whether mutations reported to be disease-related are associated with changes in secondary structure. We find that several independent measures of the secondary structure changes introduced by such mutations discriminate between disease-related and other mutations. Our work addresses a fundamental question in RNA biology that is relevant to many diseases.

## Introduction

MicroRNAs (miRNAs) are short (typically 19–27 nt (1)) regulatory molecules that are highly conserved across many species (2). They regulate gene expression by entering an RNA-induced silencing complex (RISC) (3), which typically then binds to the 3′ untranslated region (UTR) of mRNAs and inhibits their translation (4,5,6), although recent work has also uncovered miRNA-induced translational activation via 5′ UTR binding (7). In humans and other animals, this binding canonically only occurs in the short “seed” region of the miRNA (nt 2–7) (8). This canonical picture is highly simplified; it is known that partial complementary binding to nucleotides outside this region is also common (9,10), and modes of binding that do not involve the seed have also been recorded (11). All modes of miRNA binding, however, rely on binding to a short target sequence that can be found in a large set of mRNAs. A single miRNA may therefore regulate a broad range of genes (2,3). Abnormal miRNA expression is observed in many diseases (4), including Parkinson's (12) and cancer (5,8,12,13,14), with evidence indicating that miRNA expression is globally suppressed in tumor cells (4,5). Individual miRNAs typically have either oncogenic or tumor-suppressive effects (3,5), but several miRNAs (15) and miRNA families (16) can reportedly act as both oncogenes and tumor suppressors depending on context. Such a combination of tumor suppressive and carcinogenic effects is to be expected given the broad range of targets a single miRNA can regulate.

RNA molecules can fold due to pairing between some of their nucleobases, which can be described in terms of RNA secondary and tertiary structures. The role of miRNA secondary structure in the context of miRNA function has received some attention in the past, much of which has focused on miRNA precursors, also known as primary miRNAs (pri-miRNAs) or pre-miRNAs depending on their processing stage (2,17,18). In particular, Diederichs and Haber, who searched for tumor-associated mutations in cancer-derived cell lines, found no mutations within the mature miRNA sequences, and even though some of the pri-miRNA mutations dramatically altered secondary structure, they did not have an effect on in vivo processing and maturation (17). Sun et al. (19) investigated how single-nucleotide polymorphisms (SNPs) in mature miRNAs affect their function, noting that the variant miR-502-C/G produces a bulge that changes the structure of the pre-miRNA’s stem and likely affects the latter’s processing; a similar effect has also been observed in miR-125a (20). Confoundingly, there are some examples of SNPs that are associated with increased risk of some diseases but decreased risks for others; the polymorphism rs2910164 in miR-146a-3p is known to predispose carriers to breast cancer, glioma, and gastric cancer but at the same time is protective against prostate and gastric cancer (21).

It is not immediately clear to what extent the secondary structure of the mature miRNA affects its activity, since the single-stranded mature miRNA is typically bound to RISC, which may affect its folding. Before RISC binding, a pri-miRNA is converted into a pre-miRNA by the ribonuclease III enzyme Drosha and converted into a double-stranded RNA of length 22 nt, which binds to Argonaute (Ago) proteins to form RISC (22,23). However, known crystal structures of RISC proteins lack information on the position of some miRNA nucleotides, which suggests that miRNAs have some flexibility within the complex and may be able to form base pairs (24). An additional argument supporting the study of mature miRNA secondary structure is that other cases of base-pairing within ribonucleoproteins are known, with base-pairing within the ribosome (25) being a paradigmatic example. The filaments containing the genetic material of the influenza virus are another example. Although it was previously thought that RNA secondary structure is completely melted within these complexes, i.e., that there is no base-pairing, base-pair formation within them is now known to be possible (26) and has been experimentally observed; see Ref. (27) and the references therein, as well as the review of the topic in Ref. (28).

Belter et al. (29) showed that some mature miRNAs form stable secondary structures and suggested that these play a functional role. In a different study (30), the same group identified common secondary structure motifs in mature miRNAs and proposed that 1) base-pairing may confer resistance to nucleases, and that 2) target recognition may involve not only miRNA sequence but also secondary and tertiary structure. A striking example of mature miRNA folding playing a role in disease is the single-stranded mature miR-1-3p, which assumes aptamer-like secondary structure, modifying the function of ion channels in cardiomyocytes. Genetic variants affecting this miRNA, for example rs1399433486, have been reported in patients with cardiovascular diseases such as juvenile onset atrial fibrillation (31). This and a host of other biological functions of mature miRNAs beyond RISC have been reviewed by Santovito and Weber (31) and Makarova et al. (32). Moreover, evidence from multiple studies (33,34) indicates that for many miRNAs a large fraction of the molecules in vivo are not bound to RISC, but it is not currently understood where these miRNAs are located (33).

The generally low frequency of mutations in mature miRNAs has been commented on by Bracken et al. (6), who presume this is due to their small size. SNPs, particularly ones in the seed region, are associated with many diseases (22). In reviewing the literature on genetic variations in miRNAs, Borel and Anonarakis remark that data indicate a low density of polymorphisms in the seed region, suggesting a selective constraint (35).

**Figure 1:**
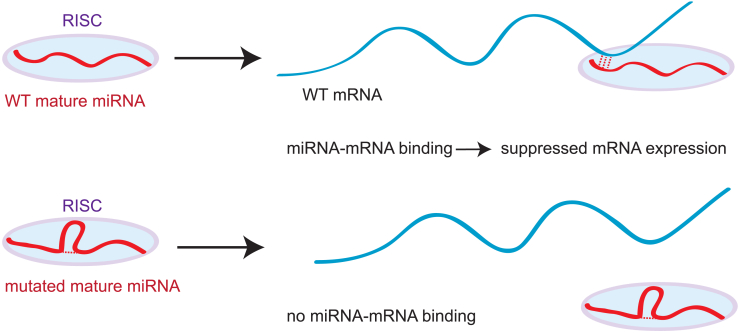
We hypothesize that miRNA mutations outside the seed region that change miRNA secondary structure can affect miRNA-mRNA binding and thus gene expression. Illustration of the proposed mechanism by which mature miRNA SS may influence miRNA activity. Folding of the mutant miRNA (*bottom*) reduces its affinity to target mRNAs in comparison with the WT (*top*). Our methods cannot provide mechanistic insights into miRNA activity, but it is possible that both miRNA activity within RISC and non-canonical pathways such as those detailed in Refs. (31,32) are affected by changes in mature miRNA secondary structure.

To our knowledge, however, no study has systematically explored mutation-induced changes in mature miRNA secondary structure or attempted to analyze the association between such changes and disease; it is this knowledge gap that we address in the present work. We hypothesize that miRNA activity could be influenced by secondary structure, because the folding of a mature miRNA may reduce its binding affinity to target mRNAs and might influence its activity within RISC, as illustrated schematically in Fig. 1, or affect non-canonical miRNA function, for example by protecting free miRNAs from degradation, as hypothesized in Ref. (32). Other researchers have designed the miRVaS tool in an attempt to predict the impact of genetic variants on miRNAs (36) based on how they affect secondary structure of miRNA precursors. Our aim, however, is not to predict the effect of mutations but to ascertain whether the secondary structure of mature miRNAs is relevant to their activity based on mutations previously characterized as associated with disease.

We study the effect of secondary structure in mature miRNAs through predicting the secondary structure changes introduced by point substitution mutations that preserve the seed region. In genotype space, such a set forms a “partial” point mutational neighborhood of the WT miRNA under consideration, as opposed to the “complete” point mutational neighborhood, which contains all mutants accessible from the WT via a point substitution mutation. We then formulate multiple quantitative criteria for measuring how the secondary structure of each mutant in the partial neighborhood differs from the wild type (WT). Thereafter, we test how well these criteria can predict which mutations are associated with disease. We do so by ranking the mutated sequences by a given criterion of secondary structure change and assess whether disease-related mutants are ranked higher than expected by chance.

When studying how mutations affect secondary structure, we place a particular emphasis on calculating how likely the fully unfolded state, i.e., the one with trivial secondary structure, is. Since miRNAs regulate gene expression by binding to mRNAs, one may expect that mutations that alter the probability that a given miRNA is fully unfolded may also affect its propensity to bind, as an unfolded molecule may interact more readily with its target than a stably folded one. Mutations that lower the likelihood that a miRNA is unfolded may affect miRNA function in this way and thus be associated with disease. This is one of the key hypotheses that we test in this study.

## Methods

### Theoretical basis

#### Secondary structure prediction

We use version 2.5.0 of the ViennaRNA package (37) to predict and analyze the secondary structure of mutant and WT miRNAs.

#### Statistical significance of ROC results

We use the area under the receiver operating characteristic (ROC) curves, *A*_ROC_, as a measure of the power of Δ*p*_unfolded_ and other SS metrics as predictors of the association of a given mutation with disease. We then assess the statistical significance of the results by calculating the two-sided Mann-Whitney *p*-values for the null hypothesis that the Δ*p*_unfolded_ distributions for the disease-related and other mutants have the same median; we use the built-in MATLAB R2022b function for this purpose (38). The error bars in ROC plots indicate pointwise 95% confidence bounds for the true positive rates and are calculated via bootstrapping with 10^4^ samples and vertical averaging for 21 equally spaced values of the false positive rate from 0 to 1; for more details, see (39) and the references cited therein.

#### Boltzmann frequency of structures

The frequency of a particular secondary structure *s* with energy *E*(*s*) in the thermodynamic equilibrium ensemble is (40)

(1) $$p_{s}=\frac{1}{Z}\;\exp\;\left(-\frac{E\left(s\right)}{RT}\right)\text{,}$$

with *R* denoting the gas constant, and *T* is the temperature, for which we use the biologically relevant value 310.15 K, and $Z=\sum_{\forall s}\;\exp\;\left(-\frac{E\left(s\right)}{RT}\right)$, the partition function; for the fully unfolded state, there is no base-pairing, *E*(*s*) = 0, and *p*_unfolded_ = *Z*^−1^.

#### RNAsubopt

We use RNAsubopt to generate a sample of structures drawn randomly from the Boltzmann ensemble according to their probability, starting with a sample size of 10. In case the fully unfolded structure is not present in the initial sample, we increase the sample size by a factor of 10, and we repeat the process until we either encounter the trivial secondary structure or reach 10^10^ samples. In the latter case, the Boltzmann weight for this structure is negligible, and we use the approximation *p*_unfolded_ ≈ 0. A sample size as small as 10 can be sufficient because RNAsubopt calculates the partition function *Z* of the ensemble and uses stochastic backtracing to generate suboptimal structures (41). The Boltzmann weights from RNAsubopt simply reflect Eq. (1), making them independent of the sample size. In contrast, when using samples generated with RNAsubopt to approximate thermodynamic ensembles and calculate distances between them, the sample size needs to be large enough to find all folds with appreciable probability. For this reason, when calculating average Hamming distances between ensembles, we use 10^5^ samples for mature miRNAs and 10^3^ samples for pre-miRNAs, with the reason for using fewer samples in the latter case being the computational cost of sampling for the much longer precursors.

#### Hamming distance metric

We measure the differences between two secondary structures *s*_1_ and *s*_2_ in the dot-bracket representation, where dots represent unpaired sites and brackets represent base pairs (see for example (42)), via the Hamming distance between the two, i.e., the number of sites for which there are discrepancies (*d*_Hamming_(*s*_1_,*s*_2_)). For each mutant in the partial point mutational neighborhoods of the miRNAs in miRNASNP-v3 (Database: miRNASNP-v3, https://guolab.wchscu.cn/miRNASNP/), we average the pairwise Hamming distance between the possible secondary structures in its equilibrium ensemble and those for the equilibrium ensemble of the WT, weighted by their joint Boltzmann probability,

(2) $$\left\langle d_{\text{Hamming}\;\text{mutant}}\right\rangle =\sum_{\forall s_{\text{mutant}}}\sum_{\forall s_{\text{WT}}}p_{s_{\text{mutant}}}p_{s_{\text{WT}}}d_{\text{Hamming}}\left(s_{\text{mutant}},s_{\text{WT}}\right)\text{.}$$

The sums in Eq. (2) contain the secondary structures that RNAsubopt has drawn from the respective equilibrium ensembles on the basis of a sample with 10^5^ entries for mature miRNAs and 10^3^ samples for pre-miRNAs. Structures that have not been encountered in drawing this sample have only a negligible contribution to ⟨*d*_Hamming_⟩. For the criteria based on ⟨*d*_Hamming_⟩*L*^−1^, unlike most other quantities we study, we rank mutants in “ascending order,” which means that top-ranked entries are closest to the WT. We do this to ease the readability of the results because for large data sets, mutants associated with disease tend to be closer in secondary structure to the WT than other mutants; however, this trend is reversed for some individual point mutational neighborhoods such as that of the microRNA hsa-miR-4537, which is illustrated in Fig. 3.

#### Additional metrics of secondary structure distance between ensembles

We use two other metrics that characterize how far the Bolztmann ensemble of a miRNA mutant is from its WT. The first one is based on the distance calculated by the ViennaRNA program RNApdist (37). RNApdist calculates a vector with the probabilities that each base is unpaired, paired upstream, or paired downstream; these vectors are then compared via an alignment algorithm. In addition to RNApdist, we use a simpler metric: for each base, we calculate the probability that it is paired to any other base $p_{\text{bp}}^{\left(i\right)}$ from the output of RNAfold (37), average this quantity over the entire molecule, and then calculate the difference between these averages for the mutant and the WT as $\left\langle \Delta p_{\text{bp}}\right\rangle =\left\langle p_{\text{bp},\text{mutant}}^{\left(i\right)}-p_{\text{bp},\text{WT}}^{\left(i\right)}\right\rangle$.

#### Positional entropy

The positional entropy of site “i” within an RNA molecule is defined as

(3) $$S^{\left(i\right)}=-\sum_{j\neq i}p_{i,j}\;\ln\;p_{i,j}-p_{i}^{\text{unpaired}}\;\ln\;p_{i}^{\text{unpaired}}\text{,}$$

where *p*_i,j_ is the probability that site “i” is paired with site “j,” and $p_{i}^{\text{unpaired}}=1-\sum_{j\neq i}p_{i,j}$ is the probability that site “i” is unpaired (43). When building ROC curves for *S*^(i)^, we sort mutants in “ascending order,” as we do for the criteria based on the mutant-WT Hamming distance. This means that the top-ranked entries are those for which the difference in positional entropy at the mutated site is lowest.

### Measures of secondary structure change in mutants

#### Change in the probability of the fully unfolded state

**Figure 2:**
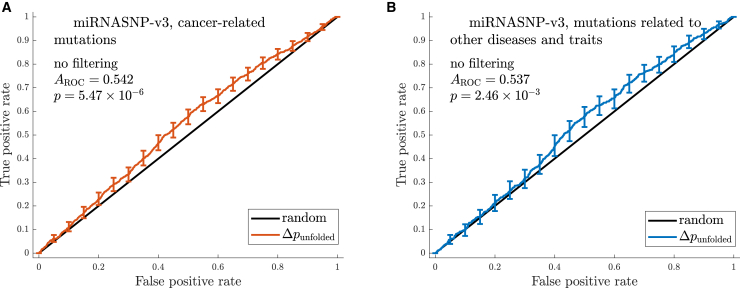
The change in probability that the miRNA is fully unfolded associated with a mutation is a predictor of the mutation’s relationship with disease. ROC curves were built using Δ*p*_unfolded_ = *p*_unfolded mutant_ − *p*_unfolded WT_ as the criterion for predicting disease-related mutations. Curves are based on all data on cancer-related mutations (*A*) and mutations related to other traits and diseases (*B*) from miRNASNP-v3, with no filtering based on *N*_mut seed_ and *N*_mut non__-__seed_. Error bars indicate pointwise 95% confidence bounds for the true positive rates in the ROC plots calculated via bootstrapping and vertical averaging for 21 equally spaced values of the false positive rate from 0 to 1; see (39) and the references cited therein. The area under the ROC curve (*A*_ROC_) and the Mann-Whitney *p*-value (38) for the curves are also indicated. The Δ*p*_unfolded_ criterion performs significantly better than the random one for both data sets (*p* < 0.05), indicating that the probability that disease-related mutants are fully unfolded tends to be higher than that for other mutants. This could be because mutants with a higher *p*_unfolded mutant_ have a higher activity than their respective WTs, and in the case of cancer-associated mutations, they may be more effective at downregulating tumor suppressor genes.

We calculate the Boltzmann probabilities *p*_unfolded,WT_ and *p*_unfolded, mutant_ that WT and mutant miRNAs are fully unfolded, using the ViennaRNA program RNAsubopt (see methods). We then consider the difference between these two quantities,

$$\Delta p_{\text{unfolded}}=p_{\text{unfolded},\text{mutant}}-p_{\text{unfolded},\text{WT},}$$

for all mutants in the point mutational neighborhood of the WT except those that have an altered seed region and rank them by Δ*p*_unfolded_. We establish whether Δ*p*_unfolded_ is a useful predictor of the association between mutations and disease by ranking all mutants in “descending order” of their values of Δ*p*_unfolded_ and building an ROC curve (44), which indicates whether disease-related mutants tend to rank higher than the rest (Fig. 2).

#### Change in the secondary structure Boltzmann ensemble

If the folding of a miRNA plays a role in its function, then disease-related mutations can be expected to be the ones that cause the greatest change in miRNA secondary structure. We formulate quantitative measures of the phenotypic distance of mutants from their WTs and rank the mutants in “descending order” according to these measures, i.e., compile a ranking with mutants that are furthest away from the WT at its top. Since our hypothesis is that miRNA secondary structure impacts function, we expect mutants that rank high according to these criteria to have a stronger association with disease than other mutants. We quantify the difference between two secondary structure Boltzmann ensembles by calculating the average Hamming distance across all pairwise comparisons between structures in the two different ensembles (see theoretical basis). We then use the average mutant-WT Hamming distance ⟨*d*_Hamming_⟩ to formulate a coarse-grained criterion, namely the percentile ranking of each individual mutant within its point mutational neighborhood. We build ROC curves for this criterion based on data from miRNASNP-v3, and check whether the corresponding Mann-Whitney *p*-values fall below our significance threshold of 0.05.

#### Change of the positional entropy of the mutated site

For any given site in an RNA molecule, we can calculate the positional entropy *S*^(i)^, which measures how variable the pairing of this site is within the Boltzmann ensemble. A site with low *S*^(i)^ is thus one that is consistently paired or unpaired across the ensemble, whereas a high *S*^(i)^ indicates that the site participates in pairings in many structures but is unpaired in many others. We quantify this effect for all mutants in the partial point mutational neighborhoods of the miRNAs represented in miRNASNP-v3 by calculating the difference between the positional entropy of the mutated site for mutant and the WT,

$$\Delta S^{\left(i\right)}=S_{\text{mut}}^{\left(i\right)}-S_{\text{WT}}^{\left(i\right)}\text{,}$$

as a measure of this change in stability (see theoretical basis).

#### Distance between Boltzmann ensembles

We calculate this with the ViennaRNA program RNApdist (37) and normalized by its maximum possible value *d*_Boltzmann_ = *d*_RNApdist_/(2*L*).

#### Difference between the average base-pairing probabilities in the mutant and the WT

This quantifies the overall change in secondary structure caused by point substitution mutations. As explained in the theoretical basis section, we calculate it as $\left\langle \Delta p_{\text{bp}}\right\rangle =\left\langle p_{\text{bp},\text{mutant}}^{\left(i\right)}-p_{\text{bp},\text{WT}}^{\left(i\right)}\right\rangle$, where $p_{\text{bp}}^{\left(i\right)}$ is the probability that base “i” is paired to any other base, calculated from the output of RNAfold (37).

The secondary structure of an miRNA may have an effect on its function in two ways; it could either have its own functional purpose, or it could affect the interaction with the target site as it would make the binding sites within the miRNA less accessible. In the first case, a mutant may disrupt function by causing a change to the minimum free energy (MFE) secondary structure or making it less stable; in the second one, a mutation may interfere with miRNA function by causing sites essential to target-binding to enter base pairs. The five metrics outlined above aim to quantify different types of secondary structure changes in order to detect any kind of association between modified miRNA secondary structure and disease.

### Sources of data on disease-related mutations

Although disease-associated miRNAs exhibit fewer SNPs than non-disease-related miRNAs (45), there is a substantial amount of data on the association of specific miRNA point mutations with disease. We used a database of this kind to examine the changes in secondary structure as a result of disease-related mutations. This database is miRNASNP-v3 (46), which contains data on miRNA mutations related to various diseases. We also analyzed the somatic mutations in cancer collected in the SomamiR 2.0 database (47) (Database: SomamiR 2.0, https://compbio.uthsc.edu/SomamiR/home.php), but as almost all of them are covered in miRNASNP-v3, we only discuss these results in the supporting material. Note that other online databases with mutations in miRNAs exist. For an overview, see Fehlmann et al. (48). The miRNASNP-v3 database, which we use in its version from May 14th, 2024, contains 2613 distinct entries recorded for the mature region. We filtered the mutations in the miRNASNP-v3 database by several criteria. Firstly, we filter out the 17 entries in miRNASNP-v3 for which the related disease was unspecified and a further 34 mutations for which the WT miRNA sequence in the miRNASNP-v3 entry is not an exact match for the one given in miRBase (49), or for which the mutated miRNA sequence specified in miRNASNP-v3 does not match the one derived from mutating the corresponding DNA sequence in the Genome Reference Consortium Human Build 38 (50).

The stringency of criteria for miRNA discovery varies by study (49), and it is difficult to distinguish bona fide miRNAs from fragments of other RNAs (49). Thus, many reported miRNAs are false positives, and previous studies have reported a false positive rate of up to two-thirds (51). As miRBase only provides minimal gate-keeping at the point of submitting a new miRNA entry (49), merely cross-checking that a miRNA is found in miRBase as we do above allows for low-confidence miRNAs in our data sets. It is not our aim here to curate or assess the quality of miRNA data, so for a more stringent filtering miRNA mutations, we make use of the manually curated database MiRGeneDB 2.1 (51) (Database: MiRGeneDB, https://web.archive.org/web/20230402091252/https://mirgenedb.org/download). We report the results of restricting our analysis only to the set of miRNAs encoded by genes that appear in the version of MiRGeneDB 2.1 (51) downloaded on October 12th, 2024. We provide information on the number of mutants in the different datasets in Table 1.

**Table 1:** Number of mutations in the datasets we analyzed split by the type of disease they are associated with (cancer vs. others), their location (seed vs. non-seed), and whether the genes encoding for them appear in MiRGeneDB

Data set	Total	Seed	*N*_mature miRNAs affected_
All valid miRNASNP-v3 entries	2562	783	1209
	Number of mutations		
Point substitutions outside the seed region		1693	
Cancer-associated point substitutions outside the seed region		1105	
Point substitutions outside the seed region associated with other diseases		588	
	Total	Cancer	Other diseases
Number of unique sequences	1526	1013	564
Total number of mutants studied		32,040	18,876
*N*_mature miRNAs affected_	938	675	400
Number of unique sequences of mutants in miRNAs represented in MiRGeneDB 2.1	553	441	124
Total number of mutants in miRNAs represented in MiRGeneDB 2.1		14,211	4566
*N*_mature miRNAs affected_ represented in MiRGeneDB 2.1	336	295	95

In some of the analysis that follows, we consider filtered subsets of the data with different minimum numbers of mutations in the seed region or the non-seed region, as the presence of several disease-associated mutations in a given miRNA suggests a higher likelihood that the miRNA is directly involved in the disease mechanism.

## Results

### A significant proportion of disease-related miRNA mutations are associated with secondary structure changes

In Table 2, we compare the three best-performing of the quantitative measures outlined above in terms of their power to predict disease-related mutations. To illustrate the performance of the criteria, we present the underlying ROC curves for Δ*p*_unfolded_ in Fig. 2. We apply the three criteria to data from miRNASNP-v3, split between mutations associated with cancer and other traits and diseases. We provide additional ROC curves in the supporting material, where we give details of other criteria we considered.

**Table 2:** The three main criteria for measuring changes in miRNA secondary structure as a result of mutations are Δ*p*_unfolded_, the ⟨*d*_Hamming_⟩ percentile, and Δ*S*^(i)^

Criterion	Cancer		Other diseases	
	*A*_ROC_	*p*-value	*A*_ROC_	*p*-value
Δ*p*_unfolded_	0.542	5.47 × 10^−6^	0.537	2.46 × 10^−3^
⟨*d*_Hamming_⟩%ile	0.548	1.61 × 10^−7^	0.532	1.04 × 10^−2^
Δ*S*^(i)^	0.528	2.62 × 10^−3^	0.534	5.41 × 10^−3^

Mutations are ranked by these criteria, and the receiver operator characteristic *A*_ROC_ is used to establish whether they predict disease-associated mutations in the miRNASNP-v3 data set, split into mutations associated with cancer and those with other diseases. The *A*_ROC_ values exceed 0.5 significantly for all three metrics in both subsets of the data, suggesting that at least for some miRNAs, secondary structure changes can be indicative of disease association. When checking for significance, we calculate the Mann-Whitney *p*-value and set the threshold for significance to 0.05.

Δ*p*_unfolded_ is significantly better at predicting disease-associated mutations than the random criterion for data from miRNASNP-v3, suggesting that miRNAs with a fully unfolded mature form differ in activity from folded ones. In particular, disease-related mutations tend to increase the likelihood that a miRNA is unfolded more than other mutations. We show the ROC curves for this criterion for the two subsets of mutations from miRNASNP-v3 in Fig. 2. We verify that mutants with large Δ*p*_unfolded_ tend to cause a change to a less folded structure by calculating the Spearman rank correlation coefficient of Δ*p*_unfolded_ with the average change in base-pairing probability, ⟨Δ*p*_bp_⟩. The resulting *R*_Spearman_ ∼ −0.30 with *p*-value < 10^−100^ shows comparatively weak but very significant negative correlation; i.e., as one would expect, the fully unfolded state tends to be more common in the mutated ensemble when the mutation causes an overall reduction in base-pairing.

Using the percentile ranking of ⟨*d*_Hamming_⟩ of mutants within their neighborhoods also leads to *A*_ROC_ significantly greater than 0.5 for data from miRNASNP-v3. This is an independent indication that miRNA secondary structure plays a role in miRNA association with disease. Since we sort mutants in “ascending order” of this criterion, the analysis indicates that disease-related mutations tend to change secondary structure less than other mutations. The criterion based on Δ*S*^(i)^, the difference in the positional entropy of the mutated site, performs better than random for the data in miRNASNP-v3. As we rank mutants in “ascending order,” this means that disease-associated mutants tend to be those for which the difference $\Delta S^{\left(i\right)}=S_{\text{mut}}^{\left(i\right)}-S_{\text{WT}}^{\left(i\right)}$ is lower.

We also calculated correlation coefficients between other pairs of secondary structure change quantifiers. *R*_Spearman_ ∼ −0.1 between Δ*p*_unfolded_ and the percentile of ⟨*d*_Hamming_⟩, indicating that these can be treated as orthogonal criteria. *R*_Spearman_ ∼ −0.4 between Δ*p*_unfolded_ and Δ*S*^(i)^, and ∼0.3 between Δ*S*^(i)^ and ⟨*d*_Hamming_⟩, which indicates that the rankings according to these criteria are more strongly correlated. We observe the same general trends when measuring correlations between the same criteria for a set of miRNAs highlighted by multiple hypothesis testing as described in the section entitled disease-related mutations change the secondary structures of a specific set of individual miRNAs below. We provide details on *R*_Spearman_ and the associated *p*-values for several pairs of criteria in Tables S11–S13 in the supporting material.

We checked whether the three main criteria, Δ*p*_unfolded_, the ⟨*d*_Hamming_⟩ percentile, and Δ*S*^(i)^, highlighted similar sets of mutations. In accordance with the order of ranking mutants we use for the respective criteria, we took the 1% of mutants with the highest Δ*p*_unfolded_ and the 1% of mutants with the lowest ⟨*d*_Hamming_⟩ percentile and Δ*S*^(i)^—this defines the sets of top 1% of mutants according to each of these criteria. We then tested whether individual mutants were among the 1% selected according to more than one criterion and found very few such mutations. Within the set of mutations related to cancer, the top 1% sets of mutants according to Δ*S*^(i)^ and Δ*p*_unfolded_ have one mutant in common, and the same is true of the top 1% sets defined according to ⟨*d*_Hamming_⟩% ile and Δ*S*^(i)^. In the set of mutations related to other diseases, one is common between the top 1% defined according to ⟨*d*_Hamming_⟩% ile and Δ*S*^(i)^. This shows that although the three rankings are significantly correlated with each other, they point to different sets of mutations as most likely to be associated with disease. These differences introduce uncertainty in our conclusions that may be due to different mechanisms by which miRNA secondary structure influences miRNA function, but also due to the indirect relationship between miRNA mutations and disease. Ideally, future experimental study of the mutational neighborhoods of selected miRNAs will help resolve these issues.

The criterion measuring the distance between the mutated and WT Boltzmann ensembles, *d*_Boltzmann_, has an *A*_ROC_ >0.5 and significantly better than random performance when applied to mutants from the miRNASNP-v3 cancer data set that are put in “ascending order.” Equivalently, in this set the mutations that cause a smaller change to the Boltzmann ensemble tend to be associated with disease. The difference between the average base-pairing probabilities in the mutant and the WT (⟨Δ*p*_bp_⟩) does not perform better than the random criterion for the miRNASNP-v3 data sets with no filtering by the number of mutations. For brevity, we only provide *A*_ROC_ and *p*-values for *d*_Boltzmann_ and ⟨Δ*p*_bp_⟩ in Tables S3–S10 in the supporting material.

As Table 3 indicates, restricting the analysis only to the high-confidence miRNAs encoded by genes from MiRGeneDB reduces the statistical power of our methods. Despite that, the Δ*p*_unfolded_ criterion consistently meets our significance threshold when applied to the data for the subset of miRNAs from miRNASNP that are represented in MiRGeneDB 2.1. The other two main criteria, ⟨*d*_Hamming_⟩ percentile and Δ*S*^(i)^, meet our threshold for significance only when applied to the cancer-related mutations in miRNASNP-v3 that also appear in MiRGeneDB 2.1. Note, however, that the *p*-values for all three main criteria (Δ*p*_unfolded_, ⟨*d*_Hamming_⟩ percentile, and Δ*S*^(i)^) and *d*_Boltzmann_ are below our threshold of 0.05 when applied to the miRNASNP data set without splitting according to disease, regardless of whether cross-validation with MiRGeneDB is performed; see Tables S1 and S2 in the supporting material for details. We considered alternative approaches to quantifying secondary structure changes as well as ways to filter the data according to the number of mutations; the interested reader may find them in the supporting material.

**Table 3:** Δ*p*_unfolded_ consistently meets our significance threshold when applied to the data for the subset of high-confidence miRNAs from miRNASNP that are represented in MiRGeneDB 2.1

Criterion	Cancer		Other diseases	
	*A*_ROC_	*p*-value	*A*_ROC_	*p*-value
Δ*p*_unfolded_	0.559	2.25 × 10^−5^	0.562	1.76 × 10^−2^
⟨*d*_Hamming_⟩%ile	0.550	3.05 × 10^−4^	*0.535*	*0.69*
Δ*S*^(i)^	0.543	2.23 × 10^−3^	*0.490*	*0.18*

The other two main criteria, ⟨*d*_Hamming_⟩ percentile and Δ*S*^(i)^, only meet the significance threshold when applied to the cancer-related mutations in miRNASNP-v3, which also appear in MiRGeneDB 2.1. As elsewhere, mutations are ranked by these criteria, and the area under the receiver operator characteristic curve (*A*_ROC_) is used to establish whether they predict disease-associated mutations in the miRNASNP-v3 data set, split into mutations associated with cancer and those with other diseases. When checking for significance, we calculate the Mann-Whitney *p*-value and set the significance threshold to 0.05; results with *p*-values above this threshold are in *italics.*

### Disease-related mutations change the secondary structures of a specific set of individual miRNAs

We next apply the criteria formulated above to data for mutations in individual miRNAs. We use data from the miRNASNP-v3 database for both cancers and other traits and diseases, and we focus on the two criteria that work best for the larger data sets—one based on the change in the probability that the miRNA is completely unfolded (Δ*p*_unfolded_) and the other based on the normalized average Hamming distance from the WT (⟨*d*_Hamming_⟩*L*^−1^). Note that at the level of the individual miRNA, ⟨*d*_Hamming_⟩*L*^−1^ and the miRNA-specific percentile of ⟨*d*_Hamming_⟩ produce identical rankings. We observe a signal in Δ*p*_unfolded_ (*p* < 0.05) for 44 miRNAs, listed in Table S17 (see supporting material), and ⟨*d*_Hamming_⟩*L*^−1^ for 48, listed in Table S18, and for 13 miRNAs *p* < 0.05 for both criteria. Since we observe that multiple criteria perform better than random for some miRNAs, we also combine the *p*-values obtained from independent tests using Fisher’s method (52). We do that for Δ*p*_unfolded_ and ⟨*d*_Hamming_⟩*L*^−1^, and we give the results for miRNASNP-v3 in Table S19 of the supporting material. We observe a signal in 46 miRNAs from this set, including seven miRNAs for which the combined *p*-value is below 0.05, whereas the individual Mann-Whitney *p*-values are not. Intriguingly, we observe that the distributions of *A*_ROC_ within the sets of miRNAs for which these two criteria perform better than random exhibit signs of bimodality, with clear peaks around 0 and 1 and few, if any, points in between them. We quantify this observation by calculating the bimodality coefficients of the distributions according to (53), and we obtain the values 0.714 for Δ*p*_unfolded_ and 0.745 for ⟨*d*_Hamming_⟩*L*^−1^. Coefficient values above 5/9 suggest bimodality (53,54). A possible explanation of this result is that the WTs of miRNAs clustered around one peak contribute to the initiation and/or progression of disease, whereas those around the other peak are essential for disease-prevention, e.g., because they act as tumor suppressors. In that case, if miRNA folding affects function, we would expect that the mutants most strongly associated with disease in disease-suppressing miRNAs would be those that change the secondary structure the most, which would translate into *A*_ROC_ >0.5 for Δ*p*_unfolded_ and *A*_ROC_ <0.5 for ⟨*d*_Hamming_⟩*L*^−1^. The comparatively small overlap between the miRNAs for which Δ*p*_unfolded_ and ⟨*d*_Hamming_⟩*L*^−1^ perform significantly better than random suggests that some miRNAs need to be fully unfolded to perform their functions, whereas for others the folding itself plays a functional role.

When performing a large number of statistical tests, as we do in the current section for hundreds of miRNAs, a *p*-value of <0.05 is not a sufficient indicator of statistical significance. For this reason, in addition to testing whether Δ*p*_unfolded_ fulfills the Mann-Whitney test for statistical significance for individual miRNAs, we estimate the positive false discovery rate (pFDR), which is the FDR in case there is at least one positive finding. We then calculate the *q*-value, an FDR-based measure of significance equal to the minimum positive false discovery rate at which a test with *p*-value *p*_i_ is considered significant. We compute the Benjamini-Hochberg linear step-up procedure as implemented in the built-in MATLAB R2022b function mafdr (55). We consider various levels of filtering by *N*_mut seed_ and *N*_mut non__-__seed_ since we expect that a large number of recorded disease-related mutations for a particular miRNA implies a stronger association between the latter miRNA and disease. Moreover, focusing on a smaller number of miRNAs makes it possible to obtain lower *q*-values as it requires fewer tests. The number of unique sequences with mutations outside the seed is 452 at *N*_mut seed_ ≥ 1 and *N*_mut non__-__seed_ ≥ 1, 172 at *N*_mut seed_ ≥ 2 and *N*_mut non__-__seed_ ≥ 2, and 113 at *N*_mut seed_ ≥ 3 and *N*_mut non__-__seed_ ≥ 3. For the reduced data set with miRNAs whose respective genes appear in MiRGeneDB, these numbers are as follows: 145, 29 at *N*_mut seed_ ≥ 1 and *N*_mut non__-__seed_ ≥ 1, at *N*_mut seed_ ≥ 2 and *N*_mut non__-__seed_ ≥ 2, and at *N*_mut seed_ ≥ 3 and *N*_mut non__-__seed_ ≥ 3. For this smaller data set, we also use finer graining in the filtering process, and we require *N*_mut seed_ ≥ 0, varying the minimum level of *N*_mut non__-__seed_. In this case, the number of unique non-seed mutants is 372 and 202 at *N*_mut non__-__seed_ ≥ 2 and *N*_mut non__-__seed_ ≥ 3, respectively.

**Figure 3:**
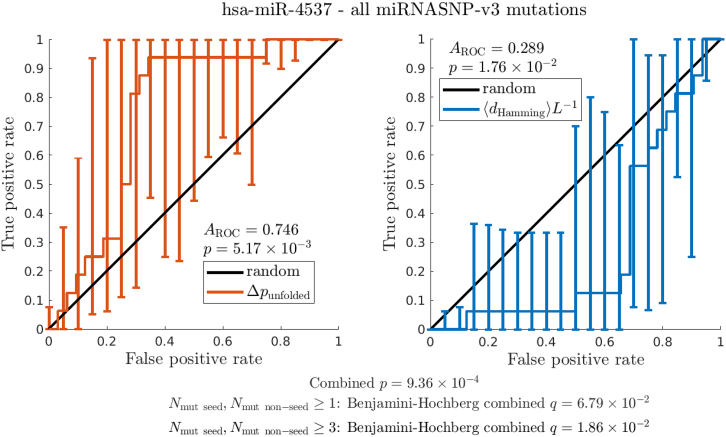
Performance of Δ*p*_unfolded_ and ⟨*d*_Hamming_⟩*L*^−1^ as predictors of disease association for mutations in the tumor suppressor hsa-miR-4537, which is known to be relevant to gastric cancer (60). Both of these metrics for the effect of point mutations on miRNA secondary structure perform significantly better than random when applied to the point mutational neighborhood of hsa-miR-4537. Note also that ⟨*d*_Hamming_⟩*L*^−1^ is equivalent to the percentile-based measure for aggregated data sets. As in the case of aggregated data sets with mutations for many miRNAs, mutations are ranked by these criteria, and the area under the receiver operator characteristic (*A*_ROC_) is used to establish whether disease-associated mutations cluster at one end of the ranking. The pointwise 95% confidence bounds for the true positive rates, indicated by error bars, are calculated via bootstrapping and vertical averaging for 21 equally spaced values of the false positive rate from 0 to 1; see (39). The *A*_ROC_ values are significantly different from the value for the random criterion (0.5) for both of these metrics. This suggests that secondary structure changes can be indicative of disease association for this particular miRNA, and in agreement with this conclusion, combining these Mann-Whitney *p*-values via the Fisher method yields *q*-values (minimum positive false discovery rates) of less than 0.05 when subjected to multiple hypothesis testing; see Table 4. According to both criteria, mutations that introduce a greater change in secondary structure tend to be associated with disease. This results in *A*_ROC_ < 0.5 for ⟨*d*_Hamming_⟩*L*^−1^ because, for consistency with Table 2, we rank mutations in “ascending” order of their associated ⟨*d*_Hamming_⟩*L*^−1^; see the methods section. This means that disease-associated mutations tend to have a greater SS Hamming distance from the WT than other mutations for this individual miRNA but a smaller one for the aggregated data sets.

Using a *q*-value threshold of 0.05, we find five miRNAs (given in Table 4) for which the association between disease-related mutations and secondary structure changes is significant (in one case, borderline). We employ two different filtering regimes, the first requiring at least one mutation in the seed region and non-seed region (*N*_mut seed_, *N*_mut non__-__seed_≥1) and the second requiring at least three mutations in each region (*N*_mut seed_, *N*_mut non__-__seed_≥3). The former covers a wider range of mutants, but this means a higher bar for significance when using the Benjamini-Hochberg procedure. The latter focuses on a smaller number of miRNAs that appear to have many disease-related mutations and are thus likely to be disease-associated. Fig. 3 is an illustration of the data underlying Table 4 for individual miRNAs with the tumor suppressor hsa-miR-4537 as an example. The figure contains ROC plots characterizing the performance of Δ*p*_unfolded_ and ⟨*d*_Hamming_⟩*L*^−1^, both of which have an associated *p*-value of less than 0.05.

**Table 4:** Benjamini-Hochberg *q*-values, i.e., minimum positive false discovery rates for which we have found a significant association between secondary structure changes and disease after accounting for multiple hypothesis testing

	Filtering levels	
miRNA	*N*_mut seed_, *N*_mut non__-__seed_≥1	*N*_mut seed_, *N*_mut non__-__seed_≥3
**hsa-miR-1269b**	2.666 × 10^−2^^∗^	–
**hsa-miR-4477b**	6.792 × 10^−2^	1.313 × 10^−2^^∗^
**hsa-miR-4537**	6.792 × 10^−2^	1.864 × 10^−2^^∗^
**hsa-miR-4641**	2.666 × 10^−2^^∗^	–
**hsa-miR-6821-3p**	>0.3	6.299 × 10^−2^

The *q*-values are based on combined Mann-Whitney *p*-values for Δ*p*_unfolded_ and ⟨*d*_Hamming_⟩*L*^−1^. Significant values (*q* < 0.05) are emphasized with an asterisk. The first two miRNAs (hsa-miR-4641 and hsa-miR-1269b) have fewer than three miRNASNP-v3 entries for mutations in the seed region or the non-seed region, and they are thus excluded by the more stringent filter (*N*_mut seed_, *N*_mut non__-__seed_≥3). The last miRNA, hsa-miR-6821-3p, is borderline significant but is nevertheless mentioned here because it becomes fully significant (*q* = 2.996 × 10^−2^) if we combine the *p*-values for Δ*p*_unfolded_ and ⟨*d*_Hamming_⟩*L*^−1^ with that for Δ*S*^(i)^. All four miRNAs have been linked with disease (56,57,58,59,60,61,62). Note that two pairs of miRNAs have equal *q*-values. The reason for this is that the *p*-values for mutants come from a small discrete set, which can yield the same *q*-value in the Benjamini-Hochberg procedure that ranks them in ascending order and multiplies them by a factor involving their rank.

We use the Fisher method to calculate a combined *p*-value for the two criteria and apply the Benjamini-Hochberg procedure to calculate the minimum false discovery at which the results are significant, i.e., the *q*-value. Applying a more stringent filter by the number of reported disease-related mutations in the mature region decreases the *q*-value because it restricts the set of miRNAs whose *p*-values are processed by the Benjamini-Hochberg method, excluding miRNAs with few mutations for which the uncertainty is greater.

Of the five miRNAs, three (hsa-miR-1269b, hsa-miR-4537, and hsa-miR-4477b) are known to play a role in cancers: hsa-miR-1269b is associated with hepatocellular carcinoma (56,57,58), a mutation in the gene coding for hsa-miR-4477b has been reported to occur during the transformation of colorectal adenoma into colorectal cancer (59), and hsa-mir-4537 is a tumor suppressor in gastric cancers (60). hsa-miR-6821-3p has been reported to be an extracellular genomic biomarker of osteoarthritis (61) and a potential biomarker for cancer (62).

One reason that only a small number of individual miRNAs are highlighted by this approach is that the analysis places a lower bound on the *p*-values. Due to the relatively small number of disease-associated mutations outside the seed region (typically one or two, versus 48 in a typical neighborhood) for many miRNAs, the lowest possible *p*-values are 4.17 × 10^−2^ (one mutant) or 1.57 × 10^−3^ (two mutants).

When we include only the higher-confidence miRNAs present in MiRGeneDB and apply the same filtering by *N*_mut seed_ and *N*_mut non__-__seed_≥1, we find no miRNAs for which *q* < 0.05. This is not surprising as the majority of these miRNAs are represented with only one or two mutations in miRNASNP-v3, and focusing on a smaller data set reduces the statistical power of our methods. However, to make the most of this limited data set, we introduce finer-grained filtering, requiring *N*_mut seed_>0 and *N*_mut non__-__seed_≥2 or *N*_mut non__-__seed_≥3. With this approach, we find *q*-values of less than 0.05 based on the combined *p*-values for Δ*p*_unfolded_, ⟨*d*_Hamming_⟩*L*^−1^, and Δ*S*^(i)^ of hsa-miR-485-5p and hsa-miR-1908-3p (see Table 5). hsa-miR-485-5p is reported to be relevant to disease, being an inhibitor of breast cancer progression according to Ref. (63). hsa-miR-1908-3p is potentially relevant to male infertility as it is reported to regulate self-renewal and apoptosis of human spermatogonial stem cells (64). We should also note that, since we perform fewer tests for individual miRNAs, a type II error—incorrectly concluding that a predictor performs significantly better than random—is less likely than when we analyze the data for all individual miRNAs present in miRNASNP-v3. Thus, although the *p*-values of the miRNAs that also appear in MiRGeneDB remain unchanged, their corresponding *q*-values are lower than when working with the full data set.

**Table 5:** Benjamini-Hochberg *q*-values, i.e., minimum positive false discovery rates, for high-confidence miRNAs (encoded by genes present in MiRGeneDB) in which we observe a significant association between changes in secondary structure and disease after accounting for multiple hypothesis testing

	Filtering levels	
miRNA	*N*_mut seed_≥0,*N*_mut non__-__seed_≥2	*N*_mut seed_≥0,*N*_mut non__-__seed_≥3
**hsa-miR-485-5p**	5.331 × 10^−2^	2.146 × 10^−2^^∗^
**hsa-miR-1908-3p**	6.516 × 10^−2^	2.623 × 10^−2^^∗^

The *q*-values are based on combined Mann-Whitney *p*-values for Δ*p*_unfolded_, ⟨*d*_Hamming_⟩*L*^−1^ and Δ*S*^(i)^. Significant values (*q* < 0.05) are emphasized with an asterisk.

### Disease-related point mutations may convert one miRNA WT into another

**Figure 4:**
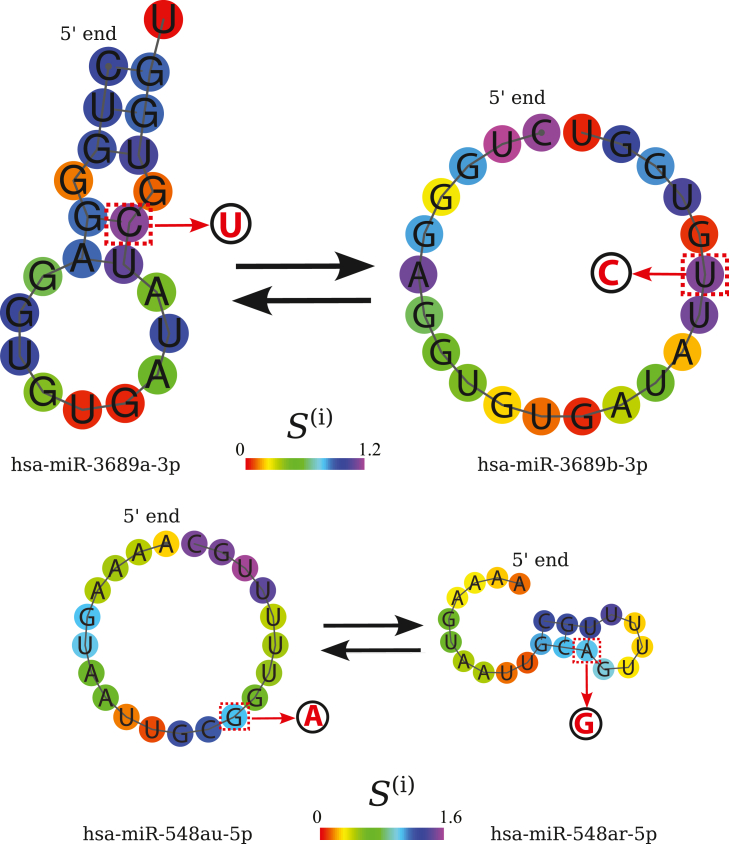
Single-point substitution mutations that convert one miRNA to another. Top: substituting the C in position 17, which has the highest predicted positional entropy (*see color and colorbar*) in the MFE structure, changes hsa-miR-3689a-3p into hsa-miR-3689b-3p. As the mutation converts a Watson-Crick GC pair into a GU wobble pair, the MFE structure changes from stably folded to fully unfolded. Both the forward and the reverse mutations are associated with disease in miRNASNP-v3. Bottom: substitution of G in position 13 with A creates an AU Watson-Crick pair, changing the MFE structure from fully unfolded to stably folded. These MFE predictions and visualizations are based on tools from the ViennaRNA suite (37).

Interestingly, the miRNASNP-v3 database contains several point mutations that convert one miRNA WT into another—we illustrate this with two examples in Fig. 4. A striking example is the mutation of C at position 17 to G, which turns hsa-miR-3689a-3p to 3689c or 3689b-3p, the latter two having the same mature sequence. This mutation, which occurs at the site with the highest positional entropy, converts a GC Watson-Crick pair to a wobble pair and turns the relatively stable MFE fold of hsa-miR-3689a-3p to a fully unfolded structure. Position 17 is outside the seed region and the supplementary region between nt 13 and 16 that is known to contribute to target recognition for some miRNAs (9,10). Thus, the mutation probably does not affect the miRNA-mRNA target complementarity, and it appears likely that the drastic change in secondary structure affects miRNA activity. Moreover, the reverse mutations, which change hsa-miR-3689c or 3689b-3p to hsa-miR-3689a-3p, are also reported to be associated with disease in miRNASNP-v3. miRNAs from the hsa-mir-3689 family have been shown to be differentially expressed in conjunctival malignant melanoma, a rare form of cancer (65).

Another mutation (ID: rs138894217) that converts one miRNA into another and dramatically changes its fold is the substitution of A to G at position 13 in hsa-miR-548ar-5p, which turns it into hsa-miR-548au-5p. It turns an AU Watson pair into a GU wobble pair, making the MFE structure fully unfolded in contrast with the stably folded WT. Although mutation rs138894217 is associated with heel bone mineral density and body mass index according to miRNASNP-v3, we were not able to find information about it in the references cited therein. This points to another possible confounder in our study: some of the associations with disease that we attempt to predict here may be simply due to mistakes in the database we source them from.

A disease-associated mutation of G at site 13 in hsa-miR-1269a to A, which changes a UG wobble pair to a UA Watson-Crick pair, changes the stability of the MFE structure and turns the sequence into that of 1269b. Although the mutant and the WT have the same MFE fold, the ensemble is substantially changed, with a normalized average Hamming distance between the mutant and the WT of ⟨*d*_Hamming_⟩*L*^−1^ = 0.35. Site 13 is in the supplementary region and may contribute to mRNA target recognition. This particular mutation (rs73239138) is known to be associated with various types of cancer, e.g., hepatocellular carcinoma (56,57,58).

We provide data on all mutations of this type that we have identified in Table S20, which contains information on the WT and mutated sequences and secondary structure, as well as the ⟨*d*_Hamming_⟩*L*^−1^ and Δ*p*_unfolded_ for them.

One may speculate that these point mutations subtly tune the activity of the miRNAs through secondary structure changes, but testing this hypothesis would require detailed information on the targets of each miRNA species. Moreover, as seen in Ref. (11), complementary interactions outside the canonical seed region may also contribute to target recognition. An additional complication is that not all genes encoding for these miRNA pairs are annotated as high confidence in MiRGeneDB, which makes it difficult to ascertain the significance of the pairs related through point mutations due to issues with miRNA identification, particularly false positives due to reads of fragments of other RNAs (49).

### AlphaFold 3 provides tentative evidence for mature miRNA secondary structure within RISC

**Figure 5:**
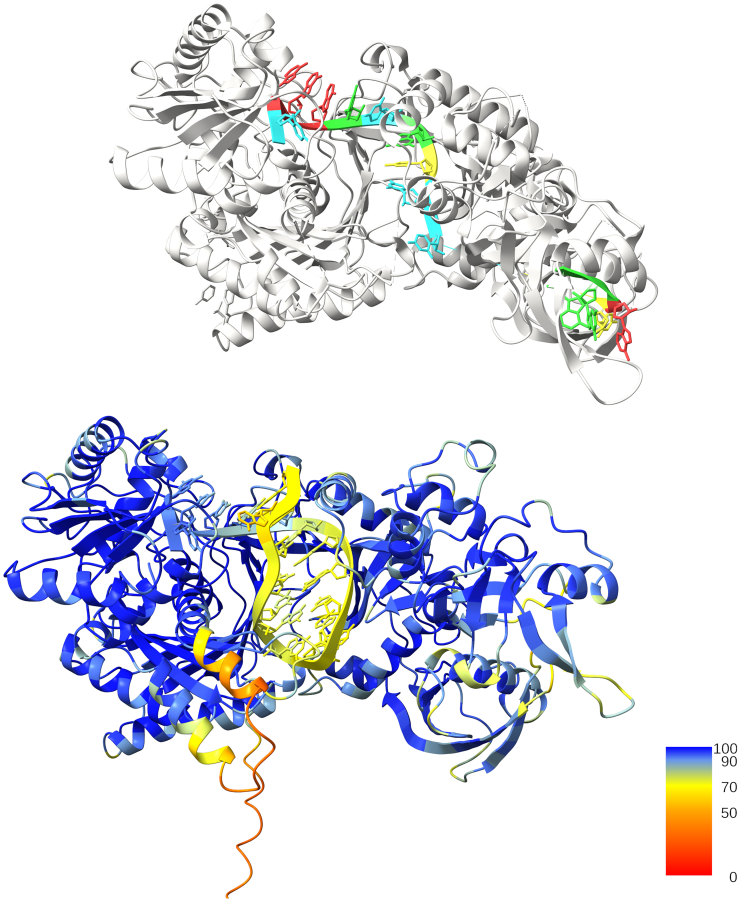
AlphaFold 3 predicts non-trivial secondary structure in the complex of a point mutant of hsa-miR-6869-3p and Argonaute 2. Crystal structure of human Argonaute-2 protein in complex with miR-20a (PDB: 4F3T, *top*) and AlphaFold 3 predictions for the non-seed point mutant of hsa-miR-6869-3p with the maximum ⟨*d*_Hamming mutant_⟩ in complex with human Argonaute-2 (*bottom*) are shown. RNA is depicted as ribbons with bases indicated; note the base-pairing in the predicted structure. All residues in the AlphaFold 3 image are colored by plDDT, a measure of the confidence of the prediction, as indicated by the color bars. Most of the miRNA structure is predicted with high confidence (70 < plDDT < 90, average: 76.4), and the predictions show base-pairing within the miRNA. The crystal structure shows no structural information about nucleotides 11 to 16. Images were created with UCSF ChimeraX version 1.09 (69).

As we note in the introduction, the canonical view of mature miRNA function is that it is carried out within RISC, which has led to the assumption that miRNAs do not have secondary structure. However, as mentioned above, secondary structure is known to form in some RNA-protein complexes, notably in the influenza virus (28). Moreover, we found that the state-of-the-art tool for predicting ribonucleoprotein tertiary and quaternary structure tool AlphaFold 3 (66) indicates that two hsa-miR-6869-3p mutants, U12/G and C8/A, are folded when bound to Argonaute 1 and 2 proteins (protein sequences taken from PDB entries 4KXT and 4F3T, respectively). We show the predicted structure for one of them alongside a PDB crystal structure of a miRNA-Argonaute 2 complex in Fig. 5; we show the equivalent for the miRNA-Argonaute 1 complex in the supporting material (Fig. S23).

Although AlphaFold 3 and other machine-learning algorithms for predicting the structure of ribonucleoproteins are useful tools, their development is at a relatively early stage (67), and their accuracy and applicability are limited by the relatively low coverage of RNA structures in databases such as PDB (see, e.g., (68)). An additional drawback is that the generative AI model underpinning AlphaFold 3 tends to predict structure even in unstructured protein regions (66). The predictions for Argonaute in complex with the C8/A mutant of hsa-miR-6869-3p also included a low-confidence helix in the protein, which is unlikely to be present in the real structure. For these reasons and due to the large computational expense of folding the tens of thousands of mutants we study in our work with a tool such as AlphaFold 3, we restrict our analysis to mature miRNA secondary structure. miRNA folding within the RISC complex is probably indeed restricted as per the canonical picture, but miRNA secondary structure may still be relevant to disease through the many non-canonical and poorly studied alternative miRNA pathways (31,32).

Additionally, we predicted the tertiary structure of complexes between Argonaute 2 and the WTs or the top-scoring mutants of the miRNAs that show significant association between secondary structure changes and disease and are not ruled out by multiple hypothesis testing. Out of these, only the hsa-miR-4537-3p mutant with maximum Δ*p*_unfolded_ was predicted to have a nontrivial fold, but with a much lower confidence (average plDDT: 65.3) than the mutant in hsa-miR-6869-3p shown in Fig. 5 (average plDDT: 76.4). We provide tables of the predicted secondary structure and CIF files with the tertiary structures as part of the supporting material.

### Some disease-related miRNA mutations outside the seed region change the fold of the mature form but not the pre-miRNA

It is possible that mutations in mature miRNA sequences affect function through altering the secondary structure of the precursor, rather than the mature miRNA itself. We study this possibility through generating predictions for the secondary structure of the pre-miRNAs of all disease-related mature miRNA mutant sequences that we considered in our study. We then characterize the differences between those mutated pre-miRNAs and their respective WTs. First, we calculate the average Hamming distance in secondary structure between mutant and WT. Comparing the distributions, which we plot in Fig. S21 in the supporting material, shows that they do not differ significantly (two-sided Mann-Whitney U test: *p* = 0.33 for miRNASNP3), which indicates that disease-associated point mutations induce comparable absolute secondary structure changes in mature and precursor miRNAs.

In addition, we calculate how the disease-related mutations affect the predicted base-pairing probabilities changed within the pre-miRNA regions that other authors have identified as important to pre-miRNA processing (70). Roden et al. found that the region at distance 5–9 nt from the base of the pre-miRNA hairpin tends to be enriched with bulges, whereas the regions at distance 16–21 nt and 28–32 nt tend to be bulge-depleted. We calculate how each disease-related mutation changes the probability that each of these positions is paired with respect to the pre-miRNA WT. We then calculate the maximum absolute value of this change, which highlights whether the pairing of any individual site has been strongly affected. This is a useful measure as bulges in individual sites have previously been reported to cause disease; see the examples of variants in miR-502 (19) and miR-125a (20) that we refer to in the introduction. Together with the maximum change of base-pairing probability for the pre-miRNA sites sensitive to secondary structure variations identified by Roden et al., we calculate the same across all sites within the mature miRNA mutants.

We then subtract the maximum local change in base-pairing probability in each pre-miRNA mutant from that of the respective mature mutant. This difference, which we denote Δ_max_, is 1 if a pre-miRNA mutation changes one or more sites from completely paired to completely unpaired or vice versa, but the same mutation leaves all sites in the mature form unchanged. Analogously, Δ_max_ = −1 if one or more sites are changed from completely paired to completely unpaired in the mature form but are unchanged in the pre-miRNA. The distribution of Δ_max_ for miRNASNP3 data, which we plot in Fig. S22 in the supporting material, contains a number of mutations that induce large changes in the pairing of sites in the mature form but practically no change in the pre-miRNA. 219 such mutants have Δ_max_ ≤ −0.5, and 39 have Δ_max_ ≤ −0.9. This means that some disease-related mutants do not affect the secondary structure at sites important for pre-miRNA processing but only change the folding of the mature form, suggesting that mature miRNA secondary structure also plays a role in the diseases covered by miRNASNP3 data.

## Discussion

This computational study explores the role of secondary structure in mature miRNA mutants based on literature data for associations between such mutants and disease. We formulate different quantitative measures for the difference between a given mutant and its respective WT and test their ability to predict disease association.

Our results for the data in miRNASNP-v3 indicate a significant association between disease and mutations that increase the probability that a given mature miRNA is unfolded. This is in contrast to the work of Diederichs and Haber (17), who found that mutations that significantly changed pri-miRNA secondary structure had no effect on their processing and maturation. Moreover, we see a significant effect of the change in the pairing of mutated sites across the miRNASNP-v3 database as measured via the positional entropy difference at the mutated site, Δ*S*^(i)^. When we apply our criteria for mutation-induced secondary structure changes to the data for individual miRNAs, we observe a particularly strong relationship between secondary structure and disease for several miRNAs. This analysis combines *p*-values for the Δ*pun*_unfolded_ and ⟨*d*_Hamming_⟩/*L* metrics using the Fisher method and employs the Benjamini-Hochberg method for multiple hypothesis testing. We furthermore filter the data according to the number of mutations recorded in the seed region and outside the seed region. The miRNAs that emerge are hsa-miR-1269b, hsa-miR-4537, hsa-miR-4477b, hsa-miR-4641, and hsa-miR-6821-3p, which include three miRNAs that are known to play a role in cancers (56,57,58,59,60).

We address the issue of possible misidentification of other RNAs as miRNAs by performing the same analysis on the subset of mutations from miRNASNP-v3 that concern miRNAs appearing in the manually curated database MiRGeneDB 2.1 (51). Despite the reduced statistical power due to the smaller size of the data set, one of our measures for characterizing the effect of secondary structure is again significantly associated with disease. On the level of individual miRNAs, in this case, we identify that secondary structure changes are associated with disease in the miRNAs hsa-miR-485-5p and hsa-miR-1908-3p.

We need to emphasize that, although we aim to exclude clearly incorrect entries by performing cross-checks between data from mutation databases such as miRNASNP and reference data sets such as miRBase, HG38, and MiRGeneDB, it is not our aim here to curate a data set of mutations in miRNAs or to probe the association of these mutations with disease. Instead, we have devised a method that, given a set of such disease-associated mutations, characterizes whether they tend to be associated with changes in secondary structure. Although an imperfect approach, this addresses our aim of characterizing the effect of mature miRNA secondary structure on miRNA activity. It would also be straightforward to apply the algorithms we have formulated here to a better-curated data set with miRNA mutations should one become available in the future.

It is likely that the significance criteria are not met for many other miRNAs because of a combination of factors: 1) the small number of mutations recorded for most miRNAs and 2) the different nature of the mutations in the database. Most of the disease-associated mutations in miRNASNP-v3 have been identified via methods such as genome-wide association studies, which do not establish a causative relationship with disease, meaning that some of these mutations are likely to simply accompany disease. Another possible confounder in our current study are mistakes in the assignment of disease association to mutations, of which we give an example in the previous section (SNP rs138894217).

Quantitative experimental data on the activity of various point mutants of a set of miRNAs would shed light on these matters. Attempts at quantifying the effect of mutations in non-coding RNAs, including for the case of such mutations that reduce the risk of disease, are collected in the ncRNAVar database by Zhang et al. (71). This database uses weighted scores to combine different forms of evidence in the literature rather than quantitative experimental evidence, and it covers a smaller number of mutations than miRNASNP-v3.

## Conclusion

As we note in the introduction, it is difficult to establish the role of mature miRNA secondary structure because the mature form of the molecule is typically incorporated in RISC, and furthermore, even mutations in the mature sequence could affect miRNA function through changing the folding and maturation of its precursor, rather than through changing the secondary structure of the mature miRNA itself; see the example of miRNA-125a in Ref. (20) and others in Ref. (72). Moreover, the same mutation can simultaneously be associated with a decreased risk of one disease and an increased risk of another, as in the example of the rs291016 polymorphism (21) that we gave in the introduction. This is likely a consequence of the complex networks of genes regulated by each miRNA, as well as potential non-canonical pathways for some of them. The fact that some of our measures of secondary structure change perform better than random may be due to different mechanisms of miRNA activity that depend on specific secondary structure features. At the same time, there is uncertainty in our conclusions that is inevitable given that the data from mutation databases include multiple possible confounders, as we discuss above.

With all this in mind, a definitive test of our hypothesis that miRNA secondary structure can have significant influence on function requires experimental data on the regulatory activity of mutants, preferably in the form of gene suppression activity for the point mutants derived from several mature miRNAs (with the seed region fixed). Based on our analysis, hsa-miR-485-5p and hsa-miR-1908-3p are the most promising candidates for such studies. The availability of such experimental data would allow for the further refinement of our methods for predicting which mutations are associated with disease, particularly cancer, and thus yield a potentially valuable diagnostic tool.

## Acknowledgments

This work was supported by the 10.13039/501100004815Isaac Newton Trust (NQAG/341) and was performed using resources provided by the Cambridge Service for Data Driven Discovery (CSD3) operated by the University of Cambridge Research Computing Service (www.csd3.cam.ac.uk), provided by Dell EMC and Intel using Tier-2 funding from the 10.13039/501100000266Engineering and Physical Sciences Research Council (capital grant EP/T022159/1), and DiRAC funding from the 10.13039/501100000271Science and Technology Facilities Council (www.dirac.ac.uk). J.K.N. acknowledges funding from the 10.13039/501100000265MRC (grant MC_FE_00035) Cross Disciplinary Fellowship (XDF) Program. A preliminary version of this work, https://doi.org/10.1101/2024.06.19.599688, was deposited in biorXiv on June 22nd, 2024. For the purpose of open access, the authors have applied a Creative Commons Attribution (CC BY) license to any Author Accepted Manuscript version arising from this submission.

## Author contributions

J.K.N.: conceptualization, software, data curation, formal analysis, investigation, methodology, writing—original draft, and writing—review and editing; S.E.A.: funding acquisition, conceptualization, software, formal analysis, methodology, and writing—review and editing.

## Declaration of interests

The authors declare no competing interests.

## Supporting material

Document S1. Figures S1–S23 and Tables S1–S24

Document S2. Article plus supporting material
