# Supplementary material for: Disease-related miRNA mutations are associated with mature miRNA secondary structure changes
Source: Biophys J. 2025 Oct 3;124(23):4141–56. doi: 10.1016/j.bpj.2025.09.049 (PMC12709415; doi:10.1016/j.bpj.2025.09.049)
Supplement: Document S1. Figures S1–S23 and Tables S1–S24 [file mmc1.pdf]

Biophysical Journal, Volume *124*

Supplemental information

Disease-related miRNA mutations are associated with mature miRNA secondary structure changes

Javor K. Novev and Sebastian E. Ahnert

Supplementary Information to Disease-related miRNA mutations are associated with mature miRNA secondary structure changes

Javor K. Novev*[1,2] and Sebastian E. Ahnert†[1,3]

[1]Department of Chemical Engineering and Biotechnology, University of Cambridge, Philippa Fawcett Drive, Cambridge CB3 0AS, UK
[2]Institute of Genetics and Cancer, The University of Edinburgh, Western General Hospital, Crewe Road, Edinburgh EH4 2XU, UK
[3]The Alan Turing Institute, 96 Euston Road, London NW1 2DB, UK

Contents

1 SomamiR database **2**

2 Additional metrics of the effect of mutations on secondary structure **2**
2.1 Change in the probability of the unfolded states with respect to the WT 3
2.1.1 Change in the probability of the fully unfolded state with respect to the WT 3
2.1.2 Change in the probability that the seed region is unfolded with respect to the WT . . 3
2.2 Hamming-distance-based criteria 3
2.2.1 Normalized Hamming distance from the WT, $\langle d_{\mathrm{Hamming}} \rangle L^{-1}$ 3
2.2.2 $\langle d_{\mathrm{Hamming}} \rangle$ percentile 3
2.3 Positional-entropy-based criteria 6
2.3.1 Change of the positional entropy of the mutated site with respect to the WT 6
2.3.2 Mutant average positional entropy 6
2.3.3 Change of the average positional entropy with respect to the WT 6

3 Tables and graphical comparisons of measures of criteria performance **25**

4 Rank correlations between selected criteria **39**

5 Tables of p-values and other data for individual miRNAs **40**

6 Table of disease-associated mutations that convert one WT miRNA to another **45**

7 Case studies **46**
7.1 hsa-miR-4537 46
7.2 hsa-miR-485-5p 46

8 Additional information on miRNAs with $q < 0.05$ **49**

*Corresponding author: ynovev@ed.ac.uk
†Corresponding author: sea31@cam.ac.uk

9 **Distributions of $\langle d_{\text{Hamming}} \rangle$ for disease-related mutations in mature miRNAs and their precursors** **52**

10 **Distributions of $\Delta_{\max}$ for disease-related mutations** **53**

11 **AlphaFold3 studies of miRNA-Argonaute complexes** **54**

12 **Code and data** **57**

Here we provide information on the criteria for relationship with disease that we only mentioned in the main text, additional ROC curves for the different predictors, plots that compare A_{ROC} for different criteria under different levels of filtering, and tables with A_{ROC} and p-values and sample sizes.

1 SomamiR database

In addition to the miRNASNP-v3 dataset discussed in the main text, we evaluate the performance of our metrics on the SomamiR dataset of miRNA mutations encountered in cancer [1]. After cross-checking the SomamiR data with HG38 and miRBase, the number of mature miRNA mutations outside the seed recorded in it is 439, with 363 unique sequences derived from 285 WT miRNAs, and the number of seed-region mutations is 173. Filtering to include only miRNAs with $N_{\text{mut seed}} \geq 1$ and $N_{\text{mut non-seed}} \geq 1$ leaves 81 mutations outside the seed region, representing 66 unique sequences and affecting 40 miRNAs.

2 Additional metrics of the effect of mutations on secondary structure

We formulated several other criteria that measure the effect of non-seed point mutations on miRNA secondary structure, and give a list of them below. We do not describe them or give details on their performance in the main text since the latter is inferior to that of the three we discuss therein.

a) The change in the probability that the seed region is fully unfolded, $\Delta p_{\text{unfolded seed}} = p_{\text{unfolded seed mutant}} - p_{\text{unfolded seed WT}}$. As the seed is key for target recognition, one may expect that a high value of $\Delta p_{\text{unfolded seed}}$ would strongly affect the activity of a miRNA.

b) The average positional entropy of a mutant, $\langle S_{\text{mut}} \rangle$, which measures the stability of a fold. We defined additional criteria related to $\langle S_{\text{mut}} \rangle$ as follows.

– The absolute mutant positional entropy, $\langle S_{\text{mut}} \rangle$.

– The difference in positional entropy between mutant and WT, $\langle \Delta S \rangle = \langle S_{\text{mut}} \rangle - \langle S_{\text{WT}} \rangle$. We apply this criterion in two different ways: **i)** we test how well it predicts association with disease for data for all miRNAs in a dataset or **ii)** by splitting datasets into subsets where the SS stability of mutant and WT can be compared via $\langle \Delta S \rangle$ because the subset only includes mutants and WTs that are **a)** both folded or **b)** both unfolded.

c) Tree editing distance d_{tree} as calculated by the RNAdistance program from the ViennaRNA suite [2]. Calculating the average of d_{tree} over the Boltzmann ensemble is too costly since we examine a set of ~ 500 secondary structures for each studied sequence. This requires $\sim 500^2$ comparisons between each pair of WT and mutant; when applied to the set of $\sim 4 \times 10^4$ mutants we consider, the computational cost of this would be orders of magnitude greater than that for conducting the rest of the study.

Due to the computational cost of calculating the average value of this distance across mutant and WT thermodynamic ensembles, we compute only the distance between the MFE structure of each mutant and WT and normalize it by its maximum possible value, $d_{\text{tree}} = d_{\text{RNAdistance}}/(2L)$.

In building the ROC curves and analyzing the performance of these criteria, we use the same approach described above for $\Delta p_{\text{unfolded}}$ - we calculate the criterion values for a set of mutants, then rank the latter in *descending order* according to that criterion. Next, we ascertain whether the criterion predicts association with disease better than random by building a ROC curve, calculating the area under it (A_{ROC}) and checking whether the associated p-values meet our significance criteria. We provide more details on the criteria and their performance below.

2.1 Change in the probability of the unfolded states with respect to the WT

2.1.1 Change in the probability of the fully unfolded state with respect to the WT

Additional ROC curves for $\Delta p_{\text{unfolded}} = p_{\text{unfolded mutant}} - p_{\text{unfolded WT}}$ are shown in Figures S1-S2.

2.1.2 Change in the probability that the seed region is unfolded with respect to the WT

As the miRNA seed region is crucial to miRNA-mRNA binding, we expect that mutations which change its folding have a significance in disease. Specifically, we test whether disease-related mutations change the probability that the seed region contains no base-pairing more than other possible point mutations. To calculate the change in probability for no seed-region base-pairing, we impose the constraint that no bases within the seed region are paired, calculate the free energy of the constrained ensemble $F_{\text{constrained}}$ and use the equation

$$p_{\text{unfolded seed}} = \exp\left(-\frac{F_{\text{constrained}} - F_{\text{unconstrained}}}{RT}\right), \tag{S1}$$

where $F_{\text{unconstrained}}$ is the free energy of the ensemble without any imposed constraints.

Having calculated $p_{\text{unfolded seed}}$ for all mutants in the point-mutational neighborhood of the WT except those that have an altered seed region, we then calculate $\Delta p_{\text{unfolded seed}} = p_{\text{unfolded seed mutant}} - p_{\text{unfolded seed WT}}$. We then use this quantity as a criterion for ranking these mutants and build an ROC curve in order to assess its power to predict disease-related mutations (Figure S3).

The change in the probability that the seed region is unfolded, $\Delta p_{\text{unfolded seed}}$, does not perform significantly better than random for any of the datasets that we studied, suggesting that the likelihood that the seed region is unfolded does not play an important role in determining miRNA activity.

2.2 Hamming-distance-based criteria

2.2.1 Normalized Hamming distance from the WT, $\langle d_{\text{Hamming}}\rangle L^{-1}$

We formulate another criterion based on the average Hamming distance $\langle d_{\text{Hamming mutant}}\rangle$ from the WT which registers smaller differences in secondary structure than the percentile-based one discussed in the main text. To do this, we normalize the average Hamming distance by the miRNA length L, $\langle d_{\text{Hamming}}\rangle L^{-1}$. We then test whether disease-related mutants from SomamiR and miRNASNP-v3 tend to change miRNA secondary structure more than other mutants by ranking mutants in *ascending order* of their $\langle d_{\text{Hamming mutant}}\rangle$ and building an ROC curve for this criterion (Figure S4).

The normalized Hamming distance, $\langle d_{\text{Hamming}}\rangle L^{-1}$, has A_{ROC} significantly smaller than 0.5 for the 45 miRNASNP-v3 entries related to diseases other than cancer for which $N_{\text{mut seed}} \geq 1$ and $N_{\text{mut non-seed}} \geq 1$. Since we order mutants in ascending order of $\langle d_{\text{Hamming}}\rangle L^{-1}$, this means that mutants with a larger $\langle d_{\text{Hamming}}\rangle L^{-1}$, which rank lower, tend to be associated with disease.

2.2.2 $\langle d_{\text{Hamming}}\rangle$ percentile

Additional ROC curves for data from miRNASNP-v3 are shown in Figure S6.

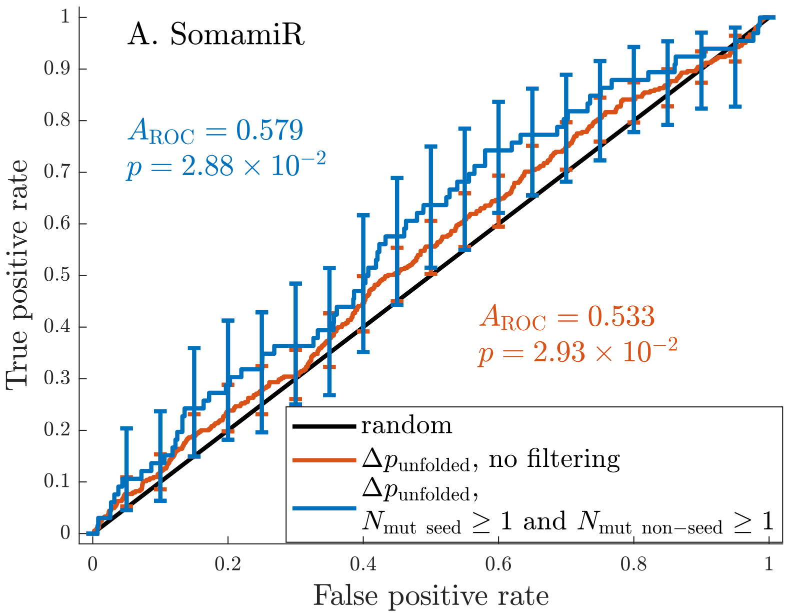
A. SomamiR
$A_{\mathrm{ROC}} = 0.579$
$p = 2.88 \times 10^{-2}$
$A_{\mathrm{ROC}} = 0.533$
$p = 2.93 \times 10^{-2}$
random
$\Delta p_{\mathrm{unfolded}}$, no filtering
$\Delta p_{\mathrm{unfolded}}$,
$N_{\mathrm{mut\ seed}} \geq 1$ and $N_{\mathrm{mut\ non-seed}} \geq 1$
True positive rate
False positive rate

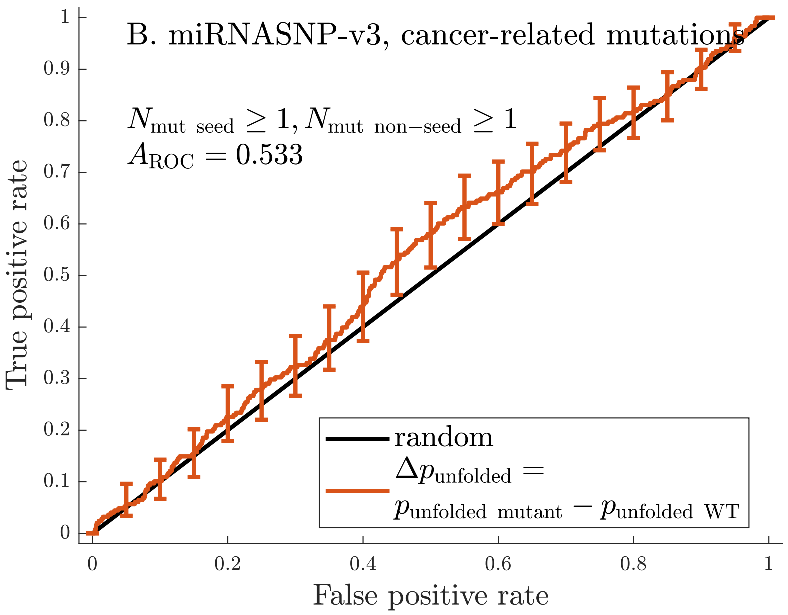
B. miRNASNP-v3, cancer-related mutations
$N_{\mathrm{mut\ seed}} \geq 1, N_{\mathrm{mut\ non-seed}} \geq 1$
$A_{\mathrm{ROC}} = 0.533$
random
$\Delta p_{\mathrm{unfolded}} =$
$p_{\mathrm{unfolded\ mutant}} - p_{\mathrm{unfolded\ WT}}$
True positive rate
False positive rate

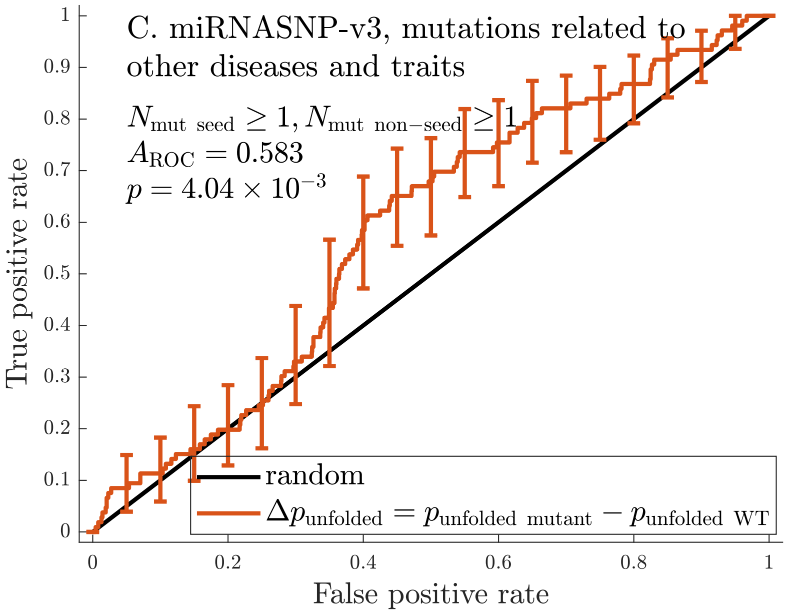

Figure S1: **The change in probability that the miRNA is fully unfolded associated with a mutation is a predictor of the mutation's relationship with disease.** ROC curves built using $\Delta p_{\rm unfolded} = p_{\rm unfolded\ mutant} - p_{\rm unfolded\ WT}$ as the criterion for predicting disease-related mutations. Data from SomamiR (A), information on cancer-related mutations (B) and mutations related to other traits and diseases (C) from miRNASNP-v3, either with no filtering based on $N_{\rm mut\ seed}$ and $N_{\rm mut\ non-seed}$ (orange curves) or with $N_{\rm mut\ seed} \geq 1$ and $N_{\rm mut\ non-seed} \geq 1$ (blue curves) for all mature miRNAs considered here. Error bars indicate pointwise 95% confidence bounds calculated at 21 equally spaced points with the bootstrapping method [3]. The area under the ROC curve ($A_{\rm ROC}$) and the Mann-Whitney p-value [4] for the curves are also indicated. The $\Delta p_{\rm unfolded}$ criterion performs significantly better than the random one for all three datasets ($p < 0.05$) if no filtering is applied, indicating that the probability that disease-related mutants are fully unfolded tends to be higher than that for other mutants. This could be because mutants with a higher $p_{\rm unfolded\ mutant}$ have a higher activity than their respective WTs, and, in the case of cancer-associated mutations, they may be more effective at downregulating tumour suppressor genes. When the analysis for each dataset is restricted to just the miRNAs for which at least one mutation in the seed region and the rest of the mature miRNA, $A_{\rm ROC}$ increases and so does the certainty that the criterion outperforms the random one, except for the set of cancer-associated mutations from miRNASNP-v3, for which we observe changes in the opposite direction.

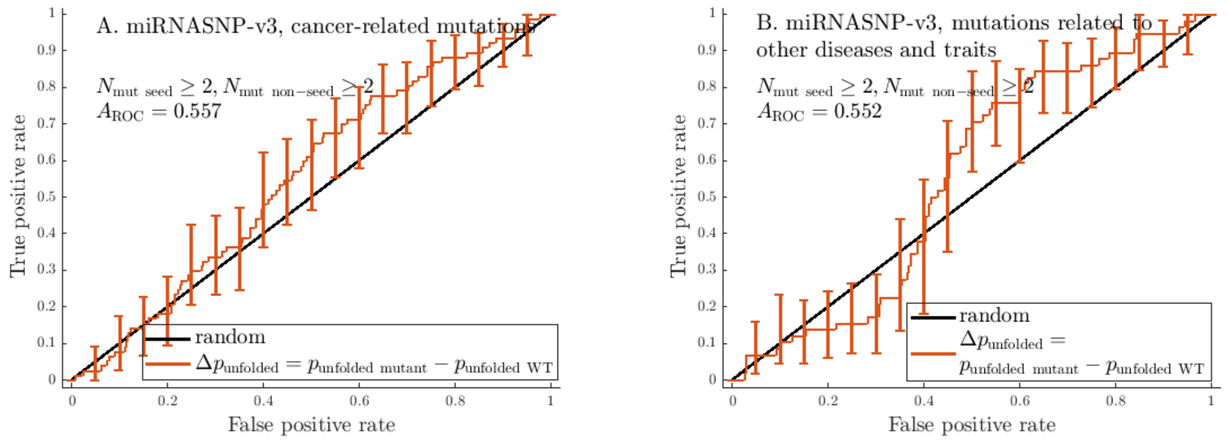

Figure S2: ROC curves built using $\Delta p_{\text{unfolded}} = p_{\text{unfolded mutant}} - p_{\text{unfolded WT}}$ as the criterion for predicting disease-related mutations. Data taken from miRNASNP-v3. Error bars indicate pointwise 95 % confidence bounds calculated at 21 equally spaced points with the bootstrapping method [3]. The area under the ROC curve (A_{ROC}) is also indicated.

2.3 Positional-entropy-based criteria

2.3.1 Change of the positional entropy of the mutated site with respect to the WT

We use the change in positional entropy $S^{(\text{i})}$ at the mutated site as defined in Eq. (3) as a criterion for predicting whether a mutation is associated with disease and present the corresponding ROC curves for various datasets in Figure S8.

2.3.2 Mutant average positional entropy

Calculating the average positional entropy, $\langle \Delta S \rangle$, for an RNA molecule from the positional entropies of the individual sites, $S^{(\text{i})}$, requires straightforward application of the definition in Eq. (**??**)

$$\langle S \rangle = -\frac{1}{L} \sum_{\text{i}=1}^{L} S^{(\text{i})}, \tag{S2}$$

where L is the number of nucleotides in the RNA molecule. The values of $\langle S \rangle$ for the mutant and the WT are comparable only if 1) the minimum-free-energy (MFE) structures both of them have secondary structure or 2) neither MFE structure has secondary structure. Note that this latter case is much less common as base-pairing is energetically favourable and usually possible. We analyze these two cases separately.

The average positional entropy of an RNA, $\langle S \rangle$ as defined above, is a measure of the stability of its fold. We use it as a criterion for predicting whether a mutation is associated with disease and present the corresponding ROC curves for various datasets in Figure S9.

$\langle S_{\text{mutant}} \rangle$, performs significantly better than random for the set of 28 cancer-related miRNAs with $N_{\text{mut seed}} \geq 2$ and $N_{\text{mut non-seed}} \geq 2$, indicating that disease-related mutants in this set tend to have a more stable fold than other mutants.

2.3.3 Change of the average positional entropy with respect to the WT

If the secondary structure of a microRNA affects its interaction with its targets, then one may hypothesize that the mutations which significantly change the stability of that structure would be the ones with the

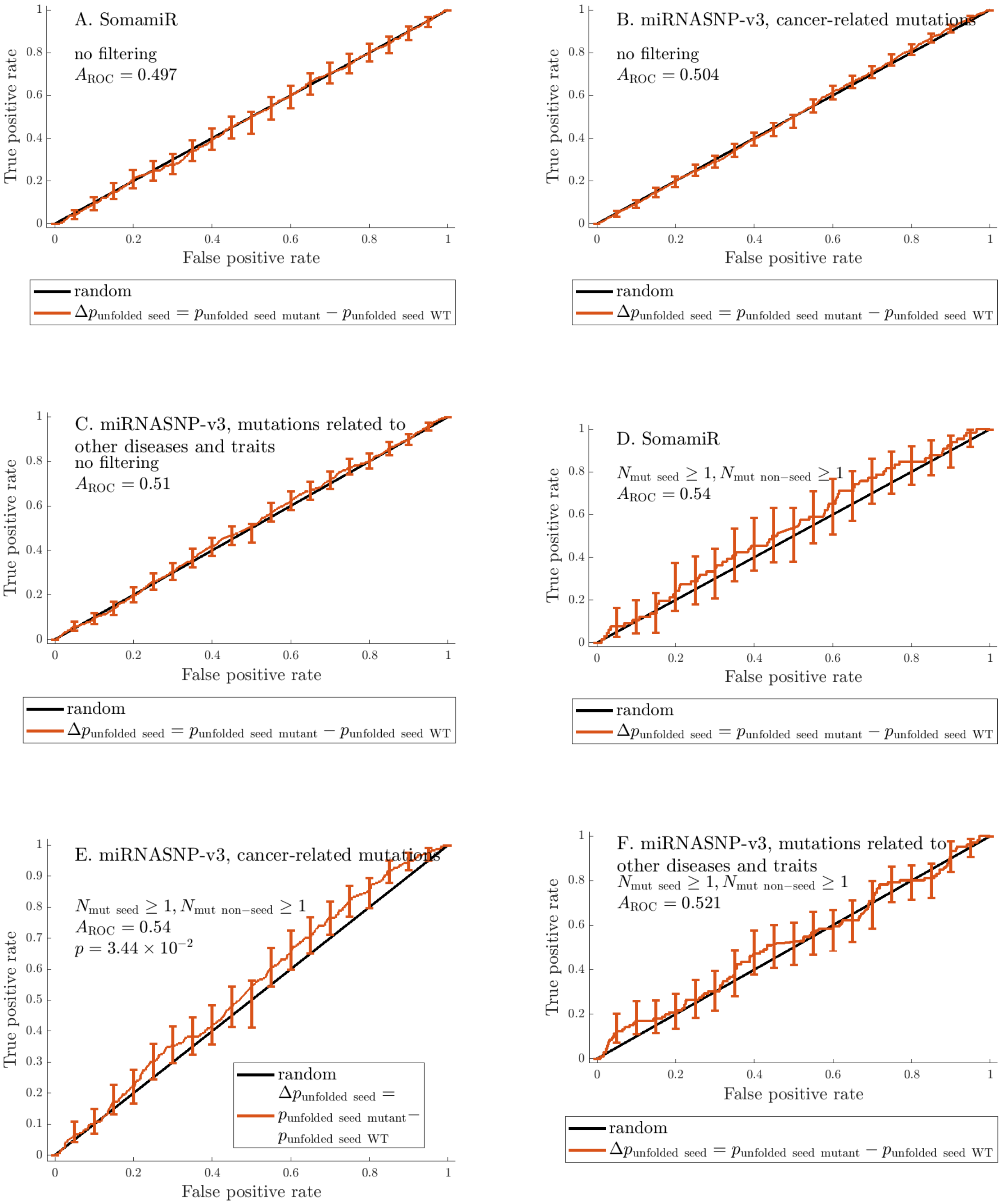
A. SomamiR
no filtering
$A_{\mathrm{ROC}} = 0.497$
B. miRNASNP-v3, cancer-related mutations
no filtering
$A_{\mathrm{ROC}} = 0.504$
C. miRNASNP-v3, mutations related to other diseases and traits
no filtering
$A_{\mathrm{ROC}} = 0.51$
D. SomamiR
$N_{\mathrm{mut\ seed}} \geq 1, N_{\mathrm{mut\ non-seed}} \geq 1$
$A_{\mathrm{ROC}} = 0.54$
E. miRNASNP-v3, cancer-related mutations
$N_{\mathrm{mut\ seed}} \geq 1, N_{\mathrm{mut\ non-seed}} \geq 1$
$A_{\mathrm{ROC}} = 0.54$
$p = 3.44 \times 10^{-2}$
F. miRNASNP-v3, mutations related to other diseases and traits
$N_{\mathrm{mut\ seed}} \geq 1, N_{\mathrm{mut\ non-seed}} \geq 1$
$A_{\mathrm{ROC}} = 0.521$
True positive rate
False positive rate
random
$\Delta p_{\mathrm{unfolded\ seed}} = p_{\mathrm{unfolded\ seed\ mutant}} - p_{\mathrm{unfolded\ seed\ WT}}$

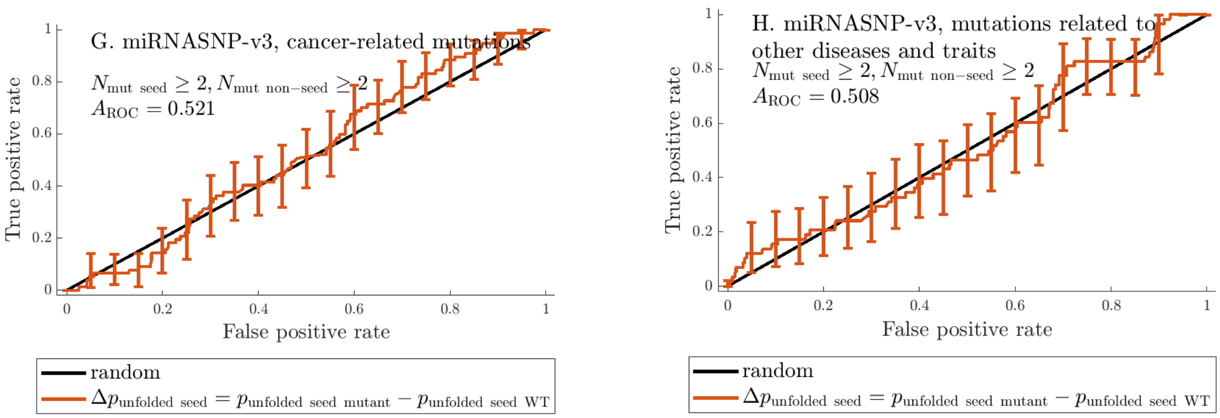

Figure S3: ROC curves built using $\Delta p_{\mathrm{unfolded\ seed}} = p_{\mathrm{unfolded\ seed\ mutant}} - p_{\mathrm{unfolded\ seed\ WT}}$ as the criterion for predicting disease-related mutations. Data taken from SomamiR and miRNASNP-v3.

greatest effect on miRNA function. We use the difference between the average positional entropy for the mutant and the WT, $\langle \Delta S \rangle = \langle S_{\mathrm{mut}} \rangle - \langle S_{\mathrm{WT}} \rangle$ as a measure of this change in stability, with low $\langle S_{\mathrm{mut}} \rangle$ indicating a stable secondary structure [5]. As a miRNA needs to bind to an mRNA in order to regulate gene expression, one may expect that the stability of its fold is relevant to its function. In particular, one may expect that mutants that are more stably folded (i.e., have lower $\langle S_{\mathrm{mut}} \rangle$) than the respective WT may be less effective at binding to mRNA and thus gene regulation, potentially leading to disease. We rank mutants according to their value of this quantity and check whether it correlates with disease association; we plot ROC curves for this criterion in Figure S10.

Strictly, the values of $\langle S \rangle$ for the mutant and the WT are comparable only if 1) the minimum-free-energy (MFE) structures both of them have secondary structure or 2) neither MFE structure has secondary structure. We analyze these two cases separately below; for comparison, we also measure the performance of the $\langle S_{\mathrm{mut}} \rangle$ without regard for whether the WT and mutants are folded.

We calculate $\langle S_{\mathrm{mut}} \rangle$ for the point mutational neighborhoods of the WT miRNAs represented in miRNASNP-v3 and SomamiR, while keeping the sequences of their seed regions (sequence positions 2-7) fixed. The change in the average positional entropy with respect to the wild type, $\langle \Delta S \rangle$, performs significantly better than random for the set of 9 miRNAs associated with diseases other than cancer for which $N_{\mathrm{mut\ seed}} \geq 2$ and $N_{\mathrm{mut\ non-seed}} \geq 2$. The p-value in this case is almost an order of magnitude higher than that for the subset of 6 miRNAs which have folded mutants and WTs, and the criterion performs no better than random for the other 3 miRNAs. This indicates that the significant effect is in the change of stability of non-trivial folds, and that, as expected, it is only appropriate to compare $\langle \Delta S \rangle$ if 1) both the mutant and the WT are folded or 2) neither is folded.

2.3.3.1 Folded mutant and WT When only miRNAs with $N_{\mathrm{mut\ seed}} \geq 2$ and $N_{\mathrm{mut\ non-seed}} \geq 2$ from miRNASNP-v3 data are considered, the change in the average positional entropy $\langle \Delta S \rangle$ becomes a useful predictor of disease association for mutants and WT that are both folded, see Figure S11. The values of A_{ROC} of 0.580 and 0.615 that we calculated for mutations related to cancer and other diseases respectively, indicate that decreasing stability is associated with disease for the clusters of 18 and 9 miRNAs in these subsets of data.

A miRNA's folding may have an effect on its function in two ways - it could either have its own functional purpose, or it could affect the interaction with the target site as it would make the binding sites within the miRNA less accessible. In the first case, a mutant may disrupt function by causing a change to the MFE

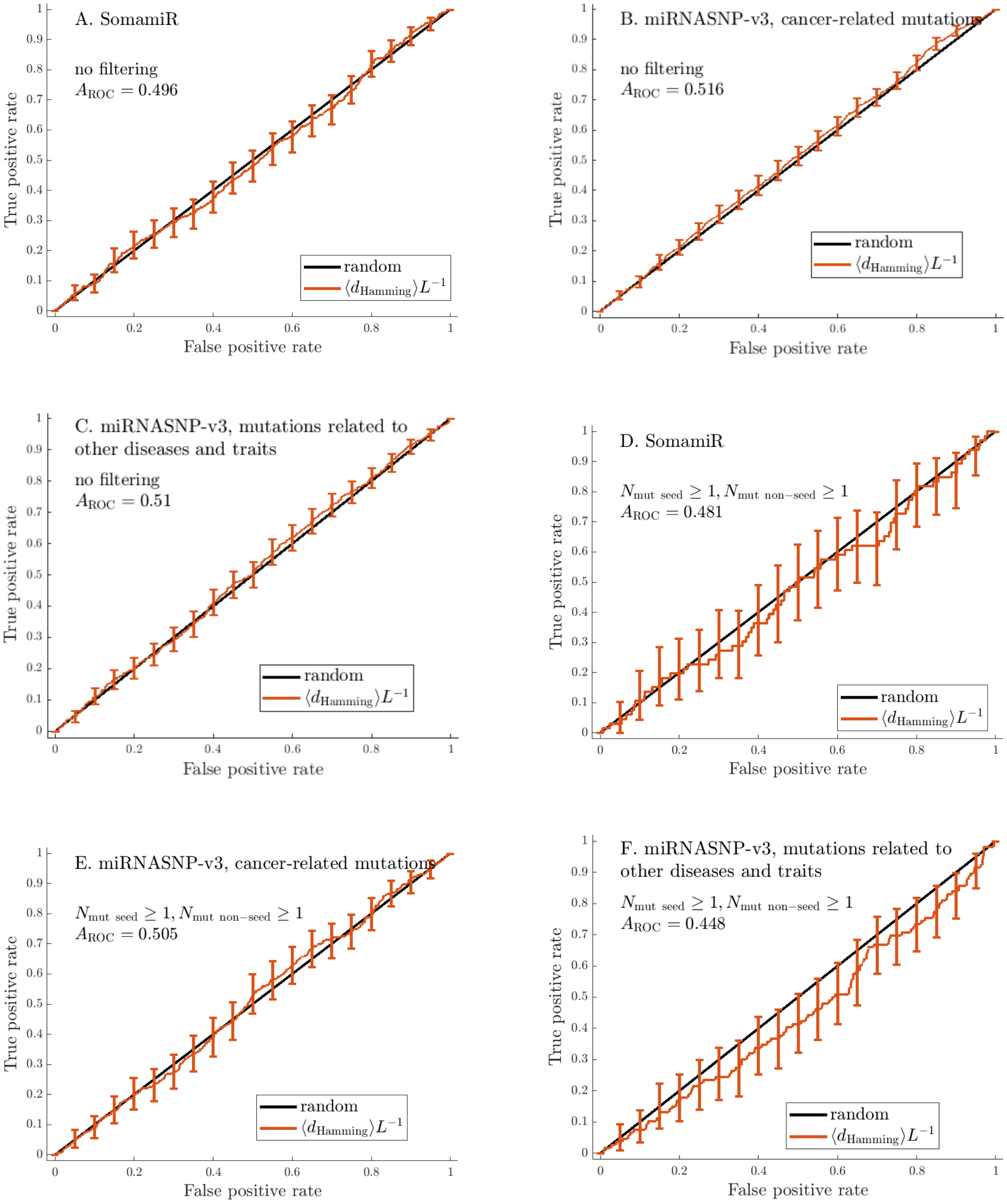
A. SomamiR
no filtering
$A_{\mathrm{ROC}} = 0.496$
B. miRNASNP-v3, cancer-related mutations
no filtering
$A_{\mathrm{ROC}} = 0.516$
C. miRNASNP-v3, mutations related to other diseases and traits
no filtering
$A_{\mathrm{ROC}} = 0.51$
D. SomamiR
$N_{\mathrm{mut\ seed}} \geq 1, N_{\mathrm{mut\ non-seed}} \geq 1$
$A_{\mathrm{ROC}} = 0.481$
E. miRNASNP-v3, cancer-related mutations
$N_{\mathrm{mut\ seed}} \geq 1, N_{\mathrm{mut\ non-seed}} \geq 1$
$A_{\mathrm{ROC}} = 0.505$
F. miRNASNP-v3, mutations related to other diseases and traits
$N_{\mathrm{mut\ seed}} \geq 1, N_{\mathrm{mut\ non-seed}} \geq 1$
$A_{\mathrm{ROC}} = 0.448$
random
$\langle d_{\mathrm{Hamming}} \rangle L^{-1}$
True positive rate
False positive rate

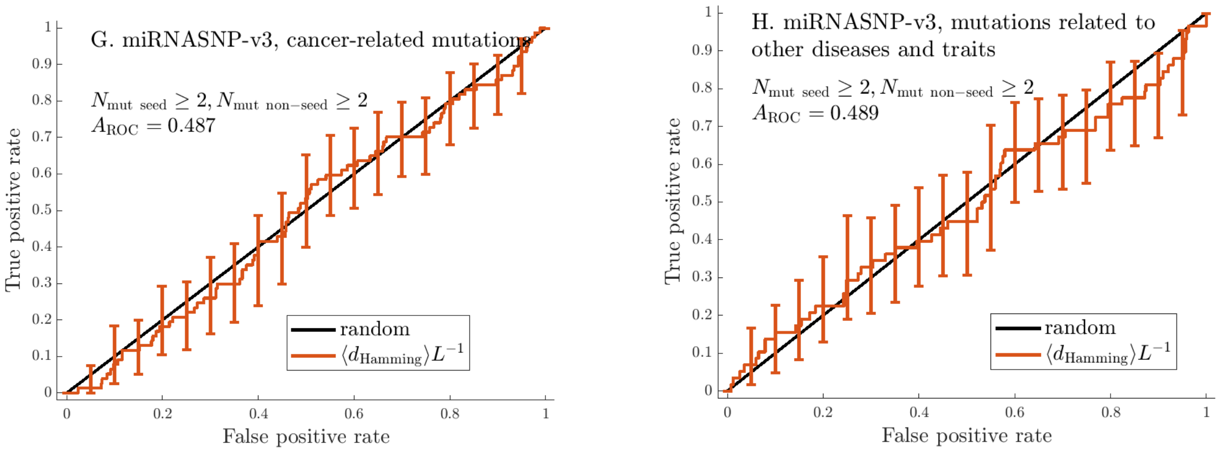

Figure S4: ROC curves built using $\langle d_{\text{Hamming}} \rangle L^{-1}$ as the criterion for predicting disease-related mutations. Data taken from SomamiR and miRNASNP-v3. The labels indicate the areas under the curves and the p-values for the criteria that perform significantly better than the random predictor (based on the two-sided Mann-Whitney test).

secondary structure or making it less stable; in the second one, it a mutation may interfere with miRNA function by causing sites essential to target binding to enter base pairs. Our metrics aim to quantify different types of secondary structure changes in order to detect any kind of association between modified miRNA secondary structure and disease.

2.3.3.2 Unfolded mutant and WT As miRNAs need to bind to mRNAs to perform their function, one may expect that the fully unfolded state with no base pairs is ideal for performing their functions. Under this hypothesis, mutations that make the unfolded state less stable would disrupt miRNA function. The ROC curves for this subset of the data are illustrated in Figure S13. As one can see in Tables S3-S10, it is much rarer for both the mutant and the WT to be unfolded and therefore, the sample size is much smaller than for the case with folded mutant and WT. This, combined with the many effects unrelated to miRNA SS, is probably the reason that the change in entropy does not perform significantly better than random for unfolded mutant and miRNA WTs.

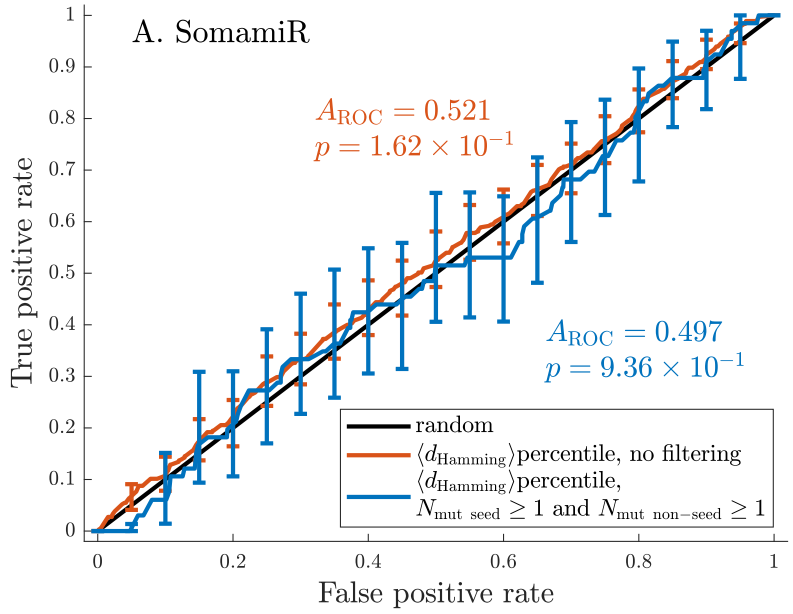
A. SomamiR
$A_{\mathrm{ROC}} = 0.521$
$p = 1.62 \times 10^{-1}$
$A_{\mathrm{ROC}} = 0.497$
$p = 9.36 \times 10^{-1}$
random
$\langle d_{\mathrm{Hamming}} \rangle$percentile, no filtering
$\langle d_{\mathrm{Hamming}} \rangle$percentile,
$N_{\mathrm{mut\ seed}} \geq 1$ and $N_{\mathrm{mut\ non-seed}} \geq 1$
True positive rate
False positive rate
0
0.1
0.2
0.3
0.4
0.5
0.6
0.7
0.8
0.9
1
0
0.2
0.4
0.6
0.8
1

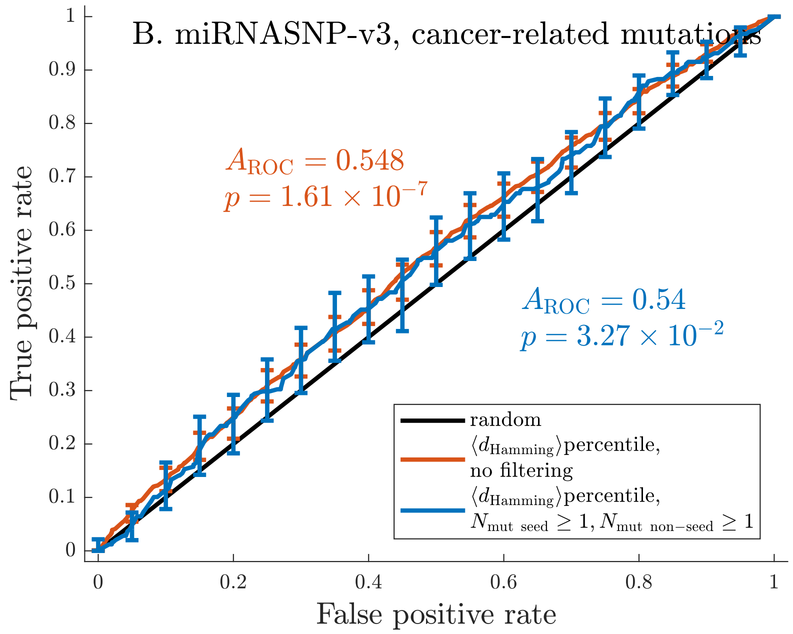
B. miRNASNP-v3, cancer-related mutations
$A_{\mathrm{ROC}} = 0.548$
$p = 1.61 \times 10^{-7}$
$A_{\mathrm{ROC}} = 0.54$
$p = 3.27 \times 10^{-2}$
random
$\langle d_{\mathrm{Hamming}} \rangle$percentile,
no filtering
$\langle d_{\mathrm{Hamming}} \rangle$percentile,
$N_{\mathrm{mut\ seed}} \geq 1, N_{\mathrm{mut\ non-seed}} \geq 1$
True positive rate
False positive rate
0
0.1
0.2
0.3
0.4
0.5
0.6
0.7
0.8
0.9
1
0
0.2
0.4
0.6
0.8
1

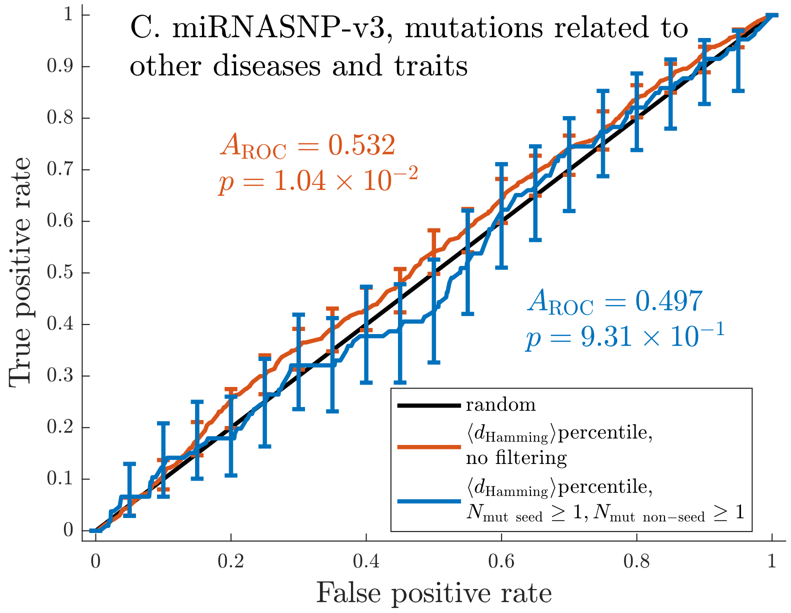

Figure S5: **The change in SS associated with a mutation as measured by the percentile of the normalized Hamming distance in SS between the WT and mutated ensembles is a predictor of the mutation's relationship with disease.** ROC curves built using $\langle d_{\mathrm{Hamming}} \rangle$%ile as the criterion for predicting disease-related mutations. Data from SomamiR (A), cancer-related mutations (B) and mutations related to other traits and diseases (C) from miRNASNP-v3, with either no filtering based on $N_{\mathrm{mut\ seed}}$ and $N_{\mathrm{mut\ non-seed}}$ (red curves) or with $N_{\mathrm{mut\ seed}} \geq 1$ and $N_{\mathrm{mut\ non-seed}} \geq 1$ (blue curves) for all mature miRNAs considered here. Error bars indicate pointwise 95% confidence bounds calculated at 21 equally spaced points with the bootstrapping method [3]. The area under the ROC curve (A_{ROC}) and the Mann-Whitney p-value [4] for the curves are also indicated. The $\Delta p_{\mathrm{unfolded}}$ criterion performs significantly better than the random one for both datasets, indicating that the probability that disease-related mutants are fully unfolded tends to be higher than that for other mutants. This could be because mutants with a higher $p_{\mathrm{unfolded\ mutant}}$ have a higher activity than their respective WTs, and, in the case of cancer-associated mutations, they may be more effective at downregulating tumour suppressor genes.

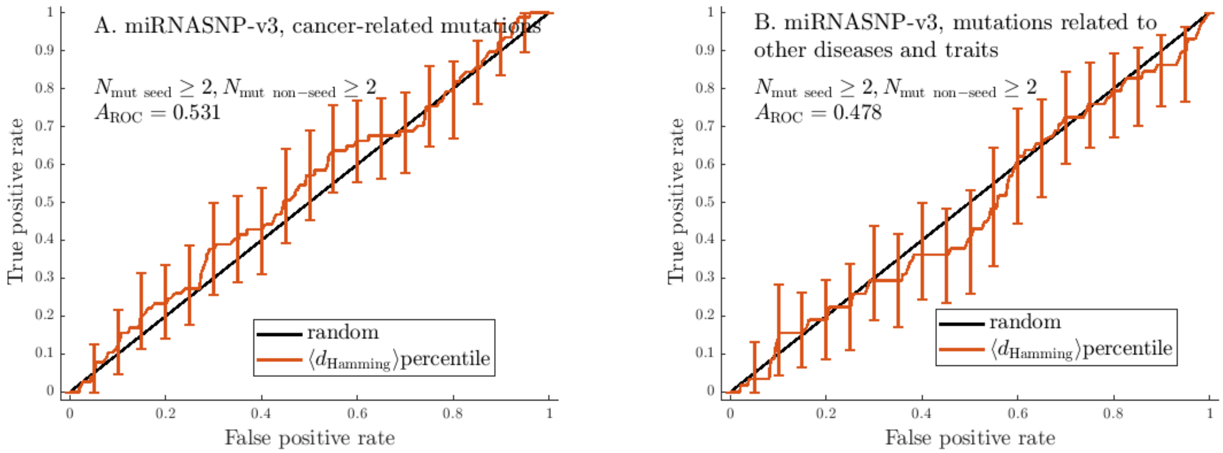

Figure S6: ROC curves built using $\langle d_{\text{Hamming}} \rangle$%ile as the criterion for predicting disease-related mutations. Data taken from miRNASNP-v3.

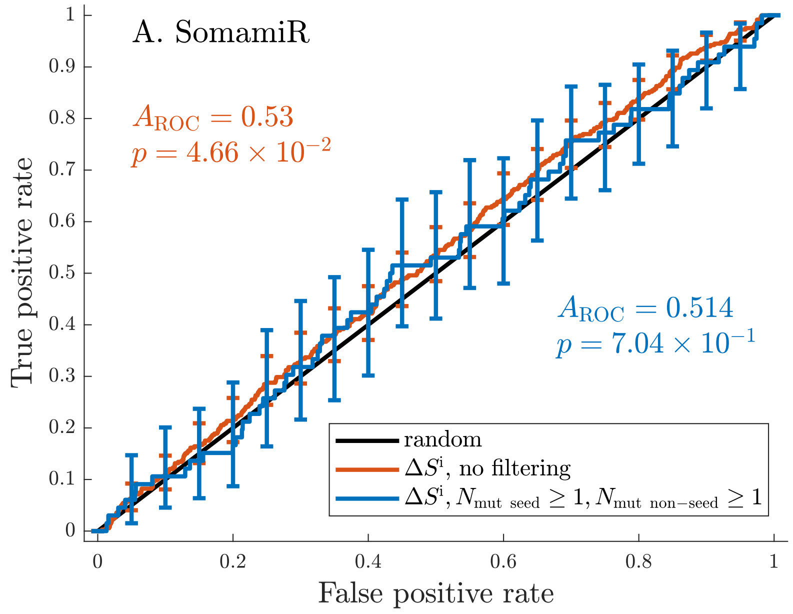
A. SomamiR
$A_{\mathrm{ROC}} = 0.53$
$p = 4.66 \times 10^{-2}$
$A_{\mathrm{ROC}} = 0.514$
$p = 7.04 \times 10^{-1}$
random
ΔS^{i}, no filtering
$\Delta S^{\mathrm{i}}, N_{\mathrm{mut\ seed}} \geq 1, N_{\mathrm{mut\ non-seed}} \geq 1$
True positive rate
False positive rate

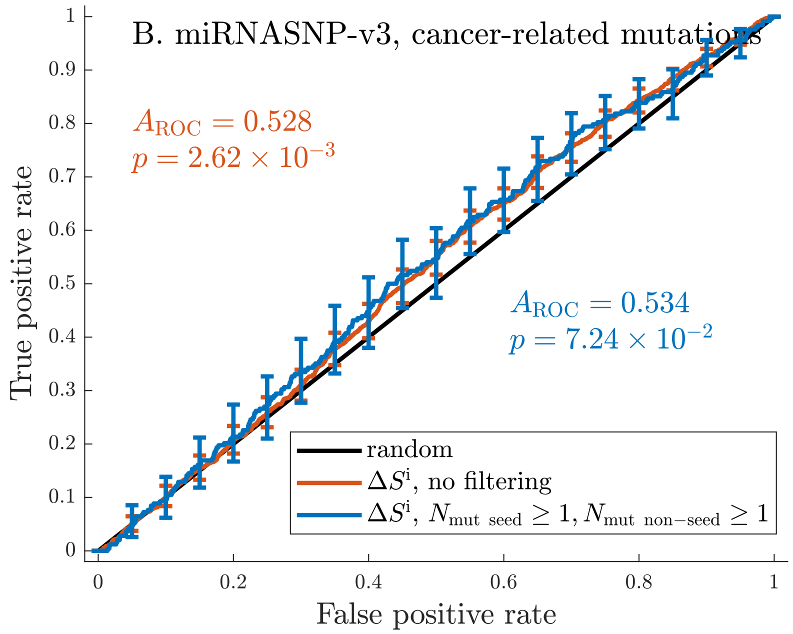
B. miRNASNP-v3, cancer-related mutations
$A_{\mathrm{ROC}} = 0.528$
$p = 2.62 \times 10^{-3}$
$A_{\mathrm{ROC}} = 0.534$
$p = 7.24 \times 10^{-2}$
random
ΔS^{i}, no filtering
$\Delta S^{\mathrm{i}}, N_{\mathrm{mut\ seed}} \geq 1, N_{\mathrm{mut\ non-seed}} \geq 1$
True positive rate
False positive rate

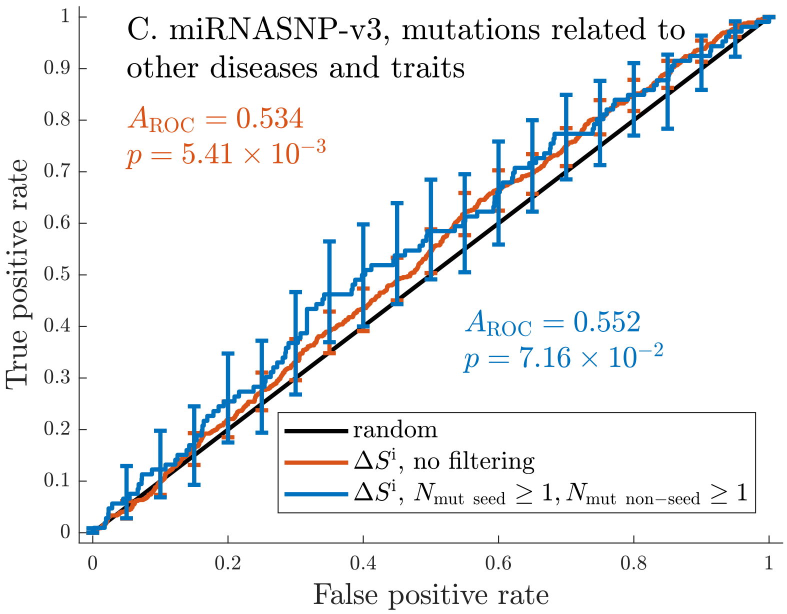

Figure S7: **The change in the positional entropy at the mutated site in a point mutant is a predictor of the mutation's relationship with disease.** ROC curves built using $\Delta S^{\mathrm{i}} = S^{\mathrm{i}}_{\mathrm{mut}} - S^{\mathrm{i}}_{\mathrm{WT}}$ as the criterion for predicting disease-related mutations. Data from SomamiR (A), cancer-related mutations (B) and mutations related to other traits and diseases (C) from miRNASNP-v3, with either no filtering based on $N_{\mathrm{mut\ seed}}$ and $N_{\mathrm{mut\ non-seed}}$ (red curves) or with $N_{\mathrm{mut\ seed}} \geq 1$ and $N_{\mathrm{mut\ non-seed}} \geq 1$ (blue curves) for all mature miRNAs considered here. Error bars indicate pointwise 95% confidence bounds calculated at 21 equally spaced points with the bootstrapping method [3]. The area under the ROC curve (A_{ROC}) and the Mann-Whitney p-value [4] for the curves are also indicated. The ΔS^{i} criterion performs significantly better than the random one for both datasets, when no filtering by $N_{\mathrm{mut\ seed}}$ and $N_{\mathrm{mut\ non-seed}}$ is applied. This indicates that the change in positional entropy at the mutated site tends to be lower for disease-related mutants.

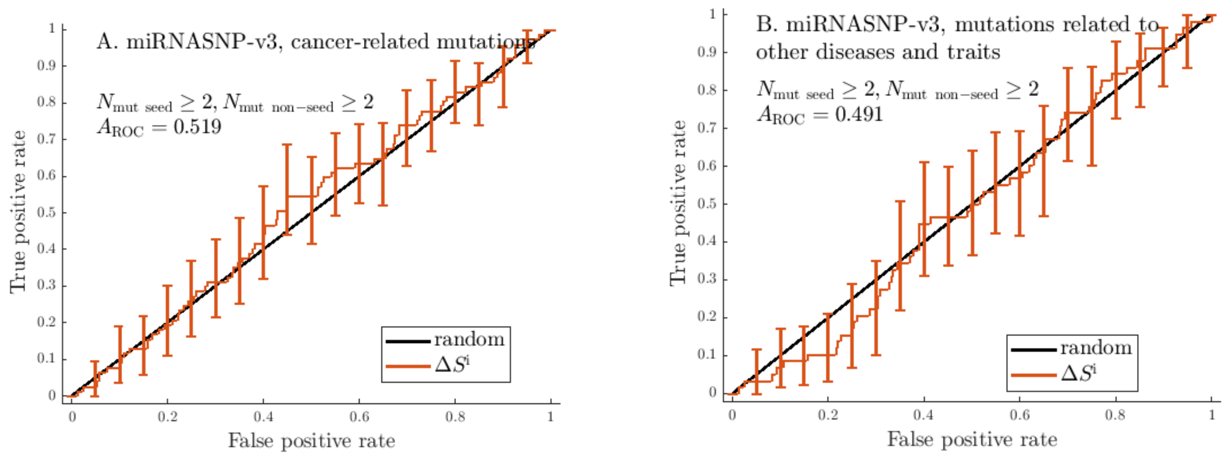

Figure S8: ROC curves built using ΔS^{i} as the criterion for predicting disease-related mutations for data from SomamiR and miRNASNP-v3. The labels indicate the areas under the curves and the p-values for the criteria that perform significantly better than the random predictor (based on the two-sided Mann-Whitney test).

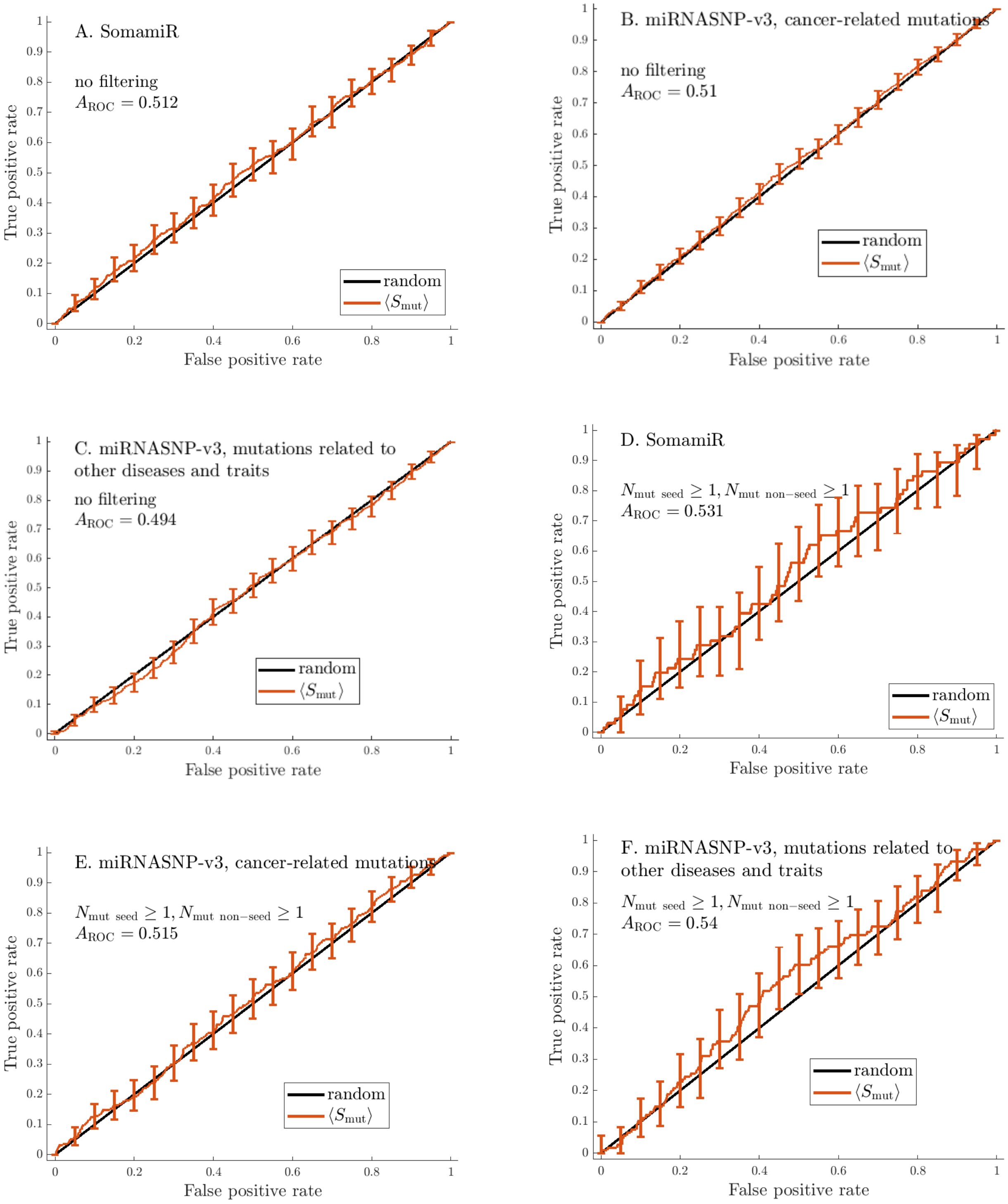

A. SomamiR
no filtering
$A_{\mathrm{ROC}} = 0.512$
B. miRNASNP-v3, cancer-related mutations
no filtering
$A_{\mathrm{ROC}} = 0.51$
C. miRNASNP-v3, mutations related to other diseases and traits
no filtering
$A_{\mathrm{ROC}} = 0.494$
D. SomamiR
$N_{\mathrm{mut\ seed}} \geq 1, N_{\mathrm{mut\ non-seed}} \geq 1$
$A_{\mathrm{ROC}} = 0.531$
E. miRNASNP-v3, cancer-related mutations
$N_{\mathrm{mut\ seed}} \geq 1, N_{\mathrm{mut\ non-seed}} \geq 1$
$A_{\mathrm{ROC}} = 0.515$
F. miRNASNP-v3, mutations related to other diseases and traits
$N_{\mathrm{mut\ seed}} \geq 1, N_{\mathrm{mut\ non-seed}} \geq 1$
$A_{\mathrm{ROC}} = 0.54$
random
$\langle S_{\mathrm{mut}} \rangle$
True positive rate
False positive rate
0
0.1
0.2
0.3
0.4
0.5
0.6
0.7
0.8
0.9
1

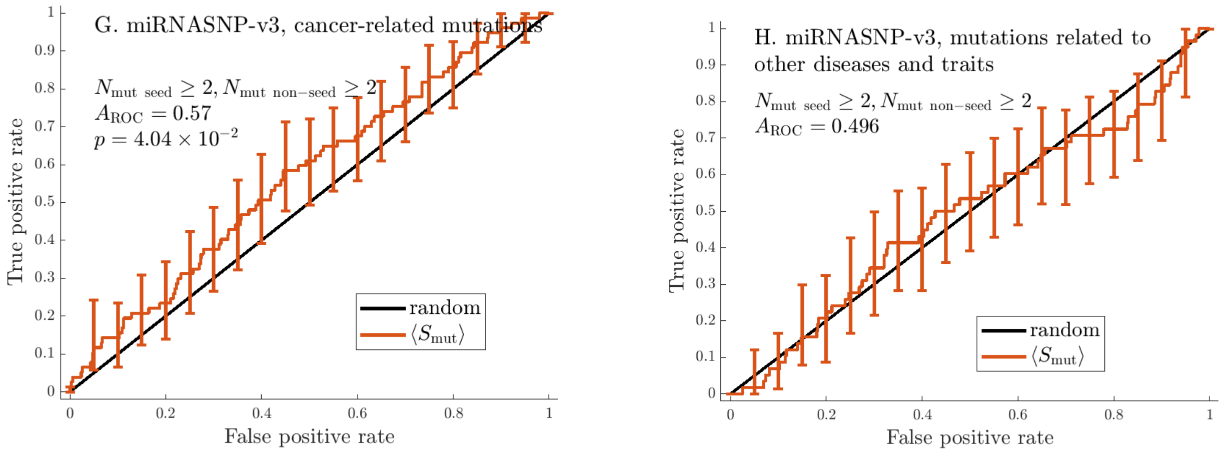

Figure S9: ROC curves built using $\langle S_{\text{mut}} \rangle$ as the criterion for predicting disease-related mutations for data from SomamiR and miRNASNP-v3. The labels indicate the areas under the curves and the p-values for the criteria that perform significantly better than the random predictor (based on the two-sided Mann-Whitney test).

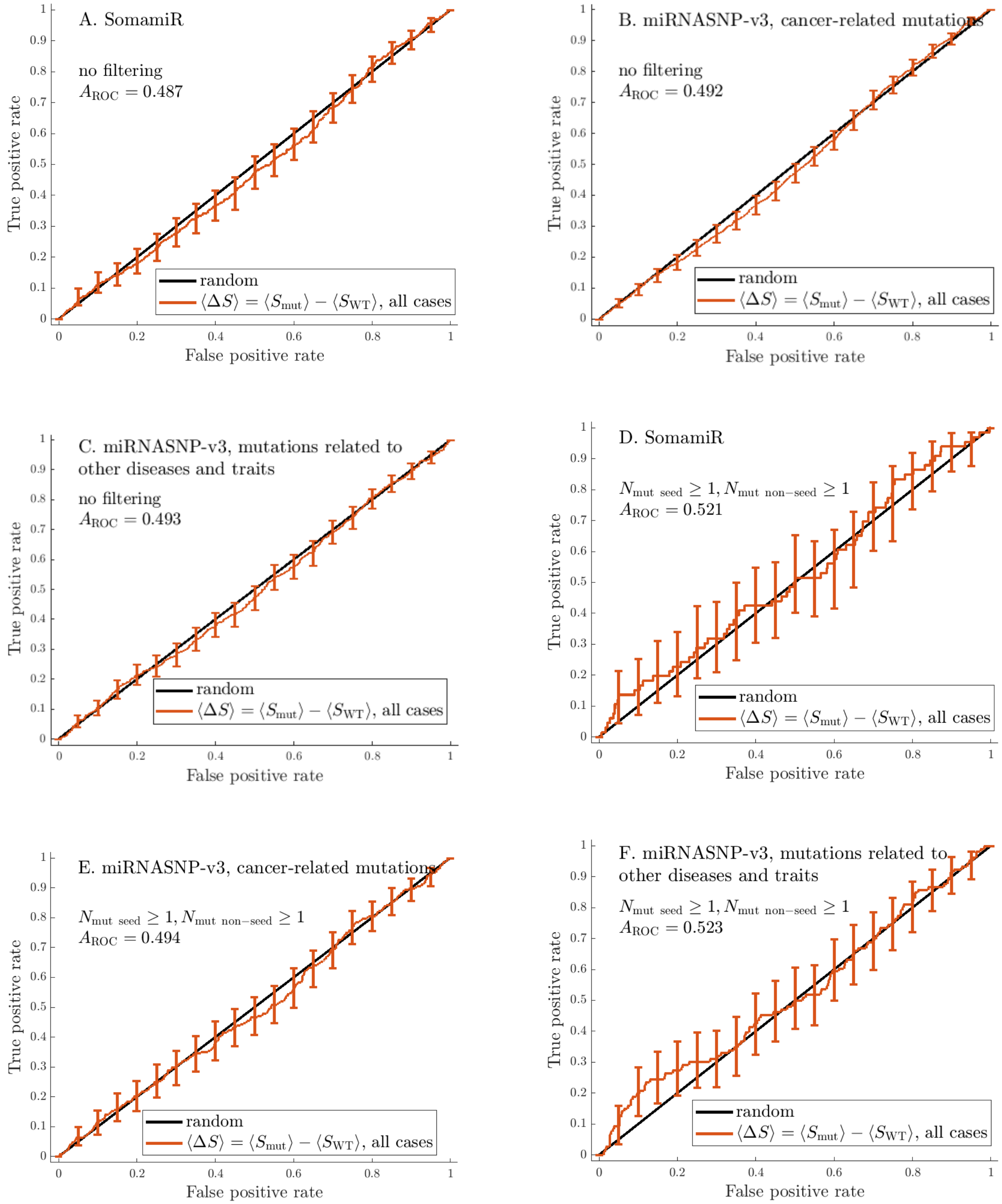
A. SomamiR
no filtering
$A_{\mathrm{ROC}} = 0.487$
B. miRNASNP-v3, cancer-related mutations
no filtering
$A_{\mathrm{ROC}} = 0.492$
C. miRNASNP-v3, mutations related to other diseases and traits
no filtering
$A_{\mathrm{ROC}} = 0.493$
D. SomamiR
$N_{\mathrm{mut\ seed}} \geq 1, N_{\mathrm{mut\ non-seed}} \geq 1$
$A_{\mathrm{ROC}} = 0.521$
E. miRNASNP-v3, cancer-related mutations
$N_{\mathrm{mut\ seed}} \geq 1, N_{\mathrm{mut\ non-seed}} \geq 1$
$A_{\mathrm{ROC}} = 0.494$
F. miRNASNP-v3, mutations related to other diseases and traits
$N_{\mathrm{mut\ seed}} \geq 1, N_{\mathrm{mut\ non-seed}} \geq 1$
$A_{\mathrm{ROC}} = 0.523$
random
$\langle \Delta S \rangle = \langle S_{\mathrm{mut}} \rangle - \langle S_{\mathrm{WT}} \rangle$, all cases
True positive rate
False positive rate

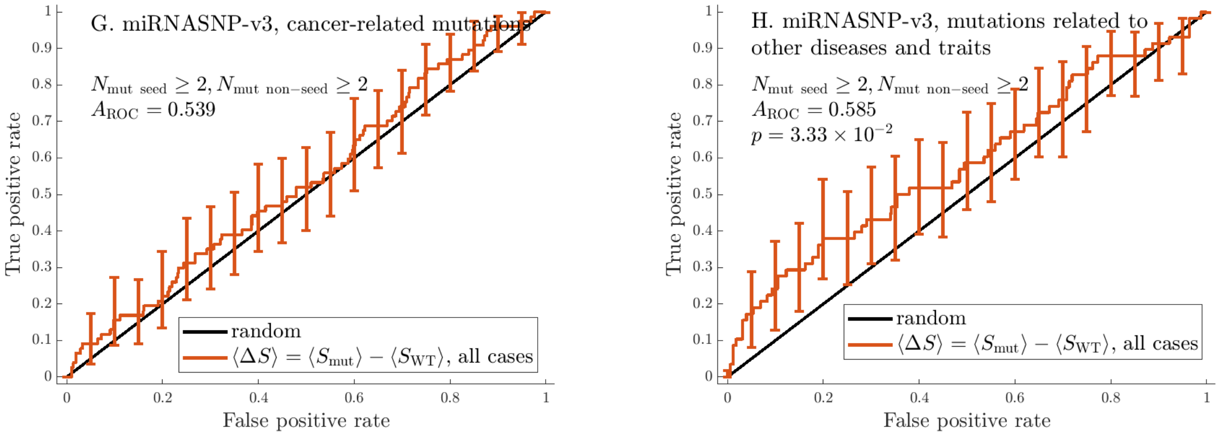

Figure S10: ROC curves built using $\langle \Delta S \rangle = \langle S_{\mathrm{mut}} \rangle - \langle S_{\mathrm{WT}} \rangle$ as the criterion for predicting disease-related mutations applied to data from SomamiR and miRNASNP-v3. The labels indicate the areas under the curves and the p-values for the criteria that perform significantly better than the random predictor (based on the two-sided Mann-Whitney test).

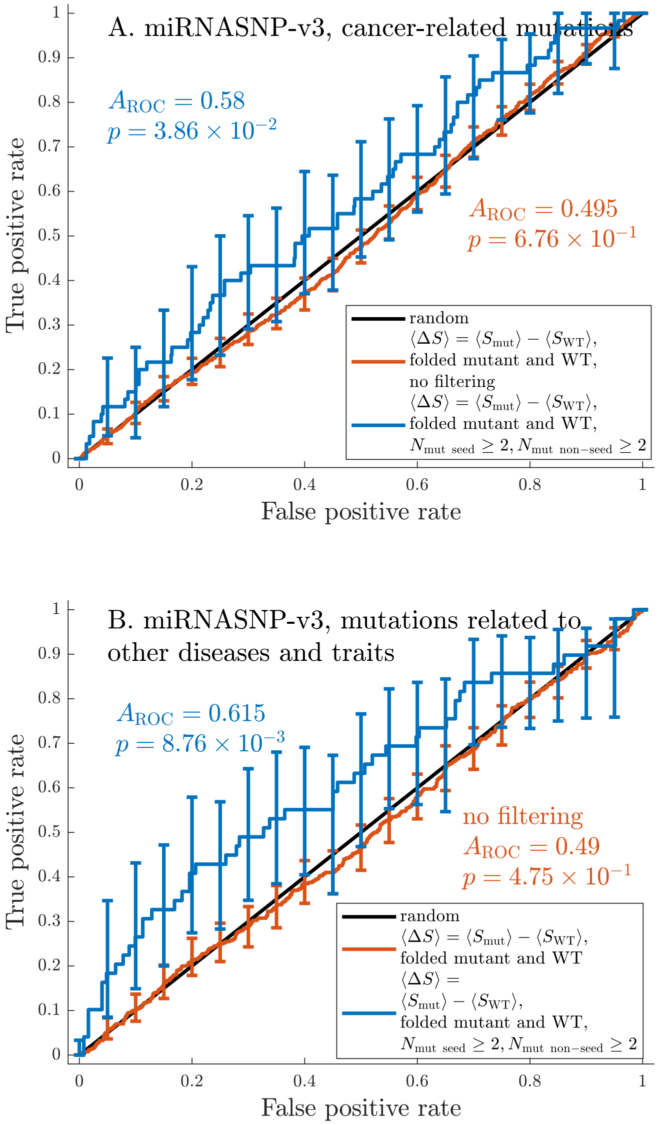

Figure S11: **The change in the average positional entropy, which measures the stability of a fold, of a miRNA associated with a mutation is a predictor of the mutation's relationship with disease for a subset of miRNAs with a comparatively large number of recorded mutations.** ROC curves built characterizing the difference in average positional entropy between mutant and WT that are both folded, $\langle \Delta S \rangle = \langle S_{\text{mut}} \rangle - \langle S_{\text{WT}} \rangle$. When applied to the unfiltered datasets on mutations related to cancer and other diseases from miRNASNP-v3, this criterion does not perform significantly better than the random one. However, when looking only at miRNAs for which the database records at least two mutations in both the seed and the rest of the mature region, the criterion performs significantly better than random, particularly for mutations unrelated to cancer. The results for cancer-related mutations highlight a cluster of 23 miRNAs for which the change in the stability of the fold plays a significant role, and the mutations related to other traits and diseases highlight another cluster of 9 miRNAs (see Tables S9-S10 in the SI for more information on the samples). The top-ranked mutations are those with the highest $\langle \Delta S \rangle$, which suggests that for miRNAs within these clusters, less stable folds tend to be associated with disease. We explore the data for one of the miRNAs from the non-cancer cluster, hsa-miR-4537, in Figure S17, and find strong association of additional criteria with disease.

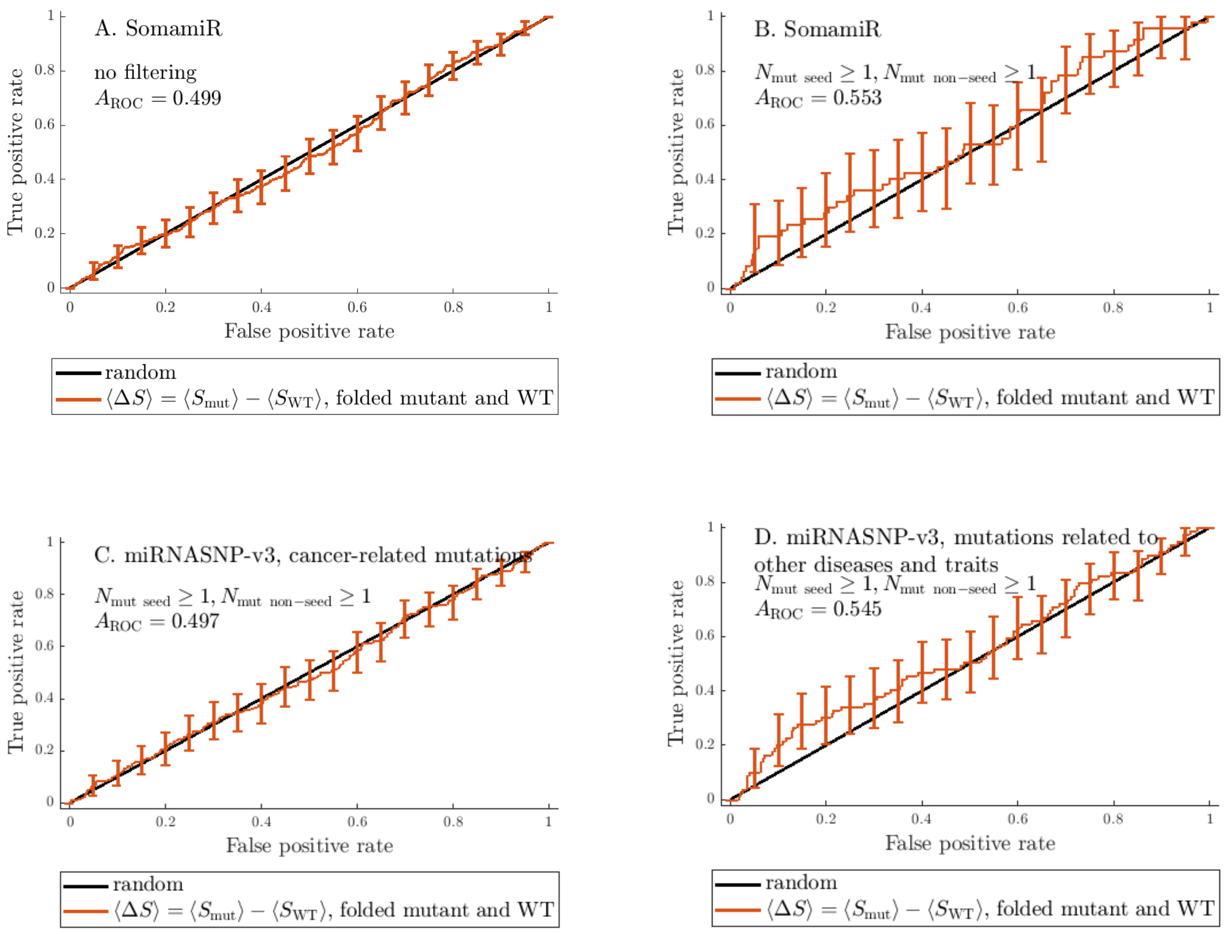

Figure S12: ROC curves built using $\langle \Delta S \rangle = \langle S_{\mathrm{mut}} \rangle - \langle S_{\mathrm{WT}} \rangle$ as the criterion for predicting disease-related mutations; the plot is based on the cases for which both the mutant and the WT MFE structures are folded. Data taken from SomamiR and miRNASNP-v3.

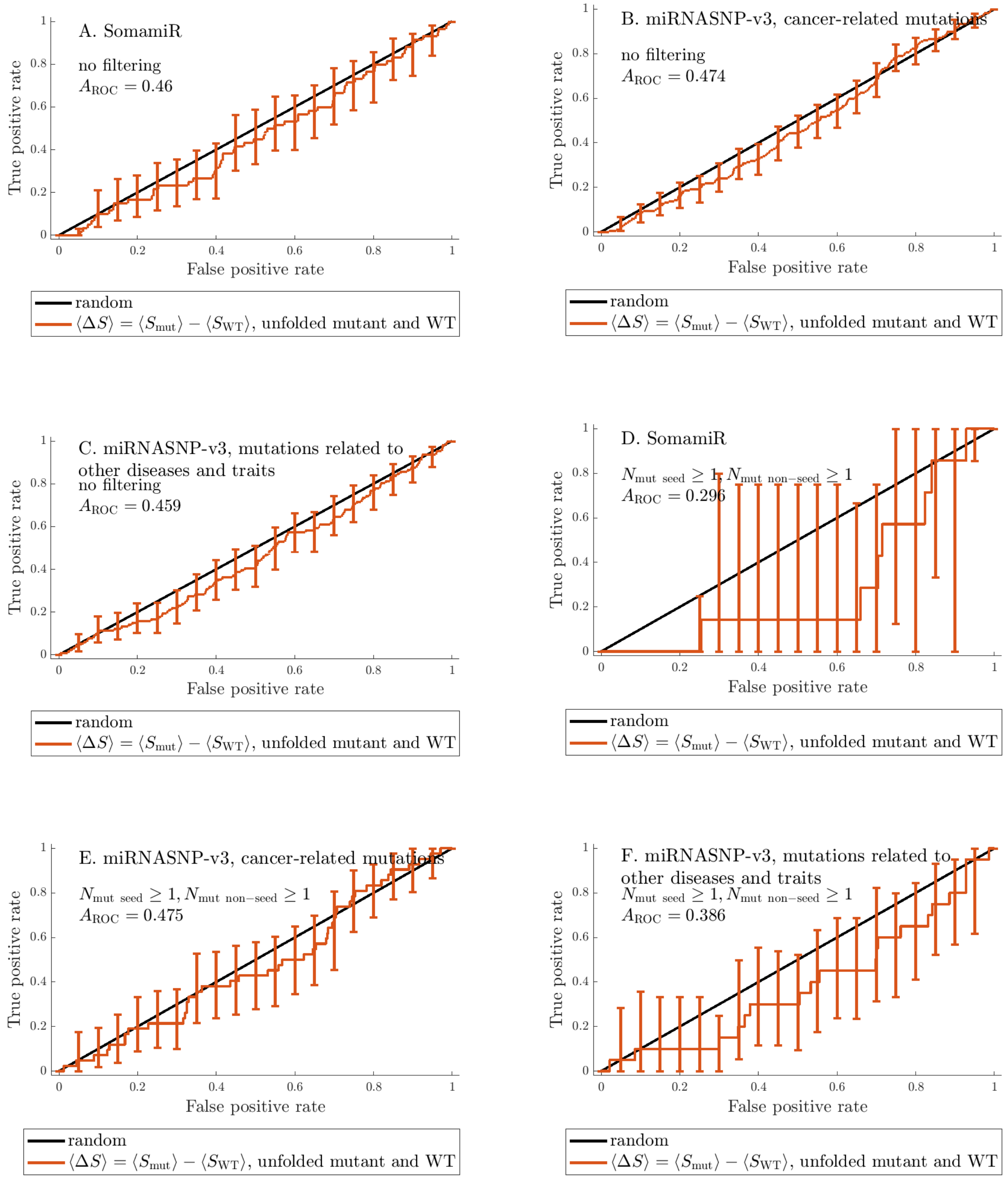
A. SomamiR
no filtering
$A_{\text{ROC}} = 0.46$
B. miRNASNP-v3, cancer-related mutations
no filtering
$A_{\text{ROC}} = 0.474$
C. miRNASNP-v3, mutations related to other diseases and traits
no filtering
$A_{\text{ROC}} = 0.459$
D. SomamiR
$N_{\text{mut seed}} \geq 1, N_{\text{mut non-seed}} \geq 1$
$A_{\text{ROC}} = 0.296$
E. miRNASNP-v3, cancer-related mutations
$N_{\text{mut seed}} \geq 1, N_{\text{mut non-seed}} \geq 1$
$A_{\text{ROC}} = 0.475$
F. miRNASNP-v3, mutations related to other diseases and traits
$N_{\text{mut seed}} \geq 1, N_{\text{mut non-seed}} \geq 1$
$A_{\text{ROC}} = 0.386$
True positive rate
False positive rate
0
0.2
0.4
0.6
0.8
1
random
$\langle \Delta S \rangle = \langle S_{\text{mut}} \rangle - \langle S_{\text{WT}} \rangle$, unfolded mutant and WT

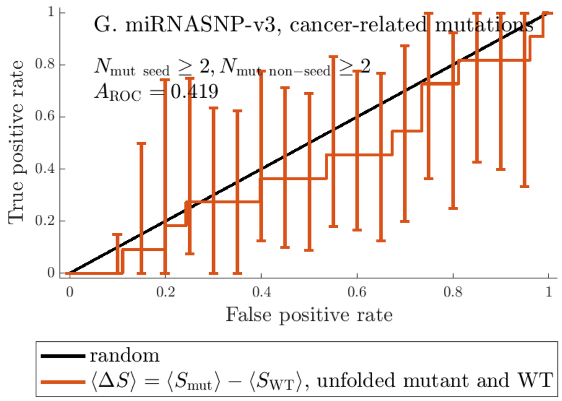

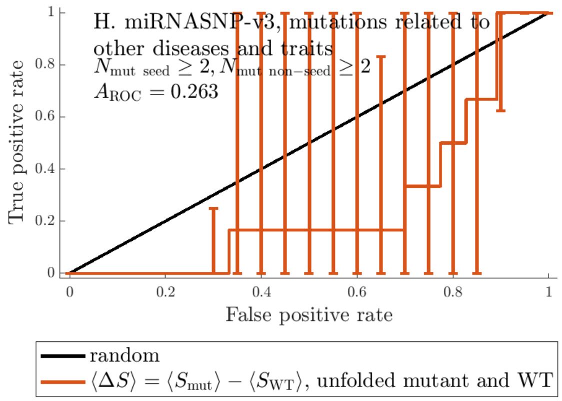

Figure S13: ROC curves built using $\langle \Delta S \rangle = \langle S_{\mathrm{mut}} \rangle - \langle S_{\mathrm{WT}} \rangle$ as the criterion for predicting disease-related mutations; the plot is based on the cases for which both the mutant and the WT MFE structures are fully unfolded. Data taken from SomamiR and miRNASNP-v3.

	all		miRNAs with genes from MiRGeneDB	
criterion	A_{ROC}	p-value	A_{ROC}	p-value
$\Delta p_{\mathrm{unfolded}}$	0.544	4.92×10^{-9}	0.561	1.10×10^{-6}
$\langle d_{\mathrm{Hamming}} \rangle$%ile	0.543	9.15×10^{-9}	0.548	1.24×10^{-4}
$\Delta S^{(\mathrm{i})}$	0.532	2.50×10^{-5}	0.531	1.15×10^{-2}
$d_{\mathrm{Boltzmann}}$	0.529	8.80×10^{-5}	0.534	6.10×10^{-3}

Table S1: The $\Delta p_{\mathrm{unfolded}}$, $\langle d_{\mathrm{Hamming}} \rangle$%ile, $\Delta S^{(\mathrm{i})}$ and $d_{\mathrm{Boltzmann}}$ criteria indicate statistically significant association of secondary structure changes with disease when applied to data from miRNASNP without filtering by disease type, regardless of whether the genes encoding the miRNAs in question are represented in MiRGeneDB 2.1. As elsewhere, mutations are ranked by these criteria, and the receiver-operator characteristic A_{ROC} is used to establish whether they predict disease-associated mutations in the miRNASNP-v3 dataset. The A_{ROC} values for $\Delta p_{\mathrm{unfolded}}$ exceed 0.5 significantly for all three metrics in both subsets of the data, suggesting that at least for some miRNAs secondary structure changes can be indicative of disease association. When checking for significance, we calculate the Mann-Whitney p-value and set the threshold to 0.05.

	miRNAs with genes from MiRGeneDB	
criterion	A_{ROC}	p-value
$\Delta p_{\mathrm{unfolded}}$	0.548	3.64×10^{-2}
$\langle d_{\mathrm{Hamming}} \rangle$%ile	*0.533*	*1.53×10^{-1}*
$\Delta S^{(\mathrm{i})}$	*0.544*	*5.46×10^{-2}*
$d_{\mathrm{Boltzmann}}$	*0.513*	*5.77×10^{-1}*

Table S2: The $\Delta p_{\mathrm{unfolded}}$ criterion is the only one that indicates statistically significant association of secondary structure changes with disease when applied to data from SomamiR pertaining to miRNAs encoded by genes represented in MiRGeneDB 2.1. As elsewhere, mutations are ranked by these criteria, and the receiver-operator characteristic A_{ROC} is used to establish whether they predict disease-associated mutations. When checking for significance, we calculate the Mann-Whitney p-value and set the threshold to 0.05; results with p-values above this threshold in *italics*.

3 Tables and graphical comparisons of measures of criteria performance

A. Performance of various criteria when applied to SomamiR data

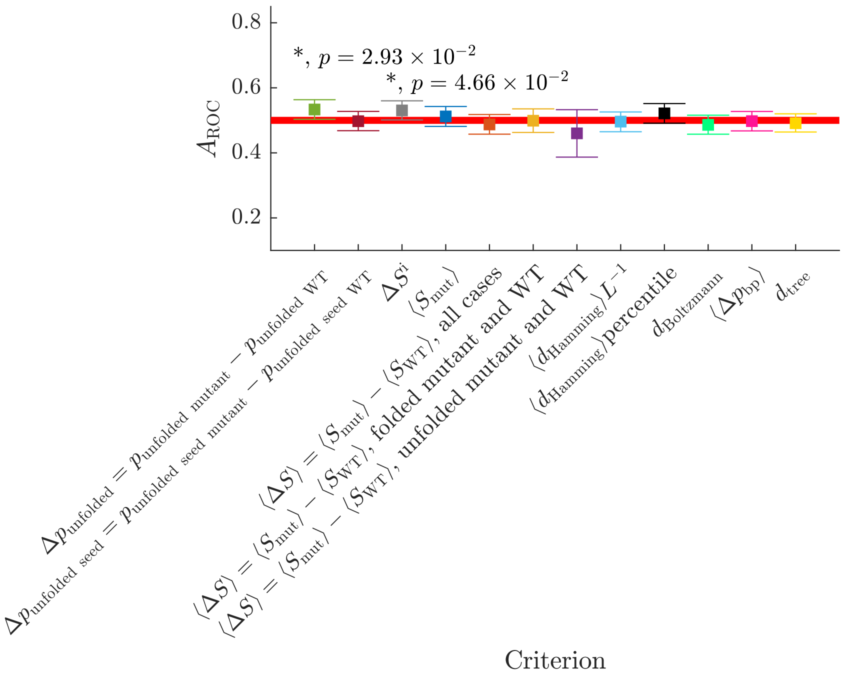

B. Performance of various criteria when applied to miRNASNP-v3 data, cancer-related mutations

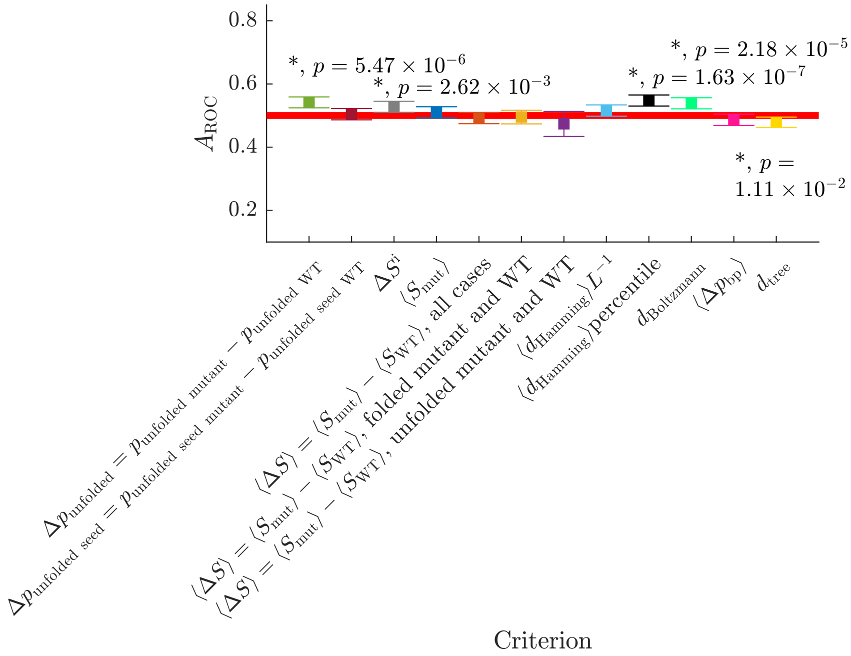

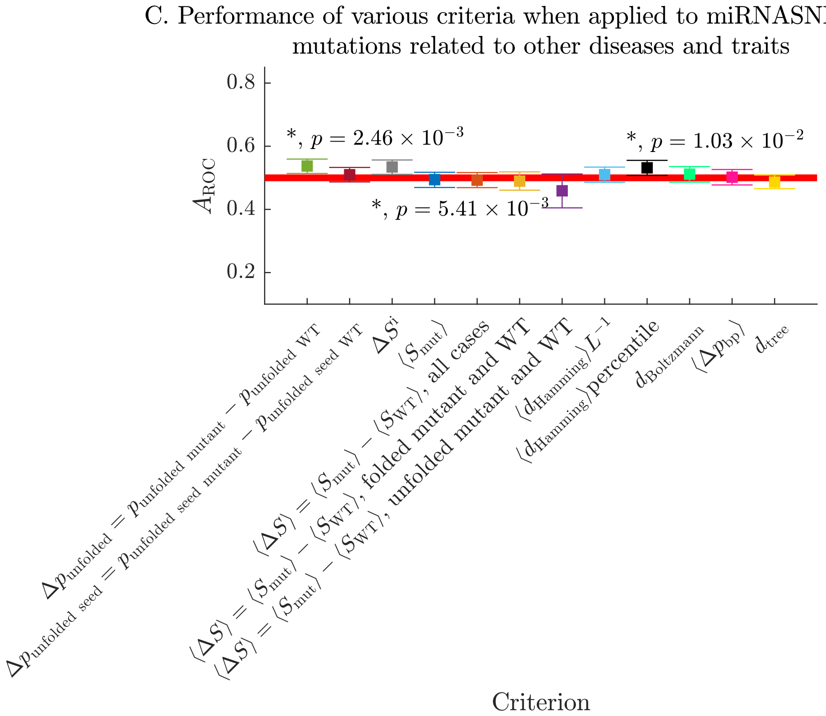

Figure S14: **Two independent SS-based criteria consistently predict association of miRNA mutations with disease better than the random criterion.** Comparison of the performance of various criteria in terms of predicting disease-associated mutations when applied to data from SomamiR (A), data on cancer-related mutations (B) and mutations related to other traits and diseases (C) from miRNASNP-v3 with no filtering based on $N_{\text{mut seed}}$ and $N_{\text{mut non-seed}}$. Squares mark A_{ROC} values, error bars show 95% confidence intervals, and Mann-Whitney p-values are indicated wherever $p < 0.05$. The red horizontal lines indicate the area under the curve for the random criterion, $A_{\text{ROC}} = 0.5$. The criterion that performs significantly better than random for all datasets is $\Delta p_{\text{unfolded}}$, which measures the change in probability that the miRNA is fully unfolded. For miRNASNP-v3 data, A_{ROC} is also significantly greater 0.5 if mutants are instead ranked in ascending order of their percentile in the $\langle d_{\text{Hamming}} \rangle$ distribution. This means that, within the two miRNASNP-v3 datasets, mutations which change miRNA secondary structure less tend to be associated with disease.

A. Performance of various criteria when applied to SomamiR data

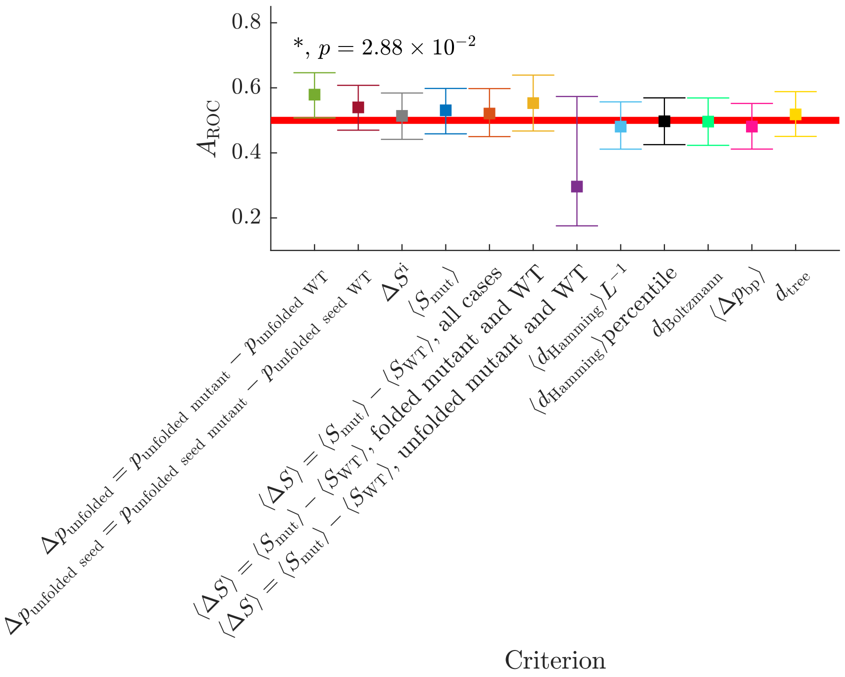

B. Performance of various criteria when applied to miRNASNP-v3 data, cancer-related mutations

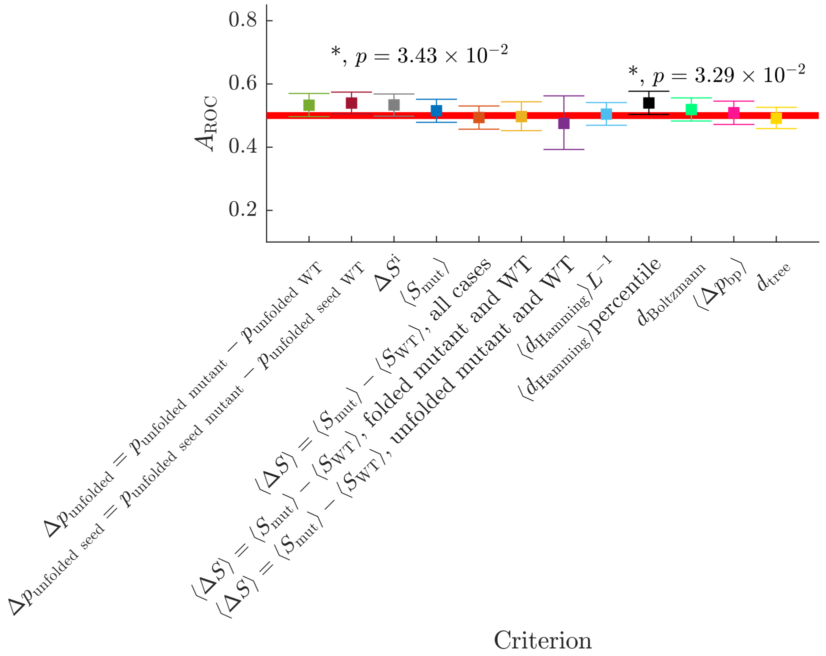

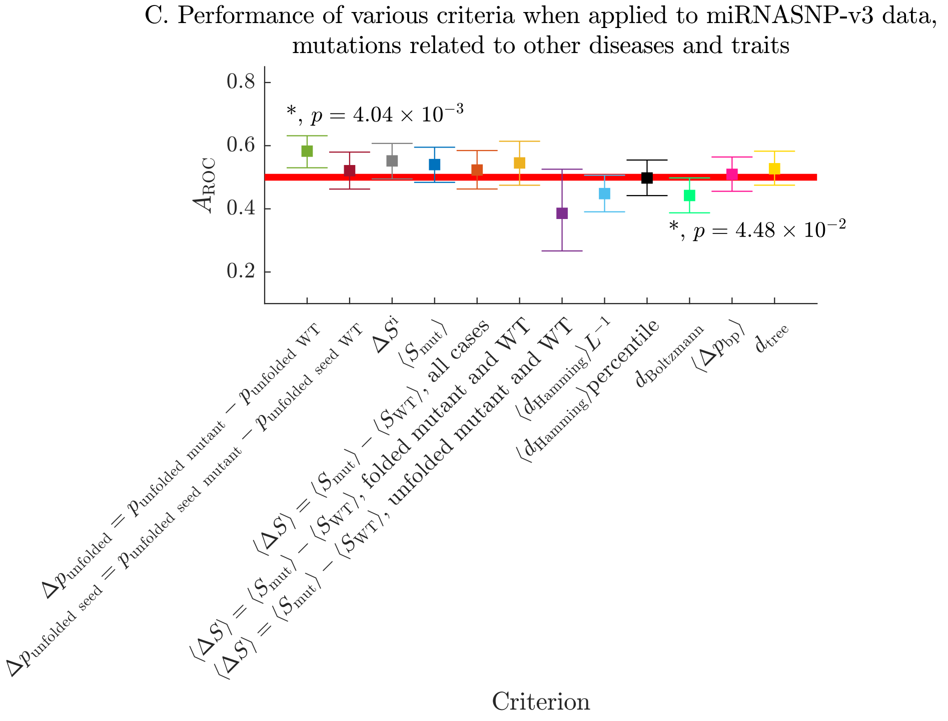

Figure S15: Comparison of the performance of various criteria in terms of predicting disease-associated mutations when applied to data from SomamiR and miRNASNP-v3 with $N_{\mathrm{mut\ seed}} \geq 1$ and $N_{\mathrm{mut\ non-seed}} \geq 1$. Squares mark A_{ROC} values, error bars show 95% confidence intervals, and p-values are indicated wherever $p < 0.05$. The red horizontal line indicates the area under the curve for the random criterion, $A_{\mathrm{ROC}} = 0.5$.

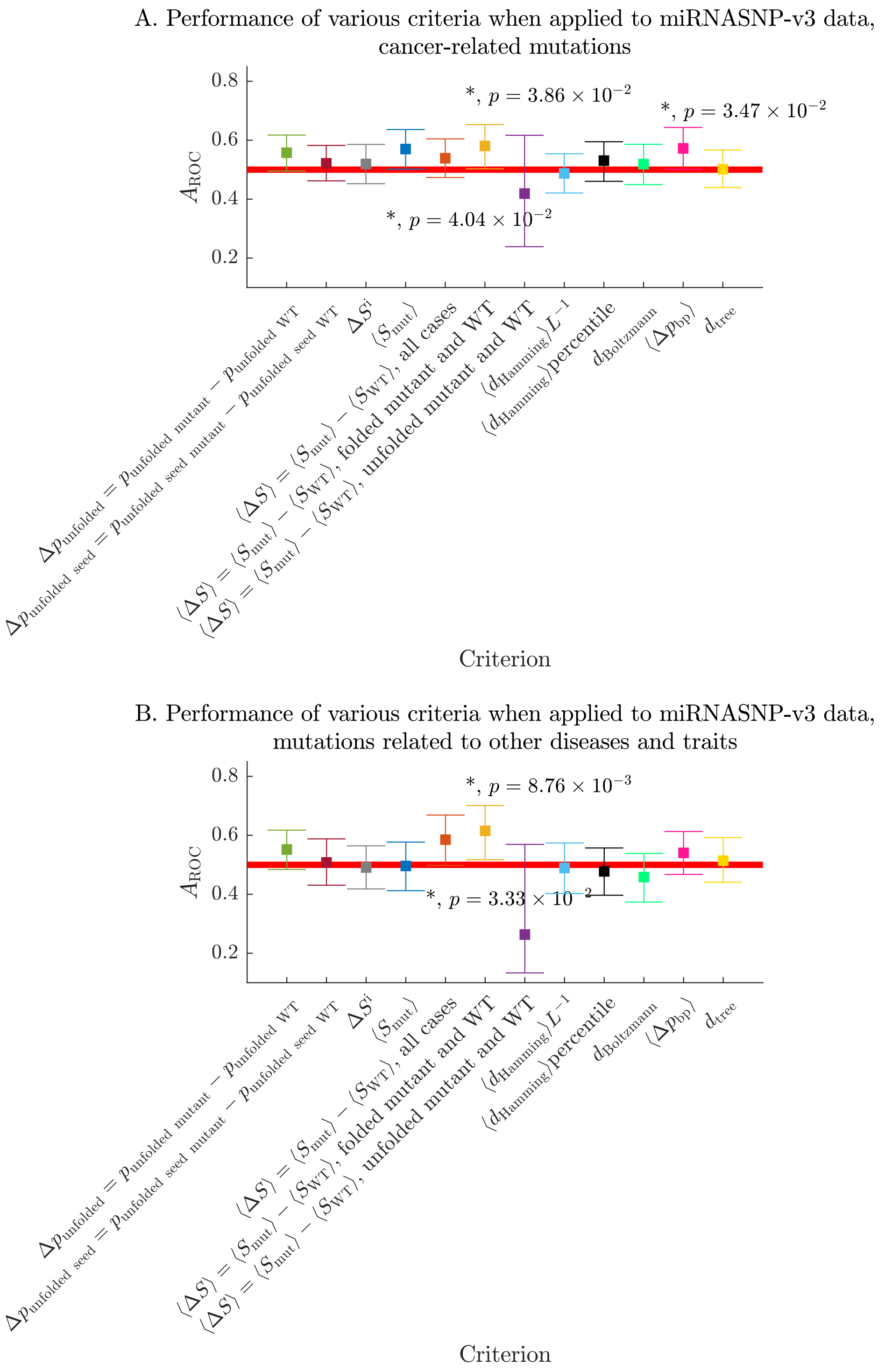

Figure S16: Comparison of the performance of various criteria in terms of predicting disease-associated mutations when applied to data from miRNASNP-v3 with $N_{\mathrm{mut\ seed}} \geq 2$ and $N_{\mathrm{mut\ non-seed}} \geq 2$. Squares mark A_{ROC} values, error bars show 95% confidence intervals, and p-values are indicated wherever $p < 0.05$. The red horizontal line indicates the area under the curve for the random criterion, $A_{\mathrm{ROC}} = 0.5$.

Table S3: Areas under the ROC curves (A_{ROC}) for various criteria applied to the miRNA entries from SomamiR with no filtering based on $N_{\text{mut seed}}$ and $N_{\text{mut non-seed}}$, and p-values based on the two-sided Mann-Whitney test. p-values of less than 0.05, which indicate criteria that perform significantly better than the random predictor (with $A_{\text{ROC}} = 0.5$), in **bold**.

Criterion	A_{ROC}	p-value	Number of unique mature miRNAs in the sample
$\Delta p_{\text{unfolded}}$	0.533	$\mathbf{2.93 \times 10^{-2}}$	285
$\Delta p_{\text{unfolded seed}}$	0.497	8.68×10^{-1}	285
$\Delta S^{(\text{i})}$	0.530	$\mathbf{4.66 \times 10^{-2}}$	285
$\langle S_{\text{mut}} \rangle$	0.512	4.32×10^{-1}	285
$\langle \Delta S \rangle$, all cases	0.487	4.11×10^{-1}	285
$\langle \Delta S \rangle$, folded mutant and WT	0.499	9.51×10^{-1}	215
$\langle \Delta S \rangle$, unfolded mutant and WT	0.460	2.90×10^{-1}	70
$\langle d_{\text{Hamming}} \rangle L^{-1}$	0.496	8.00×10^{-1}	285
$\langle d_{\text{Hamming}} \rangle$ percentile	0.521	1.62×10^{-1}	285
$d_{\text{Boltzmann}}$	0.486	3.65×10^{-1}	285
$\langle \Delta p_{\text{bp}} \rangle$	0.498	8.76×10^{-1}	285
d_{tree}	0.491	5.35×10^{-1}	285

Table S4: Results for various criteria applied to the entries from miRNASNP-v3 related to cancer; no filtering by $N_{\text{mut seed}}$ and $N_{\text{mut non-seed}}$ is applied. The table contains areas under the ROC curves (A_{ROC}), and p-values based on the two-sided Mann-Whitney test. p-values of less than 0.05, which indicate criteria that perform significantly better than the random predictor (with $A_{\text{ROC}} = 0.5$), in **bold**.

Criterion	A_{ROC}	p-value	Number of unique mature miRNAs in the sample
$\Delta p_{\text{unfolded}}$	0.542	$\mathbf{5.47 \times 10^{-6}}$	675
$\Delta p_{\text{unfolded seed}}$	0.504	6.34×10^{-1}	675
$\Delta S^{(\text{i})}$	0.528	$\mathbf{2.62 \times 10^{-3}}$	675
$\langle S_{\text{mut}} \rangle$	0.510	2.57×10^{-1}	675
$\langle \Delta S \rangle$, all cases	0.492	3.90×10^{-1}	675
$\langle \Delta S \rangle$, folded mutant and WT	0.495	6.76×10^{-1}	512
$\langle \Delta S \rangle$, unfolded mutant and WT	0.474	2.24×10^{-1}	163
$\langle d_{\text{Hamming}} \rangle L^{-1}$	0.516	7.83×10^{-2}	675
$\langle d_{\text{Hamming}} \rangle$ percentile	0.548	$\mathbf{1.61 \times 10^{-7}}$	675
$d_{\text{Boltzmann}}$	0.539	$\mathbf{2.18 \times 10^{-5}}$	675
$\langle \Delta p_{\text{bp}} \rangle$	0.486	1.41×10^{-1}	675
d_{tree}	0.479	$\mathbf{1.11 \times 10^{-2}}$	675

Table S5: Results for various criteria applied to the entries from miRNASNP-v3 related to diseases other than cancer; no filtering by $N_{\mathrm{mut\ seed}}$ and $N_{\mathrm{mut\ non-seed}}$ is applied. The table contains areas under the ROC curves (A_{ROC}), and p-values based on the two-sided Mann-Whitney test. p-values of less than 0.05, which indicate criteria that perform significantly better than the random predictor (with $A_{\mathrm{ROC}} = 0.5$), in **bold**.

Criterion	A_{ROC}	p-value	Number of unique mature miRNAs in the sample
$\Delta p_{\mathrm{unfolded}}$	0.537	$\mathbf{2.46 \times 10^{-3}}$	400
$\Delta p_{\mathrm{unfolded\ seed}}$	0.510	4.28×10^{-1}	400
$\Delta S^{(\mathrm{i})}$	0.534	$\mathbf{5.41 \times 10^{-3}}$	400
$\langle S_{\mathrm{mut}} \rangle$	0.494	6.33×10^{-1}	400
$\langle \Delta S \rangle$, all cases	0.493	5.47×10^{-1}	400
$\langle \Delta S \rangle$, folded mutant and WT	0.490	4.75×10^{-1}	292
$\langle \Delta S \rangle$, unfolded mutant and WT	0.459	1.36×10^{-1}	108
$\langle d_{\mathrm{Hamming}} \rangle L^{-1}$	0.510	4.21×10^{-1}	400
$\langle d_{\mathrm{Hamming}} \rangle$ percentile	0.532	$\mathbf{1.04 \times 10^{-2}}$	400
$d_{\mathrm{Boltzmann}}$	0.512	3.41×10^{-1}	400
$\langle \Delta p_{\mathrm{bp}} \rangle$	0.502	8.69×10^{-1}	400
d_{tree}	0.487	2.44×10^{-1}	400

Table S6: Areas under the ROC curves (A_{ROC}) for various criteria applied to the miRNA entries from SomamiR for which $N_{\text{mut seed}} \geq 1$ and $N_{\text{mut non-seed}} \geq 1$, and p-values based on the two-sided Mann-Whitney test. p-values of less than 0.05, which indicate criteria that perform significantly better than the random predictor (with $A_{\mathrm{ROC}} = 0.5$), in **bold**.

Criterion	A_{ROC}	p-value	Number of unique mature miRNAs in the sample
$\Delta p_{\text{unfolded}}$	0.579	$\mathbf{2.88 \times 10^{-2}}$	40
$\Delta p_{\text{unfolded seed}}$	0.540	2.70×10^{-1}	40
$\Delta S^{(\mathrm{i})}$	0.514	7.04×10^{-1}	40
$\langle S_{\text{mut}} \rangle$	0.531	3.93×10^{-1}	40
$\langle \Delta S \rangle$, all cases	0.521	5.64×10^{-1}	40
$\langle \Delta S \rangle$, folded mutant and WT	0.553	2.16×10^{-1}	31
$\langle \Delta S \rangle$, unfolded mutant and WT	0.296	6.59×10^{-2}	9
$\langle d_{\text{Hamming}} \rangle L^{-1}$	0.481	5.96×10^{-1}	40
$\langle d_{\text{Hamming}} \rangle$ percentile	0.497	9.36×10^{-1}	40
$d_{\text{Boltzmann}}$	0.496	9.16×10^{-1}	40
$\langle \Delta p_{\text{bp}} \rangle$	0.481	5.96×10^{-1}	40
d_{tree}	0.518	5.90×10^{-1}	40

Table S7: Results for various criteria applied to the entries from miRNASNP-v3 related to cancer for which $N_{\text{mut seed}} \geq 1$ and $N_{\text{mut non-seed}} \geq 1$. The table contains areas under the ROC curves (A_{ROC}), and p-values based on the two-sided Mann-Whitney test. p-values of less than 0.05, which indicate criteria that perform significantly better than the random predictor (with $A_{\text{ROC}} = 0.5$), in **bold**.

Criterion	A_{ROC}	p-value	Number of unique mature miRNAs in the sample
$\Delta p_{\text{unfolded}}$	0.533	7.94×10^{-2}	143
$\Delta p_{\text{unfolded seed}}$	0.540	$\mathbf{3.44 \times 10^{-2}}$	143
$\Delta S^{(\text{i})}$	0.534	7.24×10^{-2}	143
$\langle S_{\text{mut}} \rangle$	0.515	4.18×10^{-1}	143
$\langle \Delta S \rangle$, all cases	0.494	7.42×10^{-1}	143
$\langle \Delta S \rangle$, folded mutant and WT	0.497	8.85×10^{-1}	109
$\langle \Delta S \rangle$, unfolded mutant and WT	0.475	5.85×10^{-1}	34
$\langle d_{\text{Hamming}} \rangle L^{-1}$	0.505	8.09×10^{-1}	143
$\langle d_{\text{Hamming}} \rangle$ percentile	0.540	$\mathbf{3.27 \times 10^{-2}}$	143
$d_{\text{Boltzmann}}$	0.519	3.08×10^{-1}	143
$\langle \Delta p_{\text{bp}} \rangle$	0.509	6.31×10^{-1}	143
d_{tree}	0.491	6.20×10^{-1}	143

Table S8: Results for various criteria applied to the entries from miRNASNP-v3 related to diseases other than cancer for which $N_{\mathrm{mut\ seed}} \geq 1$ and $N_{\mathrm{mut\ non-seed}} \geq 1$. The table contains areas under the ROC curves (A_{ROC}), and p-values based on the two-sided Mann-Whitney test. p-values of less than 0.05, which indicate criteria that perform significantly better than the random predictor (with $A_{\mathrm{ROC}} = 0.5$), in **bold**.

Criterion	A_{ROC}	p-value	Number of unique mature miRNAs in the sample
$\Delta p_{\mathrm{unfolded}}$	0.583	$\mathbf{4.04 \times 10^{-3}}$	46
$\Delta p_{\mathrm{unfolded\ seed}}$	0.521	4.70×10^{-1}	46
$\Delta S^{(\mathrm{i})}$	0.552	7.16×10^{-2}	46
$\langle S_{\mathrm{mut}} \rangle$	0.540	1.63×10^{-2}	46
$\langle \Delta S \rangle$, all cases	0.523	4.27×10^{-1}	46
$\langle \Delta S \rangle$, folded mutant and WT	0.545	1.75×10^{-1}	32
$\langle \Delta S \rangle$, unfolded mutant and WT	0.386	8.31×10^{-2}	14
$\langle d_{\mathrm{Hamming}} \rangle L^{-1}$	0.448	7.17×10^{-2}	46
$\langle d_{\mathrm{Hamming}} \rangle$ percentile	0.498	9.35×10^{-1}	46
$d_{\mathrm{Boltzmann}}$	0.442	$\mathbf{4.48 \times 10^{-2}}$	46
$\langle \Delta p_{\mathrm{bp}} \rangle$	0.509	7.48×10^{-1}	46
d_{tree}	0.527	3.06×10^{-1}	46

Table S9: Results for various criteria applied to the entries from miRNASNP-v3 related to cancer for which $N_{\text{mut seed}} \geq 2$ and $N_{\text{mut non-seed}} \geq 2$. The table contains areas under the ROC curves (A_{ROC}), and p-values based on the two-sided Mann-Whitney test. p-values of less than 0.05, which indicate criteria that perform significantly better than the random predictor (with $A_{\text{ROC}} = 0.5$), in **bold**.

Criterion	A_{ROC}	p-value	Number of unique mature miRNAs in the sample
$\Delta p_{\text{unfolded}}$	0.557	9.26×10^{-2}	23
$\Delta p_{\text{unfolded seed}}$	0.521	5.29×10^{-1}	23
$\Delta S^{(\text{i})}$	0.519	5.71×10^{-1}	23
$\langle S_{\text{mut}} \rangle$	0.570	$\mathbf{4.04 \times 10^{-2}}$	23
$\langle \Delta S \rangle$, all cases	0.539	2.56×10^{-1}	23
$\langle \Delta S \rangle$, folded mutant and WT	0.580	$\mathbf{3.86 \times 10^{-2}}$	18
$\langle \Delta S \rangle$, unfolded mutant and WT	0.419	3.68×10^{-1}	5
$\langle d_{\text{Hamming}} \rangle L^{-1}$	0.487	7.06×10^{-1}	23
$\langle d_{\text{Hamming}} \rangle$ percentile	0.531	3.70×10^{-1}	23
$d_{\text{Boltzmann}}$	0.519	5.80×10^{-1}	23
$\langle \Delta p_{\text{bp}} \rangle$	0.572	$\mathbf{3.47 \times 10^{-2}}$	23
d_{tree}	0.501	9.69×10^{-1}	23

Table S10: Results for various criteria applied to the entries from miRNASNP-v3 related to diseases other than cancer for which $N_{\mathrm{mut\ seed}} \geq 2$ and $N_{\mathrm{mut\ non-seed}} \geq 2$. The table contains areas under the ROC curves (A_{ROC}), and p-values based on the two-sided Mann-Whitney test. p-values of less than 0.05, which indicate criteria that perform significantly better than the random predictor (with $A_{\mathrm{ROC}} = 0.5$), in **bold**.

Criterion	A_{ROC}	p-value	Number of unique mature miRNAs in the sample
$\Delta p_{\mathrm{unfolded}}$	0.552	1.95×10^{-1}	12
$\Delta p_{\mathrm{unfolded\ seed}}$	0.508	8.40×10^{-1}	12
$\Delta S^{(\mathrm{i})}$	0.491	8.19×10^{-1}	12
$\langle S_{\mathrm{mut}} \rangle$	0.496	9.22×10^{-1}	12
$\langle \Delta S \rangle$, all cases	0.585	$\mathbf{3.33 \times 10^{-2}}$	12
$\langle \Delta S \rangle$, folded mutant and WT	0.615	$\mathbf{8.76 \times 10^{-3}}$	9
$\langle \Delta S \rangle$, unfolded mutant and WT	0.263	5.38×10^{-2}	3
$\langle d_{\mathrm{Hamming}} \rangle L^{-1}$	0.489	7.88×10^{-1}	12
$\langle d_{\mathrm{Hamming}} \rangle$ percentile	0.478	5.81×10^{-1}	12
$d_{\mathrm{Boltzmann}}$	0.459	3.01×10^{-1}	12
$\langle \Delta p_{\mathrm{bp}} \rangle$	0.540	3.13×10^{-1}	12
d_{tree}	0.514	7.11×10^{-1}	12

		cancer-related mutants		mutants related to other traits and diseases	
criterion 1	criterion 2	R_{Spearman}	p-value	R_{Spearman}	p-value
$\Delta p_{\text{unfolded}}$	$\langle d_{\text{Hamming}} \rangle$%ile	-0.091	2.57×10^{-27}	-0.066	7.12×10^{-6}
$\Delta p_{\text{unfolded}}$	$\Delta S^{(\text{i})}$	-0.417	≈ 0	-0.415	2.89×10^{-189}
$\Delta p_{\text{unfolded}}$	$\langle \Delta p_{\text{bp}} \rangle$	-0.303	1.35×10^{-298}	-0.313	3.14×10^{-104}
$\Delta S^{(\text{i})}$	$\langle d_{\text{Hamming}} \rangle$%ile	0.330	≈ 0	0.332	1.38×10^{-117}

Table S11: Spearman rank correlation coefficients and associated p-values between the three main criteria and the pair $\Delta p_{\text{unfolded}}$-$\langle \Delta p_{\text{bp}} \rangle$ calculated for miRNASNP-v3 with no filtering based on the number of mutations per miRNA. Most pairs of criteria display weak but significant correlations. Correlations are particularly weak between $\Delta p_{\text{unfolded}}$ and $\langle d_{\text{Hamming}} \rangle$%ile, indicating that the two can be treated as approximately uncorrelated. $\Delta p_{\text{unfolded}}$ is negatively correlated with $\langle \Delta p_{\text{bp}} \rangle$ as one may expect since a greater $\Delta p_{\text{unfolded}}$ means that the unfolded structure is more common in the ensemble and bases throughout the miRNA are more commonly unpaired.

		miRNASNP-v3 mutants, selected miRNAs	
criterion 1	criterion 2	R_{Spearman}	p-value
$\Delta p_{\text{unfolded}}$	$\langle d_{\text{Hamming}} \rangle$%ile	-0.355	2.17×10^{-11}
$\Delta p_{\text{unfolded}}$	$\Delta S^{(\text{i})}$	-0.563	≈ 0
$\Delta p_{\text{unfolded}}$	$\langle \Delta p_{\text{bp}} \rangle$	-0.453	≈ 0
$\Delta S^{(\text{i})}$	$\langle d_{\text{Hamming}} \rangle$%ile	0.406	9.07×10^{-15}

Table S12: Rank correlation coefficient data for a subset of the miRNASNP-v3 database limited to mutations in hsa-miR-485-5p, hsa-miR-1908-3p, hsa-miR-1269b, hsa-miR-4537, hsa-miR-4477b, hsa-miR-4641, hsa-miR-6821-3p. The trends observed are similar to those in Table S11 above.

4 Rank correlations between selected criteria

Tables S11-S13 contain correlation coefficients and associated p-values for the various datasets discussed in the main text.

		SomamiR mutants	
criterion 1	criterion 2	R_{Spearman}	p-value
$\Delta p_{\text{unfolded}}$	$\langle d_{\text{Hamming}} \rangle$%ile	-0.124	8.82×10^{-48}
$\Delta p_{\text{unfolded}}$	$\Delta S^{(\text{i})}$	-0.435	≈ 0
$\Delta p_{\text{unfolded}}$	$\langle \Delta p_{\text{bp}} \rangle$	-0.301	2.70×10^{-283}
$\Delta S^{(\text{i})}$	$\langle d_{\text{Hamming}} \rangle$%ile	0.351	≈ 0

Table S13: Rank correlation coefficient data from the SomamiR database, no filtering according to the number of mutations per miRNA. The trends observed are similar to those in Table S11 above.

5 Tables of p-values and other data for individual miRNAs

Table S14: Results for individual miRNAs from SomamiR for which $\Delta p_{\text{unfolded}}$ performs better than the random criterion ($p < 0.05$). The p-values are based on the two-sided Mann-Whitney test, and the SS of the WT is predicted with ViennaRNA. For each miRNA, we give lists of references connecting it to cancer and other traits and diseases.

miRNA	A_{ROC}	p-value	Predicted SS (WT)	Ref., cancer	Ref., other traits and diseases
hsa-miR-3689b-3p	0.076	3.55×10^{-2}	....		
hsa-miR-19a-3p	0.000	3.92×10^{-2}	..((((......))))).......	[6–8]	[9–11]
hsa-miR-548g-5p	0.082	3.92×10^{-2}	(((((......)))))).......	[12]	
hsa-miR-345-3p	1.000	4.17×10^{-2}	(((((......))))))......	[13]	[14]
hsa-miR-30e-5p	1.000	4.17×10^{-2}	........(((......)))..	[15–19]	[20–22]
hsa-miR-367-3p	1.000	4.17×10^{-2}	.(((((......)))))).....	[23]	

Table S15: Results for individual miRNAs from SomamiR for which $\langle d_{\text{Hamming}} \rangle L^{-1}$ performs better than the random criterion ($p < 0.05$). The p-values are based on the two-sided Mann-Whitney test, and the SS of the WT is predicted with ViennaRNA. For each miRNA, we give lists of references connecting it to cancer and other traits and diseases.

miRNA	A_{ROC}	p-value	Predicted SS (WT)	Ref., cancer	Ref., other traits and diseases
hsa-miR-3939	0.142	1.52×10^{-2}	.....(((..(....)..))).		[24]
hsa-miR-520g-3p	1.000	3.70×10^{-2}	.......((((.......))))..	[25]	
hsa-miR-635	1.000	3.92×10^{-2}	....(((((.((...)).)))))	[26, 27]	
hsa-miR-1185-1-3p	1.000	4.17×10^{-2}	......(((((....)))))..	[28]	[29]
hsa-miR-208a-3p	1.000	4.17×10^{-2}	.....((((((.....))))))		[30, 31]
hsa-miR-887-3p	1.000	4.17×10^{-2}	.....((((......))))...	[32, 33]	[34]
hsa-miR-192-3p	1.000	4.17×10^{-2}	..(((.........)))....	[35]	[36–38]
hsa-miR-1294	1.000	4.17×10^{-2}	.........((((....)))).	[39, 40]	[41]
hsa-miR-125b-2-3p	1.000	4.17×10^{-2}	((.((((.......)))).)).	[42, 43]	
hsa-miR-485-5p	0.913	4.43×10^{-2}	...((....))...........	[44–56]	[57]

Table S16: miRNAs for which the combined p-values from the $\Delta p_{\text{unfolded}}$ and $\langle d_{\text{Hamming}} \rangle L^{-1}$ criteria is below 0.05 based on data from SomamiR. miRNA identifiers in *italics* if the p-value for $\Delta p_{\text{unfolded}}$ is also less than 0.05 and **bold** if the p-value for $\langle d_{\text{Hamming}} \rangle L^{-1}$ is less than 0.05. As elsewhere, p-values are based on the two-sided Mann-Whitney test.

miRNA	combined p-value	Predicted SS (WT)	Ref., cancer	Ref., other traits and diseases
hsa-miR-3689b-3p	2.62×10^{-2}	....		
**hsa-miR-1185-1-3p**	3.26×10^{-2}	......(((((....)))))..	[28]	[29]
hsa-miR-558	3.29×10^{-2}	....	[58]	[59]
hsa-miR-129-1-3p	4.15×10^{-2}	....	[60]	[61]
hsa-miR-129-2-3p	4.15×10^{-2}	....	[62]	[61, 63–65]
**hsa-miR-3939**	4.16×10^{-2}	.....(((..(....)..))).		[24]
hsa-miR-543	4.29×10^{-2}	....	[66]	[66, 67]
**hsa-miR-208a-3p**	4.99×10^{-2}	.....((((((.....))))))		[30, 31]
**hsa-miR-887-3p**	4.99×10^{-2}	.....((((......))))....	[32, 33]	[34]

Table S17: Results for individual miRNAs from miRNASNP-v3 for which $\Delta p_{\mathrm{unfolded}}$ performs better than the random criterion ($p < 0.05$). The p-values are based on the two-sided Mann-Whitney test, and the SS of the WT is predicted with ViennaRNA. For each miRNA, we give lists of references connecting it to cancer and other traits and diseases. The names of the miRNAs for which the $\langle d_{\mathrm{Hamming}} \rangle L^{-1}$ criterion also performs significantly better than the random one are in **bold**.

miRNA	A_{ROC}	p-value	Predicted SS (WT)	Ref., cancer	Ref., other traits and diseases
**hsa-miR-3150a-5p**	0.000	1.77×10^{-3}	....		[68]
hsa-miR-345-3p	1.000	1.77×10^{-3}	(((((......)))))......	[13]	[14]
hsa-miR-580-5p	0.000	1.77×10^{-3}	...(((.((....)).)))...	[69]	
**hsa-miR-4641**	0.990	2.80×10^{-3}	....	[70]	
hsa-miR-519a-3p	0.052	3.58×10^{-3}	.....((((.((...)).))))	[71]	[72, 73]
**hsa-miR-4537**	0.746	5.17×10^{-3}	..(((.((((...)))).))).	[74]	
**hsa-miR-539-5p**	0.933	6.13×10^{-3}	(((.......)))).........	[75–78]	
hsa-miR-4756-3p	0.022	7.09×10^{-3}	(((....)))............	[14]	[79–81]
hsa-miR-6852-5p	0.023	8.08×10^{-3}	.(((.((...)).))).... .	[82]	
**hsa-miR-1269b**	0.033	1.06×10^{-2}	.(((......)))).........	[83–85]	
hsa-miR-548i	0.864	1.33×10^{-2}	..........(((.....))).	[86]	[87]
hsa-miR-548l	0.957	1.60×10^{-2}	....	[88]	[89, 90]
hsa-miR-208a-3p	0.054	2.13×10^{-2}	.....((((((.....))))))		[30, 31]
hsa-miR-6888-3p	0.942	2.42×10^{-2}	....		
hsa-miR-4649-3p	0.942	2.42×10^{-2}	...((((....))))......		[91, 92]
**hsa-miR-6821-3p**	0.930	3.23×10^{-2}	....		[93]
hsa-miR-4518	0.860	3.36×10^{-2}	.(((((.(..(....)..).))))).	[94, 95]	[96]
**hsa-miR-19a-5p**	0.924	3.55×10^{-2}	.((..((((....))))..)).	[97]	[98, 99]
hsa-miR-190b-5p	0.076	3.55×10^{-2}	.(((((.....)))))......	[100]	[101]
hsa-miR-1307-3p	0.924	3.55×10^{-2}	..((((((...)))))).... .		
hsa-miR-362-5p	1.000	3.70×10^{-2}	...(((........)))).......	[102]	
hsa-miR-608	0.918	3.76×10^{-2}	.(((((.((......)).)))))..	[103–109]	[110, 111]
hsa-miR-323b-5p	0.854	3.90×10^{-2}	.((....))((((.....)))).		[112, 113]
hsa-miR-548g-5p	0.082	3.92×10^{-2}	(((((......))))).......	[12]	
hsa-miR-657	0.000	3.92×10^{-2}	((.(((.......))).))....	[114, 115]	
hsa-miR-19b-2-5p	1.000	4.17×10^{-2}	.....((((....)))).....		[116]
hsa-miR-3116	1.000	4.17×10^{-2}	..((((........))))....	[117]	
hsa-miR-30e-5p	1.000	4.17×10^{-2}	........(((......)))..	[15–19]	[20–22]
hsa-miR-367-3p	1.000	4.17×10^{-2}	.(((((......))))).... .	[23]	
hsa-miR-382-5p	1.000	4.17×10^{-2}	(((....)))............	[118–122]	[123, 124]
**hsa-miR-558**	0.081	4.32×10^{-2}	....	[58]	[59]
hsa-miR-492	0.847	4.40×10^{-2}	.(..((...))..).........	[125–131]	
hsa-miR-518b	0.087	4.43×10^{-2}	....((.(((.......)))))		[132]
**hsa-miR-1307-5p**	1.000	4.44×10^{-2}	....((((.......))))..		
**hsa-miR-6893-5p**	1.000	4.44×10^{-2}	....	[133]	
**hsa-miR-892c-5p**	1.000	4.44×10^{-2}	....		
hsa-miR-520b-3p	1.000	4.44×10^{-2}	.........((((...)))).	[134]	
hsa-miR-8088	1.000	4.44×10^{-2}	((((...........))))..		[135]
hsa-miR-3120-5p	1.000	4.44×10^{-2}	......(((((.....)))))	[136]	
hsa-miR-3150b-3p	0.000	4.44×10^{-2}	(((.(....).)))).......	[137]	[138–140]
hsa-miR-563	0.807	4.64×10^{-2}	.((..(((....))).)).		[141–143]
hsa-miR-3689b-3p	0.156	4.69×10^{-2}	....		
**hsa-miR-1227-3p**	1.000	4.76×10^{-2}	....		[144–146]
**hsa-miR-6794-3p**	0.000	4.76×10^{-2}	....		[147]

Table S18: Results for individual miRNAs from miRNASNP-v3 for which $\langle d_{\mathrm{Hamming}} \rangle L^{-1}$ performs better than the random criterion ($p < 0.05$). The p-values are based on the two-sided Mann-Whitney test, and the SS of the WT is predicted with ViennaRNA. For each miRNA, we give lists of references connecting it to cancer and other traits and diseases. The names of the miRNAs for which the $\Delta p_{\mathrm{unfolded}}$ criterion also performs significantly better than the random one are in **bold**.

miRNA	A_{ROC}	p-value	Predicted SS (WT)	Ref., cancer	Ref., other traits and diseases
hsa-miR-1908-3p	0.939	1.26×10^{-3}	((((...))))..........		
hsa-miR-4477b	1.000	1.77×10^{-3}	(((((..((....))..)))))	[148]	
hsa-miR-4722-3p	1.000	1.77×10^{-3}	..((((.((.......))))))	[149]	[150]
**hsa-miR-1269b**	0.000	1.77×10^{-3}	.(((......)))..........	[83–85]	
hsa-miR-485-5p	0.926	1.99×10^{-3}	...((....))..........	[44–56]	[57]
**hsa-miR-4641**	0.981	5.59×10^{-3}	....	[70]	
**hsa-miR-3150a-5p**	0.022	7.09×10^{-3}	....		[68]
**hsa-miR-19a-5p**	0.043	1.60×10^{-2}	.((..((((....))))..)).	[97]	[98, 99]
**hsa-miR-4537**	0.289	1.76×10^{-2}	..(((.((((...)))).))).	[74]	
hsa-miR-6800-3p	0.047	1.82×10^{-2}	..........(((...)))..		
hsa-miR-99a-3p	0.946	2.13×10^{-2}	...(((((......)))))...		
**hsa-miR-558**	0.054	2.43×10^{-2}	....	[58]	[59]
hsa-miR-3939	0.195	2.49×10^{-2}	.....(((..(....)..))).		[24]
hsa-miR-513c-5p	0.935	2.84×10^{-2}	.((((...))))..........	[151, 152]	[153]
hsa-miR-148b-5p	0.065	2.84×10^{-2}	....((((.........)))).		[154, 155]
hsa-miR-4802-3p	0.071	3.14×10^{-2}	....((((.((...))))))...	[156]	
hsa-miR-1248	1.000	3.17×10^{-2}	............((((...))))...	[26, 157]	[158]
hsa-miR-509-5p	0.930	3.23×10^{-2}	(((((....)))))).......	[159–161]	[162, 163]
hsa-miR-3130-3p	0.930	3.23×10^{-2}	..(((.((((...))))))).	[164]	[165]
**hsa-miR-6821-3p**	0.930	3.23×10^{-2}	....		[93]
hsa-miR-550a-5p	0.819	3.35×10^{-2}	.........(((........)))	[166]	[167]
hsa-miR-887-3p	0.924	3.55×10^{-2}	.....((((......))))...	[32, 33]	[34]
hsa-miR-624-3p	0.857	3.86×10^{-2}	....(((((.......)))))	[168]	
hsa-miR-103a-1-5p	1.000	3.92×10^{-2}	(((..(........)..)))...		
hsa-miR-302a-5p	1.000	3.92×10^{-2}	........(((....)))).....		[169]
hsa-miR-635	1.000	3.92×10^{-2}	....(((((.((...)).)))))	[26, 27]	
**hsa-miR-539-5p**	0.148	4.14×10^{-2}	(((.......)))..........	[75–78]	
hsa-miR-125b-2-3p	1.000	4.17×10^{-2}	((.((((.......)))).)).	[42, 43]	
hsa-miR-1185-1-3p	1.000	4.17×10^{-2}	......(((((....)))))..	[28]	[29]
hsa-miR-3915	1.000	4.17×10^{-2}	...((((........))))...	[170]	
hsa-miR-5195-5p	1.000	4.17×10^{-2}	..((....((....))....))		[171]
hsa-miR-5682	1.000	4.17×10^{-2}	.....((((((.....))))))	[172]	
hsa-miR-199a-3p	0.000	4.17×10^{-2}	.(((....)))...........	[173–176]	[177]
hsa-miR-381-5p	0.000	4.17×10^{-2}	.((((((.....))))))....	[178–180]	
hsa-miR-3689a-3p	0.193	4.30×10^{-2}	(((.((.........)).))).		
hsa-miR-328-5p	0.847	4.40×10^{-2}	....((((.......))))....	[181]	[36]
hsa-miR-500a-3p	0.913	4.43×10^{-2}	.....(((.....)))......	[182, 183]	
hsa-miR-4708-3p	0.913	4.43×10^{-2}	.((......))...........		[184]
hsa-miR-378i	1.000	4.44×10^{-2}	.(((.(((...))))))....	[185–187]	
**hsa-miR-892c-5p**	1.000	4.44×10^{-2}	....		
hsa-miR-3155a	1.000	4.44×10^{-2}	(((.((...))..)))....		[188]
**hsa-miR-6893-5p**	1.000	4.44×10^{-2}	....	[133]	
hsa-miR-376c-3p	0.000	4.44×10^{-2}	........(((...)))....	[189–191]	
hsa-miR-892a	0.000	4.44×10^{-2}	..((((((.......))))))	[192–195]	
**hsa-miR-1307-5p**	0.000	4.44×10^{-2}	....((((.......))))..		
hsa-miR-508-5p	0.803	4.49×10^{-2}	.......(((.......)))...	[196]	[197]
**hsa-miR-1227-3p**	1.000	4.76×10^{-2}	....		[144–146]
**hsa-miR-6794-3p**	0.000	4.76×10^{-2}	....		[147]

Table S19: miRNAs for which the combined p-values from the $\Delta p_{\text{unfolded}}$ and $\langle d_{\text{Hamming}} \rangle L^{-1}$ criteria is below 0.05 based on data from miRNASNP-v3. miRNA identifiers in *italics* if the p-value for $\Delta p_{\text{unfolded}}$ is also less than 0.05, **bold** if the p-value for $\langle d_{\text{Hamming}} \rangle L^{-1}$ is less than 0.05, and ***bold italics*** - if both are below 0.05. As elsewhere, p-values are based via the two-sided Mann-Whitney test.

miRNA	combined p-value	Predicted SS (WT)	Ref., cancer	Ref., other traits and diseases
hsa-miR-3150a-5p	1.54×10^{-4}	....		[68]
hsa-miR-4641	1.89×10^{-4}	....	[70]	
hsa-miR-1269b	2.24×10^{-4}	.(((......)))..........	[83–85]	
hsa-miR-4537	9.36×10^{-4}	..(((.((((...)))).))).	[74]	
**hsa-miR-4477b**	1.14×10^{-3}	(((((..((....))..)))))	[148]	
**hsa-miR-1908-3p**	1.69×10^{-3}	((((...))))..........		
hsa-miR-345-3p	1.91×10^{-3}	(((((......)))))......	[13]	[14]
hsa-miR-539-5p	2.35×10^{-3}	(((.......)))..........	[75–78]	
**hsa-miR-485-5p**	3.84×10^{-3}	...((....))...........	[44–56]	[57]
**hsa-miR-4722-3p**	3.94×10^{-3}	..((((.((.......))))))	[149]	[150]
hsa-miR-19a-5p	4.80×10^{-3}	.((..((((....))))..)).	[97]	[98, 99]
hsa-miR-548l	6.85×10^{-3}	....	[88]	[89, 90]
hsa-miR-6821-3p	8.22×10^{-3}	....		[93]
hsa-miR-558	8.25×10^{-3}	....	[58]	[59]
hsa-miR-4756-3p	8.81×10^{-3}	(((....)))............	[14]	[79–81]
hsa-miR-6888-3p	1.11×10^{-2}	....		
hsa-miR-580-5p	1.14×10^{-2}	...(((.((....)).)))...	[69]	
hsa-miR-892c-5p	1.43×10^{-2}	....		
hsa-miR-1307-5p	1.43×10^{-2}	....((((.......))))..		
hsa-miR-6893-5p	1.43×10^{-2}	....	[133]	
hsa-miR-1227-3p	1.61×10^{-2}	....		[144–146]
hsa-miR-6794-3p	1.61×10^{-2}	....		[147]
**hsa-miR-6800-3p**	1.66×10^{-2}	..........(((...)))..		
hsa-miR-4434	2.09×10^{-2}	....		[198]
hsa-miR-519a-3p	2.12×10^{-2}	.....((((.((...)).))))	[71]	[72, 73]
hsa-miR-563	2.29×10^{-2}	.((..(((....))).)).		[141–143]
**hsa-miR-199a-3p**	2.31×10^{-2}	.(((....)))...........	[173–176]	[177]
**hsa-miR-5682**	2.31×10^{-2}	.....((((((.....))))))	[172]	
hsa-miR-4296	2.43×10^{-2}	...(((((....)))))	[199]	[200]
hsa-miR-548i	2.48×10^{-2}	..........(((.....))).	[86]	[87]
hsa-let-7f-1-3p	2.65×10^{-2}	....	[201]	[202–204]
hsa-miR-3689b-3p	2.75×10^{-2}	....		
hsa-miR-492	2.83×10^{-2}	.(..((...))..).........	[125–131]	
hsa-miR-4518	2.87×10^{-2}	.(((((.(..(....)..).))))).	[94, 95]	[96]
hsa-miR-4649-3p	3.09×10^{-2}	...((((....))))......		[91, 92]
**hsa-miR-1185-1-3p**	3.26×10^{-2}	......(((((....)))))..	[28]	[29]
**hsa-miR-4802-3p**	3.37×10^{-2}	....((((.((...))))))...	[156]	
**hsa-miR-513c-5p**	3.43×10^{-2}	.((((...))))..........	[151, 152]	[153]
hsa-miR-208a-3p	3.61×10^{-2}	.....((((((.....))))))		[30, 31]
hsa-miR-6852-5p	3.77×10^{-2}	.(((.((...)).)))....	[82]	
hsa-miR-19b-2-5p	4.15×10^{-2}	.....((((....)))).....		[116]
hsa-miR-129-2-3p	4.15×10^{-2}	....	[62]	[61, 63–65]
**hsa-miR-3915**	4.15×10^{-2}	...((((........))))...	[170]	
hsa-miR-4441	4.34×10^{-2}	((((........)))).	[205]	
**hsa-miR-376c-3p**	4.61×10^{-2}	........(((...)))....	[189–191]	
hsa-miR-520b-3p	4.61×10^{-2}	.........((((...)))).	[134]	
**hsa-miR-3130-3p**	4.61×10^{-2}	..(((.((((...))))))).	[164]	[165]
hsa-miR-6769b-3p	4.61×10^{-2}	....	[206, 207]	
hsa-miR-7151-3p	4.61×10^{-2}	.....((((......))))..		[208]

6 Table of disease-associated mutations that convert one WT miRNA to another

Table S20: Data on mutations that convert one miRNA to another and are associated with disease; data from SomamiR and miRNASNP-v3.

miRNA WT	mutated miRNA	WT sequence	mutated sequence	SS, WT	SS, mutant	$\langle d_{\mathrm{Hamming}}\rangle \times L^{-1}$	$\Delta p_{\mathrm{unfolded}}$
hsa-miR-1269a	hsa-miR-1269b	CUGGACUGAGCCAUGCUACUGG	CUGGACUGAGCCGUGCUACUGG	.(((......))).........	.(((......))).........	0.35	-0.0086
hsa-miR-3689a-3p	hsa-miR-3689c, hsa-miR-3689b-3p	CUGGGAGGUGUGAUAUUGUGGU	CUGGGAGGUGUGAUAUCGUGGU	(((.((.........)).))).	....	0.34	0.51
hsa-miR-3689b-3p	hsa-miR-3689a-3p	CUGGGAGGUGUGAUAUCGUGGU	CUGGGAGGUGUGAUAUUGUGGU	....	(((.((.........)).))).	0.34	-0.51
hsa-miR-3689c	has-miR-3689a-3p	CUGGGAGGUGUGAUAUCGUGGU	CUGGGAGGUGUGAUAUUGUGGU	....	(((.((.........)).))).	0.34	-0.51
hsa-miR-518e-5p	hsa-miR-526a-5p, hsa-miR-520c-5p, hsa-miR-518d-5p	CUCUAGAGGGAAGCACUUUCUG	CUCUAGAGGGAAGCGCUUUCUG	...(((((((.....)))))))	...(((((((.....)))))))	0.084	3.2×10^{-4}
hsa-miR-519a-5p	hsa-miR-526a-5p, hsa-miR-520c-5p, hsa-miR-518d-5p	CUCUAGAGGGAAGCACUUUCUG	CUCUAGAGGGAAGCGCUUUCUG	...(((((((.....)))))))	...(((((((.....)))))))	0.084	3.2×10^{-4}
hsa-miR-520c-5p	hsa-miR-519c-5p, hsa-miR-519b-5p, hsa-miR-523-5p, hsa-miR-518e-5p, hsa-miR-522-5p, hsa-miR-519a-5p	CUCUAGAGGGAAGCGCUUUCUG	CUCUAGAGGGAAGCACUUUCUG	...(((((((.....)))))))	...(((((((.....)))))))	0.084	-3.2×10^{-4}
hsa-miR-522-5p	has-miR-526a-5p, hsa-miR-520c-5p, hsa-miR-518d-5p	CUCUAGAGGGAAGCACUUUCUG	CUCUAGAGGGAAGCGCUUUCUG	...(((((((.....)))))))	...(((((((.....)))))))	0.084	3.2×10^{-4}
hsa-miR-548au-5p	has-miR-548ar-5p	AAAAGUAAUUGCAGUUUUUGC	AAAAGUAAUUGCGGUUUUUGC	....	.........(((.....)))	0.26	-0.15
hsa-miR-548au-5p	has-miR-548ay-5p	AAAAGUAAUUGUGGUUUUUGC	AAAAGUAAUUGCGGUUUUUGC	....	....	0.31	0.18
hsa-miR-519a-5p	has-miR-526a-5p, hsa-miR-520c-5p, hsa-miR-518d-5p	CUCUAGAGGGAAGCACUUUCUG	CUCUAGAGGGAAGCGCUUUCUG	...(((((((.....)))))))	...(((((((.....)))))))	0.084	3.2×10^{-4}

Table S21: Results for various criteria applied to the entries from miRNASNP-v3 that concern hsa-miR-4537. The table contains areas under the ROC curves (A_{ROC}), as well as p-values based on the two-sided Mann-Whitney test. p-values of less than 0.05, which indicate criteria that perform significantly better than the random predictor (with $A_{\mathrm{ROC}} = 0.5$), in **bold**.

Criterion	A_{ROC}	p-value
$\Delta p_{\mathrm{unfolded}}$	0.746	$\mathbf{5.17 \times 10^{-3}}$
$\Delta p_{\mathrm{unfolded\ seed}}$	0.509	9.27×10^{-1}
$\Delta S^{(\mathrm{i})}$	0.473	7.70×10^{-1}
$\langle S_{\mathrm{mut}} \rangle$	0.744	$\mathbf{5.57 \times 10^{-3}}$
$\langle \Delta S \rangle$, all cases	0.744	$\mathbf{5.57 \times 10^{-3}}$
$\langle d_{\mathrm{Hamming}} \rangle L^{-1}$	0.289	$\mathbf{1.76 \times 10^{-2}}$
$d_{\mathrm{Boltzmann}}$	0.309	$\mathbf{3.19 \times 10^{-2}}$
$\langle \Delta p_{\mathrm{bp}} \rangle$	0.309	$\mathbf{3.19 \times 10^{-2}}$
d_{tree}	0.665	5.59×10^{-2}

7 Case studies

7.1 hsa-miR-4537

We take the example of one of hsa-miR-4537, one of the individual miRNAs that exhibit a significant effect of secondary structure after adjusting for multiple hypothesis testing. The database contains a total of 20 entries for disease-associated mutations affecting this miRNA, out of which there are 16 unique point mutants with an unaffected seed region.

In Figure S17 we demostrate that all applicable criteria except for $\Delta p_{\mathrm{unfolded}}$ and $\Delta p_{\mathrm{unfolded\ seed}}$ perform significantly better than random for this individual microRNA. We do not show the results for the two criteria based on $\langle d_{\mathrm{Hamming}} \rangle$ separately because the percentile ranking is fully equivalent to the one by the normalized Hamming distance. This likely indicates that, while the effect of secondary structure is by no means the only factor that determines the association of miRNAs with disease, it plays a significant role in some cases. Moreover, hsa-mir-4537 is a tumour-suppressor miRNA relevant to gastric cancer [74], and, as one would expect if the secondary structure affected activity, the mutants that change the folding most tend to be associated with disease, leading to $A_{\mathrm{ROC}} > 0.5$ for $\Delta p_{\mathrm{unfolded}}$ and $A_{\mathrm{ROC}} < 0.5$ for $\langle d_{\mathrm{Hamming}} \rangle L^{-1}$. As one would expect if the secondary structure affected activity, the mutants that change the folding most tend to be associated with disease, leading to $A_{\mathrm{ROC}} > 0.5$ for $\Delta p_{\mathrm{unfolded}}$ and $A_{\mathrm{ROC}} < 0.5$ for $\langle d_{\mathrm{Hamming}} \rangle L^{-1}$. We provide additional data on this miRNA in Figure S18, which contains the ROC curves underlying Figure S17, and Table S21, which gives the values of A_{ROC} for the various criteria.

7.2 hsa-miR-485-5p

We also present the results of applying the various criteria to data for hsa-miR-485-5p, for which the p-value that quantifies the significance of the result for $\langle d_{\mathrm{Hamming}} \rangle / L$ is the lowest ($p = 5.45 \times 10^{-3}$). The miRNASNP-v3 database contains a total of 6 entries for disease-associated mutations affecting this miRNA, out of which there are 4 unique point mutants with an unaffected seed region.

As Figure S19 illustrates, all of our criteria except for $\Delta p_{\mathrm{unfolded}}$ and $\Delta p_{\mathrm{unfolded\ seed}}$ perform significantly better than random for this individual microRNA. This provides further support for the hypothesis that secondary structure impacts the function of some miRNAs. This miRNA is known to act as an inhibitor of breast cancer progression [51]; despite that, it is the mutants with secondary structure close to that of the WT that tend to be associated with disease, as indicated by the A_{ROC} value for $\langle d_{\mathrm{Hamming}} \rangle / L$ in the figure

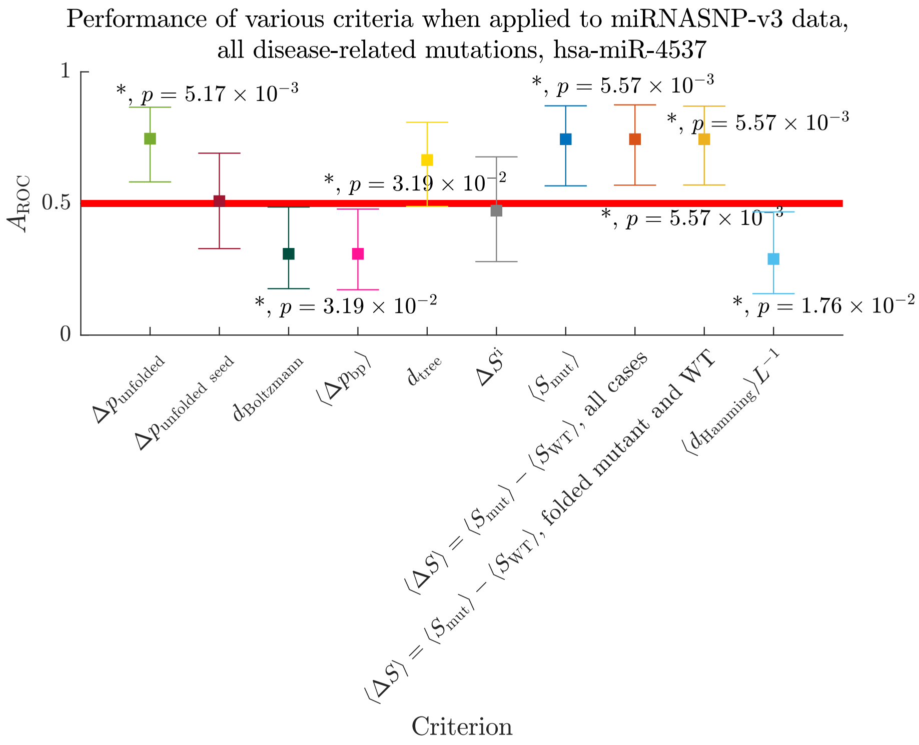

Figure S17: **Multiple independent SS-based criteria predict association of miRNA mutations with disease better than random for hsa-miR-4537.** Comparison of the performance of various criteria in terms of predicting disease-associated mutations when applied to mutations concerning hsa-miR-4537 from miRNASNP-v3. hsa-miR-4537 is known to play a tumour-suppressing role in gastric cancer [74]. Squares mark A_{ROC} values, error bars show 95% confidence intervals calculated with the bootstrapping method [209], and Mann-Whitney p-values are indicated wherever $p < 0.05$. The red horizontal line indicates the area under the curve for the random criterion, $A_{\mathrm{ROC}} = 0.5$. The only criteria that do not perform significantly better than the random one for this dataset are $\Delta p_{\mathrm{unfolded\ seed}}$, which measures the change in probability that the miRNA seed region is fully unfolded and ΔS^{i}, which measures the change in the positional entropy of the mutated site. The comparatively large values of A_{ROC} suggest that miRNA folding plays a role in disease and therefore miRNA activity.

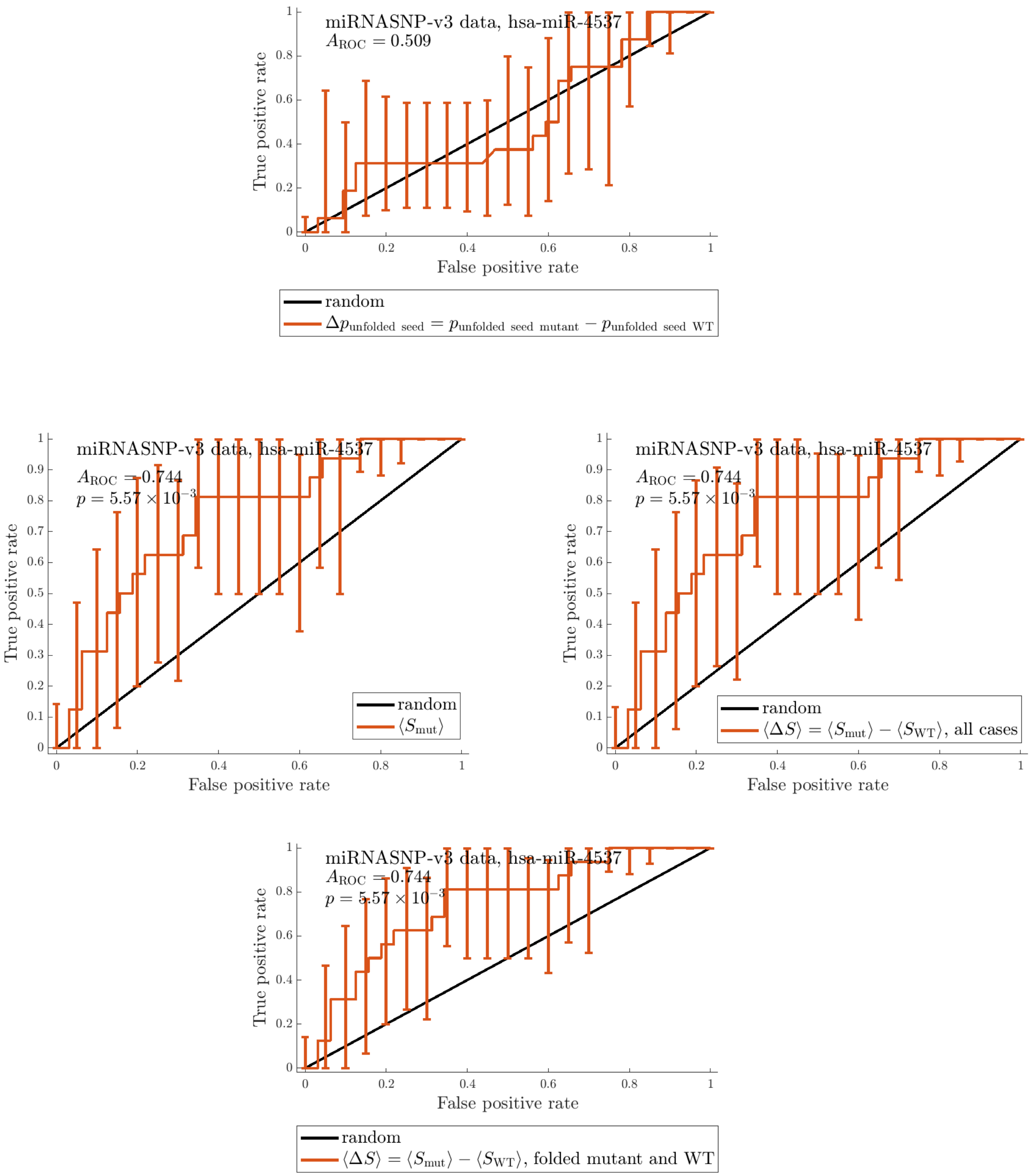

Figure S18: ROC curves characterizing the performance of all criteria discussed above for mutations affecting hsa-miR-4537 from miRNASNP-v3.

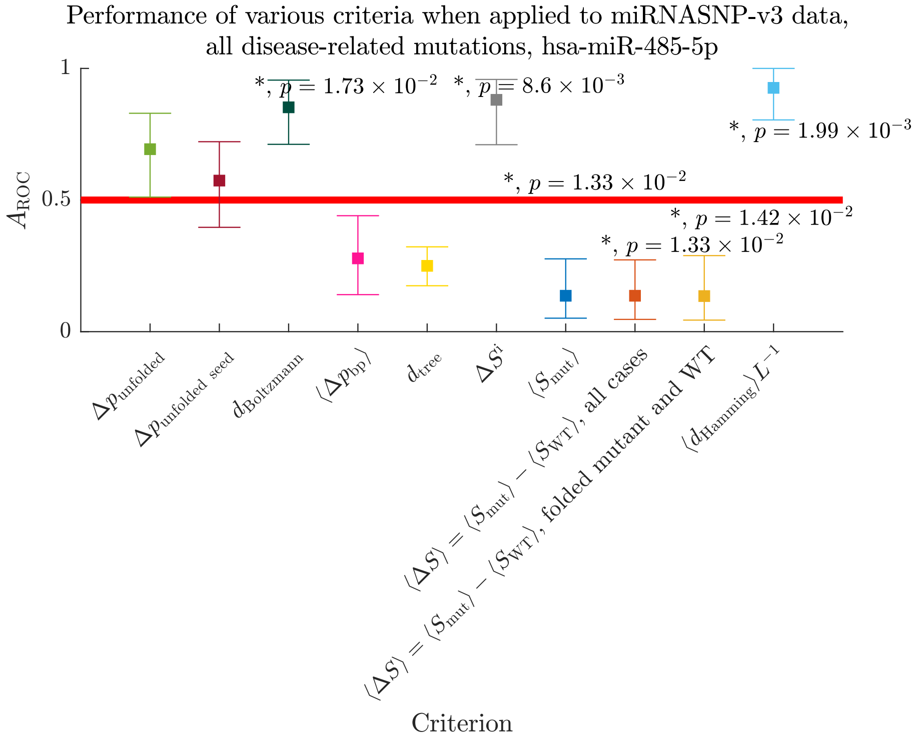

Figure S19: **Multiple independent SS-based criteria predict association of miRNA mutations with disease better than random for hsa-miR-485-5p.** Comparison of the performance of various criteria in terms of predicting disease-associated mutations when applied to mutations concerning hsa-miR-485-5p from miRNASNP-v3. hsa-miR-485-5p is associated with various types of cancer, including lung cancer, breast cancer and others [44–56] and cerebral ischemia [57]. Squares mark A_{ROC} values, error bars show 95% confidence intervals, and Mann-Whitney p-values are indicated wherever $p < 0.05$. The red horizontal lines indicate the area under the curve for the random criterion, $A_{\mathrm{ROC}} = 0.5$. The only criteria that do not perform significantly better than the random one for this dataset are those based on the probability of folded states. The comparatively large values of A_{ROC} suggest that miRNA folding plays a role in disease and therefore miRNA activity.

($A_{\mathrm{ROC}} = 0.926$). We provide additional data on this miRNA in Figure S20, which contains the ROC curves underlying Figure S19, and Table S22, which gives the relevant areas under the ROC curves.

8 Additional information on miRNAs with $q < 0.05$

When we apply the strictest level of filtering, $N_{\mathrm{mut\ seed}} \geq 3$ and $N_{\mathrm{mut\ non-seed}} \geq 3$, to the dataset with mutations associated with any traits and diseases, we find one miRNA with a q-value that passes our significance threshold for the criterion based on the mutant-WT SS Hamming distance $\langle d_{\mathrm{Hamming}} \rangle / L$ criterion - $q = 3.72 \times 10^{-2}$ for **hsa-miR-4477b**.

Additionally, we calculate the Benjamini-Hochberg q-values for the combined p-values calculated via the Fisher method [210] for $\Delta p_{\mathrm{folded}}$ and $\langle d_{\mathrm{Hamming}} \rangle / L$ of the individual miRNAs represented in SomamiR and miRNASNP-v3. When we analyse the mutations from miRNASNP-v3 that pertain to all diseases and traits with applied filtering by $N_{\mathrm{mut\ seed}}$ and $N_{\mathrm{mut\ non-seed}}$, we find q-values are below 0.05 for several miRNAs. At $N_{\mathrm{mut\ seed}} \geq 1$ and $N_{\mathrm{mut\ non-seed}} \geq 1$, $q = 2.67 \times 10^{-2}$ for **hsa-miR-4641** and **hsa-miR-1269b**. For the smaller subset with $N_{\mathrm{mut\ seed}} \geq 2$ and $N_{\mathrm{mut\ non-seed}} \geq 2$, $q = 1.86 \times 10^{-2}$ for **hsa-miR-4537** and

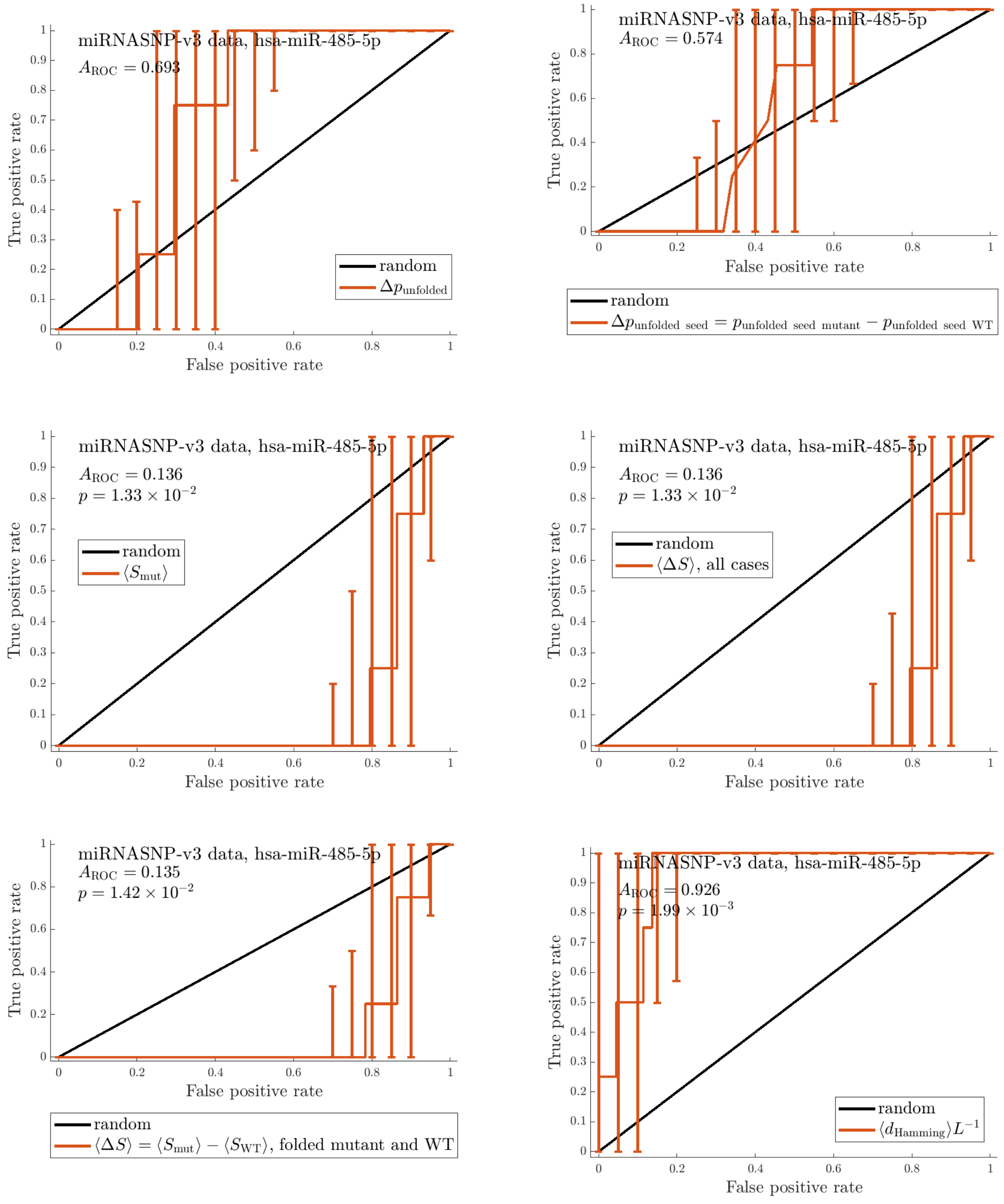

Figure S20: ROC curves characterizing the performance of all criteria discussed above for mutations affecting hsa-miR-485-5p from miRNASNP-v3.

Table S22: Results for various criteria applied to the entries from miRNASNP-v3 that concern hsa-miR-485-5p. The table contains areas under the ROC curves (A_{ROC}), and p-values based on the two-sided Mann-Whitney test. p-values of less than 0.05, which indicate criteria that perform significantly better than the random predictor (with $A_{\mathrm{ROC}} = 0.5$), in **bold**.

Criterion	A_{ROC}	p-value
$\Delta p_{\mathrm{unfolded}}$	0.693	2.21×10^{-1}
$\Delta p_{\mathrm{unfolded\ seed}}$	0.574	6.47×10^{-1}
$\Delta S^{(\mathrm{i})}$	0.881	$\mathbf{8.60 \times 10^{-3}}$
$\langle S_{\mathrm{mut}} \rangle$	0.136	$\mathbf{1.33 \times 10^{-2}}$
$\langle \Delta S \rangle$, all cases	0.136	$\mathbf{1.33 \times 10^{-2}}$
$\langle d_{\mathrm{Hamming}} \rangle L^{-1}$	0.926	$\mathbf{1.99 \times 10^{-3}}$
$d_{\mathrm{Boltzmann}}$	0.852	$\mathbf{1.73 \times 10^{-2}}$
$\langle \Delta p_{\mathrm{bp}} \rangle$	0.278	1.56×10^{-1}
d_{tree}	0.250	1.54×10^{-1}

hsa-miR-4477b, $q = 1.10 \times 10^{-2}$ for hsa-miR-1269b, and the q-value is further reduced to $q = 1.31 \times 10^{-2}$ for **hsa-miR-4537** and **hsa-miR-4477b** based on the set with $N_{\mathrm{mut\ seed}} \geq 3$ and $N_{\mathrm{mut\ non-seed}} \geq 3$.

9 Distributions of $\langle d_{\mathrm{Hamming}} \rangle$ for disease-related mutations in mature miRNAs and their precursors

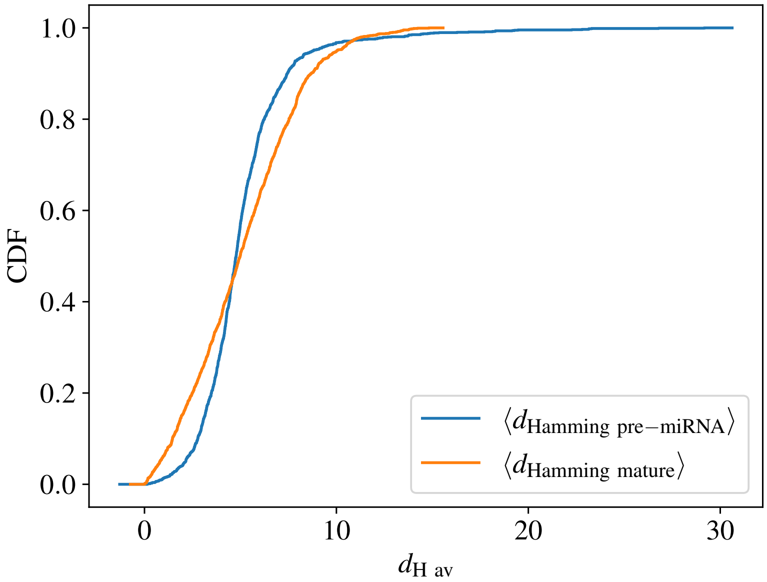

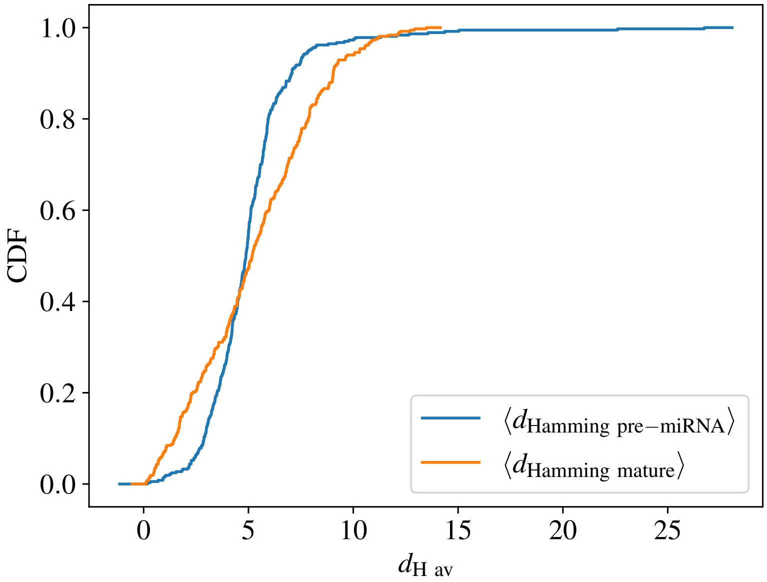

Figure S21: Cumulative distribution functions of $\langle d_{\mathrm{Hamming}} \rangle$ for the disease-related mutations in miRNASNP-v3 (top) and SomamiR (bottom) in mature miRNAs and their precursors. The average Hamming distance is not significantly different between precursors and mature forms, meaning that secondary structure changes in mature forms are greater relative to sequence length.

10 Distributions of $\Delta_{\max}$ for disease-related mutations

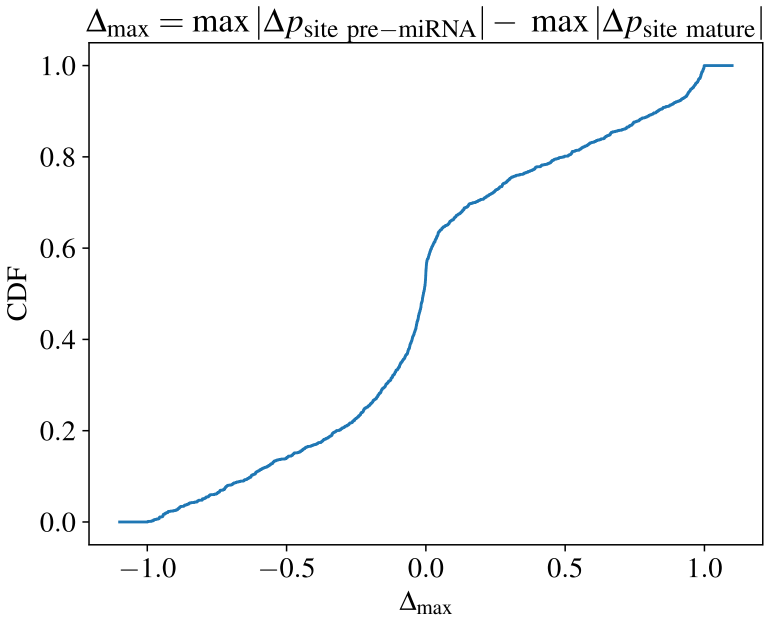

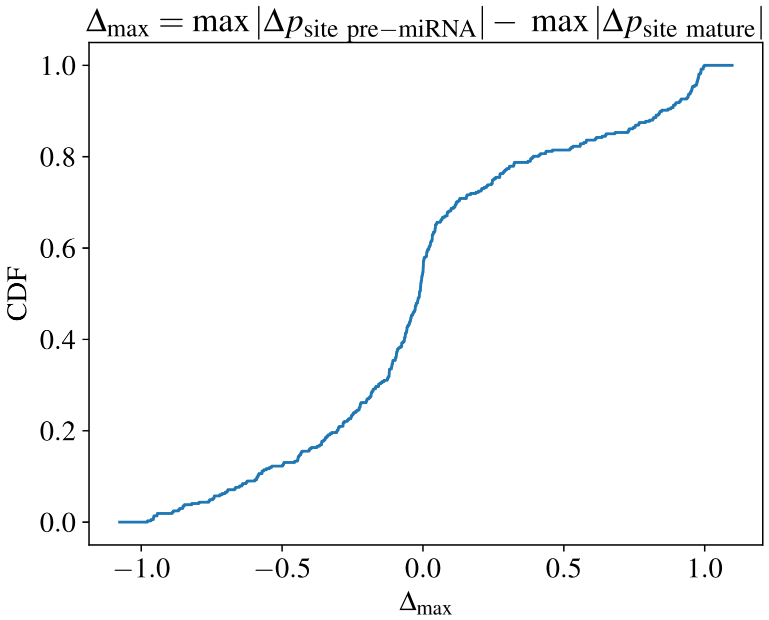

Figure S22: Cumulative distribution functions of $\Delta_{\max}$ for the disease-related mutations in miRNASNP-v3 (top) and SomamiR (bottom) in mature miRNAs and their precursors. Note that for some mutations, $\Delta_{\max} \sim -1$, meaning that the base-pairing at no sensitive site in the precursor is affected, but a site in the mature form is strongly affected.

Table S23: AlphaFold 3 predictions for the secondary structure of miRNA WTs and mutants in complex with Argonaute proteins. plDDT values are averaged over all nucleotides.

ID	sequence	SS	average plDDT
Argonaute 1, mutant hsa-miR-20a 16C>U	UAAAGUGCUUAUAGUGUAGGUAG	....	70.7
Argonaute 1, mutant hsa-miR-20a 8C>A	UAAAGUGAUUAUAGUGCAGGUAG	....	74.2
Argonaute 1, mutant hsa-miR-654-3p	UAUGUCUGCUUACCAUCACCUU	....	72.7
Argonaute 1, mutant hsa-miR-6869-3p	CGCCGCGCGCAGCGGCUCAGC	.(((((.....))))).....	68.9
Argonaute 1, mutant hsa-miR-6869-3p with maximum $\langle d_{\text{Hamming mutant}} \rangle$	CGCCGCGAGCAUCGGCUCAGC	......((((....))))...	74.2
Argonaute 1, mutant hsa-miR-6869-3p with maximum $\Delta p_{\text{unfolded}}$	CGCCGCGCGCAUCGACUCAGC	....	81.7
Argonaute 2, mutant hsa-let-7c-3p	CUGUACAAGCUUCUAGCUUUCC	....	72.2
Argonaute 2, WT hsa-let-7c-3p	CUGUACAACCUUCUAGCUUUCC	....	88.6
Argonaute 2, mutant hsa-miR-211-5p	UUCCCUUUGGCAUCCUUCGCCU	....	74.3
Argonaute 2, WT hsa-miR-211-5p	UUCCCUUUGUCAUCCUUCGCCU	....	77.1
Argonaute 2, mutant hsa-miR-3144-3p	AUAUACCUGUUCGCUCUCUUUA	....	76.2
Argonaute 2, WT hsa-miR-3144-3p	AUAUACCUGUUCGGUCUCUUUA	....	70.4
Argonaute 2, mutant hsa-miR-574-3p	CACGCUCAUUCACACACCCACA	....	73.5
Argonaute 2, WT hsa-miR-574-3p	CACGCUCAUGCACACACCCACA	....	76.1
Argonaute 2, mutant hsa-miR-769-3p	CUGGGAUCCCCGGGGUCUUGGUU	....	64.7
Argonaute 2, mutant hsa-miR-6794-3p	CUCACUCUGAGUCCCUCCCU	....	73.2
Argonaute 2, mutant hsa-miR-6869-3p	CGCCGCGCGCAGCGGCUCAGC	.(((((.....))))).....	68.0
Argonaute 2, mutant hsa-miR-6869-3p with maximum $\langle d_{\text{Hamming mutant}} \rangle$	CGCCGCGAGCAUCGGCUCAGC	....((((((....)))).))	76.4
Argonaute 2, mutant hsa-miR-6869-3p with maximum $\Delta p_{\text{unfolded}}$	CGCCGCGCGCAUCGACUCAGC	....	79.7
Argonaute 2, WT hsa-miR-6869-3p	CGCCGCGCGCAUCGGCUCAGC	....	74.2

11 AlphaFold3 studies of miRNA-Argonaute complexes

We used AlphaFold 3 [211] to study the secondary and tertiary structure of complexes between selected miRNAs and the Argonaute 1 and 2 proteins, whose sequences we took from the Protein DataBank entries 4KXT and 4F3T, respectively. We studied several WT miRNAs and point mutants in them, tabulated below. In each case, we took the top-scoring prediction as ranked by AlphaFold 3 and parsed its secondary structure with the DSSR software package [212]. We list the studied miRNA sequences and the predicted secondary structures for them in Table S23. We provide all five AlphaFold 3 predictions for the 3D-structure of the complexes in the form of CIF files with the online supplementary information alongside output from DSSR for the top-rated prediction.

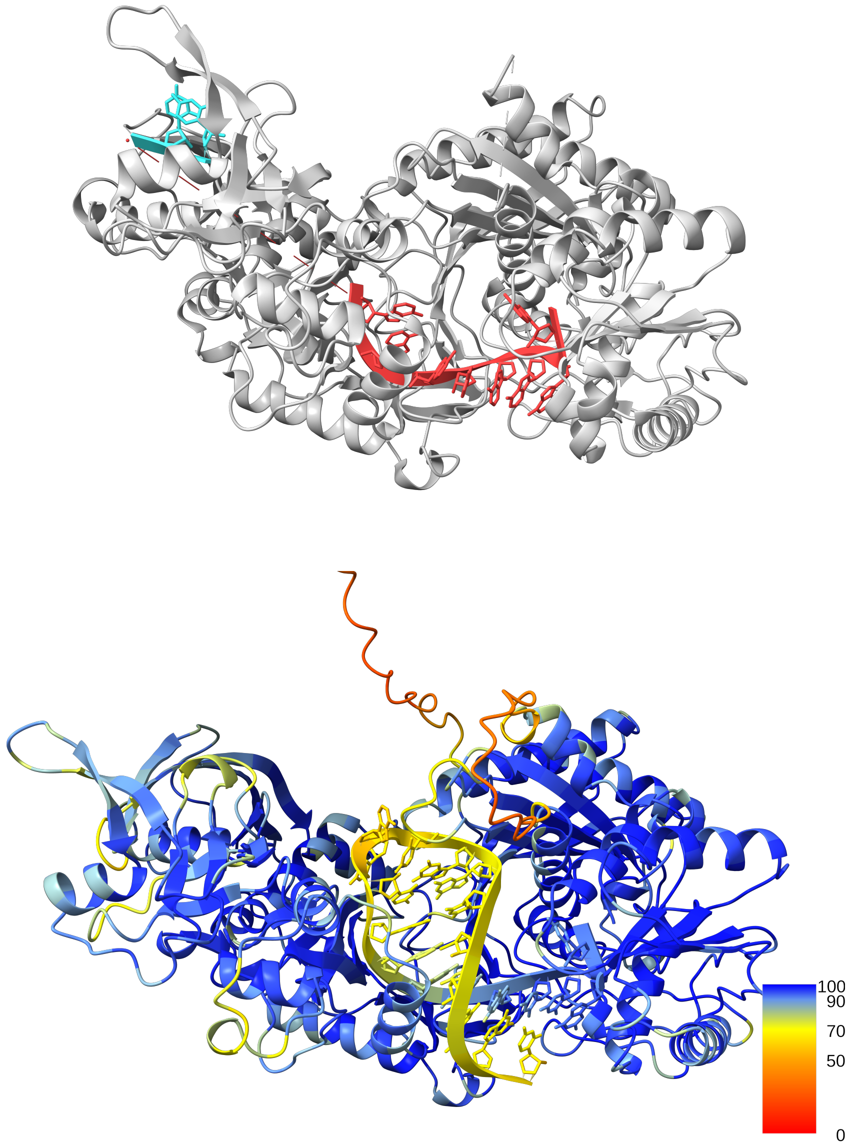

Figure S23: **AlphaFold 3 predicts non-trivial secondary structure in the complex of a point mutant of hsa-miR-6869-3p and Argonaute 1.** Crystal structure of the Argonaute 1 protein in complex with a guide RNA (PDB: 4KXT, top) and AlphaFold 3 predictions for the complex of the same protein non-seed point mutant of hsa-miR-6869-3p with the maximum $\langle d_{\mathrm{Hamming\ mutant}} \rangle$ (bottom). RNA depicted as a ribbons with bases indicated and all residues in the predicted structure coloured by plDDT, a measure of the confidence of the prediction, as indicated by the colour bars. Most of the miRNA structure is predicted with high confidence ($70 < \mathrm{plDDT} < 90$, average: 74.2), and the predictions show base-pairing within the miRNA. The crystal structure does not contain information about the positions of nucleotides 10 to 18 of the guide RNA. Images created with UCSF ChimeraX [213].

Table S24: Maximum-confidence (plDDT) AlphaFold 3 predictions for the complex between human Argonaute 2 protein taken from the PDB structure 4F3T and the miRNAs highlighted by our analysis of secondary structure and the point mutants that have the greatest value of $\Delta p_{\text{unfolded}}$. plDDT values are averaged over all nucleotides.

ID	sequence	SS	average plDDT
WT hsa-miR-485-5p	`AGAGGCUGGCCGUGAUGAAUUC`	....	71.9
hsa-miR-485-5p mutant with maximum $\Delta p_{\text{unfolded}}$	`AGAGGCUAGCCGUGAUGAAUUC`	....	56.2
WT hsa-miR-1269b-3p	`CUGGACUGAGCCAUGCUACUGG`	....	82.7
hsa-miR-1269b-3p mutant with maximum $\Delta p_{\text{unfolded}}$	`AUGGACUGAGCCAUGCUACUGG`	....	82.0
WT hsa-miR-1908-3p-3p	`CCGGCCGCCGGCUCCGCCCCG`	....	73.9
hsa-miR-1908-3p-3p mutant with maximum $\Delta p_{\text{unfolded}}$	`ACGGCCGCCGGCUCCGCCCCG`	....	72.5
WT hsa-miR-4477b-3p	`AUUAAGGACAUUUGUGAUUGAU`	....	74.1
hsa-miR-4477b-3p mutant with maximum $\Delta p_{\text{unfolded}}$	`AUUAAGGAAAUUUGUGAUUGAU`	....	65.2
WT hsa-miR-4537-3p	`UGAGCCGAGCUGAGCUUAGCUG`	....	66.4
hsa-miR-4537-3p mutant with maximum $\Delta p_{\text{unfolded}}$	`AGAGCCGAGCUGAGCUUAGCUG`	..(((.((((...)))).))).	65.3
WT hsa-miR-4641-3p	`UGCCCAUGCCAUACUUUUGCCUCA`	....	74.9
hsa-miR-4641-3p mutant with maximum $\Delta p_{\text{unfolded}}$	`AGCCCAUGCCAUACUUUUGCCUCA`	....	74.1
WT hsa-miR-6821-3p-3p	`UGACCUCUCCGCUCCGCACAG`	....	85.9
hsa-miR-6821-3p mutant with maximum $\Delta p_{\text{unfolded}}$	`AGACCUCUCCGCUCCGCACAG`	....	85.2

12 Code and data

We provide the set of Python, Bash and MATLAB scripts that we have used to generate all the results in the manuscript. We also provide the results from our computational predictions of the properties of secondary structure for all studied point mutants and tertiary structure predictions for selected miRNAs in complex with Argonaute proteins. Code and data available at https://doi.org/10.6084/m9.figshare.26490985.v1.

References

[1] A. Bhattacharya and Y. Cui, *Nucleic Acids Research*, 2016, **44**, D1005–D1010.

[2] R. Lorenz, S. H. Bernhart, C. H. zu Siederdissen, H. Tafer, C. Flamm, P. F. Stadler and I. L. Hofacker, *Algorithms for Molecular Biology*, 2011, **6**, 26.

[3] Mathworks, *Receiver operating characteristic (ROC) curve or other performance curve for classifier output - MATLAB perfcurve - MathWorks United Kingdom*, 2022, `https://uk.mathworks.com/help/stats/perfcurve.html`.

[4] Mathworks, *Wilcoxon rank sum test - MATLAB ranksum - MathWorks United Kingdom*, 2022, `https://uk.mathworks.com/help/stats/ranksum.html`.

[5] J. A. Garcia-Martin and P. Clote, *PLoS ONE*, 2015, **10**, 1–32.

[6] R. Bai, Z. Cui, Y. Ma, Y. Wu, N. Wang, L. Huang, Q. Yao and J. Sun, *Molecular Carcinogenesis*, 2019, **58**, 2254–2265.

[7] Y. Xiang, H. Liu, H. Hu, L. W. Li, Q. B. Zong, T. W. Wu, X. Y. Li, S. Q. Fang, Y. W. Liu, Y. Zhan, H. Wang and Z. X. Lu, *Aging*, 2022, **14**, 4755–4768.

[8] A. F. Christopher, M. Gupta and P. Bansal, *Gene*, 2016, **594**, 30–40.

[9] M. Zou, F. Wang, R. Gao, J. Wu, Y. Ou, X. Chen, T. Wang, X. Zhou, W. Zhu, P. Li, L. W. Qi, T. Jiang, W. Wang, C. Li, J. Chen, Q. He and Y. Chen, *Scientific Reports*, 2016, **6**, 1–15.

[10] G. Caruso, L. Falzone, G. Palermo, D. Ricci, G. Mazza, M. Libra, S. Caruso and G. Gattuso, *The Journal of Sexual Medicine*, 2023, **20**, 935–944.

[11] N. Coban, A. F. Erkan, A. S. Ozuynuk-Ertugrul and B. Ekici, *Acta Cardiologica*, 2023, **0**, 1–12.

[12] S. R. Zaker and K. Ghaedi, *Cell Journal*, 2021, **23**, 414–420.

[13] Q. Zeng, F. Jin, H. Qian, H. Chen, Y. Wang, D. Zhang, Y. Wei, T. Chen, B. Guo and C. Chai, *Carcinogenesis*, 2022, **43**, 150–159.

[14] Y. Gu, W. Wang, X. Wang, H. Xie, X. Ye and P. Shu, *Scientific Reports*, 2019, **9**, 1–8.

[15] K. Liu, F. Xie, A. Gao, R. Zhang, L. Zhang, Z. Xiao, Q. Hu, W. Huang, Q. Huang, B. Lin, J. Zhu, H. Wang, J. Que and X. Lan, *Molecular Cancer*, 2017, **16**, 1–14.

[16] S. Laudato, N. Patil, M. L. Abba, J. H. Leupold, A. Benner, T. Gaiser, A. Marx and H. Allgayer, *International Journal of Cancer*, 2017, **141**, 1879–1890.

[17] G. Xu, J. Cai, L. Wang, L. Jiang, J. Huang, R. Hu and F. Ding, *Experimental Cell Research*, 2018, **362**, 268–278.

[18] Z. Ma, F. Chao, S. Wang, Z. Song, Z. Zhuo, J. Zhang, G. Xu and G. Chen, *Biochemical and Biophysical Research Communications*, 2020, **525**, 418–424.

[19] H. Dai, J. Wang, Z. Huang, H. Zhang, X. Wang, Q. Li and W. Feng, *Technology in Cancer Research and Treatment*, 2021, **20**, 1–11.

[20] L. Sabre, P. Maddison, G. Sadalage, P. A. Ambrose and A. R. Punga, *Journal of Neuroimmunology*, 2018, **321**, 164–170.

[21] R. Mishra, S. Bhattacharya, B. S. Rawat, A. Kumar, A. Kumar, K. Niraj, A. Chande, P. Gandhi, D. Khetan, A. Aggarwal, S. Sato, P. Tailor, A. Takaoka and H. Kumar, *iScience*, 2020, **23**, 101322.

[22] B.-S. Kim, J.-Y. Jung, J.-Y. Jeon, H.-A. Kim and C.-H. Suh, *HLA*, 2016, **88**, 187–193.

[23] J. Huang, C. Deng, T. Guo, X. Chen, P. Chen, S. Du and M. Lu, *Anti-Cancer Agents in Medicinal Chemistry*, 2023, **23**, 717–725.

[24] R. M. Busch, L. Yehia, P. Bazeley, M. Seyfi, I. Blümcke, B. P. Hermann, I. M. Najm and C. Eng, *Epilepsia*, 2020, **61**, 2203–2213.

[25] Y. Zhang, L. Geng, G. Talmon and J. Wang, *Journal of Biological Chemistry*, 2015, **290**, 6215–6225.

[26] C. Wang, B. Wang, W. Liang, C. Zhou, W. Lin, Z. Meng, W. Wu, M. Wu, Y. Liao, X. Li, J. Zhao and Y. He, *BMC Cancer*, 2022, **22**, 1–11.

[27] G. Tai, M. Zhang and F. Liu, *Experimental Lung Research*, 2021, **47**, 136–148.

[28] W. Usuba, F. Urabe, Y. Yamamoto, J. Matsuzaki, H. Sasaki, M. Ichikawa, S. Takizawa, Y. Aoki, S. Niida, K. Kato, S. Egawa, T. Chikaraishi, H. Fujimoto and T. Ochiya, *Cancer Science*, 2019, **110**, 408–419.

[29] Y. Zhang, L. Cheng, Y. Chen, G. Y. Yang, J. Liu and L. Zeng, *Journal of Affective Disorders*, 2016, **193**, 51–58.

[30] F. Ren, W. C. Gao, Z. P. Ke, Y. Xu and Y. Liu, *Journal of Cellular Biochemistry*, 2019, **120**, 1932–1942.

[31] H. Cao, A. Baranova, W. Yue, H. Yu, Z. Zhu, F. Zhang and D. Liu, *Frontiers in Genetics*, 2020, **11**, 1–8.

[32] X. Xu and S. Zheng, *Cancer Management and Research*, 2020, **12**, 6137–6147.

[33] H. Ravishankar, A. S. Mangani, G. L. P. Moses, S. P. Mani, S. Parameswaran, V. Khetan, S. Ganesan and S. Krishnakumar, *Experimental Eye Research*, 2020, **199**, 108184.

[34] E. Findeiss, S. C. Schwarz, V. Evsyukov, T. W. Rösler, M. Höllerhage, T. Chakroun, N.-P. Nykänen, Y. Shen, W. Wurst, M. Kohl, J. Tost and G. U. Höglinger, *Frontiers in Cell and Developmental Biology*, 2021, **9**, 561086.

[35] R. Krattinger, A. Boström, H. B. Schiöth, W. E. Thasler, J. Mwinyi and G. A. Kullak-Ublick, *American Journal of Physiology - Gastrointestinal and Liver Physiology*, 2016, **310**, G1044–G1051.

[36] G. Zhou, X. Zhang, W. Wang, W. Zhang, H. Wang and G. Xin, *Medical Science Monitor*, 2019, **25**, 1903–1916.

[37] R. Mysore, Y. Zhou, S. Sädevirta, H. Savolainen-Peltonen, P. A. N. Haridas, J. Soronen, M. Leivonen, A. P. Sarin, P. Fischer-Posovszky, M. Wabitsch, H. Yki-Järvinen and V. M. Olkkonen, *Biochimica et Biophysica Acta - Molecular and Cell Biology of Lipids*, 2016, **1861**, 342–351.

[38] Z. Wang, K. K. Miu, X. Zhang, A. T. Y. Wan, G. Lu, H. H. Cheung, H. M. Lee, A. P. S. Kong, J. C. N. Chan and W. Y. Chan, *JHEP Reports*, 2020, **2**, 100179.

[39] J. Fang, J. Huang and X. Zhang, *Applied Biological Chemistry*, 2022, **65**, 46.

[40] T. Yang, S. Li, J. Liu, D. Yin, X. Yang, Q. Tang and S. Wang, *American Journal of Translational Research*, 2020, **12**, 2939–2955.

[41] P. L. Wander, D. A. Enquobahrie, T. K. Bammler, J. W. MacDonald, S. Srinouanprachanh, T. Kaleru, D. Khakpour and S. Trikudanathan, *Molecular and Cellular Endocrinology*, 2022, **554**, 111723.

[42] Z. lei Zeng, J. huan Lu, Y. Wang, H. Sheng, Y. nan Wang, Z. hong Chen, Q. nian Wu, J. B. Zheng, Y. xing Chen, D. dong Yang, K. Yu, H. yu Mo, J. jia Hu, P. shan Hu, Z. xian Liu, H. qiang Ju and R. H. Xu, *Cancer Medicine*, 2021, **10**, 2423–2441.

[43] X. guo Zhou, X. liang Huang, S. yuan Liang, S. mei Tang, S. kao Wu, T. tong Huang, Z. nan Mo and Q. yan Wang, *OncoTargets and Therapy*, 2018, **Volume 11**, 2815–2830.

[44] G. X. Guo, Q. Y. Li, W. L. Ma, Z. H. Shi and X. Q. Ren, *International Journal of Clinical and Experimental Pathology*, 2015, **8**, 12292–12299.

[45] X. J. Lin, C. L. He, T. Sun, X. J. Duan, Y. Sun and S. J. Xiong, *International Journal of Molecular Medicine*, 2017, **40**, 83–89.

[46] R. sheng Huang, Y. liang Zheng, C. Li, C. Ding, C. Xu and J. Zhao, *Life Sciences*, 2018, **199**, 104–111.

[47] X. X. Hu, X. N. Xu, B. S. He, H. L. Sun, T. Xu, X. X. Liu, X. X. Chen, K. X. Zeng, S. K. Wang and Y. Q. Pan, *Journal of Cancer*, 2018, **9**, 2603–2611.

[48] P. Jiang, C. Xu, L. Chen, A. Chen, X. Wu, M. Zhou, I. U. Haq, Z. Mariyam and Q. Feng, *Journal of Cellular Biochemistry*, 2018, **119**, 8623–8635.

[49] M. Wang, W. R. Cai, R. Meng, J. R. Chi, Y. R. Li, A. X. Chen, Y. Yu and X. C. Cao, *Biochemical and Biophysical Research Communications*, 2018, **501**, 48–54.

[50] D. L. Han, L. L. Wang, G. F. Zhang, W. F. Yang, J. Chai, H. M. Lin, Z. Fu and J. M. Yu, *European Review for Medical and Pharmacological Sciences*, 2019, **23**, 2809–2816.

[51] X. Wang, X. Zhou, F. Zeng, X. Wu and H. Li, *Breast Cancer*, 2020, **27**, 765–775.

[52] D. Wang, X. Zhou, J. Yin and Y. Zhou, *Open Life Sciences*, 2020, **15**, 488–500.

[53] L. Cheng, R. Peng, P. Guo, H. Zhang, D. Liu, X. Liao, Y. Liu, X. Mo and Y. Liao, *Experimental Cell Research*, 2021, **402**, 112547.

[54] Y. Rahmati, M. Alivand and H. Mollanoori, *Computational Biology and Chemistry*, 2021, **92**, 107458.

[55] Y. Chen, L. Wu and M. Bao, *Molecular Biotechnology*, 2022.

[56] J. Chen, S. Wu, J. Wang, Y. Sha and Y. Ji, *Reproductive Sciences*, 2022, **29**, 2236–2250.

[57] X. Chen, S. Zhang, P. Shi, Y. Su, D. Zhang and N. Li, *Current Neurovascular Research*, 2020, **17**, 259–266.

[58] C. Shen, Z. Wu, Y. Wang, S. Gao, L. Da, L. Xie, Y. Qie, D. Tian and H. Hu, *Cancer Medicine*, 2020, **9**, 3885–3903.

[59] S. Park, E. Cheon and H. Kim, *Osteoarthritis and Cartilage*, 2013, **21**, 981–989.

[60] X. Xu, H. Yuan, J. Pan, W. Chen, C. Chen, Y. Li and F. Li, *BMC Cancer*, 2022, **22**, 1–12.

[61] E. Raitoharju, I. Seppälä, N. Oksala, L. P. Lyytikäinen, O. Raitakari, J. Viikari, M. Ala-Korpela, P. Soininen, A. J. Kangas, M. Waldenberger, N. Klopp, T. Illig, J. Leiviskä, B. M. Loo, N. Hutri-Kähönen, M. Kähönen, R. Laaksonen and T. Lehtimäki, *Molecular and Cellular Endocrinology*, 2014, **391**, 41–49.

[62] X. Yu, H. Song, T. Xia, S. Han, B. Xiao, L. Luo, Y. Xi and J. Guo, *Gene*, 2013, **532**, 87–93.

[63] C. Eyileten, Z. Wicik, D. Keshwani, F. Aziz, F. Aberer, P. N. Pferschy, N. J. Tripolt, C. Sourij, B. Prietl, F. Prüller, D. von Lewinski, S. D. Rosa, J. M. Siller-Matula, M. Postula and H. Sourij, *Cardiovascular Diabetology*, 2022, **21**, 1–12.

[64] A. Gholaminejad, A. Roointan and Y. Gheisari, *BMC Immunology*, 2021, **22**, 1–17.

[65] Y. Sun, X. Wang, Z. Wang, Y. Zhang, N. Che, X. Luo, Z. Tan, X. Sun, X. Li, K. Yang, G. Wang, L. Luan, Y. Liu, X. Zheng, M. Wei, H. Cheng and J. Yin, *Epilepsy Research*, 2016, **127**, 276–283.

[66] C. Zhou, X. Zhao and S. Duan, *Journal of Cellular Physiology*, 2021, **236**, 15–26.

[67] M. Scheper, A. Iyer, J. J. Anink, L. Mesarosova, J. D. Mills and E. Aronica, *Neuropathology and Applied Neurobiology*, 2023, **49**, 1–13.

[68] R. N. Quiroz, A. L. Scott, E. A. Philot, L. Atencio, C. F. Ponce, G. A. Martinez, A. C. Bonfanti, L. G. Escorcia and E. N. Quiroz, *bioRxiv*, 2020.

[69] T. T. N. Nguyen, T. H. N. Nguyen, L. H. Huynh, H. N. Phan and H. T. Nguyen, *Recent Advances in Noncoding RNAs*, 2022, 1–18.

[70] L. Zhong, Y. Wang, Y. Cheng, W. Wang, B. Lu, L. Zhu and Y. Ma, *Biochemical and Biophysical Research Communications*, 2018, **499**, 1044–1049.

[71] K. Tu, Z. Liu, B. Yao, S. Han and W. Yang, *International Journal of Oncology*, 2016, **48**, 965–974.

[72] A. V. Timofeeva, V. A. Gusar, N. E. Kan, K. N. Prozorovskaya, A. O. Karapetyan, O. R. Bayev, V. V. Chagovets, S. F. Kliver, D. Y. Iakovishina, V. E. Frankevich and G. T. Sukhikh, *Placenta*, 2018, **61**, 61–71.

[73] E. Tolosa, T. Botta-Orfila, X. Morató, C. Calatayud, R. Ferrer-Lorente, M. J. Martí, M. Fernández, C. Gaig, Ángel Raya, A. Consiglio, M. Ezquerra and R. Fernández-Santiago, *Neurobiology of Aging*, 2018, **69**, 283–291.

[74] J. Liu, S. Yan, J. Hu, D. Ding, Y. Liu, X. Li, H. S. Pan, G. Liu, B. Wu and Y. Liu, *Bioengineered*, 2021, **12**, 8457–8467.

[75] A. Du, S. Zhao, L. Wan, T. Liu, Z. Peng, Z. Zhou, Z. Liao and H. Fang, *Journal of Cellular and Molecular Medicine*, 2016, **20**, 1329–1338.

[76] A. Romero-Ruiz, B. Pineda, D. Ovelleiro, C. Perdices-Lopez, E. Torres, M. J. Vazquez, I. Guler, Álvaro Jiménez, R. Pineda, M. Persano, C. Romero-Baldonado, J. E. Arjona, J. Lorente, C. Muñoz, E. Paz, F.-I. Garcia-Maceira, Álvaro Arjona-Sánchez and M. Tena-Sempere, *European Journal of Endocrinology*, 2021, **185**, 637–652.

[77] E. C. Willner, H. L. Galan, B. F. Cuneo, H. A. Hoffman, B. Neltner, E. L. Schuchardt, A. Karimpour-Fard, S. D. Miyamoto and C. C. Sucharov, *American Journal of Obstetrics and Gynecology*, 2021, **225**, 439.e1–439.e10.

[78] K. D. Silva, R. T. Demmer, D. Jönsson, A. Mousa, A. Forbes and J. Enticott, *Heliyon*, 2022, **8**, e08886.

[79] D. Anatolou, N. Dovrolis, G. Ragia, G. Kolios and V. G. Manolopoulos, *OMICS: A Journal of Integrative Biology*, 2022, **26**, 608–621.

[80] J. M. Modak, M. Roy-O'Reilly, L. Zhu, I. Staff and L. D. McCullough, *Journal of Stroke and Cerebrovascular Diseases*, 2019, **28**, 121–124.

[81] S. Zhou, Q. Meng, L. Li, L. Hai, Z. Wang, Z. Li and Y. Sun, *Frontiers in Genetics*, 2021, **12**, 1–9.

[82] D. Poudyal, A. Herman, J. W. Adelsberger, J. Yang, X. Hu, Q. Chen, M. Bosche, B. T. Sherman and T. Imamichi, *Scientific Reports*, 2018, **8**, 1–13.

[83] X. Wang, J. Gao, B. Zhou, J. Xie, G. Zhou and Y. Chen, *Life Sciences*, 2019, **232**, 116596.

[84] W. Yang, W. Xiao, Z. Cai, S. Jin and T. Li, *OncoTargets and Therapy*, 2020, **13**, 109–118.

[85] Z. Xie, C. Zhong and S. Duan, *Frontiers in Cell and Developmental Biology*, 2022, **10**, 1–11.

[86] C. Jia, Z. Yao, Z. Lin, L. Zhao, X. Cai, S. Chen, M. Deng and Q. Zhang, *Journal of Cellular Physiology*, 2021, **236**, 1252–1269.

[87] G. C. Genc, A. Dursun, S. K. Celik, M. Calik, F. Kokturk and I. E. Piskin, *Gene*, 2018, **678**, 73–78.

[88] A. Mariam, G. Miller-Atkins, A. Moro, A. I. Rodarte, S. Siddiqi, L.-A. Acevedo-Moreno, J. M. Brown, D. S. Allende, F. Aucejo and D. M. Rotroff, *PeerJ*, 2022, **10**, e12715.

[89] J. B. de Carvalho, G. L. de Morais, T. C. dos Santos Vieira, N. C. Rabelo, J. C. Llerena, S. M. de Carvalho Gonzalez and A. T. R. de Vasconcelos, *Frontiers in Genetics*, 2019, **10**, 1–11.

[90] C. Medina-Trillo, J.-D. Aroca-Aguilar, J.-J. Ferre-Fernández, C.-D. Méndez-Hernández, L. Morales, J. García-Feijoo and J. Escribano, *MicroRNA*, 2015, **4**, 50–56.

[91] Y. Wang, L. Zou, T. Wu, L. Xiong, T. Zhang, L. Kong, Y. Xue and M. Tang, *Ecotoxicology and Environmental Safety*, 2019, **169**, 863–873.

[92] Y. Yan, D. Song, X. Zhang, G. Hui and J. Wang, *Frontiers in Pharmacology*, 2020, **11**, 1–7.

[93] E. Budd, G. Nalesso and A. Mobasheri, *Expert Review of Molecular Diagnostics*, 2018, **18**, 55–74.

[94] B. Gao, Q. Shao, H. Choudhry, V. Marcus, K. Dong, J. Ragoussis and Z. H. Gao, *International Journal of Oncology*, 2016, **49**, 1108–1118.

[95] S. Nomiri, R. Hoshyar, E. Chamani, Z. Rezaei, F. Salmani, P. Larki, T. Tavakoli, F. gholipour, N. J. Tabrizi, A. Derakhshani, M. Santarpia, T. Franchina, O. Brunetti, N. Silvestris and H. Safarpour, *Biomedicine and Pharmacotherapy*, 2022, **147**, 112691.

[96] S. Orsten, I. Baysal, S. Yabanoglu-Ciftci, T. Ciftci, A. Azizova, D. Akinci, Y. Akyon and O. Akhan, *Journal of Helminthology*, 2021, **95**, e1.

[97] P. C. Sanchez-Diaz, T.-H. Hsiao, J. C. Chang, D. Yue, M. C. Tan, H.-I. H. Chen, G. E. Tomlinson, Y. Huang, Y. Chen and J. Y. Hung, *PLoS ONE*, 2013, **8**, e61622.

[98] B. Chen, S. She, D. Li, Z. Liu, X. Yang, Z. Zeng and F. Liu, *Scandinavian Journal of Gastroenterology*, 2013, **48**, 815–824.

[99] K. Kochan-Jamrozy, J. Króliczewski, A. Moszyńska, J. F. Collawn and R. Bartoszewski, *Cellular Signalling*, 2019, **54**, 150–160.

[100] P. Yan, P. Pang, X. Hu, A. Wang, H. Zhang, Y. Ma, K. Zhang, Y. Ye, B. Zhou and J. Mao, *Journal of Cancer*, 2021, **12**, 1–9.

[101] M. Chen, X. Wang, H. Wang, M. Zhang, L. Chen and H. Chen, *ResearchSquare Pre-print*, 2021, 1–12.

[102] F. Ni, Z. Gui, Q. Guo, Z. Hu, X. Wang, D. Chen and S. Wang, *Oncology Letters*, 2016, **11**, 1155–1160.

[103] B. M. Ryan, A. C. McClary, N. Valeri, D. Robinson, A. Paone, E. D. Bowman, A. I. Robles, C. Croce and C. C. Harris, *PLoS ONE*, 2012, **7**, 3–8.

[104] N. Othman, L. L. In, J. A. Harikrishna and N. Hasima, *PLoS ONE*, 2013, **8**, 1–14.

[105] J. Zheng, J. Deng, M. Xiao, L. Yang, L. Zhang, Y. You, M. Hu, N. Li, H. Wu, W. Li, J. Lu and Y. Zhou, *Cancer Research*, 2013, **73**, 5151–5162.

[106] F. Qiu, L. Yang, L. Zhang, X. Yang, R. Yang, W. Fang, D. Wu, J. Chen, C. Xie, D. Huang, Y. Zhou and J. Lu, *Gene*, 2015, **565**, 180–186.

[107] T. Xu, H. Q. Xie, Y. Li, Y. Xia, Y. Chen, L. Xu, L. Wang and B. Zhao, *Scientific Reports*, 2017, **7**, 1–10.

[108] H. Yan, S. Xin, J. Ma, H. Wang, H. Zhang and J. Liu, *Journal of Cellular Biochemistry*, 2019, **120**, 8723–8730.

[109] N. Zhang, Y. Li, Y. Zheng, L. Zhang, Y. Pan, J. Yu and M. Yang, *Laboratory Investigation*, 2019, **99**, 568–576.

[110] W. Gu, D. Wen, H. Lu, A. Zhang, H. Wang, J. Du, L. Zeng and J. Jiang, *Journal of Clinical Immunology*, 2020, **40**, 147–157.

[111] Y. Abrahams, M.-J. Laguette, S. Prince and M. Collins, *Annals of Human Genetics*, 2013, **77**, 204–214.

[112] B. Cheng, J. yi Li, X. chao Li, X. fang Wang, Z. jing Wang, J. Liu and A. ping Deng, *Scientific Reports*, 2018, **8**, 1–9.

[113] C. Sun, M. Liu, W. An, J. Liu, F. Yang, F. Wang, J. Jiang, Q. Zhou, Y. Jia, Y. Wang, J. Yuan, L. Ma, X. Sun, L. Wang, Z. Liao and Z. Li, *The Journal of Gene Medicine*, 2022, 1–10.

[114] X. He and Y. Feng, *Anti-Cancer Drugs*, 2022, **33**, 478–488.

[115] Y. Wang, M. Chen, Z. Tao, Q. Hua, S. Chen and B. Xiao, *Cancer Genetics*, 2013, **206**, 340–346.

[116] S. K. Saini, P. Kalaiarasan, R. K. Singh, S. Manvati and R. N. Bamezai, *Mitochondrion*, 2018, **43**, 30–36.

[117] S. Kong, Y. Cao, X. Li, Z. Li, Y. Xin and Y. Meng, *Journal of Cellular and Molecular Medicine*, 2020, **24**, 4677–4686.

[118] D. Liu, L. Zhong, Z. Yuan, J. Yao, P. Zhong, J. Liu, S. Yao, Y. Zhao, L. Liu, M. Chen, L. Li and B. Liu, *Cellular Signalling*, 2019, **54**, 1–9.

[119] J. Du, F. Bai, P. Zhao, X. Li, X. Li, L. Gao, C. Ma and X. Liang, *Biochimica et Biophysica Acta - Molecular Cell Research*, 2018, **1865**, 1–11.

[120] Z. Derakhshan, G. Khamisipour, F. H. Soleimani and N. Motamed, *Gene Reports*, 2022, **27**, 101582.

[121] X. Nie, H. Liu, X. Wei, L. Li, L. Lan, L. Fan, H. Ma, L. Liu, Y. Zhou, R. Hou and W.-D. Chen, *Cancer Management and Research*, 2021, **Volume 13**, 8025–8035.

[122] R. Fang, Y. Zhu, L. Hu, V. S. Khadka, J. Ai, H. Zou, D. Ju, B. Jiang, Y. Deng and X. Hu, *Frontiers in Physiology*, 2019, **10**, 1–12.

[123] Y. W. Hu, J. Y. Zhao, S. F. Li, J. L. Huang, Y. R. Qiu, X. Ma, S. G. Wu, Z. P. Chen, Y. R. Hu, J. Y. Yang, Y. C. Wang, J. J. Gao, Y. H. Sha, L. Zheng and Q. Wang, *Arteriosclerosis, Thrombosis, and Vascular Biology*, 2015, **35**, 87–101.

[124] G. Wojciechowska, L. Szczerbinski, M. Kretowski, M. Niemira, H. R. Hady and A. Kretowski, *Obesity*, 2022, **30**, 435–446.

[125] J. J. Zhao, J. Yang, J. Lin, N. Yao, Y. Zhu, J. Zheng, J. Xu, J. Q. Cheng, J. Y. Lin and X. Ma, *Child's Nervous System*, 2009, **25**, 13–20.

[126] J. von Frowein, P. Pagel, R. Kappler, D. von Schweinitz, A. Roscher and I. Schmid, *Hepatology*, 2011, **53**, 833–842.

[127] X. Song, Y. Xie, Y. Liu, M. Shao and W. Yang, *International Journal of Molecular Medicine*, 2017, **40**, 891–897.

[128] E. Taghizadeh, F. Taheri, D. Rostami, P. G. Renani, G. A. Ferns, A. Pasdar and M. G. Mobarhan, *Current Cancer Therapy Reviews*, 2020, **16**, 269–275.

[129] K. Wang, H. Lü, H. Qu, Q. Xie, T. Sun, O. Gan and B. Hu, *OncoTargets and Therapy*, 2019, **12**, 11453–11464.

[130] S. Zhao, L. Tang, W. Chen, J. Su, F. Li, X. Chen and L. Wu, *Naunyn-Schmiedeberg's Archives of Pharmacology*, 2021, **394**, 797–807.

[131] N. N. A. Deen, N. A. Lanman, S. Chittiboyina, S. Fostok, R. Nasr, S. Lelièvre and R. Talhouk, *Scientific reports*, 2022, **12**, 21974.

[132] D. S. Jairajpuri, Z. H. Malalla, N. Mahmood and W. Y. Almawi, *Gene*, 2017, **627**, 543–548.

[133] R. Shams, S. Saberi, M. Zali, A. Sadeghi, S. Ghafouri-Fard and H. A. Aghdaei, *Scientific Reports*, 2020, **10**, 1–15.

[134] K. A. Gaither, C. J. Watson, B. Madarampalli and P. Lazarus, *PLoS ONE*, 2020, **15**, 1–19.

[135] J. D. Galley, P. Mar, Y. Wang, R. Han, A. Rajab and G. E. Besner, *Journal of Pediatric Surgery*, 2021, **56**, 1966–1975.

[136] L. Hongdan and L. Feng, *Biochemical and Biophysical Research Communications*, 2018, **496**, 302–308.

[137] H. Li, Q. Zhao and Z. Tang, *Pathology Research and Practice*, 2021, **226**, 153566.

[138] K. J. Capistrano, J. Richner, J. Schwartz, S. K. Mukherjee, D. Shukla and A. R. Naqvi, *Biochimica et Biophysica Acta - Molecular Basis of Disease*, 2023, **1869**, 166612.

[139] A. Fernández-Pato, A. Virseda-Berdices, S. Resino, P. Ryan, O. Martínez-González, F. Pérez-García, M. Martin-Vicente, D. Valle-Millares, O. Brochado-Kith, R. Blancas, A. Martínez, F. C. Ceballos, S. Bartolome-Sánchez, E. J. Vidal-Alcántara, D. Alonso, N. Blanca-López, I. R. Martinez-Acitores, L. Martin-Pedraza, M. Ángeles Jiménez-Sousa and A. Fernández-Rodríguez, *Emerging Microbes and Infections*, 2022, **11**, 676–688.

[140] E. Chang, G. Fishbein, M. Bakir, G. Bondar, N. Jackson, D. Liem, S. Litovsky, J. Tallaj, C. Starling, P. Ping, E. Reed, M. Deng, E. Tabak and M. Cadeiras, *Circulation*, 2015, **132**,.

[141] J. M. Moreno, M. J. Núñez, A. Quiñonero, S. Martínez, M. D. L. Orden, C. Simón, A. Pellicer, C. Díaz-García and F. Domínguez, *Fertility and Sterility*, 2015, **104**, 1037–1046.e1.

[142] X. Zhuang, Z. Li, H. Lin, L. Gu, Q. Lin, Z. Lu and C. M. Tzeng, *Scientific Reports*, 2015, **5**, 1–9.

[143] Y. Liang, G. Zhao, L. Tang, J. Zhang, T. Li and Z. Liu, *Experimental Cell Research*, 2016, **347**, 312–321.

[144] L. Ou, W. Huang, T. Zhang, D. Xu, D. Kong and Y. Meng, *Transplant Immunology*, 2023, **77**, 101747.

[145] A. K. Singh, S. B. Rooge, A. Varshney, M. Vasudevan, A. Bhardwaj, S. K. Venugopal, N. Trehanpati, M. Kumar, R. Geffers, V. Kumar and S. K. Sarin, *Hepatology*, 2018, **67**, 1695–1709.

[146] J. Cui, X. Kang, Y. Shan, M. Zhang, Y. Gao, W. Wu and L. Chen, *Scientific Reports*, 2022, **12**, 1–10.

[147] N. Coban, A. S. Ozuynuk, A. F. Erkan, F. Guclu-Geyik and B. Ekici, *Molecular Biology Reports*, 2021, **48**, 7719–7732.

[148] X. He, G. Cheng, F. Xiao, L. Zhang, G. Jin, X. Zhao, Y. Liu, J. Liang, Y. Li, Z. Liu, Q. Yuan, H. Ren, Q. Wu, J. Wu, L. Xue, J. Feng, Z. Wang, Y. Xing, W. Wu, Z. Li, D. Wei and X. Song, *Journal of Gastrointestinal Oncology*, 2021, **12**, 69–78.

[149] H. Yang, Q. Li, Y. Wu, J. Dong, Y. Lao, Z. Ding, C. Xiao, J. Fu and S. Bai, *Oncology Reports*, 2020, **44**, 2045–2055.

[150] T. Nishimura, E. Tamizu, S. Uno, Y. Uwamino, H. Fujiwara, K. Nishio, Y. Nakano, H. Shiono, H. Namkoong, Y. Hoshino, S. Iwata and N. Hasegawa, *Journal of Infection and Chemotherapy*, 2017, **23**, 703–708.

[151] P. Muti, S. Donzelli, A. Sacconi, A. Hossain, F. Ganci, T. Frixa, S. Sieri, V. Krogh, F. Berrino, F. Biagioni, S. Strano, J. Beyene, Y. Yarden and G. Blandino, *Carcinogenesis*, 2018, **39**, 98–108.

[152] L. Falzone, G. Romano, R. Salemi, C. Bucolo, B. Tomasello, G. Lupo, C. Anfuso, D. Spandidos, M. Libra and S. Candido, *Molecular Medicine Reports*, 2019, **19**, 2599–2610.

[153] Q. Zhou, H. Li, Y. Zhang, W. Peng, H. Hou, M. Gu, F. Zhang, X. Wang, X. Gu and L. Li, *BMC Pregnancy and Childbirth*, 2021, **21**, 837.

[154] J. Ma, S. Shang, J. Wang, T. Zhang, F. Nie, X. Song, H. Zhao, C. Zhu, R. Zhang and D. Hao, *Psychiatry Research*, 2018, **265**, 70–76.

[155] G. N. López-Sánchez, E. Montalvo-Javé, M. Domínguez-Perez, B. Antuna-Puente, F. O. Beltrán-Anaya, A. Hidalgo-Miranda, N. C. Chávez-Tapia, M. Uribe and N. Nuño-Lámbarri, *Annals of Hepatology*, 2022, **27**, 100756.

[156] P. Kuang, P. Chen, L. Wang, W. Li, B. Chen, Y. Liu, Y. Xu, H. Wang, S. Zhao, L. Ye, F. Yu, H. Ji and Y. He, *Annals of Translational Medicine*, 2020, **8**, 121–121.

[157] M. Tanic, K. Yanowski, G. Gómez-López, M. S. Rodriguez-Pinilla, I. Marquez-Rodas, A. Osorio, D. G. Pisano, B. Martinez-Delgado and J. Benítez, *International Journal of Cancer*, 2015, **136**, 593–602.

[158] S. I. Jang, M. Tandon, L. Teos, C. Y. Zheng, B. M. Warner and I. Alevizos, *EBioMedicine*, 2019, **48**, 526–538.

[159] H. Hidaka, N. Seki, H. Yoshino, T. Yamasaki, Y. Yamada, N. Nohata, M. Fuse, M. Nakagawa and H. Enokida, *Oncotarget*, 2012, **3**, 44–57.

[160] A. Oroujalian, M. Peymani and K. Ghaedi, *Nucleosides, Nucleotides and Nucleic Acids*, 2021, **40**, 779–789.

[161] J. Guo, X. Fang, J. Zhou, L. Zeng and B. Yu, *Medicine (United States)*, 2022, **101**, E29705.

[162] A. Keller, P. Leidinger, J. Lange, A. Borries, H. Schroers, M. Scheffler, H.-P. Lenhof, K. Ruprecht and E. Meese, *PLoS ONE*, 2009, **4**, e7440.

[163] F. Finocchi, M. Pelloni, G. Balercia, F. Pallotti, A. F. Radicioni, A. Lenzi, F. Lombardo and D. Paoli, *Molecular Biology Reports*, 2020, **47**, 4373–4382.

[164] X. Jiang, M. Jiang, M. Xu, J. Xu and Y. Li, *Pathology - Research and Practice*, 2019, **215**, 900–904.

[165] Y. hao Wang, Y. wang Chen, W. li Xiao, X. lian Li, L. Feng, Y. lin Liu and X. xia Duan, *Current Medical Science*, 2022, **42**, 871–884.

[166] L. Meng, S. Chang, Y. Sang, P. Ding, L. Wang, X. Nan, R. Xu, F. Liu, L. Gu, Y. Zheng, Z. Li and M. Sang, *Breast Cancer Research*, 2022, **24**, 1–13.

[167] U. Heilmeier, M. Hackl, S. Skalicky, S. Weilner, F. Schroeder, K. Vierlinger, J. M. Patsch, T. Baum, E. Oberbauer, I. Lobach, A. J. Burghardt, A. V. Schwartz, J. Grillari and T. M. Link, *Journal of Bone and Mineral Research*, 2016, **31**, 2173–2192.

[168] H. Jia, H. Wang, F. Xia, Y. Sun, H. Liu, L. Yan, S. Li, D. Jiang and M. Xu, *DNA and Cell Biology*, 2020, **39**, 2257–2264.

[169] C. Tang, H. Wang, H. Wu, S. Yan, Z. Han, Z. Jiang, M. Na, M. Guo, D. Lu and Z. Lin, *Cellular and Molecular Neurobiology*, 2019, **39**, 461–470.

[170] J. Li, X. Yang, H. Guan, A. Mizokami, E. T. Keller, X. Xu, X. Liu, J. Tan, L. Hu, Y. Lu and J. Zhang, *International Journal of Oncology*, 2016, **49**, 838–846.

[171] Y. Hao, C. Lu, B. Zhang, Z. Xu, H. Guo and G. Zhang, *Clinical Interventions in Aging*, 2021, **16**, 187–202.

[172] L. Qi, C. Gao, F. Feng, T. Zhang, Y. Yao, X. Wang, C. Liu, J. Li, J. Li and C. Sun, *Journal of Cellular Biochemistry*, 2019, **120**, 18956–18966.

[173] Y. Qu, X. Huang, Z. Li, J. Liu, J. Wu, D. Chen, F. Zhao and D. Mu, *American Journal of Pathology*, 2014, **184**, 1541–1549.

[174] B. F. Chen, S. Gu, Y. K. Suen, L. Li and W. Y. Chan, *Epigenetics*, 2014, **9**, 119–128.

[175] L. Li, Y. P. Mou, Y. Y. Wang, H. J. Wang and X. Z. Mou, *Pathology Research and Practice*, 2019, **215**, 152511.

[176] R. Zhu, K. Nasu, N. Hijiya, M. Yoshihashi, T. Hirakawa, Y. Aoyagi and H. Narahara, *Reproductive Sciences*, 2021, **28**, 3498–3507.

[177] P. Bardin, E. Marchal-Duval, F. Sonneville, S. Blouquit-Laye, N. Rousselet, P. L. Rouzic, H. Corvol and O. Tabary, *Journal of Pathology*, 2018, **245**, 410–420.

[178] A. Shang, C. Zhou, G. Bian, W. Chen, W. Lu, W. Wang and D. Li, *Journal of Cellular Biochemistry*, 2019, **120**, 778–789.

[179] Y. Z. Yu, Q. Mu, Q. Ren, L. J. Xie, Q. T. Wang and C. P. Wang, *World Journal of Surgical Oncology*, 2021, **19**, 1–11.

[180] L. Bi, C. Zhang, Y. Yao and Z. He, *Journal of Biosciences*, 2021, **46**, 20.

[181] Z. Liu, Y. Yu, Z. Huang, Y. Kong, X. Hu, W. Xiao, J. Quan and X. Fan, *Cell Death & Disease*, 2019, **10**, 900.

[182] V. N. Aushev, D. D. Esposti, E. Lee, H. Vargas, Z. Herceg, J. Zhu and J. Chen, *Cancer Research*, 2016, **76**, 1896–1896.

[183] Z. Yang, S. Lu, Y. Wang, H. Tang, B. Wang, X. Sun, J. Qu and B. Rao, *International Journal of General Medicine*, 2022, **15**, 555–565.

[184] M. Asakage, Y. Usui, N. Nezu, H. Shimizu, K. Tsubota, N. Yamakawa, M. Takanashi, M. Kuroda and H. Goto, *Investigative Opthalmology & Visual Science*, 2020, **61**, 4.

[185] D. F. Pellatt, J. R. Stevens, R. K. Wolff, L. E. Mullany, J. S. Herrick, W. Samowitz and M. L. Slattery, *Clinical and Translational Gastroenterology*, 2016, **7**, e152.

[186] L. Liu, S. Han, X. Xiao, X. An, J. Gladkich, U. Hinz, S. Hillmer, T. Hoppe-Tichy, Y. Xu, M. Schaefer, O. Strobel and I. Herr, *Cell Death & Disease*, 2022, **13**, 1052.

[187] C. Gungormez, H. G. Aktas, N. Dilsiz and E. Borazan, *Molecular Biology Reports*, 2019, **46**, 4175–4183.

[188] Z. H. Huang, H. Wang, D. M. Wang, X. Y. Zhao, W. W. Liu, X. Zhong, D. M. He, B. R. Mu and M. H. Lu, *Journal of Cellular and Molecular Medicine*, 2022, **26**, 5779–5793.

[189] Y. Ma, X. Cong, Y. Zhang, X. Yin, Z. Zhu and Y. Xue, *Cancer Cell International*, 2020, **20**, 1–12.

[190] L. Zhang, F. Liu, Z. Meng, Q. Luo, D. Pan and Y. Qian, *Genomics*, 2021, **113**, 3512–3522.

[191] K. Wang, J. Jin, T. Ma and H. Zhai, *Biomedicine and Pharmacotherapy*, 2017, **91**, 517–525.

[192] M. J. Hsieh, C. W. Lin, S. C. Su, R. J. Reiter, A. W. G. Chen, M. K. Chen and S. F. Yang, *Molecular Therapy - Nucleic Acids*, 2020, **19**, 877–889.

[193] W. long Liang, J. Cao, B. Xu, P. Yang, F. Shen, Z. Sun, W. lin Li, Q. Wang and F. Liu, *Biomedicine & Pharmacotherapy*, 2015, **72**, 119–124.

[194] S. Li, Y. Liu, G. Qiu, Y. Luo, L. Luan, T. Xu, Y. Wang and S. Xia, *Cancer Management and Research*, 2021, **Volume 13**, 1967–1979.

[195] J. Dong, J. Wang, C. Shan, H. Zhang and O. Xu, *Experimental Biology and Medicine*, 2020, **245**, 1222–1232.

[196] Y. Shang, Z. Zhang, Z. Liu, B. Feng, G. Ren, K. Li, L. Zhou, Y. Sun, M. Li, J. Zhou, Y. An, K. Wu, Y. Nie and D. Fan, *Oncogene*, 2014, **33**, 3267–3276.

[197] Q. Liu, H. Li, N. Wang, H. Chen and J. Wang, *International Journal of Cardiology*, 2013, **168**, 2082–2088.

[198] B. Yang, X. Huang, S. Xu, L. Li, W. Wu, Y. Dai, M. X. Ge, L. Yuan, W. Cao, M. Yang, Y. Wu and D. Deng, *Frontiers in Immunology*, 2021, **12**, 1–14.

[199] H. Mutlu, S. Mutlu and M. Bostancıklıoğlu, *Anti-Cancer Agents in Medicinal Chemistry*, 2021, **21**, 1732–1737.

[200] Y. Dai, L. Huang, H. Zhang, G. Hong, Y. He, J. Hu and Y. Liu, *Injury*, 2021, **52**, 11–18.

[201] Y. Yang, Y. Liu, N. Xie, L. Shao, H. Sun, Y. Wei, Y. Sun, P. Wang, Y. Yan, S. Xie and Y. Li, *Experimental and Therapeutic Medicine*, 2021, **22**, 1305.

[202] H. Wang, M. Chen, S. Xu, Y. Pan, Y. Zhang, H. Huang and L. Xu, *Journal of Clinical Laboratory Analysis*, 2021, **35**, e23945.

[203] L. Mei, Y. Zheng, X. Gao, T. Ma, B. Xia, Y. Hao, B. Wei, Y. Wei, Z. Luo and J. Huang, *Pharmacological Research*, 2022, **186**, 106537.

[204] A. Bogucka-Kocka, D. P. Zalewski, K. P. Ruszel, A. Stępniewski, D. Gałkowski, J. Bogucki, Łukasz Komsta, P. Kołodziej, T. Zubilewicz, M. Feldo and J. Kocki, *Frontiers in Genetics*, 2019, **10**, 1–14.

[205] H. Hozhabri, M. M. Moghaddam, M. M. Moghaddam and A. Mohammadian, *Scientific Reports*, 2022, **12**, 1–19.

[206] S. M. Wang, P. W. Yang, X. J. Feng, Y. W. Zhu, F. J. Qiu, X. D. Hu and S. H. Zhang, *Frontiers in Oncology*, 2021, **11**, 1–12.

[207] Y. Hou, X. Zhang, H. Yao, L. Hou, Q. Zhang, E. Tao, X. Zhu, S. Jiang, Y. Ren, X. Hong, S. Lu, X. Leng, Y. Xie, Y. Gao, Y. Liang, T. Zhong, B. Long, J. Fang and X. Meng, *EMBO reports*, 2023, **24**, 1–21.

[208] Y. Yang and Q. Wang, *Scientific Reports*, 2023, **13**, 1–15.

[209] Mathworks, *Bootstrap confidence interval - MATLAB bootci - MathWorks United Kingdom*, `https://uk.mathworks.com/help/stats/bootci.html`.

[210] M. B. Brown, *Biometrics*, 1975, **31**, 987.

[211] J. Abramson, J. Adler, J. Dunger, R. Evans, T. Green, A. Pritzel, O. Ronneberger, L. Willmore, A. J. Ballard, J. Bambrick, S. W. Bodenstein, D. A. Evans, C. C. Hung, M. O'Neill, D. Reiman, K. Tunyasuvunakool, Z. Wu, A. Žemgulytė, E. Arvaniti, C. Beattie, O. Bertolli, A. Bridgland, A. Cherepanov, M. Congreve, A. I. Cowen-Rivers, A. Cowie, M. Figurnov, F. B. Fuchs, H. Gladman, R. Jain, Y. A. Khan, C. M. Low, K. Perlin, A. Potapenko, P. Savy, S. Singh, A. Stecula, A. Thillaisundaram, C. Tong, S. Yakneen, E. D. Zhong, M. Zielinski, A. Žídek, V. Bapst, P. Kohli, M. Jaderberg, D. Hassabis and J. M. Jumper, *Nature*, 2024, **630**, 493–500.

[212] X.-J. Lu, H. J. Bussemaker and W. K. Olson, *Nucleic Acids Research*, 2015, **43**, e142.

[213] E. C. Meng, T. D. Goddard, E. F. Pettersen, G. S. Couch, Z. J. Pearson, J. H. Morris and T. E. Ferrin, *Protein Science*, 2023, **32**, e4792.
