# Supplementary material for: Disease-related miRNA mutations are associated with mature miRNA secondary structure changes
Source: Biophys J. 2025 Oct 3;124(23):4141–56. doi: 10.1016/j.bpj.2025.09.049 (PMC12709415; doi:10.1016/j.bpj.2025.09.049)
Supplement: Document S2. Article plus supporting material [file mmc2.pdf]

Biophysical Journal
Article

Biophysical Society

Disease-related miRNA mutations are associated with mature miRNA secondary structure changes

Javor K. Novev[1,2,*] and Sebastian E. Ahnert[1,3,*]
[1]Department of Chemical Engineering and Biotechnology, University of Cambridge, Cambridge, United Kingdom; [2]Institute of Genetics and Cancer, The University of Edinburgh, Western General Hospital, Edinburgh, United Kingdom; and [3]The Alan Turing Institute, London, United Kingdom

ABSTRACT MicroRNAs (miRNAs) are ubiquitous short RNAs regulating gene expression in many organisms, including humans. How the secondary structure (SS) of a mature miRNA affects its regulatory function remains an open question. Here, we investigate this question through computational SS predictions of miRNA point mutants. We explore the mutational neighborhoods of miRNAs with association to human diseases, including cancer. We focus on possible SS changes independent of target-site complementarity by leaving the seed region unchanged. We formulate metrics of the SS differences between such mutants and their wild types (WTs) and test whether disease-associated mutations tend to differ from others in terms of these metrics by comparing our results with the miRNASNP-v3 database. We find that disease-related mutants tend to have a higher probability of being fully unfolded than their WT; this and other SS-related measures are statistically significant at the database level. This is confirmed when we restrict the analysis to the better-validated miRNAs encoded by genes that appear in the manually curated MiRGeneDB database. With the same approach, we identify a subset of individual miRNAs for which SS changes are most likely to be related to disease. These are hsa-miR-1269b, hsa-miR-4537, hsa-miR-4477b, hsa-miR-4641, and hsa-miR-6821-3p; when focusing on the higher-confidence MiRGeneDB miRNAs, we find that hsa-miR-485-5p and hsa-miR-1908-3p are the ones for which SS changes are most likely to be linked to disease. In addition, we show that there are pairs of known miRNA WTs differing only by disease-related point mutations outside the seed region that exhibit very different SS. These pairs include hsa-miR-1269a—hsa-miR-1269b and hsa-miR-3689a-3p—hsa-miR-3689b-3p.

SIGNIFICANCE microRNAs regulate the expression of large numbers of genes, including many disease-associated ones, through binding to mRNAs as part of the RNA-induced silencing complex (RISC). Their mature form, which is found in RISC, is canonically thought not to have secondary structure, and the folding of mature microRNAs has received little attention in the literature. We use in silico tools to predict the folding of microRNA point mutants and then check whether mutations reported to be disease-related are associated with changes in secondary structure. We find that several independent measures of the secondary structure changes introduced by such mutations discriminate between disease-related and other mutations. Our work addresses a fundamental question in RNA biology that is relevant to many diseases.

INTRODUCTION

MicroRNAs (miRNAs) are short (typically 19–27 nt (1)) regulatory molecules that are highly conserved across many species (2). They regulate gene expression by entering an RNA-induced silencing complex (RISC) (3), which typically then binds to the 3′ untranslated region (UTR) of mRNAs and inhibits their translation (4–6), although recent work has also uncovered miRNA-induced translational activation via 5′ UTR binding (7). In humans and other animals, this binding canonically only occurs in the short "seed" region of the miRNA (nt 2–7) (8). This canonical picture is highly simplified; it is known that partial complementary binding to nucleotides outside this region is also common (9,10), and modes of binding that do not involve the seed have also been recorded (11). All modes of miRNA binding, however, rely on binding to a short target sequence that can be found in a large set of mRNAs. A single miRNA may therefore regulate a broad range of genes (2,3). Abnormal miRNA expression is observed in many diseases (4), including Parkinson's (12) and cancer (5,8,12–14), with evidence indicating that

Submitted April 16, 2025, and accepted for publication September 29, 2025.
*Correspondence: ynovev@ed.ac.uk or sea31@cam.ac.uk
Editor: Frauke Graeter.
https://doi.org/10.1016/j.bpj.2025.09.049
© 2025 The Author(s). Published by Elsevier Inc. on behalf of Biophysical Society.
This is an open access article under the CC BY license (http://creativecommons.org/licenses/by/4.0/).

miRNA expression is globally suppressed in tumor cells (4,5). Individual miRNAs typically have either oncogenic or tumor-suppressive effects (3,5), but several miRNAs (15) and miRNA families (16) can reportedly act as both oncogenes and tumor suppressors depending on context. Such a combination of tumor suppressive and carcinogenic effects is to be expected given the broad range of targets a single miRNA can regulate.

RNA molecules can fold due to pairing between some of their nucleobases, which can be described in terms of RNA secondary and tertiary structures. The role of miRNA secondary structure in the context of miRNA function has received some attention in the past, much of which has focused on miRNA precursors, also known as primary miRNAs (pri-miRNAs) or pre-miRNAs depending on their processing stage (2,17,18). In particular, Diederichs and Haber, who searched for tumor-associated mutations in cancer-derived cell lines, found no mutations within the mature miRNA sequences, and even though some of the pri-miRNA mutations dramatically altered secondary structure, they did not have an effect on in vivo processing and maturation (17). Sun et al. (19) investigated how single-nucleotide polymorphisms (SNPs) in mature miRNAs affect their function, noting that the variant miR-502-C/G produces a bulge that changes the structure of the pre-miRNA's stem and likely affects the latter's processing; a similar effect has also been observed in miR-125a (20). Confoundingly, there are some examples of SNPs that are associated with increased risk of some diseases but decreased risks for others; the polymorphism rs2910164 in miR-146a-3p is known to predispose carriers to breast cancer, glioma, and gastric cancer but at the same time is protective against prostate and gastric cancer (21).

It is not immediately clear to what extent the secondary structure of the mature miRNA affects its activity, since the single-stranded mature miRNA is typically bound to RISC, which may affect its folding. Before RISC binding, a pri-miRNA is converted into a pre-miRNA by the ribonuclease III enzyme Drosha and converted into a double-stranded RNA of length 22 nt, which binds to Argonaute (Ago) proteins to form RISC (22,23). However, known crystal structures of RISC proteins lack information on the position of some miRNA nucleotides, which suggests that miRNAs have some flexibility within the complex and may be able to form base pairs (24). An additional argument supporting the study of mature miRNA secondary structure is that other cases of base-pairing within ribonucleoproteins are known, with base-pairing within the ribosome (25) being a paradigmatic example. The filaments containing the genetic material of the influenza virus are another example. Although it was previously thought that RNA secondary structure is completely melted within these complexes, i.e., that there is no base-pairing, base-pair formation within them is now known to be possible (26) and has been experimentally observed; see Ref. (27) and the references therein, as well as the review of the topic in Ref. (28).

Belter et al. (29) showed that some mature miRNAs form stable secondary structures and suggested that these play a functional role. In a different study (30), the same group identified common secondary structure motifs in mature miRNAs and proposed that 1) base-pairing may confer resistance to nucleases, and that 2) target recognition may involve not only miRNA sequence but also secondary and tertiary structure. A striking example of mature miRNA folding playing a role in disease is the single-stranded mature miR-1-3p, which assumes aptamer-like secondary structure, modifying the function of ion channels in cardiomyocytes. Genetic variants affecting this miRNA, for example rs1399433486, have been reported in patients with cardiovascular diseases such as juvenile onset atrial fibrillation (31). This and a host of other biological functions of mature miRNAs beyond RISC have been reviewed by Santovito and Weber (31) and Makarova et al. (32). Moreover, evidence from multiple studies (33,34) indicates that for many miRNAs a large fraction of the molecules in vivo are not bound to RISC, but it is not currently understood where these miRNAs are located (33).

The generally low frequency of mutations in mature miRNAs has been commented on by Bracken et al. (6), who presume this is due to their small size. SNPs, particularly ones in the seed region, are associated with many diseases (22). In reviewing the literature on genetic variations in miRNAs, Borel and Anonarakis remark that data indicate a low density of polymorphisms in the seed region, suggesting a selective constraint (35).

To our knowledge, however, no study has systematically explored mutation-induced changes in mature miRNA secondary structure or attempted to analyze the association between such changes and disease; it is this knowledge gap that we address in the present work. We hypothesize that miRNA activity could be influenced by secondary structure, because the folding of a mature miRNA may reduce its binding affinity to target mRNAs and might influence its activity within RISC, as illustrated schematically in Fig. 1, or affect non-canonical miRNA function, for example by protecting free miRNAs from degradation, as hypothesized in Ref. (32). Other researchers have designed the miRVaS tool in an attempt to predict the impact of genetic variants on miRNAs (36) based on how they affect secondary structure of miRNA precursors. Our aim, however, is not to predict the effect of mutations but to ascertain whether the secondary structure of mature miRNAs is relevant to their activity based on mutations previously characterized as associated with disease.

We study the effect of secondary structure in mature miRNAs through predicting the secondary structure changes introduced by point substitution mutations that preserve the seed region. In genotype space, such a set forms a "partial" point mutational neighborhood of the WT miRNA under consideration, as opposed to the "complete" point mutational neighborhood, which contains all mutants accessible from the WT via a point substitution mutation. We then formulate multiple quantitative criteria for measuring how

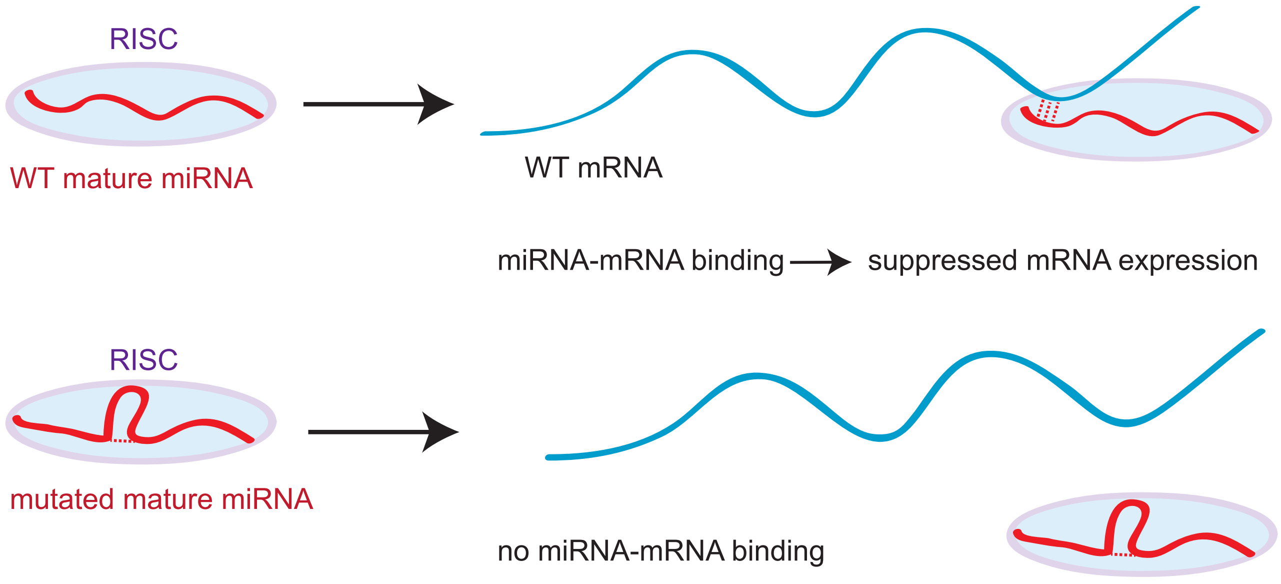

FIGURE 1 We hypothesize that miRNA mutations outside the seed region that change miRNA secondary structure can affect miRNA-mRNA binding and thus gene expression. Illustration of the proposed mechanism by which mature miRNA SS may influence miRNA activity. Folding of the mutant miRNA (*bottom*) reduces its affinity to target mRNAs in comparison with the WT (*top*). Our methods cannot provide mechanistic insights into miRNA activity, but it is possible that both miRNA activity within RISC and non-canonical pathways such as those detailed in Refs. (31,32) are affected by changes in mature miRNA secondary structure.

the secondary structure of each mutant in the partial neighborhood differs from the wild type (WT). Thereafter, we test how well these criteria can predict which mutations are associated with disease. We do so by ranking the mutated sequences by a given criterion of secondary structure change and assess whether disease-related mutants are ranked higher than expected by chance.

When studying how mutations affect secondary structure, we place a particular emphasis on calculating how likely the fully unfolded state, i.e., the one with trivial secondary structure, is. Since miRNAs regulate gene expression by binding to mRNAs, one may expect that mutations that alter the probability that a given miRNA is fully unfolded may also affect its propensity to bind, as an unfolded molecule may interact more readily with its target than a stably folded one. Mutations that lower the likelihood that a miRNA is unfolded may affect miRNA function in this way and thus be associated with disease. This is one of the key hypotheses that we test in this study.

METHODS

Theoretical basis

Secondary structure prediction

We use version 2.5.0 of the ViennaRNA package (37) to predict and analyze the secondary structure of mutant and WT miRNAs.

Statistical significance of ROC results

We use the area under the receiver operating characteristic (ROC) curves, A_{ROC}, as a measure of the power of $\Delta p_{\mathrm{unfolded}}$ and other SS metrics as predictors of the association of a given mutation with disease. We then assess the statistical significance of the results by calculating the two-sided Mann-Whitney p-values for the null hypothesis that the $\Delta p_{\mathrm{unfolded}}$ distributions for the disease-related and other mutants have the same median; we use the built-in MATLAB R2022b function for this purpose (38). The error bars in ROC plots indicate pointwise 95% confidence bounds for the true positive rates and are calculated via bootstrapping with 10^4 samples and vertical averaging for 21 equally spaced values of the false positive rate from 0 to 1; for more details, see (39) and the references cited therein.

Boltzmann frequency of structures

The frequency of a particular secondary structure s with energy $E(s)$ in the thermodynamic equilibrium ensemble is (40)

$$p_s = \frac{1}{Z} \exp\left(-\frac{E(s)}{RT}\right), \tag{1}$$

with R denoting the gas constant, and T is the temperature, for which we use the biologically relevant value 310.15 K, and $Z = \sum_{\forall s} \exp\left(-\frac{E(s)}{RT}\right)$, the partition function; for the fully unfolded state, there is no base-pairing, $E(s) = 0$, and $p_{\mathrm{unfolded}} = Z^{-1}$.

RNAsubopt

We use RNAsubopt to generate a sample of structures drawn randomly from the Boltzmann ensemble according to their probability, starting with a sample size of 10. In case the fully unfolded structure is not present in the initial sample, we increase the sample size by a factor of 10, and we repeat the process until we either encounter the trivial secondary structure or reach 10^{10} samples. In the latter case, the Boltzmann weight for this structure is negligible, and we use the approximation $p_{\mathrm{unfolded}} \approx 0$. A sample size as small as 10 can be sufficient because RNAsubopt calculates the partition function Z of the ensemble and uses stochastic backtracing to generate suboptimal structures (41). The Boltzmann weights from RNAsubopt simply reflect Eq. (1), making them independent of the sample size. In contrast, when using samples generated with RNAsubopt to approximate thermodynamic ensembles and calculate distances between them, the sample size needs to be large

enough to find all folds with appreciable probability. For this reason, when calculating average Hamming distances between ensembles, we use 10^5 samples for mature miRNAs and 10^3 samples for pre-miRNAs, with the reason for using fewer samples in the latter case being the computational cost of sampling for the much longer precursors.

Hamming distance metric

We measure the differences between two secondary structures s_1 and s_2 in the dot-bracket representation, where dots represent unpaired sites and brackets represent base pairs (see for example (42)), via the Hamming distance between the two, i.e., the number of sites for which there are discrepancies ($d_{\mathrm{Hamming}}(s_1,s_2)$). For each mutant in the partial point mutational neighborhoods of the miRNAs in miRNASNP-v3 (Database: miRNASNP-v3, https://guolab.wchscu.cn/miRNASNP/), we average the pairwise Hamming distance between the possible secondary structures in its equilibrium ensemble and those for the equilibrium ensemble of the WT, weighted by their joint Boltzmann probability,

$$\langle d_{\mathrm{Hamming\ mutant}} \rangle = \sum_{\forall s_{\mathrm{mutant}}} \sum_{\forall s_{\mathrm{WT}}} p_{s_{\mathrm{mutant}}} p_{s_{\mathrm{WT}}} d_{\mathrm{Hamming}}(s_{\mathrm{mutant}}, s_{\mathrm{WT}}). \quad (2)$$

The sums in Eq. (2) contain the secondary structures that RNAsubopt has drawn from the respective equilibrium ensembles on the basis of a sample with 10^5 entries for mature miRNAs and 10^3 samples for pre-miRNAs. Structures that have not been encountered in drawing this sample have only a negligible contribution to $\langle d_{\mathrm{Hamming}} \rangle$. For the criteria based on $\langle d_{\mathrm{Hamming}} \rangle L^{-1}$, unlike most other quantities we study, we rank mutants in "ascending order," which means that top-ranked entries are closest to the WT. We do this to ease the readability of the results because for large data sets, mutants associated with disease tend to be closer in secondary structure to the WT than other mutants; however, this trend is reversed for some individual point mutational neighborhoods such as that of the microRNA hsa-miR-4537, which is illustrated in Fig. 3.

Additional metrics of secondary structure distance between ensembles

We use two other metrics that characterize how far the Bolztmann ensemble of a miRNA mutant is from its WT. The first one is based on the distance calculated by the ViennaRNA program RNApdist (37). RNApdist calculates a vector with the probabilities that each base is unpaired, paired upstream, or paired downstream; these vectors are then compared via an alignment algorithm. In addition to RNApdist, we use a simpler metric: for each base, we calculate the probability that it is paired to any other base $p_{\mathrm{bp}}^{(\mathrm{i})}$ from the output of RNAfold (37), average this quantity over the entire molecule, and then calculate the difference between these averages for the mutant and the WT as $\langle \Delta p_{\mathrm{bp}} \rangle = \langle p_{\mathrm{bp,mutant}}^{(\mathrm{i})} - p_{\mathrm{bp,WT}}^{(\mathrm{i})} \rangle$.

Positional entropy

The positional entropy of site "i" within an RNA molecule is defined as

$$S^{(\mathrm{i})} = -\sum_{\mathrm{j} \neq \mathrm{i}} p_{\mathrm{i,j}} \ln p_{\mathrm{i,j}} - p_{\mathrm{i}}^{\mathrm{unpaired}} \ln p_{\mathrm{i}}^{\mathrm{unpaired}}, \quad (3)$$

where $p_{\mathrm{i,j}}$ is the probability that site "i" is paired with site "j," and $p_{\mathrm{i}}^{\mathrm{unpaired}} = 1 - \sum_{\mathrm{j} \neq \mathrm{i}} p_{\mathrm{i,j}}$ is the probability that site "i" is unpaired (43). When building ROC curves for $S^{(\mathrm{i})}$, we sort mutants in "ascending order," as we do for the criteria based on the mutant-WT Hamming distance. This means that the top-ranked entries are those for which the difference in positional entropy at the mutated site is lowest.

Measures of secondary structure change in mutants

Change in the probability of the fully unfolded state

We calculate the Boltzmann probabilities $p_{\mathrm{unfolded,WT}}$ and $p_{\mathrm{unfolded,\ mutant}}$ that WT and mutant miRNAs are fully unfolded, using the ViennaRNA

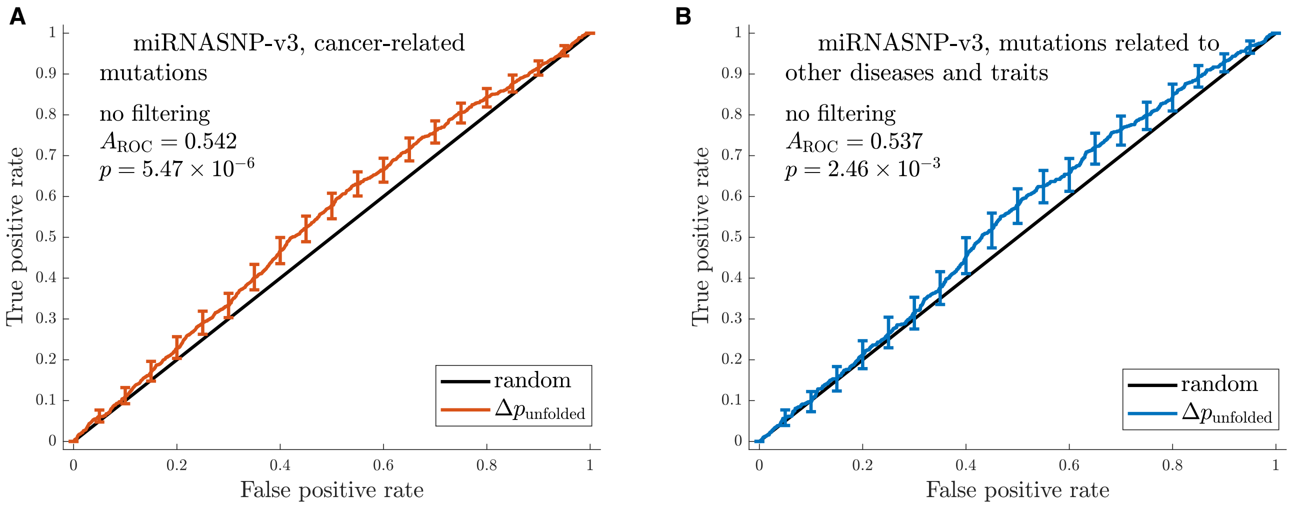

FIGURE 2 The change in probability that the miRNA is fully unfolded associated with a mutation is a predictor of the mutation's relationship with disease. ROC curves were built using $\Delta p_{\mathrm{unfolded}} = p_{\mathrm{unfolded\ mutant}} - p_{\mathrm{unfolded\ WT}}$ as the criterion for predicting disease-related mutations. Curves are based on all data on cancer-related mutations (*A*) and mutations related to other traits and diseases (*B*) from miRNASNP-v3, with no filtering based on $N_{\mathrm{mut\ seed}}$ and $N_{\mathrm{mut\ non\text{-}seed}}$. Error bars indicate pointwise 95% confidence bounds for the true positive rates in the ROC plots calculated via bootstrapping and vertical averaging for 21 equally spaced values of the false positive rate from 0 to 1; see (39) and the references cited therein. The area under the ROC curve (A_{ROC}) and the Mann-Whitney p-value (38) for the curves are also indicated. The $\Delta p_{\mathrm{unfolded}}$ criterion performs significantly better than the random one for both data sets ($p < 0.05$), indicating that the probability that disease-related mutants are fully unfolded tends to be higher than that for other mutants. This could be because mutants with a higher $p_{\mathrm{unfolded\ mutant}}$ have a higher activity than their respective WTs, and in the case of cancer-associated mutations, they may be more effective at downregulating tumor suppressor genes.

program RNAsubopt (see methods). We then consider the difference between these two quantities,

$$\Delta p_{\text{unfolded}} = p_{\text{unfolded,mutant}} - p_{\text{unfolded,WT}},$$

for all mutants in the point mutational neighborhood of the WT except those that have an altered seed region and rank them by $\Delta p_{\text{unfolded}}$. We establish whether $\Delta p_{\text{unfolded}}$ is a useful predictor of the association between mutations and disease by ranking all mutants in "descending order" of their values of $\Delta p_{\text{unfolded}}$ and building an ROC curve (44), which indicates whether disease-related mutants tend to rank higher than the rest (Fig. 2).

Change in the secondary structure Boltzmann ensemble

If the folding of a miRNA plays a role in its function, then disease-related mutations can be expected to be the ones that cause the greatest change in miRNA secondary structure. We formulate quantitative measures of the phenotypic distance of mutants from their WTs and rank the mutants in "descending order" according to these measures, i.e., compile a ranking with mutants that are furthest away from the WT at its top. Since our hypothesis is that miRNA secondary structure impacts function, we expect mutants that rank high according to these criteria to have a stronger association with disease than other mutants. We quantify the difference between two secondary structure Boltzmann ensembles by calculating the average Hamming distance across all pairwise comparisons between structures in the two different ensembles (see theoretical basis). We then use the average mutant-WT Hamming distance $\langle d_{\text{Hamming}} \rangle$ to formulate a coarse-grained criterion, namely the percentile ranking of each individual mutant within its point mutational neighborhood. We build ROC curves for this criterion based on data from miRNASNP-v3, and check whether the corresponding Mann-Whitney p-values fall below our significance threshold of 0.05.

Change of the positional entropy of the mutated site

For any given site in an RNA molecule, we can calculate the positional entropy $S^{(i)}$, which measures how variable the pairing of this site is within the Boltzmann ensemble. A site with low $S^{(i)}$ is thus one that is consistently paired or unpaired across the ensemble, whereas a high $S^{(i)}$ indicates that the site participates in pairings in many structures but is unpaired in many others. We quantify this effect for all mutants in the partial point mutational neighborhoods of the miRNAs represented in miRNASNP-v3 by calculating the difference between the positional entropy of the mutated site for mutant and the WT,

$$\Delta S^{(i)} = S^{(i)}_{\text{mut}} - S^{(i)}_{\text{WT}},$$

as a measure of this change in stability (see theoretical basis).

Distance between Boltzmann ensembles

We calculate this with the ViennaRNA program RNApdist (37) and normalized by its maximum possible value $d_{\text{Boltzmann}} = d_{\text{RNApdist}}/(2L)$.

Difference between the average base-pairing probabilities in the mutant and the WT

This quantifies the overall change in secondary structure caused by point substitution mutations. As explained in the theoretical basis section, we calculate it as $\langle \Delta p_{\text{bp}} \rangle = \langle p^{(i)}_{\text{bp,mutant}} - p^{(i)}_{\text{bp,WT}} \rangle$, where $p^{(i)}_{\text{bp}}$ is the probability that base "i" is paired to any other base, calculated from the output of RNAfold (37).

The secondary structure of an miRNA may have an effect on its function in two ways; it could either have its own functional purpose, or it could affect the interaction with the target site as it would make the binding sites within the miRNA less accessible. In the first case, a mutant may disrupt function by causing a change to the minimum free energy (MFE) secondary structure or making it less stable; in the second one, a mutation may interfere with miRNA function by causing sites essential to target-binding to enter base pairs. The five metrics outlined above aim to quantify different types of secondary structure changes in order to detect any kind of association between modified miRNA secondary structure and disease.

Sources of data on disease-related mutations

Although disease-associated miRNAs exhibit fewer SNPs than non-disease-related miRNAs (45), there is a substantial amount of data on the association of specific miRNA point mutations with disease. We used a database of this kind to examine the changes in secondary structure as a result of disease-related mutations. This database is miRNASNP-v3 (46), which contains data on miRNA mutations related to various diseases. We also analyzed the somatic mutations in cancer collected in the SomamiR 2.0 database (47) (Database: SomamiR 2.0, https://compbio.uthsc.edu/SomamiR/home.php), but as almost all of them are covered in miRNASNP-v3, we only discuss these results in the supporting material. Note that other online databases with mutations in miRNAs exist. For an overview, see Fehlmann et al. (48). The miRNASNP-v3 database, which we use in its version from May 14th, 2024, contains 2613 distinct entries recorded for the mature region. We filtered the mutations in the miRNASNP-v3 database by several criteria. Firstly, we filter out the 17 entries in miRNASNP-v3 for which the related disease was unspecified and a further 34 mutations for which the WT miRNA sequence in the miRNASNP-v3 entry is not an exact match for the one given in miRBase (49), or for which the mutated miRNA sequence specified in miRNASNP-v3 does not match the one derived from mutating the corresponding DNA sequence in the Genome Reference Consortium Human Build 38 (50).

The stringency of criteria for miRNA discovery varies by study (49), and it is difficult to distinguish bona fide miRNAs from fragments of other RNAs (49). Thus, many reported miRNAs are false positives, and previous studies have reported a false positive rate of up to two-thirds (51). As miRBase only provides minimal gate-keeping at the point of submitting a new miRNA entry (49), merely cross-checking that a miRNA is found in miRBase as we do above allows for low-confidence miRNAs in our data sets. It is not our aim here to curate or assess the quality of miRNA data, so for a more stringent filtering miRNA mutations, we make use of the manually curated database MiRGeneDB 2.1 (51) (Database: MiRGeneDB, https://web.archive.org/web/20230402091252/https://mirgenedb.org/download). We report the results of restricting our analysis only to the set of miRNAs encoded by genes that appear in the version of MiRGeneDB 2.1 (51) downloaded on October 12th, 2024. We provide information on the number of mutants in the different datasets in Table 1.

In some of the analysis that follows, we consider filtered subsets of the data with different minimum numbers of mutations in the seed region or the non-seed region, as the presence of several disease-associated mutations in a given miRNA suggests a higher likelihood that the miRNA is directly involved in the disease mechanism.

RESULTS

A significant proportion of disease-related miRNA mutations are associated with secondary structure changes

In Table 2, we compare the three best-performing of the quantitative measures outlined above in terms of their power to predict disease-related mutations. To illustrate the performance of the criteria, we present the underlying ROC curves for $\Delta p_{\text{unfolded}}$ in Fig. 2. We apply the three criteria to data from miRNASNP-v3, split between mutations associated with cancer and other traits and diseases. We provide additional ROC curves in the supporting material, where we give details of other criteria we considered.

TABLE 1 Number of mutations in the datasets we analyzed split by the type of disease they are associated with (cancer vs. others), their location (seed vs. non-seed), and whether the genes encoding for them appear in MiRGeneDB

Data set	Total	Seed	$N_{\text{mature miRNAs affected}}$
All valid miRNASNP-v3 entries	2562	783	1209
		Number of mutations	
Point substitutions outside the seed region		1693	
Cancer-associated point substitutions outside the seed region		1105	
Point substitutions outside the seed region associated with other diseases		588	
	Total	Cancer	Other diseases
Number of unique sequences	1526	1013	564
Total number of mutants studied		32,040	18,876
$N_{\text{mature miRNAs affected}}$	938	675	400
Number of unique sequences of mutants in miRNAs represented in MiRGeneDB 2.1	553	441	124
Total number of mutants in miRNAs represented in MiRGeneDB 2.1		14,211	4566
$N_{\text{mature miRNAs affected}}$ represented in MiRGeneDB 2.1	336	295	95

$\Delta p_{\text{unfolded}}$ is significantly better at predicting disease-associated mutations than the random criterion for data from miRNASNP-v3, suggesting that miRNAs with a fully unfolded mature form differ in activity from folded ones. In particular, disease-related mutations tend to increase the likelihood that a miRNA is unfolded more than other mutations. We show the ROC curves for this criterion for the two subsets of mutations from miRNASNP-v3 in Fig. 2. We verify that mutants with large $\Delta p_{\text{unfolded}}$ tend to cause a change to a less folded structure by calculating the Spearman rank correlation coefficient of $\Delta p_{\text{unfolded}}$ with the average change in base-pairing probability, $\langle \Delta p_{\text{bp}} \rangle$. The resulting $R_{\text{Spearman}} \sim -0.30$ with p-value $< 10^{-100}$ shows comparatively weak but very significant negative correlation; i.e., as one would expect, the fully unfolded state tends to be more common in the mutated ensemble when the mutation causes an overall reduction in base-pairing.

Using the percentile ranking of $\langle d_{\text{Hamming}} \rangle$ of mutants within their neighborhoods also leads to A_{ROC} significantly greater than 0.5 for data from miRNASNP-v3. This is an independent indication that miRNA secondary structure plays a role in miRNA association with disease. Since we sort mutants in "ascending order" of this criterion, the analysis indicates that disease-related mutations tend to change secondary structure less than other mutations. The criterion based on $\Delta S^{(i)}$, the difference in the positional entropy of the mutated site, performs better than random for the data in miRNASNP-v3. As we rank mutants in "ascending order," this means that disease-associated mutants tend to be those for which the difference $\Delta S^{(i)} = S^{(i)}_{\text{mut}} - S^{(i)}_{\text{WT}}$ is lower.

We also calculated correlation coefficients between other pairs of secondary structure change quantifiers. $R_{\text{Spearman}} \sim -0.1$ between $\Delta p_{\text{unfolded}}$ and the percentile of $\langle d_{\text{Hamming}} \rangle$, indicating that these can be treated as orthogonal criteria. $R_{\text{Spearman}} \sim -0.4$ between $\Delta p_{\text{unfolded}}$ and $\Delta S^{(i)}$, and $\sim$0.3 between $\Delta S^{(i)}$ and $\langle d_{\text{Hamming}} \rangle$, which indicates that the rankings according to these criteria are more strongly correlated. We observe the same general trends when measuring correlations between the same criteria for a set of miRNAs highlighted by multiple hypothesis testing as described in the section entitled disease-related mutations change the secondary structures of a specific set of individual miRNAs below. We provide details on R_{Spearman} and the associated p-values for several pairs of criteria in Tables S11–S13 in the supporting material.

We checked whether the three main criteria, $\Delta p_{\text{unfolded}}$, the $\langle d_{\text{Hamming}} \rangle$ percentile, and $\Delta S^{(i)}$, highlighted similar sets of mutations. In accordance with the order of ranking mutants we use for the respective criteria, we took the 1% of mutants with the highest $\Delta p_{\text{unfolded}}$ and the 1% of mutants with the lowest $\langle d_{\text{Hamming}} \rangle$ percentile and $\Delta S^{(i)}$—this defines the sets of top 1% of mutants according to each of these criteria. We then tested whether individual mutants were among the 1% selected according to more than one criterion

TABLE 2 The three main criteria for measuring changes in miRNA secondary structure as a result of mutations are $\Delta p_{\text{unfolded}}$, the $\langle d_{\text{Hamming}} \rangle$ percentile, and $\Delta S^{(i)}$

	Cancer		Other diseases	
Criterion	A_{ROC}	p-value	A_{ROC}	p-value
$\Delta p_{\text{unfolded}}$	0.542	5.47×10^{-6}	0.537	2.46×10^{-3}
$\langle d_{\text{Hamming}} \rangle$%ile	0.548	1.61×10^{-7}	0.532	1.04×10^{-2}
$\Delta S^{(i)}$	0.528	2.62×10^{-3}	0.534	5.41×10^{-3}

Mutations are ranked by these criteria, and the receiver operator characteristic A_{ROC} is used to establish whether they predict disease-associated mutations in the miRNASNP-v3 data set, split into mutations associated with cancer and those with other diseases. The A_{ROC} values exceed 0.5 significantly for all three metrics in both subsets of the data, suggesting that at least for some miRNAs, secondary structure changes can be indicative of disease association. When checking for significance, we calculate the Mann-Whitney p-value and set the threshold for significance to 0.05.

TABLE 3 $\Delta p_{unfolded}$ consistently meets our significance threshold when applied to the data for the subset of high-confidence miRNAs from miRNASNP that are represented in MiRGeneDB 2.1

	Cancer		Other diseases	
Criterion	A_{ROC}	p-value	A_{ROC}	p-value
$\Delta p_{unfolded}$	0.559	2.25×10^{-5}	0.562	1.76×10^{-2}
$\langle d_{Hamming} \rangle$%ile	0.550	3.05×10^{-4}	*0.535*	*0.69*
$\Delta S^{(i)}$	0.543	2.23×10^{-3}	*0.490*	*0.18*

The other two main criteria, $\langle d_{Hamming} \rangle$ percentile and $\Delta S^{(i)}$, only meet the significance threshold when applied to the cancer-related mutations in miRNASNP-v3, which also appear in MiRGeneDB 2.1. As elsewhere, mutations are ranked by these criteria, and the area under the receiver operator characteristic curve (A_{ROC}) is used to establish whether they predict disease-associated mutations in the miRNASNP-v3 data set, split into mutations associated with cancer and those with other diseases. When checking for significance, we calculate the Mann-Whitney p-value and set the significance threshold to 0.05; results with p-values above this threshold are in *italics.*

and found very few such mutations. Within the set of mutations related to cancer, the top 1% sets of mutants according to $\Delta S^{(i)}$ and $\Delta p_{unfolded}$ have one mutant in common, and the same is true of the top 1% sets defined according to $\langle d_{Hamming} \rangle$% ile and $\Delta S^{(i)}$. In the set of mutations related to other diseases, one is common between the top 1% defined according to $\langle d_{Hamming} \rangle$% ile and $\Delta S^{(i)}$. This shows that although the three rankings are significantly correlated with each other, they point to different sets of mutations as most likely to be associated with disease. These differences introduce uncertainty in our conclusions that may be due to different mechanisms by which miRNA secondary structure influences miRNA function, but also due to the indirect relationship between miRNA mutations and disease. Ideally, future experimental study of the mutational neighborhoods of selected miRNAs will help resolve these issues.

The criterion measuring the distance between the mutated and WT Boltzmann ensembles, $d_{Boltzmann}$, has an A_{ROC} >0.5 and significantly better than random performance when applied to mutants from the miRNASNP-v3 cancer data set that are put in "ascending order." Equivalently, in this set the mutations that cause a smaller change to the Boltzmann ensemble tend to be associated with disease. The difference between the average base-pairing probabilities in the mutant and the WT ($\langle \Delta p_{bp} \rangle$) does not perform better than the random criterion for the miRNASNP-v3 data sets with no filtering by the number of mutations. For brevity, we only provide A_{ROC} and p-values for $d_{Boltzmann}$ and $\langle \Delta p_{bp} \rangle$ in Tables S3–S10 in the supporting material.

As Table 3 indicates, restricting the analysis only to the high-confidence miRNAs encoded by genes from MiRGeneDB reduces the statistical power of our methods. Despite that, the $\Delta p_{unfolded}$ criterion consistently meets our significance threshold when applied to the data for the subset of miRNAs from miRNASNP that are represented in MiRGeneDB 2.1. The other two main criteria, $\langle d_{Hamming} \rangle$ percentile and $\Delta S^{(i)}$, meet our threshold for significance only when applied to the cancer-related mutations in miRNASNP-v3 that also appear in MiRGeneDB 2.1. Note, however, that the p-values for all three main criteria ($\Delta p_{unfolded}$, $\langle d_{Hamming} \rangle$ percentile, and $\Delta S^{(i)}$) and $d_{Boltzmann}$ are below our threshold of 0.05 when applied to the miRNASNP data set without splitting according to disease, regardless of whether cross-validation with MiRGeneDB is performed; see Tables S1 and S2 in the supporting material for details. We considered alternative approaches to quantifying secondary structure changes as well as ways to filter the data according to the number of mutations; the interested reader may find them in the supporting material.

Disease-related mutations change the secondary structures of a specific set of individual miRNAs

We next apply the criteria formulated above to data for mutations in individual miRNAs. We use data from the miRNASNP-v3 database for both cancers and other traits and diseases, and we focus on the two criteria that work best for the larger data sets—one based on the change in the probability that the miRNA is completely unfolded ($\Delta p_{unfolded}$) and the other based on the normalized average Hamming distance from the WT ($\langle d_{Hamming} \rangle L^{-1}$). Note that at the level of the individual miRNA, $\langle d_{Hamming} \rangle L^{-1}$ and the miRNA-specific percentile of $\langle d_{Hamming} \rangle$ produce identical rankings. We observe a signal in $\Delta p_{unfolded}$ ($p < 0.05$) for 44 miRNAs, listed in Table S17 (see supporting material), and $\langle d_{Hamming} \rangle L^{-1}$ for 48, listed in Table S18, and for 13 miRNAs $p < 0.05$ for both criteria. Since we observe that multiple criteria perform better than random for some miRNAs, we also combine the p-values obtained from independent tests using Fisher's method (52). We do that for $\Delta p_{unfolded}$ and $\langle d_{Hamming} \rangle L^{-1}$, and we give the results for miRNASNP-v3 in Table S19 of the supporting material. We observe a signal in 46 miRNAs from this set, including seven miRNAs for which the combined p-value is below 0.05, whereas the individual Mann-Whitney p-values are not. Intriguingly, we observe that the distributions of A_{ROC} within the sets of miRNAs for which these two criteria perform better than random exhibit signs of bimodality, with clear peaks around 0 and 1 and few, if any, points in between them. We quantify this observation by calculating the bimodality coefficients of the distributions according to (53), and we obtain the values 0.714 for $\Delta p_{unfolded}$ and 0.745 for $\langle d_{Hamming} \rangle L^{-1}$. Coefficient values above 5/9 suggest bimodality (53,54). A possible explanation of this result is that the WTs of miRNAs clustered around one peak contribute to the initiation and/or progression of disease, whereas those around the other peak are essential for disease-prevention, e.g., because they act as tumor suppressors. In that case, if miRNA folding affects function, we would expect that the mutants most strongly associated with disease in disease-suppressing miRNAs

would be those that change the secondary structure the most, which would translate into A_{ROC} >0.5 for $\Delta p_{unfolded}$ and A_{ROC} <0.5 for $\langle d_{Hamming}\rangle L^{-1}$. The comparatively small overlap between the miRNAs for which $\Delta p_{unfolded}$ and $\langle d_{Hamming}\rangle L^{-1}$ perform significantly better than random suggests that some miRNAs need to be fully unfolded to perform their functions, whereas for others the folding itself plays a functional role.

When performing a large number of statistical tests, as we do in the current section for hundreds of miRNAs, a p-value of <0.05 is not a sufficient indicator of statistical significance. For this reason, in addition to testing whether $\Delta p_{unfolded}$ fulfills the Mann-Whitney test for statistical significance for individual miRNAs, we estimate the positive false discovery rate (pFDR), which is the FDR in case there is at least one positive finding. We then calculate the q-value, an FDR-based measure of significance equal to the minimum positive false discovery rate at which a test with p-value p_i is considered significant. We compute the Benjamini-Hochberg linear step-up procedure as implemented in the built-in MATLAB R2022b function mafdr (55). We consider various levels of filtering by $N_{mut\ seed}$ and $N_{mut\ non\text{-}seed}$ since we expect that a large number of recorded disease-related mutations for a particular miRNA implies a stronger association between the latter miRNA and disease. Moreover, focusing on a smaller number of miRNAs makes it possible to obtain lower q-values as it requires fewer tests. The number of unique sequences with mutations outside the seed is 452 at $N_{mut\ seed} \geq 1$ and $N_{mut\ non\text{-}seed} \geq 1$, 172 at $N_{mut\ seed} \geq 2$ and $N_{mut\ non\text{-}seed} \geq 2$, and 113 at $N_{mut\ seed} \geq 3$ and $N_{mut\ non\text{-}seed} \geq 3$. For the reduced data set with miRNAs whose respective genes appear in MiRGeneDB, these numbers are as follows: 145, 29 at $N_{mut\ seed} \geq 1$ and $N_{mut\ non\text{-}seed} \geq 1$, at $N_{mut\ seed} \geq 2$ and $N_{mut\ non\text{-}seed} \geq 2$, and at $N_{mut\ seed} \geq 3$ and $N_{mut\ non\text{-}seed} \geq 3$. For this smaller data set, we also use finer graining in the filtering process, and we require $N_{mut\ seed} \geq 0$, varying the minimum level of $N_{mut\ non\text{-}seed}$. In this case, the number of unique non-seed mutants is 372 and 202 at $N_{mut\ non\text{-}seed} \geq 2$ and $N_{mut\ non\text{-}seed} \geq 3$, respectively.

Using a q-value threshold of 0.05, we find five miRNAs (given in Table 4) for which the association between disease-related mutations and secondary structure changes is significant (in one case, borderline). We employ two different filtering regimes, the first requiring at least one mutation in the seed region and non-seed region ($N_{mut\ seed}$, $N_{mut\ non\text{-}seed} \geq 1$) and the second requiring at least three mutations in each region ($N_{mut\ seed}$, $N_{mut\ non\text{-}seed} \geq 3$). The former covers a wider range of mutants, but this means a higher bar for significance when using the Benjamini-Hochberg procedure. The latter focuses on a smaller number of miRNAs that appear to have many disease-related mutations and are thus likely to be disease-associated. Fig. 3 is an illustration of the data underlying Table 4 for individual miRNAs with the tumor suppressor hsa-miR-4537 as an example. The figure contains ROC plots characterizing the performance of $\Delta p_{unfolded}$ and $\langle d_{Hamming}\rangle L^{-1}$, both of which have an associated p-value of less than 0.05.

TABLE 4 Benjamini-Hochberg *q*-values, i.e., minimum positive false discovery rates for which we have found a significant association between secondary structure changes and disease after accounting for multiple hypothesis testing

	Filtering levels	
miRNA	$N_{mut\ seed}$, $N_{mut\ non\text{-}seed} \geq 1$	$N_{mut\ seed}$, $N_{mut\ non\text{-}seed} \geq 3$
**hsa-miR-1269b**	2.666×10^{-2} *	–
**hsa-miR-4477b**	6.792×10^{-2}	1.313×10^{-2} *
**hsa-miR-4537**	6.792×10^{-2}	1.864×10^{-2} *
**hsa-miR-4641**	2.666×10^{-2} *	–
**hsa-miR-6821-3p**	>0.3	6.299×10^{-2}

The q-values are based on combined Mann-Whitney p-values for $\Delta p_{unfolded}$ and $\langle d_{Hamming}\rangle L^{-1}$. Significant values ($q$ < 0.05) are emphasized with an asterisk. The first two miRNAs (hsa-miR-4641 and hsa-miR-1269b) have fewer than three miRNASNP-v3 entries for mutations in the seed region or the non-seed region, and they are thus excluded by the more stringent filter ($N_{mut\ seed}$, $N_{mut\ non\text{-}seed} \geq 3$). The last miRNA, hsa-miR-6821-3p, is borderline significant but is nevertheless mentioned here because it becomes fully significant ($q = 2.996 \times 10^{-2}$) if we combine the p-values for $\Delta p_{unfolded}$ and $\langle d_{Hamming}\rangle L^{-1}$ with that for $\Delta S^{(i)}$. All four miRNAs have been linked with disease (56–62). Note that two pairs of miRNAs have equal q-values. The reason for this is that the p-values for mutants come from a small discrete set, which can yield the same q-value in the Benjamini-Hochberg procedure that ranks them in ascending order and multiplies them by a factor involving their rank.

We use the Fisher method to calculate a combined p-value for the two criteria and apply the Benjamini-Hochberg procedure to calculate the minimum false discovery at which the results are significant, i.e., the q-value. Applying a more stringent filter by the number of reported disease-related mutations in the mature region decreases the q-value because it restricts the set of miRNAs whose p-values are processed by the Benjamini-Hochberg method, excluding miRNAs with few mutations for which the uncertainty is greater.

Of the five miRNAs, three (hsa-miR-1269b, hsa-miR-4537, and hsa-miR-4477b) are known to play a role in cancers: hsa-miR-1269b is associated with hepatocellular carcinoma (56–58), a mutation in the gene coding for hsa-miR-4477b has been reported to occur during the transformation of colorectal adenoma into colorectal cancer (59), and hsa-mir-4537 is a tumor suppressor in gastric cancers (60). hsa-miR-6821-3p has been reported to be an extracellular genomic biomarker of osteoarthritis (61) and a potential biomarker for cancer (62).

One reason that only a small number of individual miRNAs are highlighted by this approach is that the analysis places a lower bound on the p-values. Due to the relatively small number of disease-associated mutations outside the seed region (typically one or two, versus 48 in a typical neighborhood) for many miRNAs, the lowest possible p-values are 4.17×10^{-2} (one mutant) or 1.57×10^{-3} (two mutants).

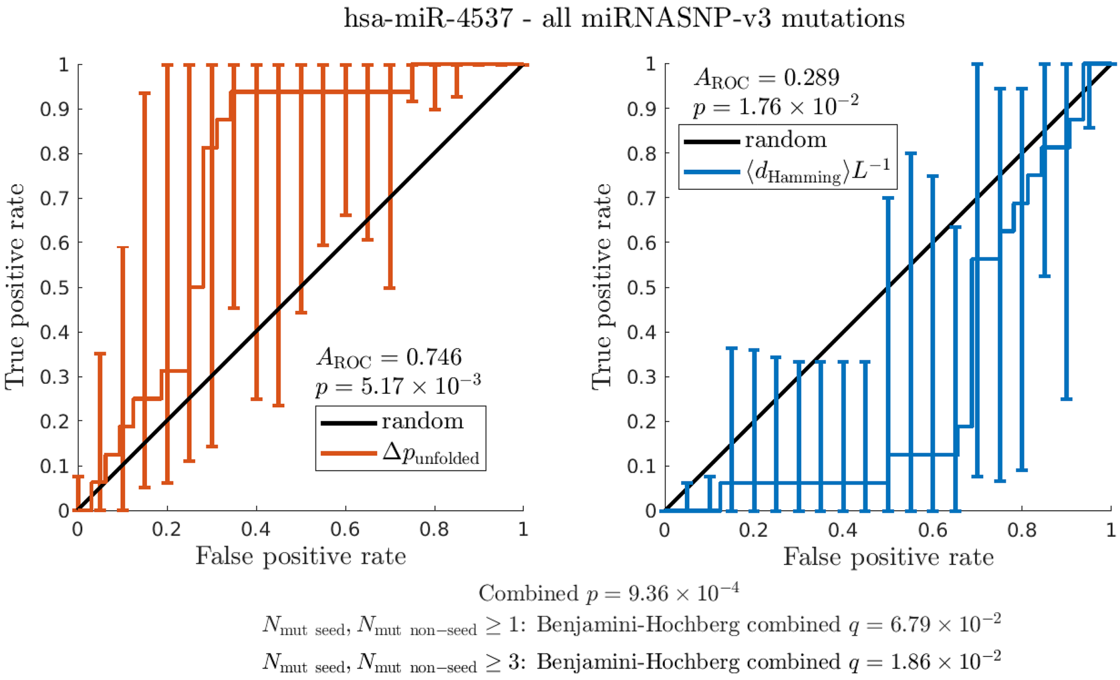

FIGURE 3 Performance of $\Delta p_{unfolded}$ and $\langle d_{Hamming} \rangle L^{-1}$ as predictors of disease association for mutations in the tumor suppressor hsa-miR-4537, which is known to be relevant to gastric cancer (60). Both of these metrics for the effect of point mutations on miRNA secondary structure perform significantly better than random when applied to the point mutational neighborhood of hsa-miR-4537. Note also that $\langle d_{Hamming} \rangle L^{-1}$ is equivalent to the percentile-based measure for aggregated data sets. As in the case of aggregated data sets with mutations for many miRNAs, mutations are ranked by these criteria, and the area under the receiver operator characteristic (A_{ROC}) is used to establish whether disease-associated mutations cluster at one end of the ranking. The pointwise 95% confidence bounds for the true positive rates, indicated by error bars, are calculated via bootstrapping and vertical averaging for 21 equally spaced values of the false positive rate from 0 to 1; see (39). The A_{ROC} values are significantly different from the value for the random criterion (0.5) for both of these metrics. This suggests that secondary structure changes can be indicative of disease association for this particular miRNA, and in agreement with this conclusion, combining these Mann-Whitney p-values via the Fisher method yields q-values (minimum positive false discovery rates) of less than 0.05 when subjected to multiple hypothesis testing; see Table 4. According to both criteria, mutations that introduce a greater change in secondary structure tend to be associated with disease. This results in $A_{ROC} < 0.5$ for $\langle d_{Hamming} \rangle L^{-1}$ because, for consistency with Table 2, we rank mutations in "ascending" order of their associated $\langle d_{Hamming} \rangle L^{-1}$; see the methods section. This means that disease-associated mutations tend to have a greater SS Hamming distance from the WT than other mutations for this individual miRNA but a smaller one for the aggregated data sets.

When we include only the higher-confidence miRNAs present in MiRGeneDB and apply the same filtering by $N_{mut\ seed}$ and $N_{mut\ non\text{-}seed} \geq 1$, we find no miRNAs for which $q < 0.05$. This is not surprising as the majority of these miRNAs are represented with only one or two mutations in miRNASNP-v3, and focusing on a smaller data set reduces the statistical power of our methods. However, to make the most of this limited data set, we introduce finer-grained filtering, requiring $N_{mut\ seed} > 0$ and $N_{mut\ non\text{-}seed} \geq 2$ or $N_{mut\ non\text{-}seed} \geq 3$. With this approach, we find q-values of less than 0.05 based on the combined p-values for $\Delta p_{unfolded}$, $\langle d_{Hamming} \rangle L^{-1}$, and $\Delta S^{(i)}$ of hsa-miR-485-5p and hsa-miR-1908-3p (see Table 5). hsa-miR-485-5p is reported to be relevant to disease, being an inhibitor of breast cancer progression according to Ref. (63). hsa-miR-1908-3p is potentially relevant to male infertility as it is reported to regulate self-renewal and apoptosis of human spermatogonial stem cells (64). We should also note that, since we perform fewer tests for individual miRNAs, a type II error—incorrectly concluding that a predictor performs significantly better than random—is less likely than when we analyze the data for all individual miRNAs present in miRNASNP-v3. Thus, although the p-values of the miRNAs that also appear in MiRGeneDB remain unchanged, their corresponding q-values are lower than when working with the full data set.

Disease-related point mutations may convert one miRNA WT into another

Interestingly, the miRNASNP-v3 database contains several point mutations that convert one miRNA WT into another—we illustrate this with two examples in Fig. 4. A striking example is the mutation of C at position 17 to G, which turns hsa-miR-3689a-3p to 3689c or 3689b-3p, the latter two having the same mature sequence. This mutation, which occurs at the site with the highest positional entropy, converts a GC Watson-Crick pair to a wobble pair and turns the relatively stable MFE fold of hsa-miR-3689a-3p to a fully unfolded structure. Position 17 is outside the seed region and the supplementary region between nt 13 and 16 that is known to contribute to target recognition for some miRNAs (9,10). Thus, the mutation probably does not affect the miRNA-mRNA target

TABLE 5 Benjamini-Hochberg *q*-values, i.e., minimum positive false discovery rates, for high-confidence miRNAs (encoded by genes present in MiRGeneDB) in which we observe a significant association between changes in secondary structure and disease after accounting for multiple hypothesis testing

	Filtering levels	
miRNA	$N_{\text{mut seed}} \geq 0, N_{\text{mut non-seed}} \geq 2$	$N_{\text{mut seed}} \geq 0, N_{\text{mut non-seed}} \geq 3$
**hsa-miR-485-5p**	5.331×10^{-2}	2.146×10^{-2} *
**hsa-miR-1908-3p**	6.516×10^{-2}	2.623×10^{-2} *

The q-values are based on combined Mann-Whitney p-values for $\Delta p_{\text{unfolded}}$, $\langle d_{\text{Hamming}} \rangle L^{-1}$ and $\Delta S^{(i)}$. Significant values ($q < 0.05$) are emphasized with an asterisk.

complementarity, and it appears likely that the drastic change in secondary structure affects miRNA activity. Moreover, the reverse mutations, which change hsa-miR-3689c or 3689b-3p to hsa-miR-3689a-3p, are also reported to be associated with disease in miRNASNP-v3. miRNAs from the hsa-mir-3689 family have been shown to be differentially expressed in conjunctival malignant melanoma, a rare form of cancer (65).

Another mutation (ID: rs138894217) that converts one miRNA into another and dramatically changes its fold is the substitution of A to G at position 13 in hsa-miR-548ar-5p, which turns it into hsa-miR-548au-5p. It turns an AU Watson pair into a GU wobble pair, making the MFE structure fully unfolded in contrast with the stably folded WT. Although mutation rs138894217 is associated with heel bone mineral density and body mass index according to miRNASNP-v3, we were not able to find information about it in the references cited therein. This points to another possible confounder in our study: some of the associations with disease that we attempt to predict here may be simply due to mistakes in the database we source them from.

A disease-associated mutation of G at site 13 in hsa-miR-1269a to A, which changes a UG wobble pair to a UA Watson-Crick pair, changes the stability of the MFE structure and turns the sequence into that of 1269b. Although the mutant and the WT have the same MFE fold, the ensemble is substantially changed, with a normalized average Hamming distance between the mutant and the WT of $\langle d_{\text{Hamming}} \rangle L^{-1} = 0.35$. Site 13 is in the supplementary region and may contribute to mRNA target recognition. This particular mutation (rs73239138) is known to be associated with various types of cancer, e.g., hepatocellular carcinoma (56–58).

We provide data on all mutations of this type that we have identified in Table S20, which contains information on the WT and mutated sequences and secondary structure, as well as the $\langle d_{\text{Hamming}} \rangle L^{-1}$ and $\Delta p_{\text{unfolded}}$ for them.

One may speculate that these point mutations subtly tune the activity of the miRNAs through secondary structure changes, but testing this hypothesis would require detailed information on the targets of each miRNA species. Moreover, as seen in Ref. (11), complementary interactions outside the canonical seed region may also contribute to target recognition. An additional complication is that not all genes encoding for these miRNA pairs are annotated as high confidence in MiRGeneDB, which makes it difficult to ascertain the significance of the pairs related through point mutations due to issues with miRNA identification, particularly false positives due to reads of fragments of other RNAs (49).

AlphaFold 3 provides tentative evidence for mature miRNA secondary structure within RISC

As we note in the introduction, the canonical view of mature miRNA function is that it is carried out within RISC, which has led to the assumption that miRNAs do not have secondary structure. However, as mentioned above, secondary structure is known to form in some RNA-protein complexes, notably in the influenza virus (28). Moreover, we found that the state-of-the-art tool for predicting ribonucleoprotein tertiary and quaternary structure tool AlphaFold 3 (66) indicates that two hsa-miR-6869-3p mutants, U12/G and C8/A, are folded when bound to Argonaute 1 and 2 proteins (protein sequences taken from PDB entries 4KXT and 4F3T, respectively). We show the predicted structure for one of them alongside a PDB crystal structure of a miRNA-Argonaute 2 complex in Fig. 5; we show the equivalent for the miRNA-Argonaute 1 complex in the supporting material (Fig. S23).

Although AlphaFold 3 and other machine-learning algorithms for predicting the structure of ribonucleoproteins are useful tools, their development is at a relatively early stage (67), and their accuracy and applicability are limited by the relatively low coverage of RNA structures in databases such as PDB (see, e.g., (68)). An additional drawback is that the generative AI model underpinning AlphaFold 3 tends to predict structure even in unstructured protein regions (66). The predictions for Argonaute in complex with the C8/A mutant of hsa-miR-6869-3p also included a low-confidence helix in the protein, which is unlikely to be present in the real structure. For these reasons and due to the large computational expense of folding the tens of thousands of mutants we study in our work with a tool such as AlphaFold 3, we restrict our analysis to mature miRNA secondary structure. miRNA folding within the RISC complex is probably indeed restricted as per the canonical picture, but miRNA secondary structure may still be relevant to disease through the many non-canonical and poorly studied alternative miRNA pathways (31,32).

Additionally, we predicted the tertiary structure of complexes between Argonaute 2 and the WTs or the top-scoring mutants of the miRNAs that show significant association between secondary structure changes and disease and are not ruled out by multiple hypothesis testing. Out of these, only the hsa-miR-4537-3p mutant with maximum $\Delta p_{\text{unfolded}}$ was predicted to have a nontrivial fold, but with a much lower confidence (average plDDT: 65.3) than the mutant in hsa-miR-6869-3p shown in Fig. 5 (average plDDT: 76.4). We provide tables of the predicted secondary structure and CIF files with the tertiary structures as part of the supporting material.

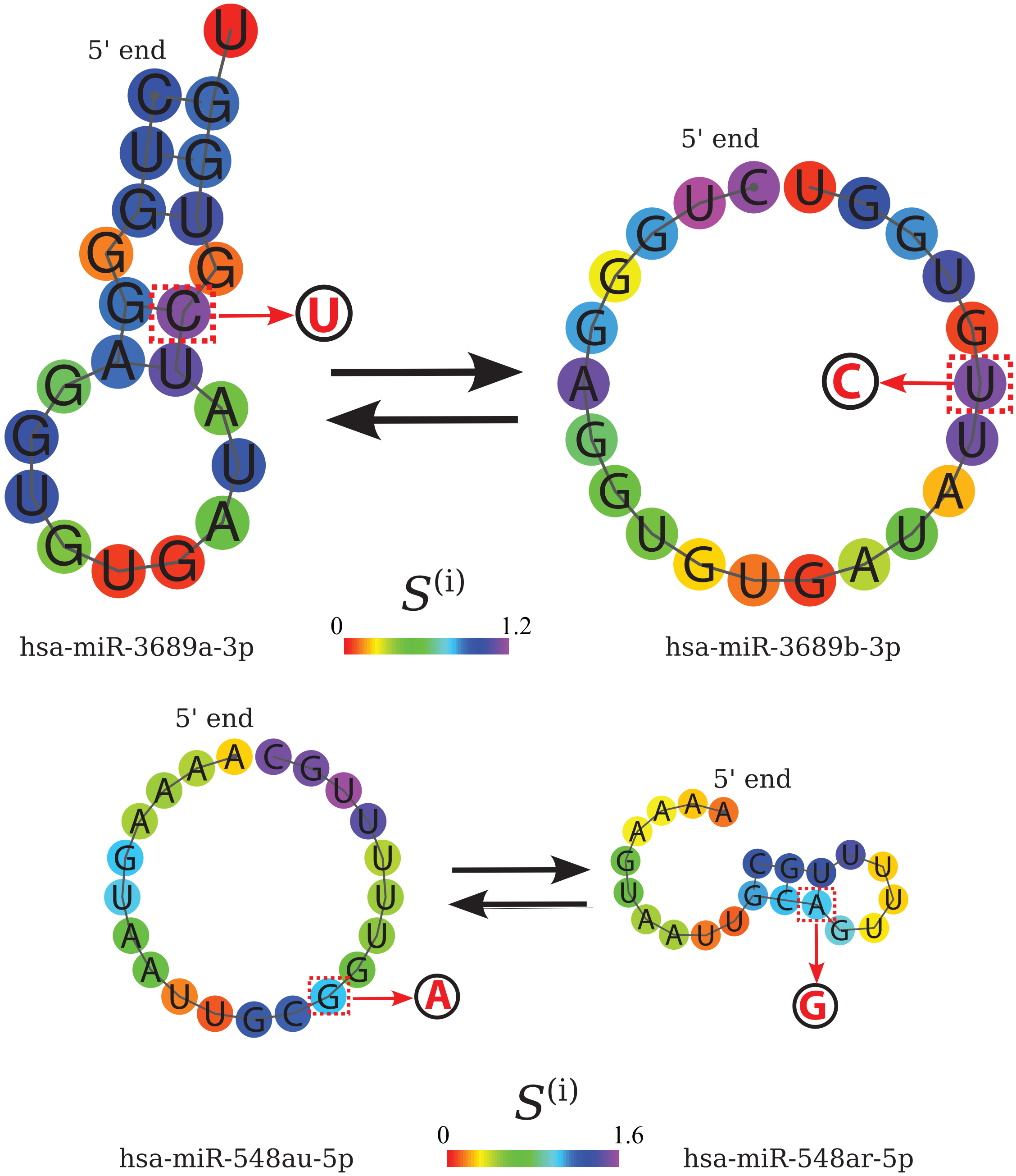

FIGURE 4 Single-point substitution mutations that convert one miRNA to another. Top: substituting the C in position 17, which has the highest predicted positional entropy (*see color and colorbar*) in the MFE structure, changes hsa-miR-3689a-3p into hsa-miR-3689b-3p. As the mutation converts a Watson-Crick GC pair into a GU wobble pair, the MFE structure changes from stably folded to fully unfolded. Both the forward and the reverse mutations are associated with disease in miRNASNP-v3. Bottom: substitution of G in position 13 with A creates an AU Watson-Crick pair, changing the MFE structure from fully unfolded to stably folded. These MFE predictions and visualizations are based on tools from the ViennaRNA suite (37).

Some disease-related miRNA mutations outside the seed region change the fold of the mature form but not the pre-miRNA

It is possible that mutations in mature miRNA sequences affect function through altering the secondary structure of the precursor, rather than the mature miRNA itself. We study this possibility through generating predictions for the secondary structure of the pre-miRNAs of all disease-related mature miRNA mutant sequences that we considered in our study. We then characterize the differences between those mutated pre-miRNAs and their respective WTs. First, we calculate the average Hamming distance in secondary structure between mutant and WT. Comparing the distributions, which we plot in Fig. S21 in the supporting material, shows that they do not differ significantly (two-sided Mann-Whitney U test: $p =$

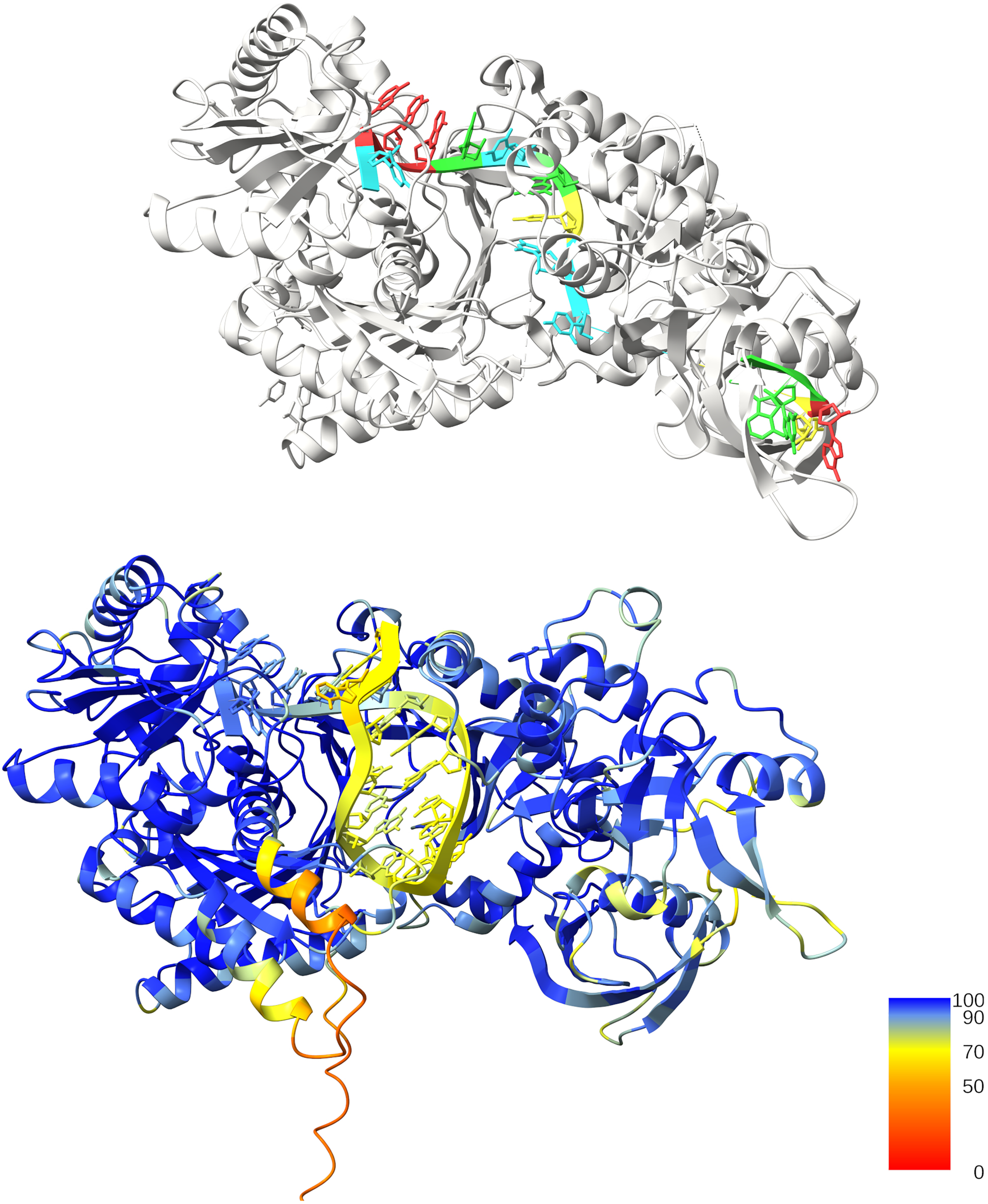

FIGURE 5 AlphaFold 3 predicts non-trivial secondary structure in the complex of a point mutant of hsa-miR-6869-3p and Argonaute 2. Crystal structure of human Argonaute-2 protein in complex with miR-20a (PDB: 4F3T, *top*) and AlphaFold 3 predictions for the non-seed point mutant of hsa-miR-6869-3p with the maximum $\langle d_{\text{Hamming mutant}} \rangle$ in complex with human Argonaute-2 (*bottom*) are shown. RNA is depicted as ribbons with bases indicated; note the base-pairing in the predicted structure. All residues in the AlphaFold 3 image are colored by plDDT, a measure of the confidence of the prediction, as indicated by the color bars. Most of the miRNA structure is predicted with high confidence ($70 < \text{plDDT} < 90$, average: 76.4), and the predictions show base-pairing within the miRNA. The crystal structure shows no structural information about nucleotides 11 to 16. Images were created with UCSF ChimeraX version 1.09 (69).

0.33 for miRNASNP3), which indicates that disease-associated point mutations induce comparable absolute secondary structure changes in mature and precursor miRNAs.

In addition, we calculate how the disease-related mutations affect the predicted base-pairing probabilities changed within the pre-miRNA regions that other authors have identified as important to pre-miRNA processing (70). Roden et al. found that the region at distance 5–9 nt from the base of the pre-miRNA hairpin tends to be enriched with bulges, whereas the regions at distance 16–21 nt and 28–32 nt tend to be bulge-depleted. We calculate how each disease-related mutation changes the probability that each of these positions is paired with respect to the pre-miRNA WT. We then calculate the maximum absolute value of this change, which highlights whether the pairing of any individual site has been strongly affected. This is a useful measure as bulges in individual sites have previously been reported to cause disease; see the examples of variants in miR-502 (19) and miR-125a (20) that we refer to in the introduction. Together with the maximum change of base-pairing probability for the pre-miRNA sites sensitive to secondary structure variations identified by Roden et al., we calculate the same across all sites within the mature miRNA mutants.

We then subtract the maximum local change in base-pairing probability in each pre-miRNA mutant from that of the respective mature mutant. This difference, which we denote Δ_{max}, is 1 if a pre-miRNA mutation changes one or more sites from completely paired to completely unpaired or vice versa, but the same mutation leaves all sites in the mature form unchanged. Analogously, $\Delta_{max} = -1$ if one or more sites are changed from completely paired to completely unpaired in the mature form but are unchanged in the pre-miRNA. The distribution of Δ_{max} for miRNASNP3 data, which we plot in Fig. S22 in the supporting material, contains a number of mutations that induce large changes in the pairing of sites in the mature form but practically no change in the pre-miRNA. 219 such mutants have $\Delta_{max} \leq -0.5$, and 39 have $\Delta_{max} \leq -0.9$. This means that some disease-related mutants do not affect the secondary structure at sites important for pre-miRNA processing but only change the folding of the mature form, suggesting that mature miRNA secondary structure also plays a role in the diseases covered by miRNASNP3 data.

DISCUSSION

This computational study explores the role of secondary structure in mature miRNA mutants based on literature data for associations between such mutants and disease. We formulate different quantitative measures for the difference between a given mutant and its respective WT and test their ability to predict disease association.

Our results for the data in miRNASNP-v3 indicate a significant association between disease and mutations that increase the probability that a given mature miRNA is unfolded. This is in contrast to the work of Diederichs and Haber (17), who found that mutations that significantly changed pri-miRNA secondary structure had no effect on their processing and maturation. Moreover, we see a significant effect of the change in the pairing of mutated sites across the miRNASNP-v3 database as measured via the positional entropy difference at the mutated site, $\Delta S^{(i)}$. When we apply our criteria for mutation-induced secondary structure changes to the data for individual miRNAs, we observe a particularly strong relationship between secondary structure and disease for several miRNAs. This analysis combines p-values for the $\Delta pun_{\mathrm{unfolded}}$ and $\langle d_{\mathrm{Hamming}} \rangle / L$ metrics using the Fisher method and employs the Benjamini-Hochberg method for multiple hypothesis testing. We furthermore filter the data according to the number of mutations recorded in the seed region and outside the seed region. The miRNAs that emerge are hsa-miR-1269b, hsa-miR-4537, hsa-miR-4477b, hsa-miR-4641, and hsa-miR-6821-3p, which include three miRNAs that are known to play a role in cancers (56–60).

We address the issue of possible misidentification of other RNAs as miRNAs by performing the same analysis on the subset of mutations from miRNASNP-v3 that concern miRNAs appearing in the manually curated database MiRGeneDB 2.1 (51). Despite the reduced statistical power due to the smaller size of the data set, one of our measures for characterizing the effect of secondary structure is again significantly associated with disease. On the level of individual miRNAs, in this case, we identify that secondary structure changes are associated with disease in the miRNAs hsa-miR-485-5p and hsa-miR-1908-3p.

We need to emphasize that, although we aim to exclude clearly incorrect entries by performing cross-checks between data from mutation databases such as miRNASNP and reference data sets such as miRBase, HG38, and MiRGeneDB, it is not our aim here to curate a data set of mutations in miRNAs or to probe the association of these mutations with disease. Instead, we have devised a method that, given a set of such disease-associated mutations, characterizes whether they tend to be associated with changes in secondary structure. Although an imperfect approach, this addresses our aim of characterizing the effect of mature miRNA secondary structure on miRNA activity. It would also be straightforward to apply the algorithms we have formulated here to a better-curated data set with miRNA mutations should one become available in the future.

It is likely that the significance criteria are not met for many other miRNAs because of a combination of factors: 1) the small number of mutations recorded for most miRNAs and 2) the different nature of the mutations in the database. Most of the disease-associated mutations in miRNASNP-v3 have been identified via methods such as genome-wide association studies, which do not establish a causative relationship with disease, meaning that some of these mutations are likely to simply accompany disease. Another possible

confounder in our current study are mistakes in the assignment of disease association to mutations, of which we give an example in the previous section (SNP rs138894217).

Quantitative experimental data on the activity of various point mutants of a set of miRNAs would shed light on these matters. Attempts at quantifying the effect of mutations in non-coding RNAs, including for the case of such mutations that reduce the risk of disease, are collected in the ncRNA-Var database by Zhang et al. (71). This database uses weighted scores to combine different forms of evidence in the literature rather than quantitative experimental evidence, and it covers a smaller number of mutations than miRNASNP-v3.

CONCLUSION

As we note in the introduction, it is difficult to establish the role of mature miRNA secondary structure because the mature form of the molecule is typically incorporated in RISC, and furthermore, even mutations in the mature sequence could affect miRNA function through changing the folding and maturation of its precursor, rather than through changing the secondary structure of the mature miRNA itself; see the example of miRNA-125a in Ref. (20) and others in Ref. (72). Moreover, the same mutation can simultaneously be associated with a decreased risk of one disease and an increased risk of another, as in the example of the rs291016 polymorphism (21) that we gave in the introduction. This is likely a consequence of the complex networks of genes regulated by each miRNA, as well as potential non-canonical pathways for some of them. The fact that some of our measures of secondary structure change perform better than random may be due to different mechanisms of miRNA activity that depend on specific secondary structure features. At the same time, there is uncertainty in our conclusions that is inevitable given that the data from mutation databases include multiple possible confounders, as we discuss above.

With all this in mind, a definitive test of our hypothesis that miRNA secondary structure can have significant influence on function requires experimental data on the regulatory activity of mutants, preferably in the form of gene suppression activity for the point mutants derived from several mature miRNAs (with the seed region fixed). Based on our analysis, hsa-miR-485-5p and hsa-miR-1908-3p are the most promising candidates for such studies. The availability of such experimental data would allow for the further refinement of our methods for predicting which mutations are associated with disease, particularly cancer, and thus yield a potentially valuable diagnostic tool.

ACKNOWLEDGMENTS

This work was supported by the Isaac Newton Trust (NQAG/341) and was performed using resources provided by the Cambridge Service for Data Driven Discovery (CSD3) operated by the University of Cambridge Research Computing Service (www.csd3.cam.ac.uk), provided by Dell EMC and Intel using Tier-2 funding from the Engineering and Physical Sciences Research Council (capital grant EP/T022159/1), and DiRAC funding from the Science and Technology Facilities Council (www.dirac.ac.uk). J. K.N. acknowledges funding from the MRC (grant MC_FE_00035) Cross Disciplinary Fellowship (XDF) Program. A preliminary version of this work, https://doi.org/10.1101/2024.06.19.599688, was deposited in biorXiv on June 22nd, 2024. For the purpose of open access, the authors have applied a Creative Commons Attribution (CC BY) license to any Author Accepted Manuscript version arising from this submission.

AUTHOR CONTRIBUTIONS

J.K.N.: conceptualization, software, data curation, formal analysis, investigation, methodology, writing—original draft, and writing—review and editing; S.E.A.: funding acquisition, conceptualization, software, formal analysis, methodology, and writing—review and editing.

DECLARATION OF INTERESTS

The authors declare no competing interests.

SUPPORTING MATERIAL

Supporting material can be found online at https://doi.org/10.1016/j.bpj.2025.09.049. We provide the set of Python, Bash and MATLAB scripts that we have used to generate all the results in the manuscript. We also provide the results from our computational predictions of the properties of secondary structure for all studied point mutants and tertiary structure predictions for selected miRNAs in complex with Argonaute proteins. Code and data available at https://doi.org/10.6084/m9.figshare.26490985.v1.

REFERENCES

1. Oulas, A., N. Karathanasis, …, I. Iliopoulos. 2015. Prediction of miRNA Targets. Humana Press, pp. 207–229. http://link.springer.com/10.1007/978-1-4939-2291-8_13.
2. Wu, M., N. Jolicoeur, …, S.-H. Shen. 2008. Genetic variations of microRNAs in human cancer and their effects on the expression of miRNAs. *Carcinogenesis.* 29:1710–1716. https://academic.oup.com/carcin/article-lookup/doi/10.1093/carcin/bgn073.
3. Iorio, M. V., and C. M. Croce. 2012. MicroRNA dysregulation in cancer: Diagnostics, monitoring and therapeutics. A comprehensive review. *EMBO Mol. Med.* 4:143–159.
4. Gebert, L. F. R., and I. J. MacRae. 2019. Regulation of microRNA function in animals. *Nat. Rev. Mol. Cell Biol.* 20:21–37.
5. Lin, S., and R. I. Gregory. 2015. MicroRNA biogenesis pathways in cancer. *Nat. Rev. Cancer.* 15:321–333. http://www.nature.com/articles/nrc3932.
6. Bracken, C. P., H. S. Scott, and G. J. Goodall. 2016. A network-biology perspective of microRNA function and dysfunction in cancer. *Nat. Rev. Genet.* 17:719–732.
7. Ørom, U. A., F. C. Nielsen, and A. H. Lund. 2008. MicroRNA-10a Binds the 5’UTR of Ribosomal Protein mRNAs and Enhances Their Translation. *Mol. Cell.* 30:460–471.
8. Winter, J., and S. Diederichs. 2011. MicroRNA Biogenesis and Cancer. Springer, pp. 3–22. http://link.springer.com/10.1007/978-1-60761-863-8_1.
9. Broughton, J. P., M. T. Lovci, …, A. E. Pasquinelli. 2016. Pairing beyond the Seed Supports MicroRNA Targeting Specificity. *Mol. Cell.* 64:320–333. https://doi.org/10.1016/j.molcel.2016.09.004.

10. Brennecke, J., A. Stark, …, S. M. Cohen. 2005. Principles of microRNA-target recognition. *PLoS Biol.* 3:e85–e418.

11. Helwak, A., G. Kudla, …, D. Tollervey. 2013. Mapping the human miRNA interactome by CLASH reveals frequent noncanonical binding. *Cell.* 153:654–665. https://doi.org/10.1016/j.cell.2013.03.043.

12. Backes, C., E. Meese, and A. Keller. 2016. Specific miRNA Disease Biomarkers in Blood, Serum and Plasma: Challenges and Prospects. *Mol. Diagn. Ther.* 20:509–518.

13. Jansson, M. D., and A. H. Lund. 2012. MicroRNA and cancer. *Mol. Oncol.* 6:590–610. https://doi.org/10.1016/j.molonc.2012.09.006.

14. Urbanek-Trzeciak, M. O., P. Galka-Marciniak, …, P. Kozlowski. 2020. Pan-cancer analysis of somatic mutations in miRNA genes. *EBioMedicine.* 61:103051. https://linkinghub.elsevier.com/retrieve/pii/S2352396420304278.

15. Li, T., H. Pan, and R. Li. 2016. The dual regulatory role of miR-204 in cancer. *Tumour Biol.* 37:11667–11677. http://link.springer.com/10.1007/s13277-016-5144-5.

16. Gebeshuber, C. A., K. Zatloukal, and J. Martinez. 2009. miR-29a suppresses tristetraprolin, which is a regulator of epithelial polarity and metastasis. *EMBO Rep.* 10:400–405. https://www.embopress.org/doi/10.1038/embor.2009.9.

17. Diederichs, S., and D. A. Haber. 2006. Sequence variations of microRNAs in human cancer: Alterations in predicted secondary structure do not affect processing. *Cancer Res.* 66:6097–6104.

18. Imran, M., T. Liu, …, M. Zhang. 2022. Nested miRNA Secondary Structure Is a Unique Determinant of miR159 Efficacy in Arabidopsis. *Front. Plant Sci.* 13:905264. https://www.frontiersin.org/articles/10.3389/fpls.2022.905264/full.

19. Sun, G., J. Yan, …, J. J. Rossi. 2009. SNPs in human miRNA genes affect biogenesis and function. *RNA.* 15:1640–1651.

20. Duan, R., C. Pak, and P. Jin. 2007. Single nucleotide polymorphism associated with mature miR-125a alters the processing of pri-miRNA. *Hum. Mol. Genet.* 16:1124–1131. http://academic.oup.com/hmg/article/16/9/1124/688755/Single-nucleotide-polymorphism-associated-with.

21. Tüfekci, K. U., M. G. Öner, …, Ş. Genç. 2014. The Role of MicroRNAs in Human Diseases. *Humana Press.* 1107:33–50. http://link.springer.com/10.1007/978-1-62703-748-8_3.

22. Ergin, K., and R. Çetinkaya. 2022. Regulation of MicroRNAs1107, Second edition. Humana Press, pp. 1–32. https://link.springer.com/10.1007/978-1-0716-1170-8_1.

23. Kawamata, T., H. Seitz, and Y. Tomari. 2009. Structural determinants of miRNAs for RISC loading and slicer-independent unwinding. *Nat. Struct. Mol. Biol.* 16:953–960.

24. Elkayam, E., C.-D. Kuhn, …, L. Joshua-Tor. 2012. The Structure of Human Argonaute-2 in Complex with miR-20a. *Cell.* 150:100–110. https://linkinghub.elsevier.com/retrieve/pii/S0092867412006186.

25. Noller, H. F., J. Kop, …, C. R. Waese. 1981. Secondary structure model for 23S ribosomal RNA. *Nucleic Acids Res.* 9:6167–6189.

26. Eisfeld, A. J., G. Neumann, and Y. Kawaoka. 2015. At the centre: influenza A virus ribonucleoproteins. *Nat. Rev. Microbiol.* 13:28–41.

27. Kobayashi, Y., B. Dadonaite, …, O. G. Pybus. 2016. Computational and molecular analysis of conserved influenza A virus RNA secondary structures involved in infectious virion production. *RNA Biol.* 13:883–894.

28. Dawson, W. K., M. Lazniewski, and D. Plewczynski. 2017. RNA structure interactions and ribonucleoprotein processes of the influenza A virus. *Brief. Funct. Genomics.* 17:402–414. https://doi.org/10.1093/bfgp/elx028.

29. Belter, A., D. Gudanis, …, J. Barciszewski. 2014. Mature MiRNAs Form Secondary Structure, which Suggests Their Function beyond RISC. *PLoS One.* 9:e113848. https://dx.plos.org/10.1371/journal.pone.0113848.

30. Rolle, K., M. Piwecka, …, J. Barciszewski. 2016. The Sequence and Structure Determine the Function of Mature Human miRNAs. *PLoS One.* 11:e0151246.

31. Santovito, D., and C. Weber. 2022. Non-canonical features of microRNAs: paradigms emerging from cardiovascular disease. *Nat. Rev. Cardiol.* 19:620–638.

32. Makarova, J. A., M. U. Shkurnikov, …, A. G. Tonevitsky. 2016. Intracellular and extracellular microRNA: An update on localization and biological role. *Prog. Histochem. Cytochem.* 51:33–49.

33. Flores, O., E. M. Kennedy, …, B. R. Cullen. 2014. Differential RISC association of endogenous human microRNAs predicts their inhibitory potential. *Nucleic Acids Res.* 42:4629–4639.

34. Stalder, L., W. Heusermann, …, N. C. Meisner-Kober. 2013. The rough endoplasmatic reticulum is a central nucleation site of siRNA-mediated RNA silencing. *EMBO J.* 32:1115–1127.

35. Borel, C., and S. E. Antonarakis. 2008. Functional genetic variation of human miRNAs and phenotypic consequences. *Mamm. Genome.* 19:503–509.

36. Cammaerts, S., M. Strazisar, …, P. De Rijk. 2016. MiRVaS: A tool to predict the impact of genetic variants on miRNAs. *Nucleic Acids Res.* 44:e23.

37. Lorenz, R., S. H. Bernhart, …, I. L. Hofacker. 2011. ViennaRNA Package 2.0. *Algorithm Mol. Biol.* 6:26. https://almob.biomedcentral.com/articles/10.1186/1748-7188-6-26.

38. Mathworks. 2022. Wilcoxon rank sum test - MATLAB ranksum - MathWorks United Kingdom. https://uk.mathworks.com/help/stats/ranksum.html.

39. Mathworks. 2022. Receiver operating characteristic (ROC) curve or other performance curve for classifier output - MATLAB perfcurve - MathWorks United Kingdom. https://uk.mathworks.com/help/stats/perfcurve.html.

40. Hofacker, I. L. 2003. RNA Secondary Structure Analysis Using the ViennaRNA Package. *Curr. Protoc. Bioinformatics.* 4:1–12. https://onlinelibrary.wiley.com/doi/10.1002/0471250953.bi1202s04.

41. Hofacker, I. L. 2014. Energy-Directed RNA Structure Prediction. Humana Press, pp. 71–84.

42. Dingle, K., F. Ghaddar, …, A. A. Louis. 2022. Phenotype Bias Determines How Natural RNA Structures Occupy the Morphospace of All Possible Shapes. *Mol. Biol. Evol.* 39:1–11. https://academic.oup.com/mbe/article/doi/10.1093/molbev/msab280/6372700.

43. Gruber, A. R., R. Lorenz, …, I. L. Hofacker. 2008. The Vienna RNA websuite. *Nucleic Acids Res.* 36:70–74.

44. Mason, S. J., and N. E. Graham. 2002. Areas beneath the relative operating characteristics (ROC) and relative operating levels (ROL) curves: Statistical significance and interpretation. *Q. J. R. Meteorol. Soc.* 128:2145–2166.

45. Lu, M., Q. Zhang, …, Q. Cui. 2008. An analysis of human microRNA and disease associations. *PLoS One.* 3:e3420–e3425.

46. Liu, C.-J., X. Fu, …, A.-Y. Guo. 2021. miRNASNP-v3: a comprehensive database for SNPs and disease-related variations in miRNAs and miRNA targets. *Nucleic Acids Res.* 49:D1276–D1281. https://academic.oup.com/nar/article/49/D1/D1276/5912820.

47. Bhattacharya, A., and Y. Cui. 2016. SomamiR 2.0: a database of cancer somatic mutations altering microRNA–ceRNA interactions. *Nucleic Acids Res.* 44:D1005–D1010. https://academic.oup.com/nar/article-lookup/doi/10.1093/nar/gkv1220.

48. Fehlmann, T., S. Sahay, …, C. Backes. 2019. A review of databases predicting the effects of SNPs in miRNA genes or miRNA-binding sites. *Brief Bioinformatics.* 20:1011–1020. https://academic.oup.com/bib/article/20/3/1011/4665691.

49. Kozomara, A., M. Birgaoanu, and S. Griffiths-Jones. 2019. MiRBase: From microRNA sequences to function. *Nucleic Acids Res.* 47:D155–D162.

50. The Genome Reference Consortium. https://www.ncbi.nlm.nih.gov/grc.

51. Fromm, B., E. Høye, …, K. J. Peterson. 2022. MirGeneDB 2.1: Toward a complete sampling of all major animal phyla. *Nucleic Acids Res.* 50:D204–D210.

52. Brown, M. B. 1975. 400: A Method for Combining Non-Independent, One-Sided Tests of Significance. *Biometrics.* 31:987. https://www.jstor.org/stable/2529826?origin=crossref.

53. Pfister, R., K. A. Schwarz, …, J. B. Freeman. 2013. Good things peak in pairs: a note on the bimodality coefficient. *Front. Psychol.* 4:700–797. http://journal.frontiersin.org/article/10.3389/fpsyg.2013.00700/abstract.

54. Freeman, J. B., and R. Dale. 2013. Assessing bimodality to detect the presence of a dual cognitive process. *Behav. Res. Methods.* 45:83–97.

55. Mathworks. 2023. Estimate positive false discovery rate for multiple hypothesis testing - MATLAB mafdr - MathWorks United Kingdom. https://uk.mathworks.com/help/bioinfo/ref/mafdr.html.

56. Torruella-Loran, I., H. Laayouni, …, Y. Espinosa-Parrilla. 2016. MicroRNA Genetic Variation: From Population Analysis to Functional Implications of Three Allele Variants Associated with Cancer. *Hum. Mutat.* 37:1060–1073. https://onlinelibrary.wiley.com/doi/10.1002/humu.23045.

57. Min, P., W. Li, …, M. Zhang. 2017. A single nucleotide variant in microRNA-1269a promotes the occurrence and process of hepatocellular carcinoma by targeting to oncogenes SPATS2L and LRP6. *Bull. Cancer.* 104:311–320. https://doi.org/10.1016/j.bulcan.2016.11.021.

58. Villegas-Mirón, P., A. Gallego, …, Y. Espinosa-Parrilla. 2022. Signatures of genetic variation in human microRNAs point to processes of positive selection and population-specific disease risks. *Hum. Genet.* 141:1673–1693. https://doi.org/10.1007/s00439-021-02423-8.

59. He, X., G. Cheng, …, X. Song. 2021. miR-4477b gene as a novel pathogenic mutation occurring during the transformation of colorectal adenoma into colorectal cancer. *J. Gastrointest. Oncol.* 12:69–78. https://jgo.amegroups.com/article/view/49353/html.

60. Liu, J., S. Yan, …, Y. Liu. 2021. MiRNA-4537 functions as a tumor suppressor in gastric cancer and increases the radiosensitivity of gastric cancer cells. *Bioengineered.* 12:8457–8467.

61. Budd, E., G. Nalesso, and A. Mobasheri. 2018. Extracellular genomic biomarkers of osteoarthritis. *Expert Rev. Mol. Diagn.* 18:55–74. https://doi.org/10.1080/14737159.2018.1415757.

62. Yasui, T., T. Yanagida, …, Y. Baba. 2017. Unveiling massive numbers of cancer-related urinary-microRNA candidates via nanowires. *Sci. Adv.* 3:e1701133. https://www.science.org/doi/10.1126/sciadv.1701133.

63. Wang, X., X. Zhou, …, H. Li. 2020. miR-485-5p inhibits the progression of breast cancer cells by negatively regulating MUC1. *Breast Cancer.* 27:765–775. https://doi.org/10.1007/s12282-020-01075-2.

64. Chen, X., S. Zhang, …, N. Li. 2020. MiR-485-5p Promotes Neuron Survival through Mediating Rac1/Notch2 Signaling Pathway after Cerebral Ischemia/Reperfusion. *Curr. Neurovascular Res.* 17:259–266. https://www.eurekaselect.com/180920/article.

65. Larsen, A. C. 2016. Conjunctival malignant melanoma in Denmark: epidemiology, treatment and prognosis with special emphasis on tumorigenesis and genetic profile. *Acta Ophthalmol.* 94:1–27. https://onlinelibrary.wiley.com/doi/10.1111/aos.13100.

66. Abramson, J., J. Adler, …, J. M. Jumper. 2024. Accurate structure prediction of biomolecular interactions with AlphaFold 3. *Nature.* 630:493–500.

67. Bernard, C., G. Postic, …, F. Tahi. 2025. Has AlphaFold 3 achieved success for RNA? *Acta Crystallogr. D Struct. Biol.* 81:49–62.

68. Wang, W., C. Feng, …, J. Yang. 2023. trRosettaRNA: automated prediction of RNA 3D structure with transformer network. *Nat. Commun.* 14:7266.

69. Meng, E. C., T. D. Goddard, …, T. E. Ferrin. 2023. UCSF ChimeraX : Tools for structure building and analysis. *Protein Sci.* 32:e4792.

70. Roden, C., J. Gaillard, …, J. Lu. 2017. Novel determinants of mammalian primary microRNA processing revealed by systematic evaluation of hairpin-containing transcripts and human genetic variation. *Genome Res.* 27:374–384.

71. Zhang, W., B. Zeng, …, W. Li. 2021. ncRNAVar: A Manually Curated Database for Identification of Noncoding RNA Variants Associated with Human Diseases. *J. Mol. Biol.* 433:166727.

72. de Carvalho, J. B., G. L. de Morais, …, A. T. R. de Vasconcelos. 2019. miRNA Genetic Variants Alter Their Secondary Structure and Expression in Patients With RASopathies Syndromes. *Front. Genet.* 10:1144.

Biophysical Journal, Volume *124*

Supplemental information

Disease-related miRNA mutations are associated with mature miRNA secondary structure changes

Javor K. Novev and Sebastian E. Ahnert

Supplementary Information to Disease-related miRNA mutations are associated with mature miRNA secondary structure changes

Javor K. Novev*[1,2] and Sebastian E. Ahnert†[1,3]

[1]Department of Chemical Engineering and Biotechnology, University of Cambridge, Philippa Fawcett Drive, Cambridge CB3 0AS, UK
[2]Institute of Genetics and Cancer, The University of Edinburgh, Western General Hospital, Crewe Road, Edinburgh EH4 2XU, UK
[3]The Alan Turing Institute, 96 Euston Road, London NW1 2DB, UK

Contents

1 SomamiR database **2**

2 Additional metrics of the effect of mutations on secondary structure **2**
2.1 Change in the probability of the unfolded states with respect to the WT 3
2.1.1 Change in the probability of the fully unfolded state with respect to the WT 3
2.1.2 Change in the probability that the seed region is unfolded with respect to the WT . . 3
2.2 Hamming-distance-based criteria 3
2.2.1 Normalized Hamming distance from the WT, $\langle d_{\mathrm{Hamming}}\rangle L^{-1}$ 3
2.2.2 $\langle d_{\mathrm{Hamming}}\rangle$ percentile 3
2.3 Positional-entropy-based criteria 6
2.3.1 Change of the positional entropy of the mutated site with respect to the WT 6
2.3.2 Mutant average positional entropy 6
2.3.3 Change of the average positional entropy with respect to the WT 6

3 Tables and graphical comparisons of measures of criteria performance **25**

4 Rank correlations between selected criteria **39**

5 Tables of p-values and other data for individual miRNAs **40**

6 Table of disease-associated mutations that convert one WT miRNA to another **45**

7 Case studies **46**
7.1 hsa-miR-4537 46
7.2 hsa-miR-485-5p 46

8 Additional information on miRNAs with $q < 0.05$ **49**

*Corresponding author: ynovev@ed.ac.uk
†Corresponding author: sea31@cam.ac.uk

9 Distributions of $\langle d_{\mathrm{Hamming}} \rangle$ for disease-related mutations in mature miRNAs and their precursors **52**

10 Distributions of $\Delta_{\max}$ for disease-related mutations **53**

11 AlphaFold3 studies of miRNA-Argonaute complexes **54**

12 Code and data **57**

Here we provide information on the criteria for relationship with disease that we only mentioned in the main text, additional ROC curves for the different predictors, plots that compare A_{ROC} for different criteria under different levels of filtering, and tables with A_{ROC} and p-values and sample sizes.

1 SomamiR database

In addition to the miRNASNP-v3 dataset discussed in the main text, we evaluate the performance of our metrics on the SomamiR dataset of miRNA mutations encountered in cancer [1]. After cross-checking the SomamiR data with HG38 and miRBase, the number of mature miRNA mutations outside the seed recorded in it is 439, with 363 unique sequences derived from 285 WT miRNAs, and the number of seed-region mutations is 173. Filtering to include only miRNAs with $N_{\mathrm{mut\ seed}} \geq 1$ and $N_{\mathrm{mut\ non-seed}} \geq 1$ leaves 81 mutations outside the seed region, representing 66 unique sequences and affecting 40 miRNAs.

2 Additional metrics of the effect of mutations on secondary structure

We formulated several other criteria that measure the effect of non-seed point mutations on miRNA secondary structure, and give a list of them below. We do not describe them or give details on their performance in the main text since the latter is inferior to that of the three we discuss therein.

a) The change in the probability that the seed region is fully unfolded, $\Delta p_{\mathrm{unfolded\ seed}} = p_{\mathrm{unfolded\ seed\ mutant}} - p_{\mathrm{unfolded\ seed\ WT}}$. As the seed is key for target recognition, one may expect that a high value of $\Delta p_{\mathrm{unfolded\ seed}}$ would strongly affect the activity of a miRNA.

b) The average positional entropy of a mutant, $\langle S_{\mathrm{mut}} \rangle$, which measures the stability of a fold. We defined additional criteria related to $\langle S_{\mathrm{mut}} \rangle$ as follows.

– The absolute mutant positional entropy, $\langle S_{\mathrm{mut}} \rangle$.

– The difference in positional entropy between mutant and WT, $\langle \Delta S \rangle = \langle S_{\mathrm{mut}} \rangle - \langle S_{\mathrm{WT}} \rangle$. We apply this criterion in two different ways: **i)** we test how well it predicts association with disease for data for all miRNAs in a dataset or **ii)** by splitting datasets into subsets where the SS stability of mutant and WT can be compared via $\langle \Delta S \rangle$ because the subset only includes mutants and WTs that are **a)** both folded or **b)** both unfolded.

c) Tree editing distance d_{tree} as calculated by the RNAdistance program from the ViennaRNA suite [2]. Calculating the average of d_{tree} over the Boltzmann ensemble is too costly since we examine a set of ~ 500 secondary structures for each studied sequence. This requires $\sim 500^2$ comparisons between each pair of WT and mutant; when applied to the set of $\sim 4 \times 10^4$ mutants we consider, the computational cost of this would be orders of magnitude greater than that for conducting the rest of the study.

Due to the computational cost of calculating the average value of this distance across mutant and WT thermodynamic ensembles, we compute only the distance between the MFE structure of each mutant and WT and normalize it by its maximum possible value, $d_{\mathrm{tree}} = d_{\mathrm{RNAdistance}}/(2L)$.

In building the ROC curves and analyzing the performance of these criteria, we use the same approach described above for $\Delta p_{\text{unfolded}}$ - we calculate the criterion values for a set of mutants, then rank the latter in *descending order* according to that criterion. Next, we ascertain whether the criterion predicts association with disease better than random by building a ROC curve, calculating the area under it (A_{ROC}) and checking whether the associated p-values meet our significance criteria. We provide more details on the criteria and their performance below.

2.1 Change in the probability of the unfolded states with respect to the WT

2.1.1 Change in the probability of the fully unfolded state with respect to the WT

Additional ROC curves for $\Delta p_{\text{unfolded}} = p_{\text{unfolded mutant}} - p_{\text{unfolded WT}}$ are shown in Figures S1-S2.

2.1.2 Change in the probability that the seed region is unfolded with respect to the WT

As the miRNA seed region is crucial to miRNA-mRNA binding, we expect that mutations which change its folding have a significance in disease. Specifically, we test whether disease-related mutations change the probability that the seed region contains no base-pairing more than other possible point mutations. To calculate the change in probability for no seed-region base-pairing, we impose the constraint that no bases within the seed region are paired, calculate the free energy of the constrained ensemble $F_{\text{constrained}}$ and use the equation

$$p_{\text{unfolded seed}} = \exp\left(-\frac{F_{\text{constrained}} - F_{\text{unconstrained}}}{RT}\right), \tag{S1}$$

where $F_{\text{unconstrained}}$ is the free energy of the ensemble without any imposed constraints.

Having calculated $p_{\text{unfolded seed}}$ for all mutants in the point-mutational neighborhood of the WT except those that have an altered seed region, we then calculate $\Delta p_{\text{unfolded seed}} = p_{\text{unfolded seed mutant}} - p_{\text{unfolded seed WT}}$. We then use this quantity as a criterion for ranking these mutants and build an ROC curve in order to assess its power to predict disease-related mutations (Figure S3).

The change in the probability that the seed region is unfolded, $\Delta p_{\text{unfolded seed}}$, does not perform significantly better than random for any of the datasets that we studied, suggesting that the likelihood that the seed region is unfolded does not play an important role in determining miRNA activity.

2.2 Hamming-distance-based criteria

2.2.1 Normalized Hamming distance from the WT, $\langle d_{\text{Hamming}} \rangle L^{-1}$

We formulate another criterion based on the average Hamming distance $\langle d_{\text{Hamming mutant}} \rangle$ from the WT which registers smaller differences in secondary structure than the percentile-based one discussed in the main text. To do this, we normalize the average Hamming distance by the miRNA length L, $\langle d_{\text{Hamming}} \rangle L^{-1}$. We then test whether disease-related mutants from SomamiR and miRNASNP-v3 tend to change miRNA secondary structure more than other mutants by ranking mutants in *ascending order* of their $\langle d_{\text{Hamming mutant}} \rangle$ and building an ROC curve for this criterion (Figure S4).

The normalized Hamming distance, $\langle d_{\text{Hamming}} \rangle L^{-1}$, has A_{ROC} significantly smaller than 0.5 for the 45 miRNASNP-v3 entries related to diseases other than cancer for which $N_{\text{mut seed}} \geq 1$ and $N_{\text{mut non-seed}} \geq 1$. Since we order mutants in ascending order of $\langle d_{\text{Hamming}} \rangle L^{-1}$, this means that mutants with a larger $\langle d_{\text{Hamming}} \rangle L^{-1}$, which rank lower, tend to be associated with disease.

2.2.2 $\langle d_{\text{Hamming}} \rangle$ percentile

Additional ROC curves for data from miRNASNP-v3 are shown in Figure S6.

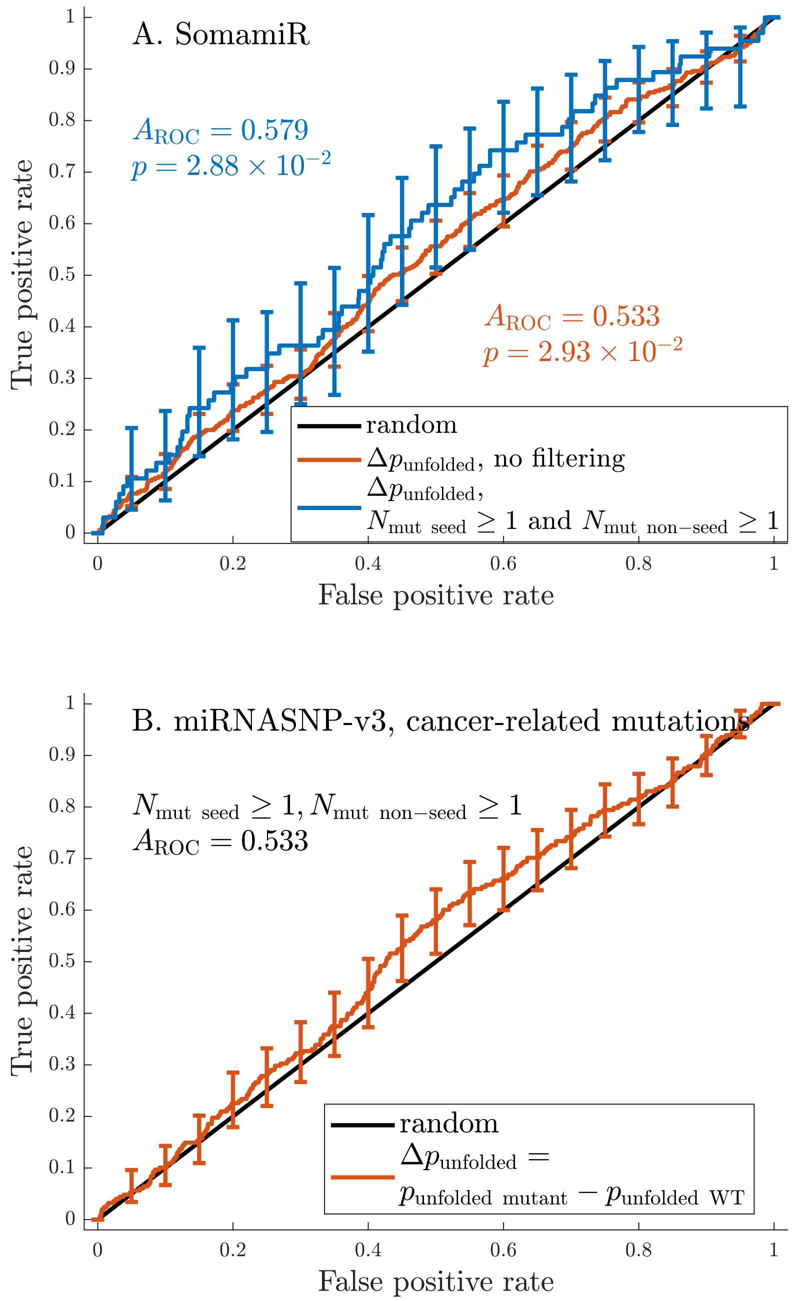
A. SomamiR
$A_{\mathrm{ROC}} = 0.579$
$p = 2.88 \times 10^{-2}$
$A_{\mathrm{ROC}} = 0.533$
$p = 2.93 \times 10^{-2}$
random
$\Delta p_{\mathrm{unfolded}}$, no filtering
$\Delta p_{\mathrm{unfolded}}$,
$N_{\mathrm{mut\ seed}} \geq 1$ and $N_{\mathrm{mut\ non-seed}} \geq 1$
True positive rate
False positive rate
B. miRNASNP-v3, cancer-related mutations
$N_{\mathrm{mut\ seed}} \geq 1, N_{\mathrm{mut\ non-seed}} \geq 1$
$A_{\mathrm{ROC}} = 0.533$
random
$\Delta p_{\mathrm{unfolded}} =$
$p_{\mathrm{unfolded\ mutant}} - p_{\mathrm{unfolded\ WT}}$
True positive rate
False positive rate

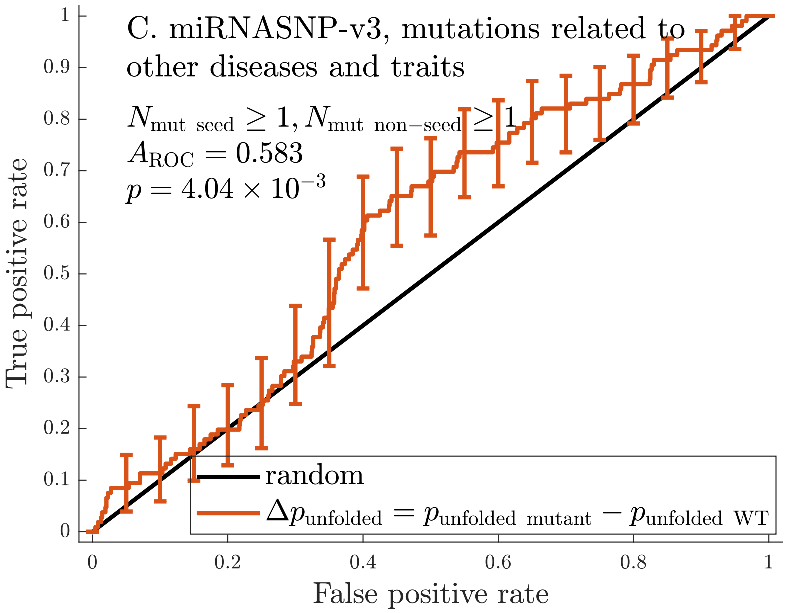

Figure S1: **The change in probability that the miRNA is fully unfolded associated with a mutation is a predictor of the mutation's relationship with disease.** ROC curves built using $\Delta p_{\mathrm{unfolded}} = p_{\mathrm{unfolded\ mutant}} - p_{\mathrm{unfolded\ WT}}$ as the criterion for predicting disease-related mutations. Data from SomamiR (A), information on cancer-related mutations (B) and mutations related to other traits and diseases (C) from miRNASNP-v3, either with no filtering based on $N_{\mathrm{mut\ seed}}$ and $N_{\mathrm{mut\ non-seed}}$ (orange curves) or with $N_{\mathrm{mut\ seed}} \geq 1$ and $N_{\mathrm{mut\ non-seed}} \geq 1$ (blue curves) for all mature miRNAs considered here. Error bars indicate pointwise 95% confidence bounds calculated at 21 equally spaced points with the bootstrapping method [3]. The area under the ROC curve (A_{ROC}) and the Mann-Whitney p-value [4] for the curves are also indicated. The $\Delta p_{\mathrm{unfolded}}$ criterion performs significantly better than the random one for all three datasets ($p < 0.05$) if no filtering is applied, indicating that the probability that disease-related mutants are fully unfolded tends to be higher than that for other mutants. This could be because mutants with a higher $p_{\mathrm{unfolded\ mutant}}$ have a higher activity than their respective WTs, and, in the case of cancer-associated mutations, they may be more effective at downregulating tumour suppressor genes. When the analysis for each dataset is restricted to just the miRNAs for which at least one mutation in the seed region and the rest of the mature miRNA, A_{ROC} increases and so does the certainty that the criterion outperforms the random one, except for the set of cancer-associated mutations from miRNASNP-v3, for which we observe changes in the opposite direction.

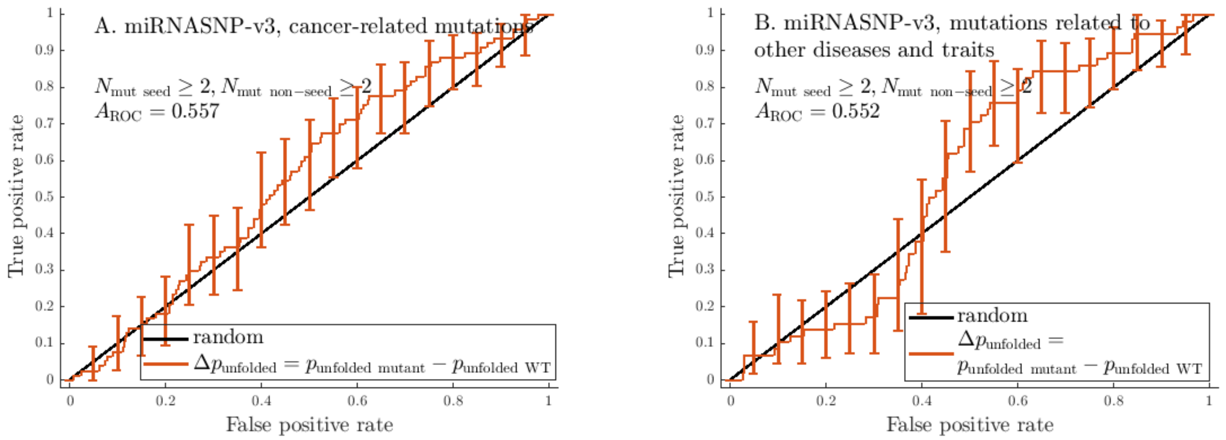

Figure S2: ROC curves built using $\Delta p_{\text{unfolded}} = p_{\text{unfolded mutant}} - p_{\text{unfolded WT}}$ as the criterion for predicting disease-related mutations. Data taken from miRNASNP-v3. Error bars indicate pointwise 95 % confidence bounds calculated at 21 equally spaced points with the bootstrapping method [3]. The area under the ROC curve (A_{ROC}) is also indicated.

2.3 Positional-entropy-based criteria

2.3.1 Change of the positional entropy of the mutated site with respect to the WT

We use the change in positional entropy $S^{(\text{i})}$ at the mutated site as defined in Eq. (3) as a criterion for predicting whether a mutation is associated with disease and present the corresponding ROC curves for various datasets in Figure S8.

2.3.2 Mutant average positional entropy

Calculating the average positional entropy, $\langle \Delta S \rangle$, for an RNA molecule from the positional entropies of the individual sites, $S^{(\text{i})}$, requires straightforward application of the definition in Eq. (**??**)

$$\langle S \rangle = -\frac{1}{L} \sum_{\text{i}=1}^{L} S^{(\text{i})}, \tag{S2}$$

where L is the number of nucleotides in the RNA molecule. The values of $\langle S \rangle$ for the mutant and the WT are comparable only if 1) the minimum-free-energy (MFE) structures both of them have secondary structure or 2) neither MFE structure has secondary structure. Note that this latter case is much less common as base-pairing is energetically favourable and usually possible. We analyze these two cases separately.

The average positional entropy of an RNA, $\langle S \rangle$ as defined above, is a measure of the stability of its fold. We use it as a criterion for predicting whether a mutation is associated with disease and present the corresponding ROC curves for various datasets in Figure S9.

$\langle S_{\text{mutant}} \rangle$, performs significantly better than random for the set of 28 cancer-related miRNAs with $N_{\text{mut seed}} \geq 2$ and $N_{\text{mut non-seed}} \geq 2$, indicating that disease-related mutants in this set tend to have a more stable fold than other mutants.

2.3.3 Change of the average positional entropy with respect to the WT

If the secondary structure of a microRNA affects its interaction with its targets, then one may hypothesize that the mutations which significantly change the stability of that structure would be the ones with the

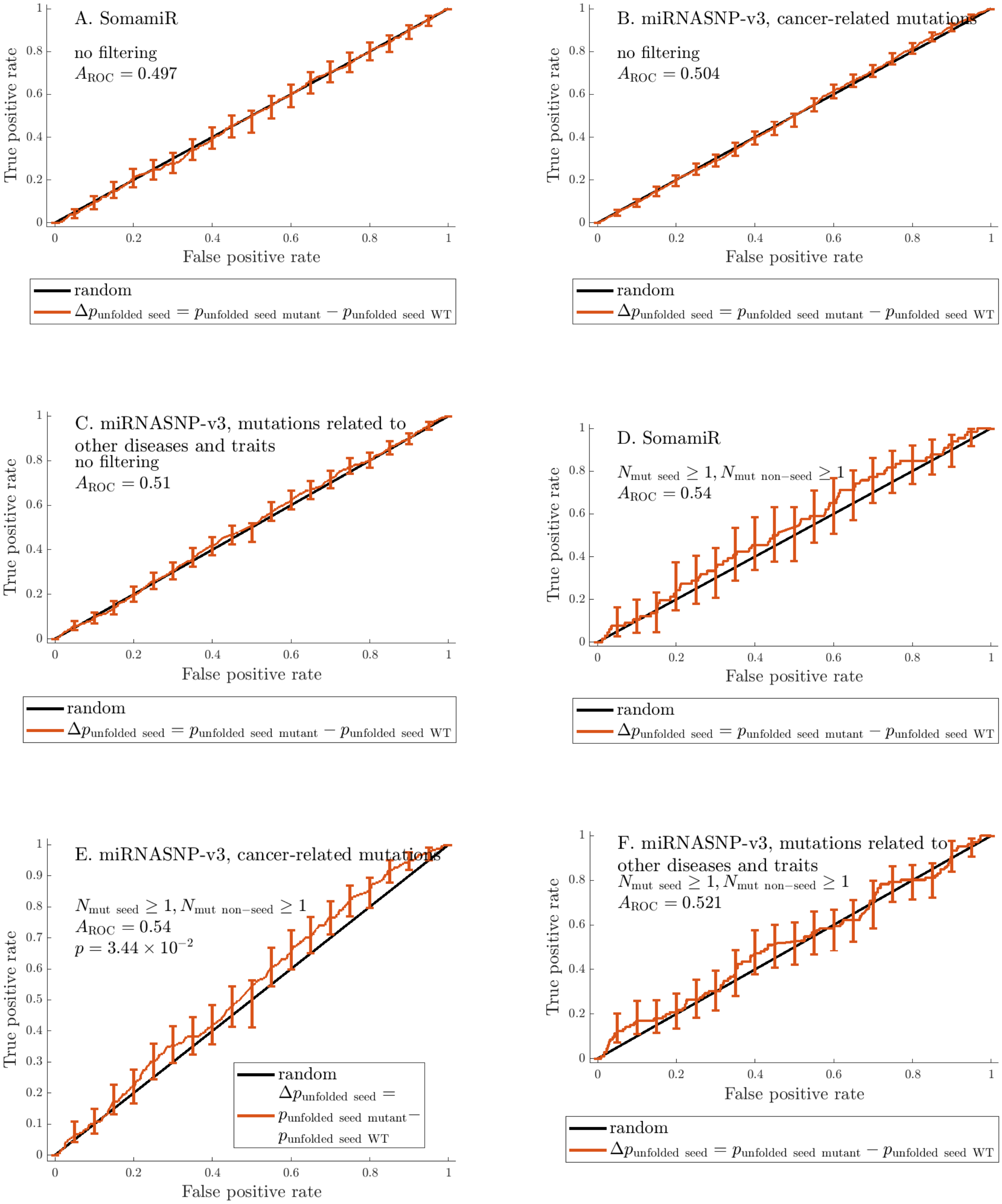
A. SomamiR
no filtering
$A_{\text{ROC}} = 0.497$
B. miRNASNP-v3, cancer-related mutations
no filtering
$A_{\text{ROC}} = 0.504$
C. miRNASNP-v3, mutations related to other diseases and traits
no filtering
$A_{\text{ROC}} = 0.51$
D. SomamiR
$N_{\text{mut seed}} \geq 1, N_{\text{mut non-seed}} \geq 1$
$A_{\text{ROC}} = 0.54$
E. miRNASNP-v3, cancer-related mutations
$N_{\text{mut seed}} \geq 1, N_{\text{mut non-seed}} \geq 1$
$A_{\text{ROC}} = 0.54$
$p = 3.44 \times 10^{-2}$
F. miRNASNP-v3, mutations related to other diseases and traits
$N_{\text{mut seed}} \geq 1, N_{\text{mut non-seed}} \geq 1$
$A_{\text{ROC}} = 0.521$
True positive rate
False positive rate
random
$\Delta p_{\text{unfolded seed}} = p_{\text{unfolded seed mutant}} - p_{\text{unfolded seed WT}}$

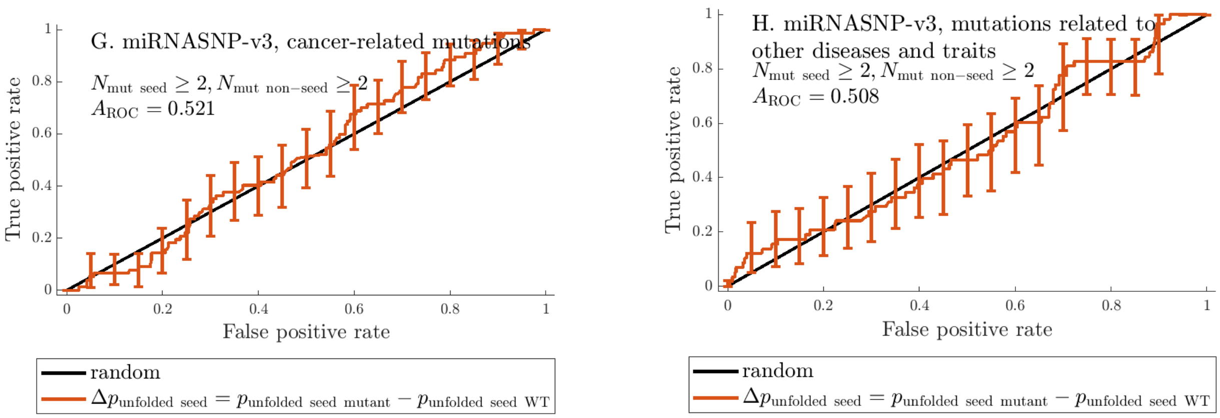

Figure S3: ROC curves built using $\Delta p_{\text{unfolded seed}} = p_{\text{unfolded seed mutant}} - p_{\text{unfolded seed WT}}$ as the criterion for predicting disease-related mutations. Data taken from SomamiR and miRNASNP-v3.

greatest effect on miRNA function. We use the difference between the average positional entropy for the mutant and the WT, $\langle \Delta S \rangle = \langle S_{\text{mut}} \rangle - \langle S_{\text{WT}} \rangle$ as a measure of this change in stability, with low $\langle S_{\text{mut}} \rangle$ indicating a stable secondary structure [5]. As a miRNA needs to bind to an mRNA in order to regulate gene expression, one may expect that the stability of its fold is relevant to its function. In particular, one may expect that mutants that are more stably folded (i.e., have lower $\langle S_{\text{mut}} \rangle$) than the respective WT may be less effective at binding to mRNA and thus gene regulation, potentially leading to disease. We rank mutants according to their value of this quantity and check whether it correlates with disease association; we plot ROC curves for this criterion in Figure S10.

Strictly, the values of $\langle S \rangle$ for the mutant and the WT are comparable only if 1) the minimum-free-energy (MFE) structures both of them have secondary structure or 2) neither MFE structure has secondary structure. We analyze these two cases separately below; for comparison, we also measure the performance of the $\langle S_{\text{mut}} \rangle$ without regard for whether the WT and mutants are folded.

We calculate $\langle S_{\text{mut}} \rangle$ for the point mutational neighborhoods of the WT miRNAs represented in miRNASNP-v3 and SomamiR, while keeping the sequences of their seed regions (sequence positions 2-7) fixed. The change in the average positional entropy with respect to the wild type, $\langle \Delta S \rangle$, performs significantly better than random for the set of 9 miRNAs associated with diseases other than cancer for which $N_{\text{mut seed}} \geq 2$ and $N_{\text{mut non-seed}} \geq 2$. The p-value in this case is almost an order of magnitude higher than that for the subset of 6 miRNAs which have folded mutants and WTs, and the criterion performs no better than random for the other 3 miRNAs. This indicates that the significant effect is in the change of stability of non-trivial folds, and that, as expected, it is only appropriate to compare $\langle \Delta S \rangle$ if 1) both the mutant and the WT are folded or 2) neither is folded.

2.3.3.1 Folded mutant and WT When only miRNAs with $N_{\text{mut seed}} \geq 2$ and $N_{\text{mut non-seed}} \geq 2$ from miRNASNP-v3 data are considered, the change in the average positional entropy $\langle \Delta S \rangle$ becomes a useful predictor of disease association for mutants and WT that are both folded, see Figure S11. The values of A_{ROC} of 0.580 and 0.615 that we calculated for mutations related to cancer and other diseases respectively, indicate that decreasing stability is associated with disease for the clusters of 18 and 9 miRNAs in these subsets of data.

A miRNA's folding may have an effect on its function in two ways - it could either have its own functional purpose, or it could affect the interaction with the target site as it would make the binding sites within the miRNA less accessible. In the first case, a mutant may disrupt function by causing a change to the MFE

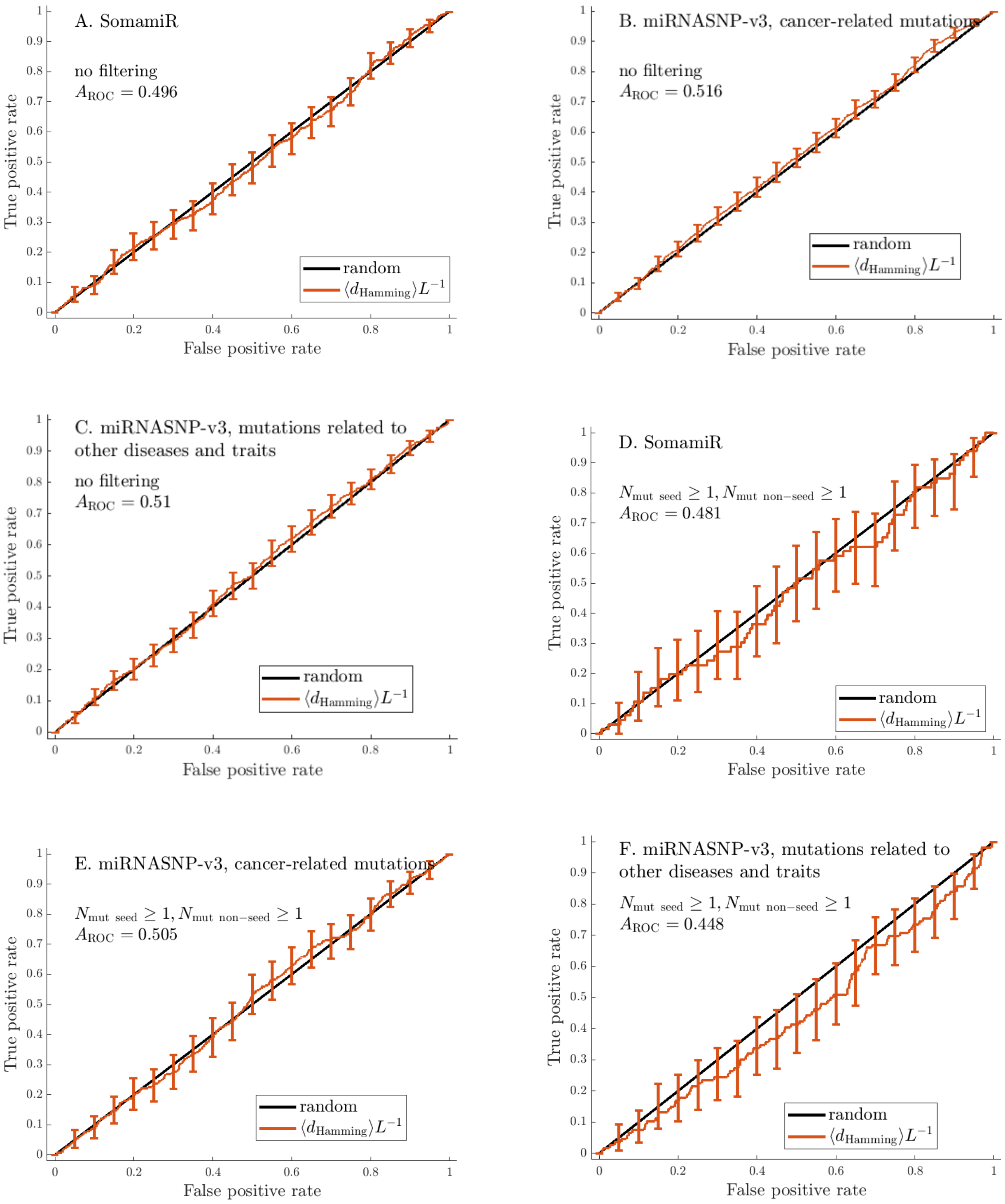
A. SomamiR
no filtering
$A_{\mathrm{ROC}} = 0.496$
B. miRNASNP-v3, cancer-related mutations
no filtering
$A_{\mathrm{ROC}} = 0.516$
C. miRNASNP-v3, mutations related to other diseases and traits
no filtering
$A_{\mathrm{ROC}} = 0.51$
D. SomamiR
$N_{\mathrm{mut\ seed}} \geq 1, N_{\mathrm{mut\ non-seed}} \geq 1$
$A_{\mathrm{ROC}} = 0.481$
E. miRNASNP-v3, cancer-related mutations
$N_{\mathrm{mut\ seed}} \geq 1, N_{\mathrm{mut\ non-seed}} \geq 1$
$A_{\mathrm{ROC}} = 0.505$
F. miRNASNP-v3, mutations related to other diseases and traits
$N_{\mathrm{mut\ seed}} \geq 1, N_{\mathrm{mut\ non-seed}} \geq 1$
$A_{\mathrm{ROC}} = 0.448$
random
$\langle d_{\mathrm{Hamming}} \rangle L^{-1}$
True positive rate
False positive rate

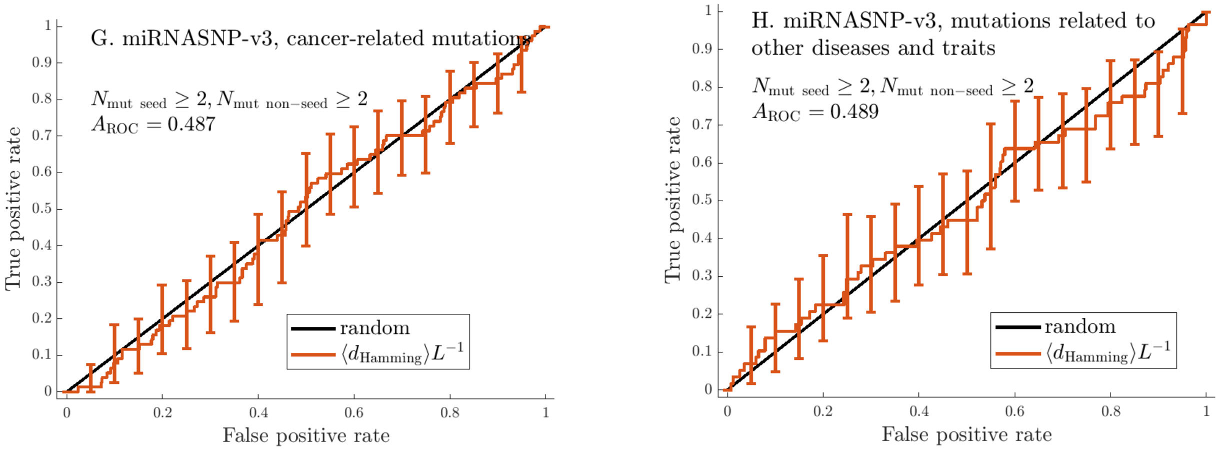

Figure S4: ROC curves built using $\langle d_{\text{Hamming}} \rangle L^{-1}$ as the criterion for predicting disease-related mutations. Data taken from SomamiR and miRNASNP-v3. The labels indicate the areas under the curves and the p-values for the criteria that perform significantly better than the random predictor (based on the two-sided Mann-Whitney test).

secondary structure or making it less stable; in the second one, it a mutation may interfere with miRNA function by causing sites essential to target binding to enter base pairs. Our metrics aim to quantify different types of secondary structure changes in order to detect any kind of association between modified miRNA secondary structure and disease.

2.3.3.2 Unfolded mutant and WT As miRNAs need to bind to mRNAs to perform their function, one may expect that the fully unfolded state with no base pairs is ideal for performing their functions. Under this hypothesis, mutations that make the unfolded state less stable would disrupt miRNA function. The ROC curves for this subset of the data are illustrated in Figure S13. As one can see in Tables S3-S10, it is much rarer for both the mutant and the WT to be unfolded and therefore, the sample size is much smaller than for the case with folded mutant and WT. This, combined with the many effects unrelated to miRNA SS, is probably the reason that the change in entropy does not perform significantly better than random for unfolded mutant and miRNA WTs.

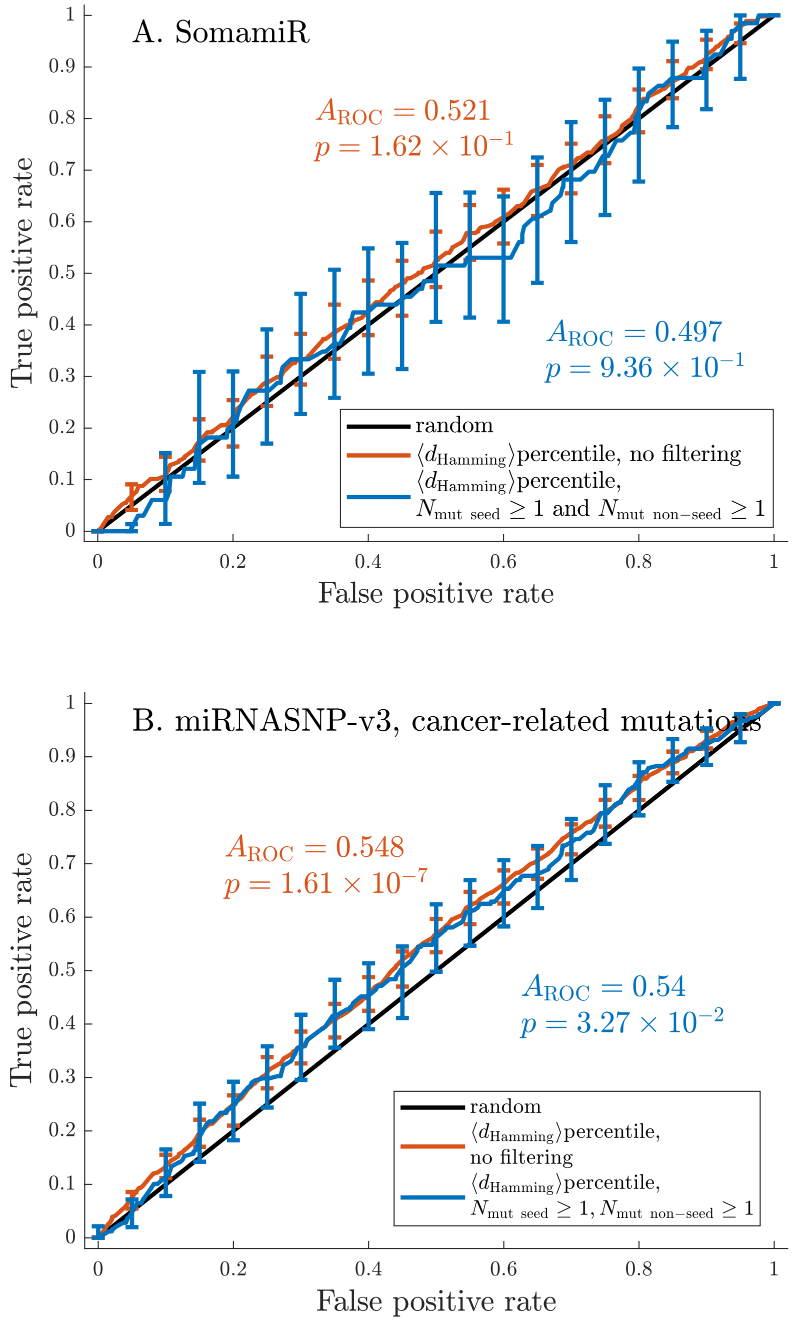

A. SomamiR
$A_{ROC} = 0.521$
$p = 1.62 \times 10^{-1}$
$A_{ROC} = 0.497$
$p = 9.36 \times 10^{-1}$
random
$\langle d_{\text{Hamming}} \rangle$percentile, no filtering
$\langle d_{\text{Hamming}} \rangle$percentile,
$N_{\text{mut seed}} \geq 1$ and $N_{\text{mut non-seed}} \geq 1$
True positive rate
False positive rate
B. miRNASNP-v3, cancer-related mutations
$A_{ROC} = 0.548$
$p = 1.61 \times 10^{-7}$
$A_{ROC} = 0.54$
$p = 3.27 \times 10^{-2}$
random
$\langle d_{\text{Hamming}} \rangle$percentile,
no filtering
$\langle d_{\text{Hamming}} \rangle$percentile,
$N_{\text{mut seed}} \geq 1, N_{\text{mut non-seed}} \geq 1$
True positive rate
False positive rate

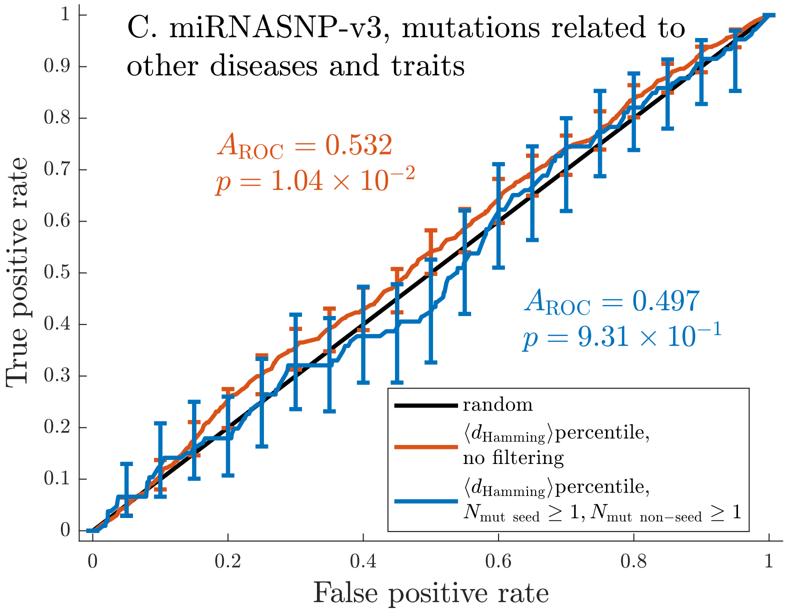

Figure S5: **The change in SS associated with a mutation as measured by the percentile of the normalized Hamming distance in SS between the WT and mutated ensembles is a predictor of the mutation's relationship with disease.** ROC curves built using $\langle d_{\mathrm{Hamming}} \rangle$%ile as the criterion for predicting disease-related mutations. Data from SomamiR (A), cancer-related mutations (B) and mutations related to other traits and diseases (C) from miRNASNP-v3, with either no filtering based on $N_{\mathrm{mut\ seed}}$ and $N_{\mathrm{mut\ non-seed}}$ (red curves) or with $N_{\mathrm{mut\ seed}} \geq 1$ and $N_{\mathrm{mut\ non-seed}} \geq 1$ (blue curves) for all mature miRNAs considered here. Error bars indicate pointwise 95% confidence bounds calculated at 21 equally spaced points with the bootstrapping method [3]. The area under the ROC curve (A_{ROC}) and the Mann-Whitney p-value [4] for the curves are also indicated. The $\Delta p_{\mathrm{unfolded}}$ criterion performs significantly better than the random one for both datasets, indicating that the probability that disease-related mutants are fully unfolded tends to be higher than that for other mutants. This could be because mutants with a higher $p_{\mathrm{unfolded\ mutant}}$ have a higher activity than their respective WTs, and, in the case of cancer-associated mutations, they may be more effective at downregulating tumour suppressor genes.

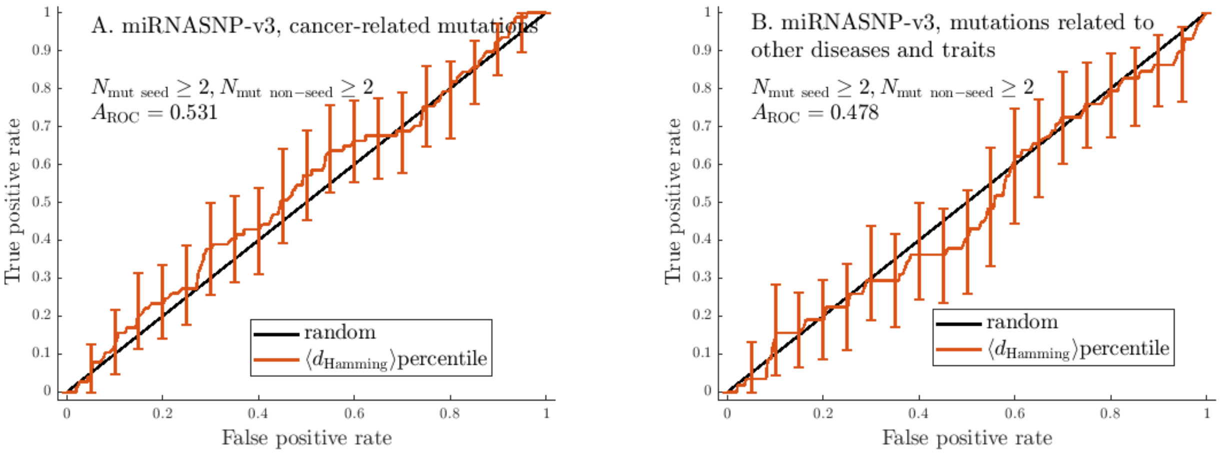

Figure S6: ROC curves built using $\langle d_{\text{Hamming}} \rangle$%ile as the criterion for predicting disease-related mutations. Data taken from miRNASNP-v3.

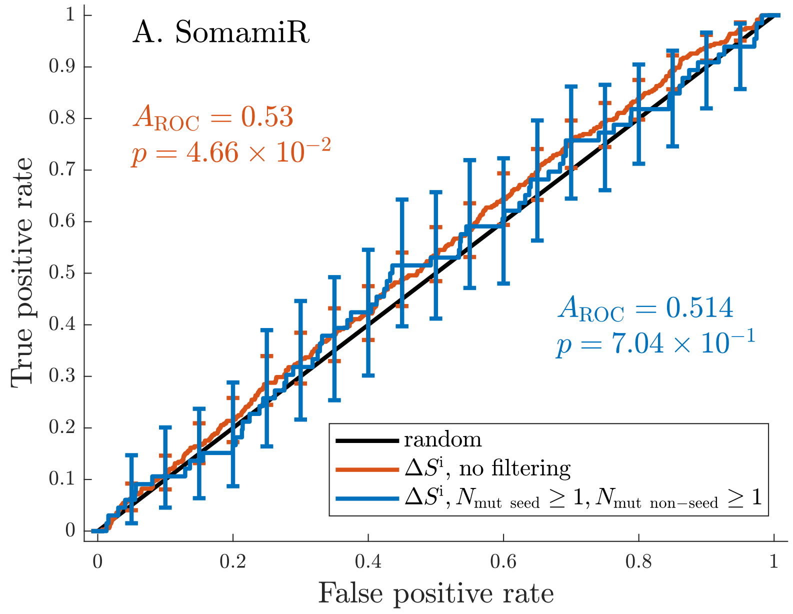
A. SomamiR
$A_{\mathrm{ROC}} = 0.53$
$p = 4.66 \times 10^{-2}$
$A_{\mathrm{ROC}} = 0.514$
$p = 7.04 \times 10^{-1}$
random
ΔS^{i}, no filtering
$\Delta S^{\mathrm{i}}, N_{\mathrm{mut\ seed}} \geq 1, N_{\mathrm{mut\ non-seed}} \geq 1$
True positive rate
False positive rate
0
0.1
0.2
0.3
0.4
0.5
0.6
0.7
0.8
0.9
1
0
0.2
0.4
0.6
0.8
1

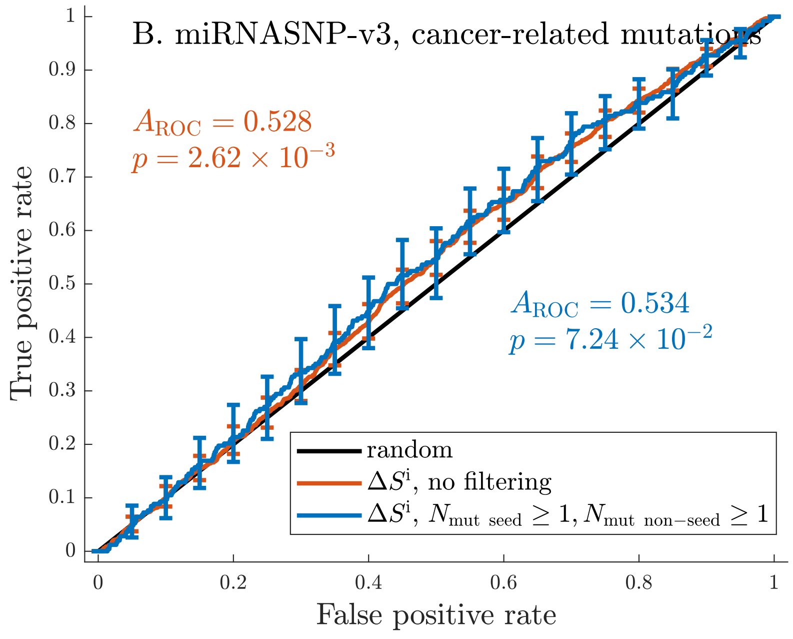
B. miRNASNP-v3, cancer-related mutations
$A_{\mathrm{ROC}} = 0.528$
$p = 2.62 \times 10^{-3}$
$A_{\mathrm{ROC}} = 0.534$
$p = 7.24 \times 10^{-2}$
random
ΔS^{i}, no filtering
$\Delta S^{\mathrm{i}}, N_{\mathrm{mut\ seed}} \geq 1, N_{\mathrm{mut\ non-seed}} \geq 1$
True positive rate
False positive rate
0
0.1
0.2
0.3
0.4
0.5
0.6
0.7
0.8
0.9
1
0
0.2
0.4
0.6
0.8
1

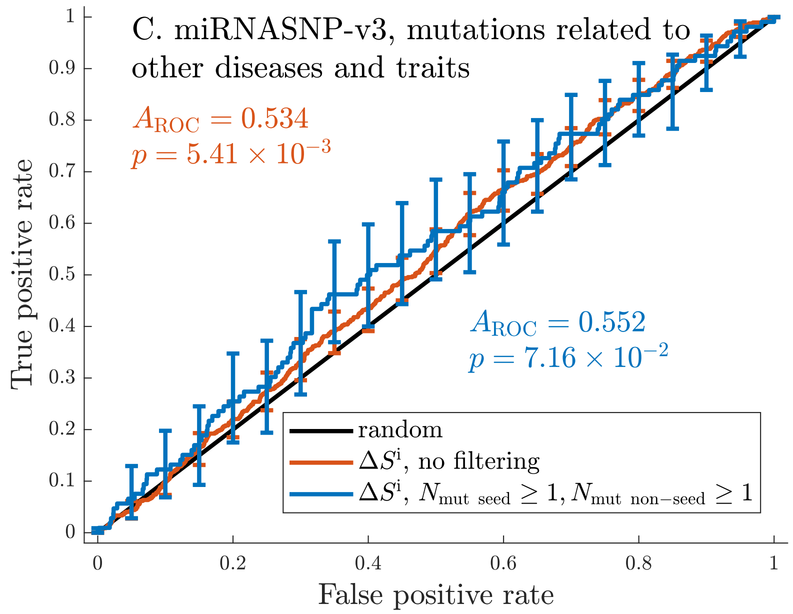

Figure S7: **The change in the positional entropy at the mutated site in a point mutant is a predictor of the mutation's relationship with disease.** ROC curves built using $\Delta S^{\text{i}} = S^{\text{i}}_{\text{mut}} - S^{\text{i}}_{\text{WT}}$ as the criterion for predicting disease-related mutations. Data from SomamiR (A), cancer-related mutations (B) and mutations related to other traits and diseases (C) from miRNASNP-v3, with either no filtering based on $N_{\text{mut seed}}$ and $N_{\text{mut non-seed}}$ (red curves) or with $N_{\text{mut seed}} \geq 1$ and $N_{\text{mut non-seed}} \geq 1$ (blue curves) for all mature miRNAs considered here. Error bars indicate pointwise 95% confidence bounds calculated at 21 equally spaced points with the bootstrapping method [3]. The area under the ROC curve (A_{ROC}) and the Mann-Whitney p-value [4] for the curves are also indicated. The ΔS^{i} criterion performs significantly better than the random one for both datasets, when no filtering by $N_{\text{mut seed}}$ and $N_{\text{mut non-seed}}$ is applied. This indicates that the change in positional entropy at the mutated site tends to be lower for disease-related mutants.

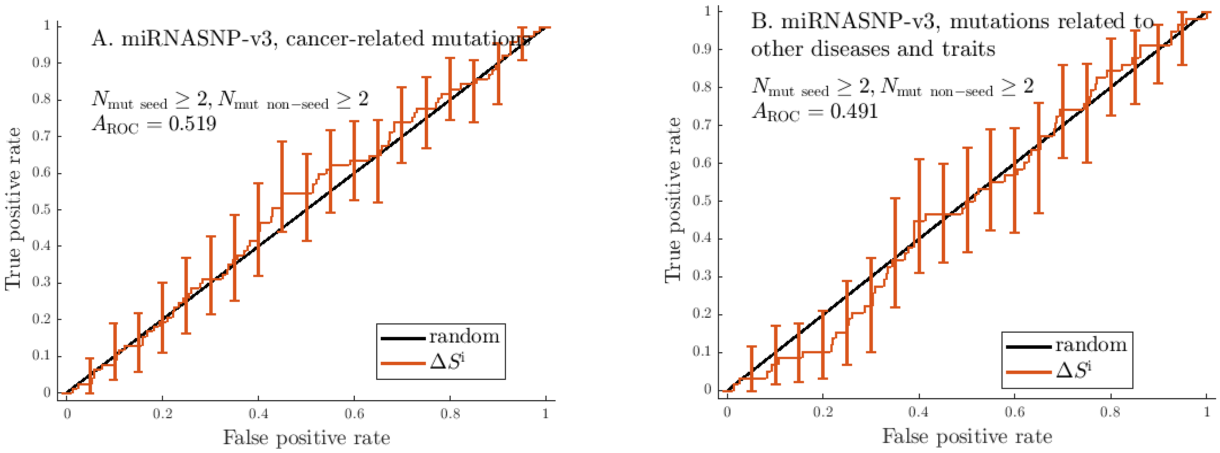

Figure S8: ROC curves built using ΔS^{i} as the criterion for predicting disease-related mutations for data from SomamiR and miRNASNP-v3. The labels indicate the areas under the curves and the p-values for the criteria that perform significantly better than the random predictor (based on the two-sided Mann-Whitney test).

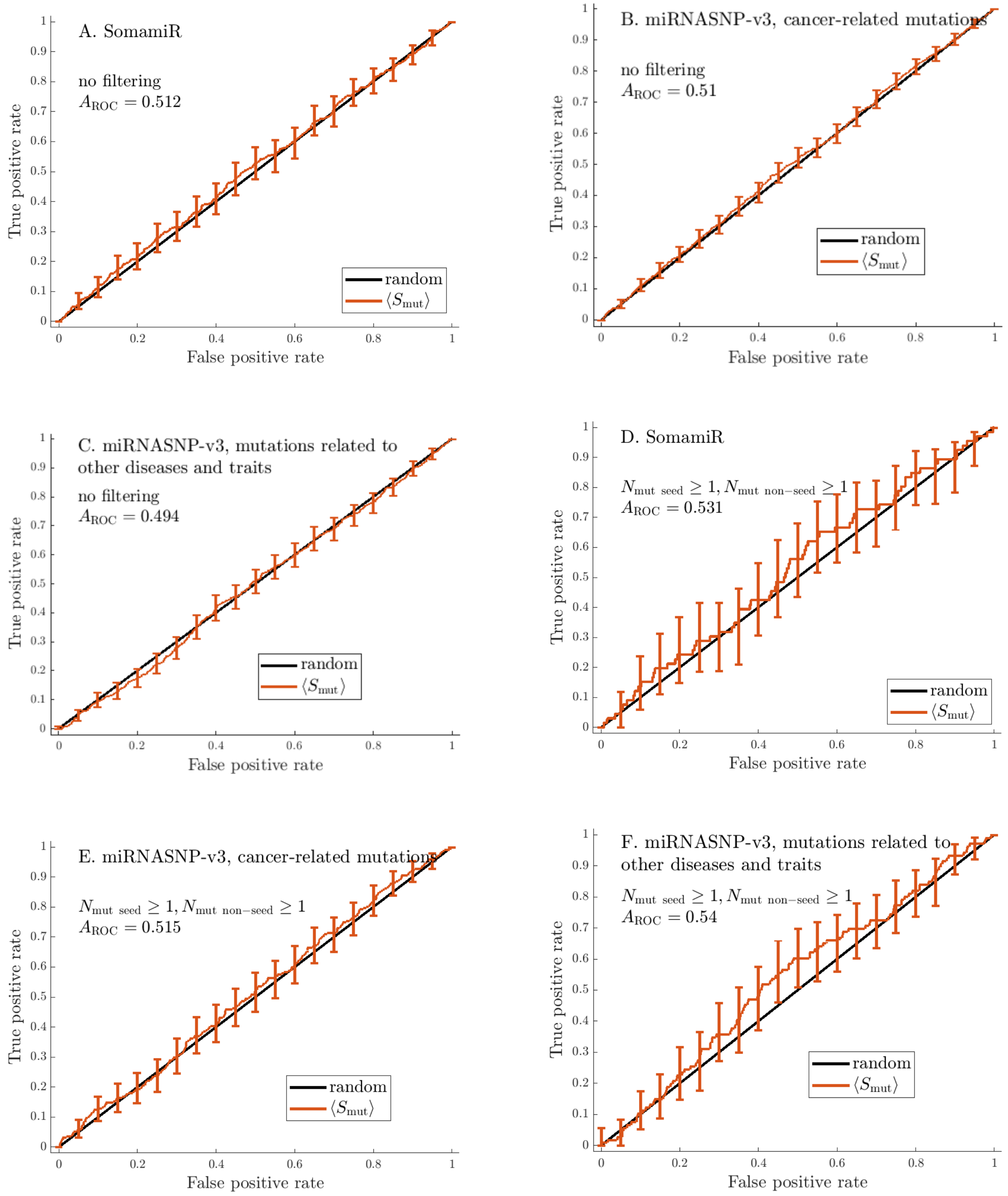
A. SomamiR
no filtering
$A_{\mathrm{ROC}} = 0.512$
B. miRNASNP-v3, cancer-related mutations
no filtering
$A_{\mathrm{ROC}} = 0.51$
C. miRNASNP-v3, mutations related to other diseases and traits
no filtering
$A_{\mathrm{ROC}} = 0.494$
D. SomamiR
$N_{\mathrm{mut\ seed}} \geq 1, N_{\mathrm{mut\ non-seed}} \geq 1$
$A_{\mathrm{ROC}} = 0.531$
E. miRNASNP-v3, cancer-related mutations
$N_{\mathrm{mut\ seed}} \geq 1, N_{\mathrm{mut\ non-seed}} \geq 1$
$A_{\mathrm{ROC}} = 0.515$
F. miRNASNP-v3, mutations related to other diseases and traits
$N_{\mathrm{mut\ seed}} \geq 1, N_{\mathrm{mut\ non-seed}} \geq 1$
$A_{\mathrm{ROC}} = 0.54$
True positive rate
False positive rate
random
$\langle S_{\mathrm{mut}} \rangle$

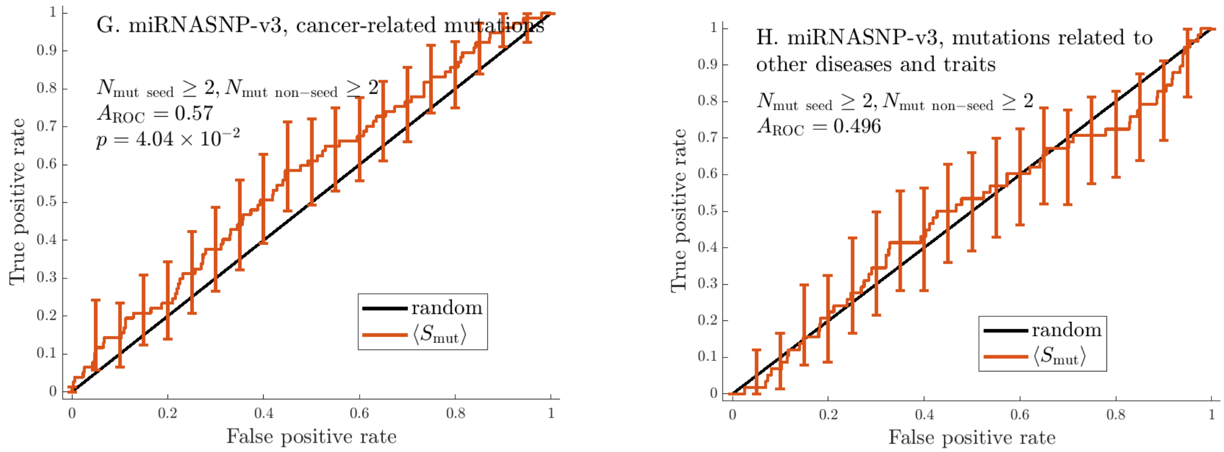

Figure S9: ROC curves built using $\langle S_{\text{mut}} \rangle$ as the criterion for predicting disease-related mutations for data from SomamiR and miRNASNP-v3. The labels indicate the areas under the curves and the p-values for the criteria that perform significantly better than the random predictor (based on the two-sided Mann-Whitney test).

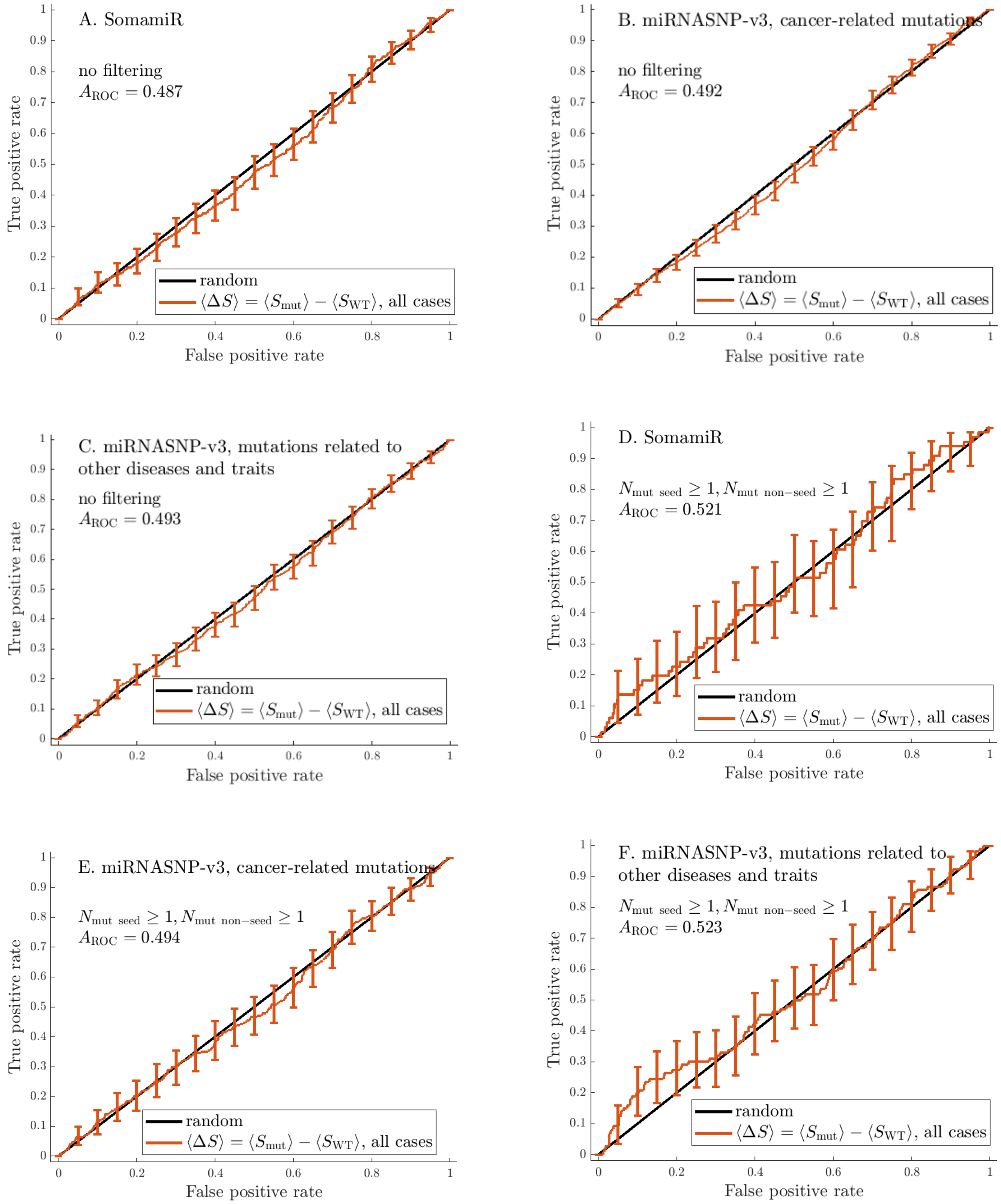
A. SomamiR
no filtering
$A_{\mathrm{ROC}} = 0.487$
B. miRNASNP-v3, cancer-related mutations
no filtering
$A_{\mathrm{ROC}} = 0.492$
C. miRNASNP-v3, mutations related to other diseases and traits
no filtering
$A_{\mathrm{ROC}} = 0.493$
D. SomamiR
$N_{\mathrm{mut\ seed}} \geq 1, N_{\mathrm{mut\ non-seed}} \geq 1$
$A_{\mathrm{ROC}} = 0.521$
E. miRNASNP-v3, cancer-related mutations
$N_{\mathrm{mut\ seed}} \geq 1, N_{\mathrm{mut\ non-seed}} \geq 1$
$A_{\mathrm{ROC}} = 0.494$
F. miRNASNP-v3, mutations related to other diseases and traits
$N_{\mathrm{mut\ seed}} \geq 1, N_{\mathrm{mut\ non-seed}} \geq 1$
$A_{\mathrm{ROC}} = 0.523$
random
$\langle \Delta S \rangle = \langle S_{\mathrm{mut}} \rangle - \langle S_{\mathrm{WT}} \rangle$, all cases
True positive rate
False positive rate

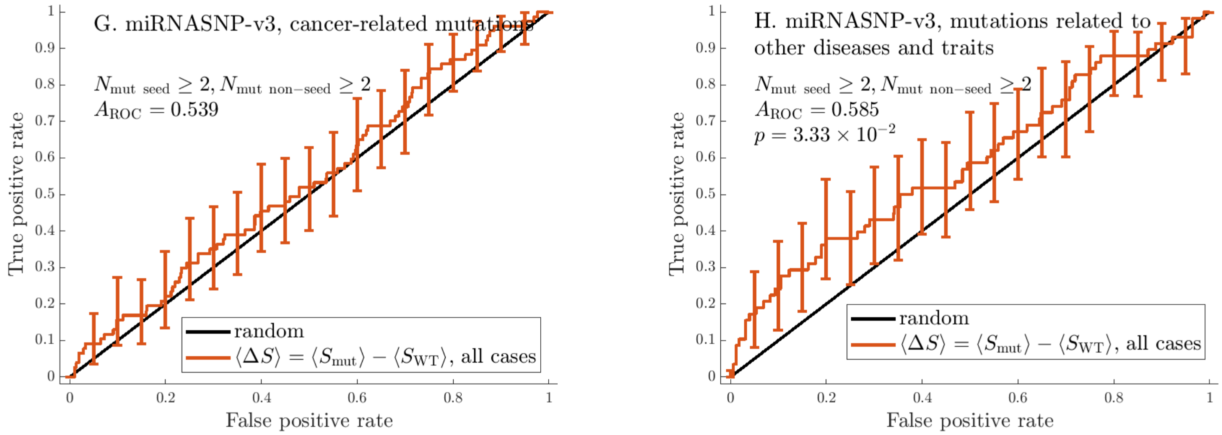

Figure S10: ROC curves built using $\langle \Delta S \rangle = \langle S_{\text{mut}} \rangle - \langle S_{\text{WT}} \rangle$ as the criterion for predicting disease-related mutations applied to data from SomamiR and miRNASNP-v3. The labels indicate the areas under the curves and the p-values for the criteria that perform significantly better than the random predictor (based on the two-sided Mann-Whitney test).

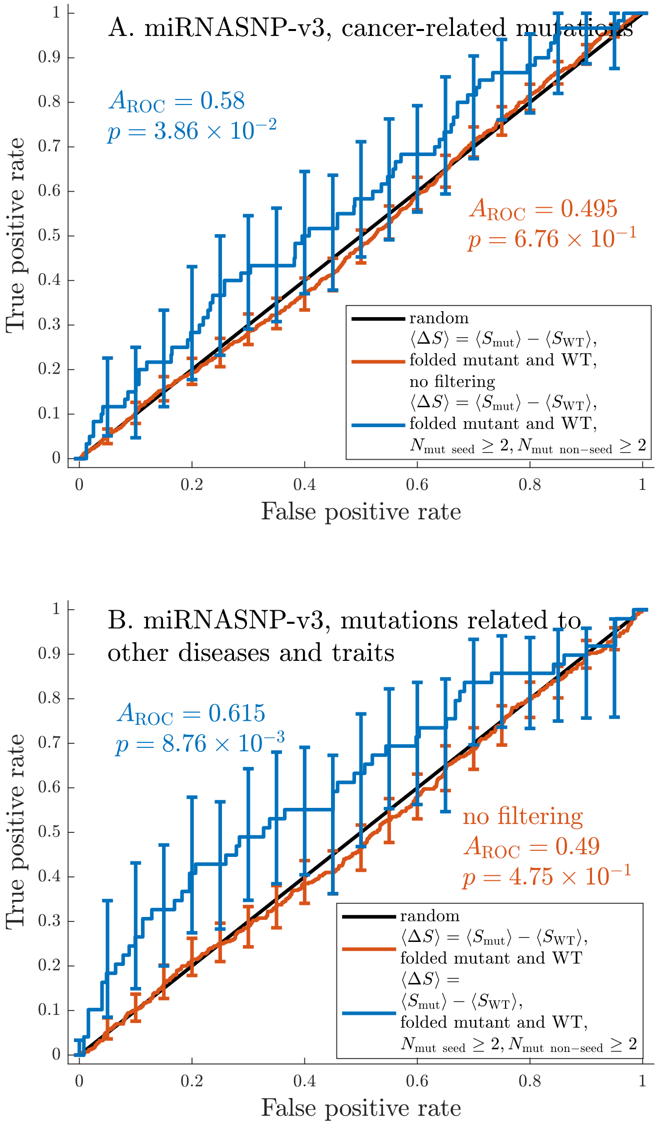

Figure S11: **The change in the average positional entropy, which measures the stability of a fold, of a miRNA associated with a mutation is a predictor of the mutation's relationship with disease for a subset of miRNAs with a comparatively large number of recorded mutations.** ROC curves built characterizing the difference in average positional entropy between mutant and WT that are both folded, $\langle \Delta S \rangle = \langle S_{\rm mut} \rangle - \langle S_{\rm WT} \rangle$. When applied to the unfiltered datasets on mutations related to cancer and other diseases from miRNASNP-v3, this criterion does not perform significantly better than the random one. However, when looking only at miRNAs for which the database records at least two mutations in both the seed and the rest of the mature region, the criterion performs significantly better than random, particularly for mutations unrelated to cancer. The results for cancer-related mutations highlight a cluster of 23 miRNAs for which the change in the stability of the fold plays a significant role, and the mutations related to other traits and diseases highlight another cluster of 9 miRNAs (see Tables S9-S10 in the SI for more information on the samples). The top-ranked mutations are those with the highest $\langle \Delta S \rangle$, which suggests that for miRNAs within these clusters, less stable folds tend to be associated with disease. We explore the data for one of the miRNAs from the non-cancer cluster, hsa-miR-4537, in Figure S17, and find strong association of additional criteria with disease.

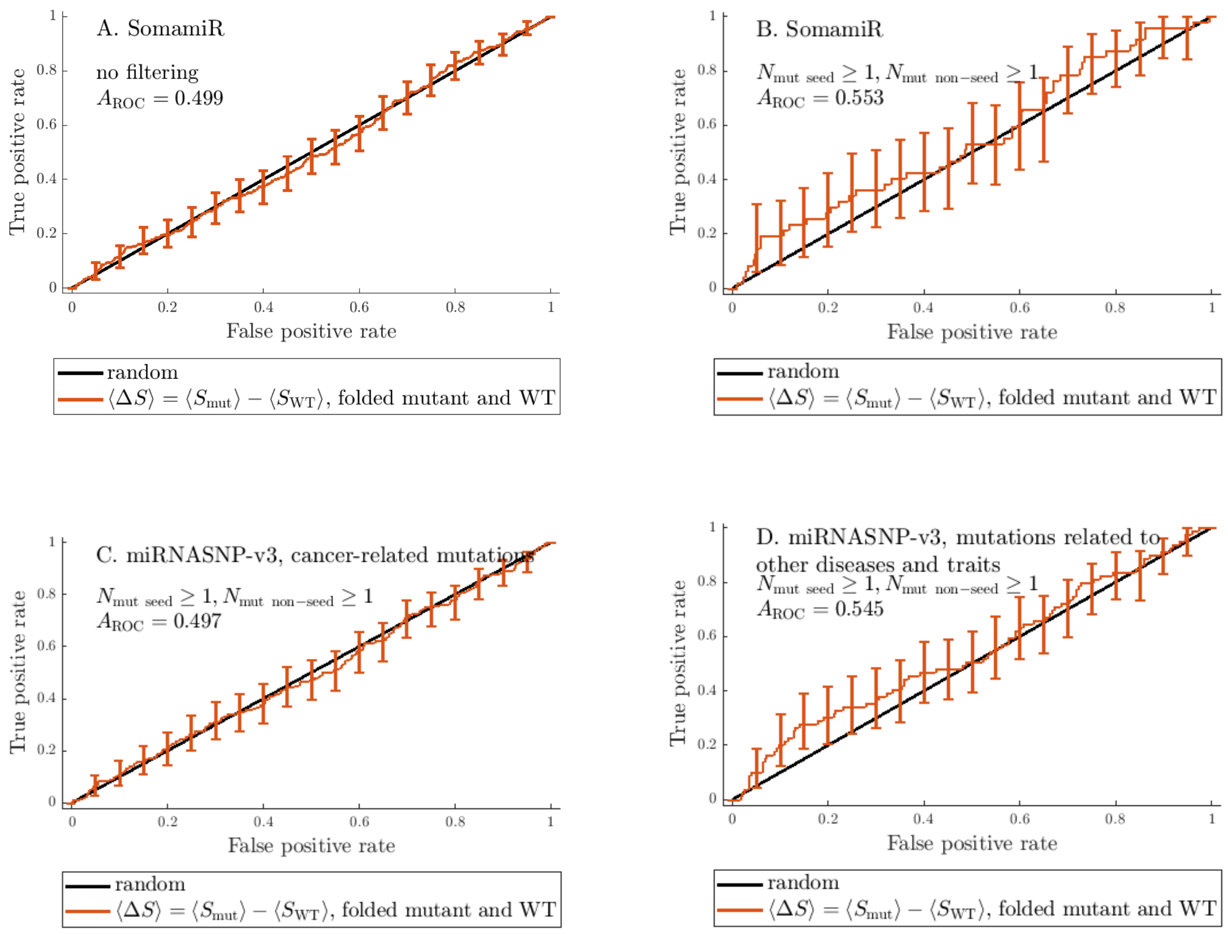

Figure S12: ROC curves built using $\langle \Delta S \rangle = \langle S_{\rm mut} \rangle - \langle S_{\rm WT} \rangle$ as the criterion for predicting disease-related mutations; the plot is based on the cases for which both the mutant and the WT MFE structures are folded. Data taken from SomamiR and miRNASNP-v3.

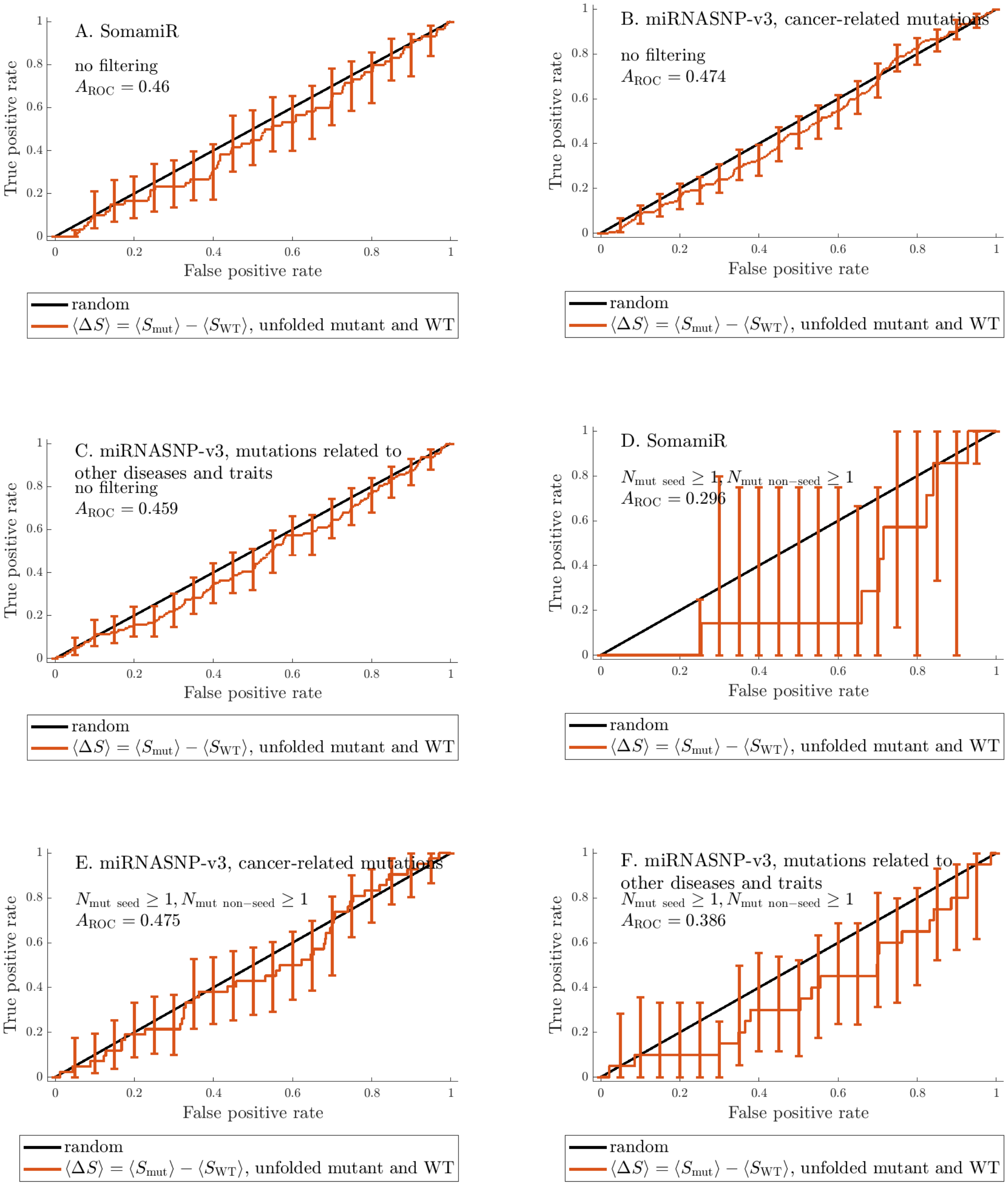
A. SomamiR
no filtering
$A_{\mathrm{ROC}} = 0.46$
B. miRNASNP-v3, cancer-related mutations
no filtering
$A_{\mathrm{ROC}} = 0.474$
C. miRNASNP-v3, mutations related to other diseases and traits
no filtering
$A_{\mathrm{ROC}} = 0.459$
D. SomamiR
$N_{\mathrm{mut\ seed}} \geq 1, N_{\mathrm{mut\ non-seed}} \geq 1$
$A_{\mathrm{ROC}} = 0.296$
E. miRNASNP-v3, cancer-related mutations
$N_{\mathrm{mut\ seed}} \geq 1, N_{\mathrm{mut\ non-seed}} \geq 1$
$A_{\mathrm{ROC}} = 0.475$
F. miRNASNP-v3, mutations related to other diseases and traits
$N_{\mathrm{mut\ seed}} \geq 1, N_{\mathrm{mut\ non-seed}} \geq 1$
$A_{\mathrm{ROC}} = 0.386$
True positive rate
False positive rate
random
$\langle \Delta S \rangle = \langle S_{\mathrm{mut}} \rangle - \langle S_{\mathrm{WT}} \rangle$, unfolded mutant and WT

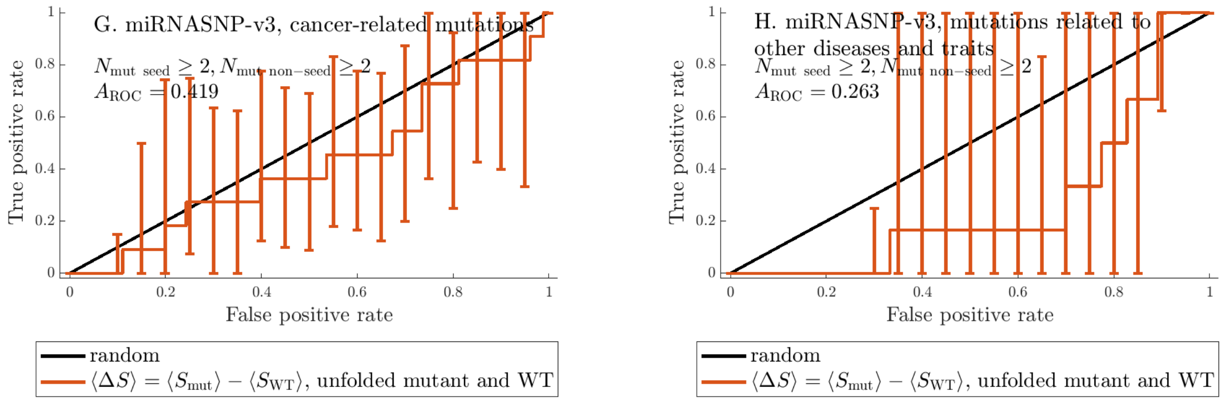

Figure S13: ROC curves built using $\langle \Delta S \rangle = \langle S_{\mathrm{mut}} \rangle - \langle S_{\mathrm{WT}} \rangle$ as the criterion for predicting disease-related mutations; the plot is based on the cases for which both the mutant and the WT MFE structures are fully unfolded. Data taken from SomamiR and miRNASNP-v3.

	all		miRNAs with genes from MiRGeneDB	
criterion	A_{ROC}	p-value	A_{ROC}	p-value
$\Delta p_{\mathrm{unfolded}}$	0.544	4.92×10^{-9}	0.561	1.10×10^{-6}
$\langle d_{\mathrm{Hamming}} \rangle$%ile	0.543	9.15×10^{-9}	0.548	1.24×10^{-4}
$\Delta S^{(\mathrm{i})}$	0.532	2.50×10^{-5}	0.531	1.15×10^{-2}
$d_{\mathrm{Boltzmann}}$	0.529	8.80×10^{-5}	0.534	6.10×10^{-3}

Table S1: The $\Delta p_{\mathrm{unfolded}}$, $\langle d_{\mathrm{Hamming}} \rangle$%ile, $\Delta S^{(\mathrm{i})}$ and $d_{\mathrm{Boltzmann}}$ criteria indicate statistically significant association of secondary structure changes with disease when applied to data from miRNASNP without filtering by disease type, regardless of whether the genes encoding the miRNAs in question are represented in MiRGeneDB 2.1. As elsewhere, mutations are ranked by these criteria, and the receiver-operator characteristic A_{ROC} is used to establish whether they predict disease-associated mutations in the miRNASNP-v3 dataset. The A_{ROC} values for $\Delta p_{\mathrm{unfolded}}$ exceed 0.5 significantly for all three metrics in both subsets of the data, suggesting that at least for some miRNAs secondary structure changes can be indicative of disease association. When checking for significance, we calculate the Mann-Whitney p-value and set the threshold to 0.05.

	miRNAs with genes from MiRGeneDB	
criterion	A_{ROC}	p-value
$\Delta p_{\mathrm{unfolded}}$	0.548	3.64×10^{-2}
$\langle d_{\mathrm{Hamming}} \rangle$%ile	*0.533*	*1.53×10^{-1}*
$\Delta S^{(\mathrm{i})}$	*0.544*	*5.46×10^{-2}*
$d_{\mathrm{Boltzmann}}$	*0.513*	*5.77×10^{-1}*

Table S2: The $\Delta p_{\mathrm{unfolded}}$ criterion is the only one that indicates statistically significant association of secondary structure changes with disease when applied to data from SomamiR pertaining to miRNAs encoded by genes represented in MiRGeneDB 2.1. As elsewhere, mutations are ranked by these criteria, and the receiver-operator characteristic A_{ROC} is used to establish whether they predict disease-associated mutations. When checking for significance, we calculate the Mann-Whitney p-value and set the threshold to 0.05; results with p-values above this threshold in *italics*.

3 Tables and graphical comparisons of measures of criteria performance

A. Performance of various criteria when applied to SomamiR data

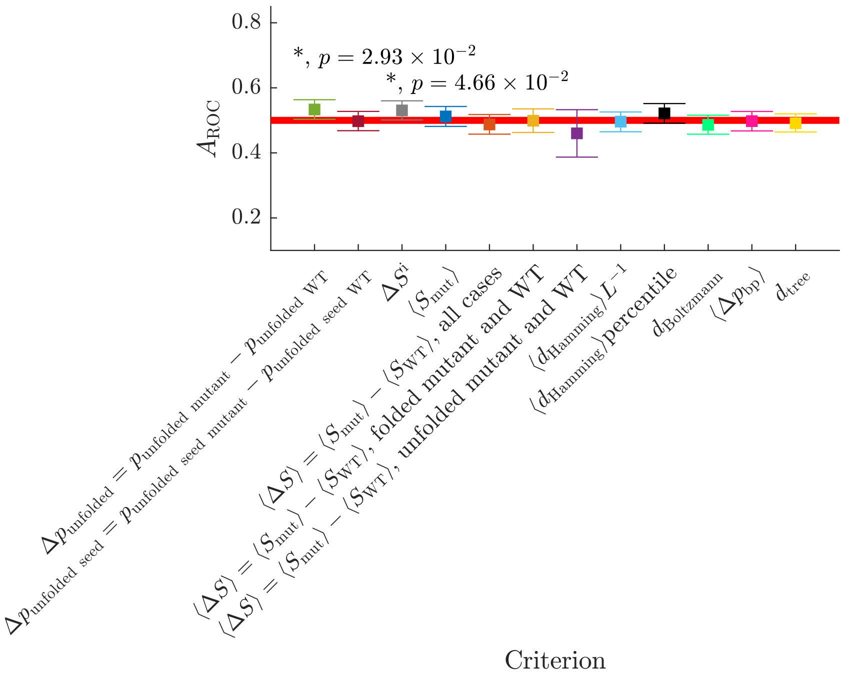

B. Performance of various criteria when applied to miRNASNP-v3 data, cancer-related mutations

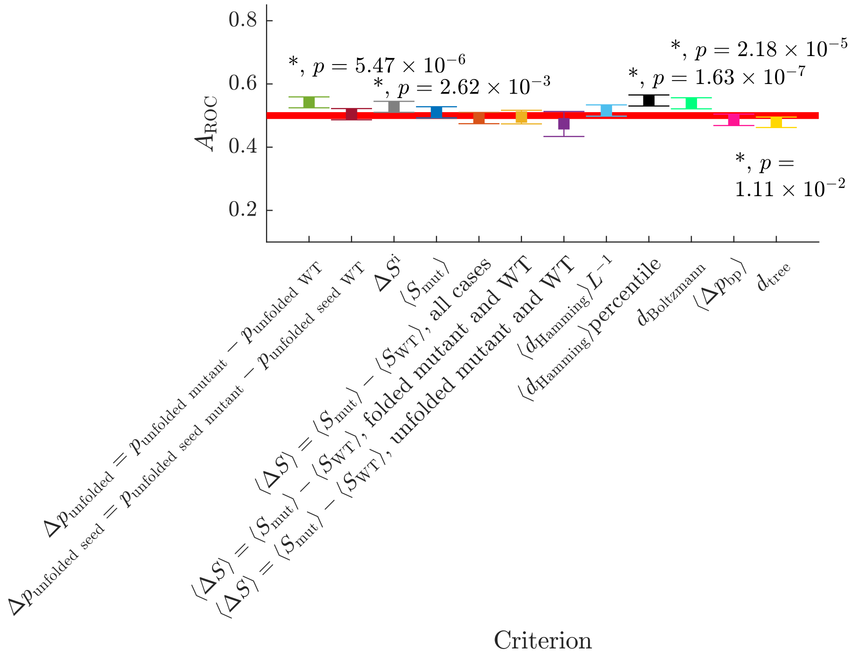

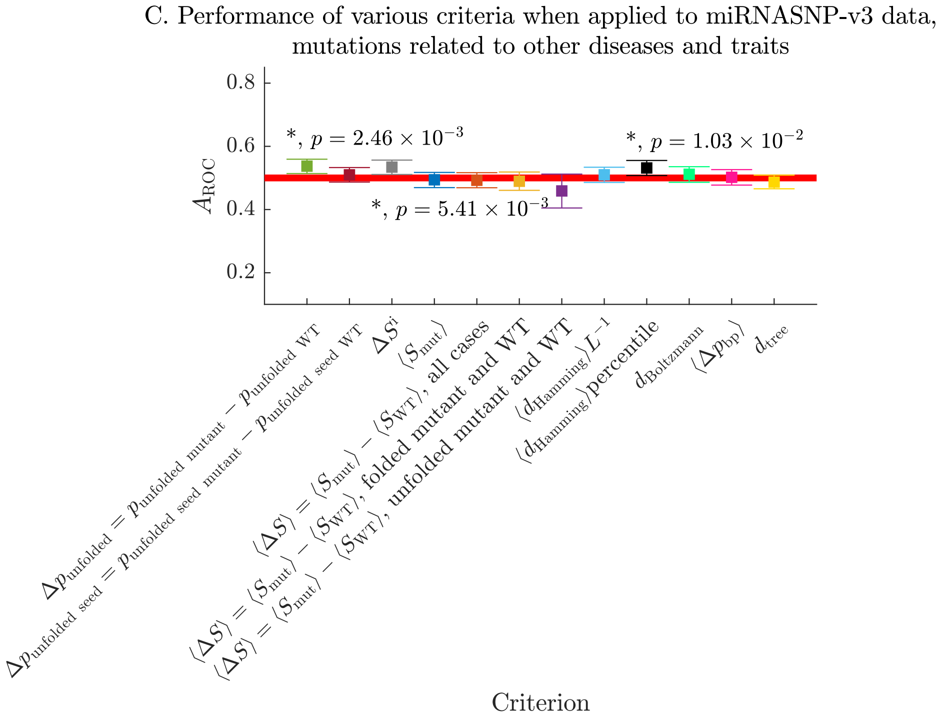

Figure S14: **Two independent SS-based criteria consistently predict association of miRNA mutations with disease better than the random criterion.** Comparison of the performance of various criteria in terms of predicting disease-associated mutations when applied to data from SomamiR (A), data on cancer-related mutations (B) and mutations related to other traits and diseases (C) from miRNASNP-v3 with no filtering based on $N_{\mathrm{mut\ seed}}$ and $N_{\mathrm{mut\ non-seed}}$. Squares mark A_{ROC} values, error bars show 95% confidence intervals, and Mann-Whitney p-values are indicated wherever $p < 0.05$. The red horizontal lines indicate the area under the curve for the random criterion, $A_{\mathrm{ROC}} = 0.5$. The criterion that performs significantly better than random for all datasets is $\Delta p_{\mathrm{unfolded}}$, which measures the change in probability that the miRNA is fully unfolded. For miRNASNP-v3 data, A_{ROC} is also significantly greater 0.5 if mutants are instead ranked in ascending order of their percentile in the $\langle d_{\mathrm{Hamming}} \rangle$ distribution. This means that, within the two miRNASNP-v3 datasets, mutations which change miRNA secondary structure less tend to be associated with disease.

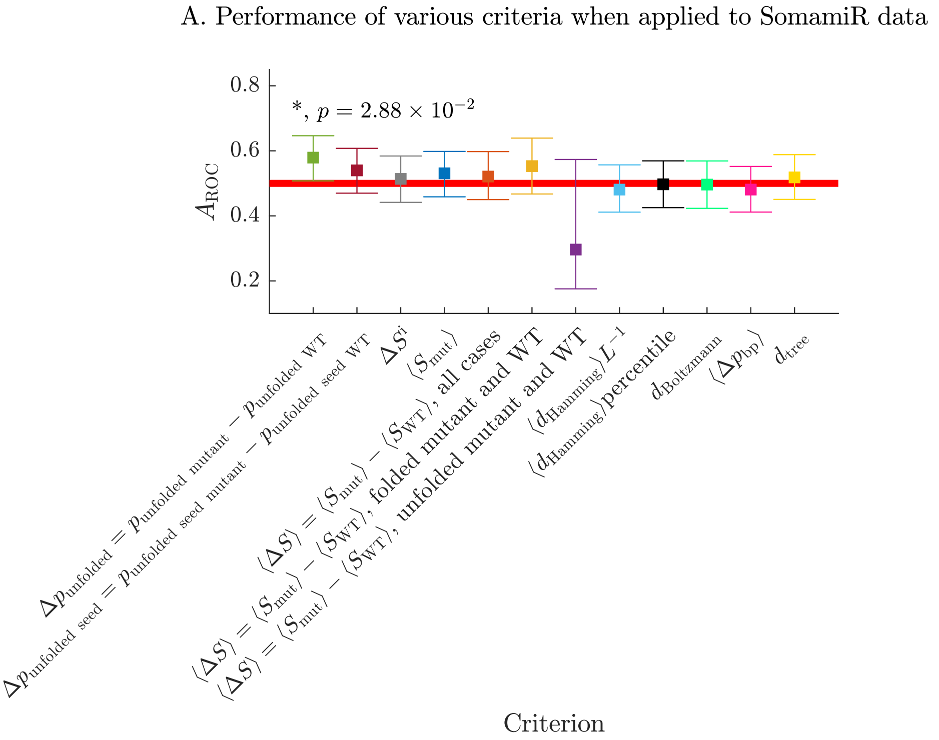
A. Performance of various criteria when applied to SomamiR data
0.8
0.6
0.4
0.2
A_{ROC}
*, $p = 2.88 \times 10^{-2}$
$\Delta p_{\text{unfolded}} = p_{\text{unfolded mutant}} - p_{\text{unfolded WT}}$
$\Delta p_{\text{unfolded seed}} = p_{\text{unfolded seed mutant}} - p_{\text{unfolded seed WT}}$
ΔS^{i}
$\langle S_{\mathrm{mut}} \rangle$
$\langle \Delta S \rangle = \langle S_{\mathrm{mut}} \rangle - \langle S_{\mathrm{WT}} \rangle$, all cases
$\langle \Delta S \rangle = \langle S_{\mathrm{mut}} \rangle - \langle S_{\mathrm{WT}} \rangle$, folded mutant and WT
$\langle \Delta S \rangle = \langle S_{\mathrm{mut}} \rangle - \langle S_{\mathrm{WT}} \rangle$, unfolded mutant and WT
$\langle d_{\mathrm{Hamming}} \rangle L^{-1}$
$\langle d_{\mathrm{Hamming}} \rangle$percentile
$d_{\mathrm{Boltzmann}}$
$\langle \Delta p_{\mathrm{bp}} \rangle$
d_{tree}
Criterion

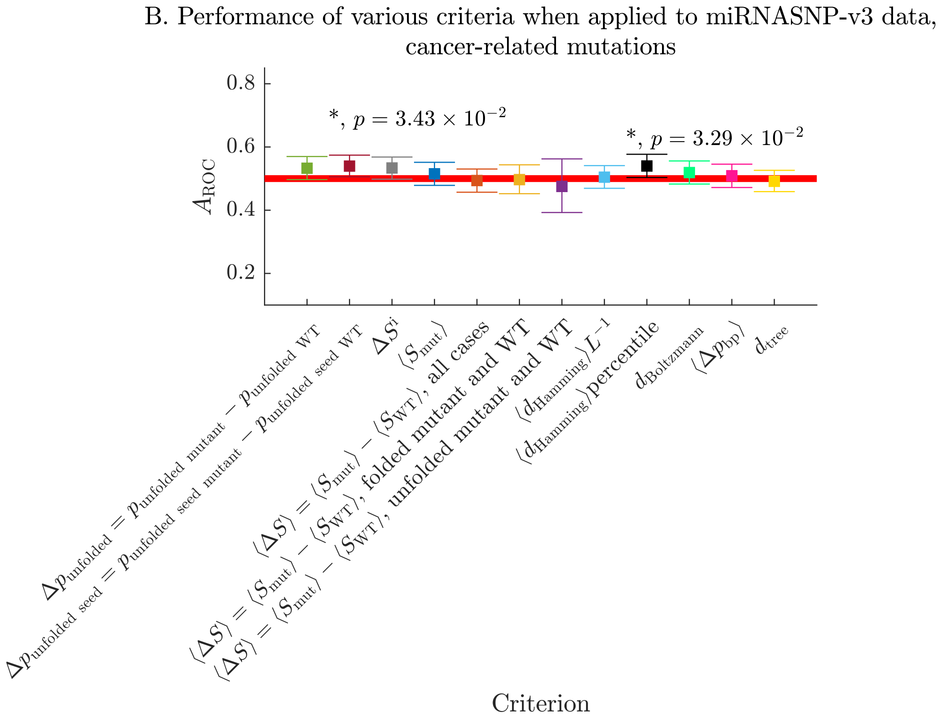
B. Performance of various criteria when applied to miRNASNP-v3 data, cancer-related mutations
0.8
0.6
0.4
0.2
A_{ROC}
*, $p = 3.43 \times 10^{-2}$
*, $p = 3.29 \times 10^{-2}$
$\Delta p_{\text{unfolded}} = p_{\text{unfolded mutant}} - p_{\text{unfolded WT}}$
$\Delta p_{\text{unfolded seed}} = p_{\text{unfolded seed mutant}} - p_{\text{unfolded seed WT}}$
ΔS^{i}
$\langle S_{\mathrm{mut}} \rangle$
$\langle \Delta S \rangle = \langle S_{\mathrm{mut}} \rangle - \langle S_{\mathrm{WT}} \rangle$, all cases
$\langle \Delta S \rangle = \langle S_{\mathrm{mut}} \rangle - \langle S_{\mathrm{WT}} \rangle$, folded mutant and WT
$\langle \Delta S \rangle = \langle S_{\mathrm{mut}} \rangle - \langle S_{\mathrm{WT}} \rangle$, unfolded mutant and WT
$\langle d_{\mathrm{Hamming}} \rangle L^{-1}$
$\langle d_{\mathrm{Hamming}} \rangle$percentile
$d_{\mathrm{Boltzmann}}$
$\langle \Delta p_{\mathrm{bp}} \rangle$
d_{tree}
Criterion

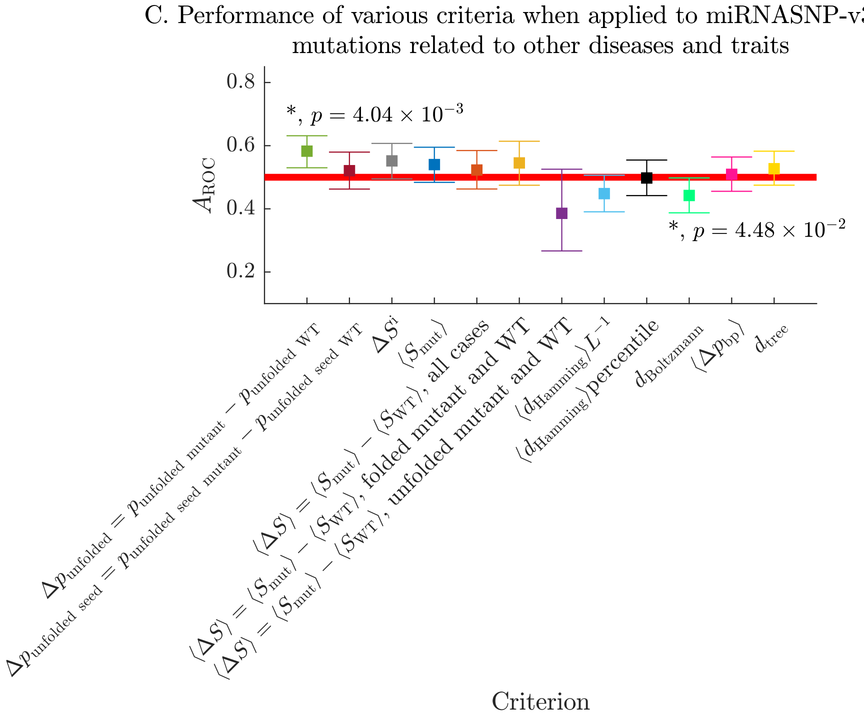

Figure S15: Comparison of the performance of various criteria in terms of predicting disease-associated mutations when applied to data from SomamiR and miRNASNP-v3 with $N_{\mathrm{mut\ seed}} \geq 1$ and $N_{\mathrm{mut\ non-seed}} \geq 1$. Squares mark A_{ROC} values, error bars show 95% confidence intervals, and p-values are indicated wherever $p < 0.05$. The red horizontal line indicates the area under the curve for the random criterion, $A_{\mathrm{ROC}} = 0.5$.

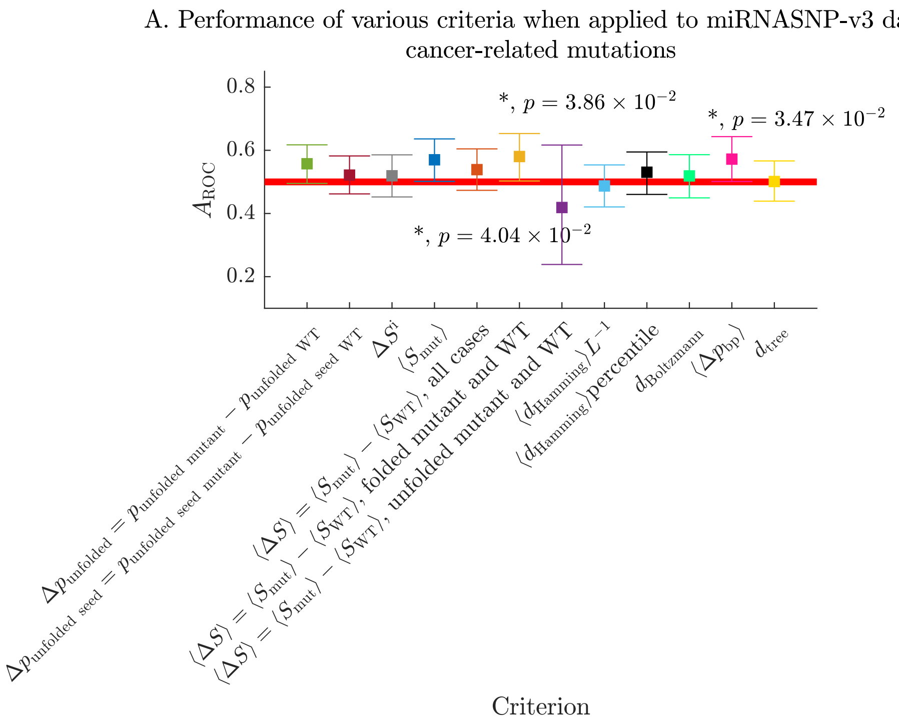

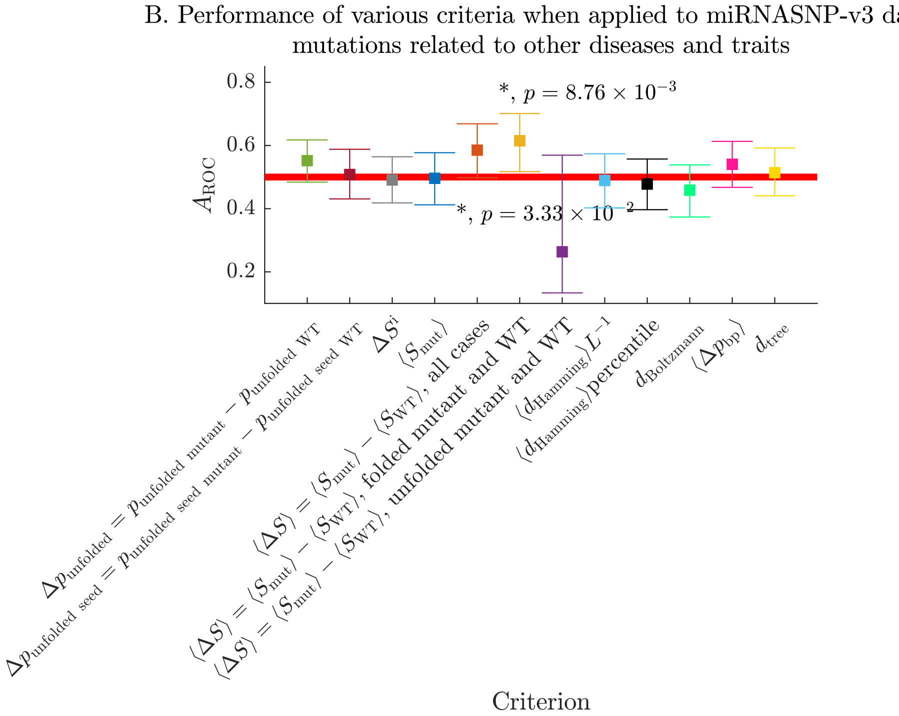

Figure S16: Comparison of the performance of various criteria in terms of predicting disease-associated mutations when applied to data from miRNASNP-v3 with $N_{\mathrm{mut\ seed}} \geq 2$ and $N_{\mathrm{mut\ non-seed}} \geq 2$. Squares mark A_{ROC} values, error bars show 95% confidence intervals, and p-values are indicated wherever $p < 0.05$. The red horizontal line indicates the area under the curve for the random criterion, $A_{\mathrm{ROC}} = 0.5$.

Table S3: Areas under the ROC curves (A_{ROC}) for various criteria applied to the miRNA entries from SomamiR with no filtering based on $N_{\mathrm{mut\ seed}}$ and $N_{\mathrm{mut\ non-seed}}$, and p-values based on the two-sided Mann-Whitney test. p-values of less than 0.05, which indicate criteria that perform significantly better than the random predictor (with $A_{\mathrm{ROC}} = 0.5$), in **bold**.

Criterion	A_{ROC}	p-value	Number of unique mature miRNAs in the sample
$\Delta p_{\mathrm{unfolded}}$	0.533	$\mathbf{2.93 \times 10^{-2}}$	285
$\Delta p_{\mathrm{unfolded\ seed}}$	0.497	8.68×10^{-1}	285
$\Delta S^{(\mathrm{i})}$	0.530	$\mathbf{4.66 \times 10^{-2}}$	285
$\langle S_{\mathrm{mut}} \rangle$	0.512	4.32×10^{-1}	285
$\langle \Delta S \rangle$, all cases	0.487	4.11×10^{-1}	285
$\langle \Delta S \rangle$, folded mutant and WT	0.499	9.51×10^{-1}	215
$\langle \Delta S \rangle$, unfolded mutant and WT	0.460	2.90×10^{-1}	70
$\langle d_{\mathrm{Hamming}} \rangle L^{-1}$	0.496	8.00×10^{-1}	285
$\langle d_{\mathrm{Hamming}} \rangle$ percentile	0.521	1.62×10^{-1}	285
$d_{\mathrm{Boltzmann}}$	0.486	3.65×10^{-1}	285
$\langle \Delta p_{\mathrm{bp}} \rangle$	0.498	8.76×10^{-1}	285
d_{tree}	0.491	5.35×10^{-1}	285

Table S4: Results for various criteria applied to the entries from miRNASNP-v3 related to cancer; no filtering by $N_{\text{mut seed}}$ and $N_{\text{mut non-seed}}$ is applied. The table contains areas under the ROC curves (A_{ROC}), and p-values based on the two-sided Mann-Whitney test. p-values of less than 0.05, which indicate criteria that perform significantly better than the random predictor (with $A_{\text{ROC}} = 0.5$), in **bold**.

Criterion	A_{ROC}	p-value	Number of unique mature miRNAs in the sample
$\Delta p_{\text{unfolded}}$	0.542	$\mathbf{5.47 \times 10^{-6}}$	675
$\Delta p_{\text{unfolded seed}}$	0.504	6.34×10^{-1}	675
$\Delta S^{(\text{i})}$	0.528	$\mathbf{2.62 \times 10^{-3}}$	675
$\langle S_{\text{mut}} \rangle$	0.510	2.57×10^{-1}	675
$\langle \Delta S \rangle$, all cases	0.492	3.90×10^{-1}	675
$\langle \Delta S \rangle$, folded mutant and WT	0.495	6.76×10^{-1}	512
$\langle \Delta S \rangle$, unfolded mutant and WT	0.474	2.24×10^{-1}	163
$\langle d_{\text{Hamming}} \rangle L^{-1}$	0.516	7.83×10^{-2}	675
$\langle d_{\text{Hamming}} \rangle$ percentile	0.548	$\mathbf{1.61 \times 10^{-7}}$	675
$d_{\text{Boltzmann}}$	0.539	$\mathbf{2.18 \times 10^{-5}}$	675
$\langle \Delta p_{\text{bp}} \rangle$	0.486	1.41×10^{-1}	675
d_{tree}	0.479	$\mathbf{1.11 \times 10^{-2}}$	675

Table S5: Results for various criteria applied to the entries from miRNASNP-v3 related to diseases other than cancer; no filtering by $N_{\text{mut seed}}$ and $N_{\text{mut non-seed}}$ is applied. The table contains areas under the ROC curves (A_{ROC}), and p-values based on the two-sided Mann-Whitney test. p-values of less than 0.05, which indicate criteria that perform significantly better than the random predictor (with $A_{\text{ROC}} = 0.5$), in **bold**.

Criterion	A_{ROC}	p-value	Number of unique mature miRNAs in the sample
$\Delta p_{\text{unfolded}}$	0.537	$\mathbf{2.46 \times 10^{-3}}$	400
$\Delta p_{\text{unfolded seed}}$	0.510	4.28×10^{-1}	400
$\Delta S^{(\text{i})}$	0.534	$\mathbf{5.41 \times 10^{-3}}$	400
$\langle S_{\text{mut}} \rangle$	0.494	6.33×10^{-1}	400
$\langle \Delta S \rangle$, all cases	0.493	5.47×10^{-1}	400
$\langle \Delta S \rangle$, folded mutant and WT	0.490	4.75×10^{-1}	292
$\langle \Delta S \rangle$, unfolded mutant and WT	0.459	1.36×10^{-1}	108
$\langle d_{\text{Hamming}} \rangle L^{-1}$	0.510	4.21×10^{-1}	400
$\langle d_{\text{Hamming}} \rangle$ percentile	0.532	$\mathbf{1.04 \times 10^{-2}}$	400
$d_{\text{Boltzmann}}$	0.512	3.41×10^{-1}	400
$\langle \Delta p_{\text{bp}} \rangle$	0.502	8.69×10^{-1}	400
d_{tree}	0.487	2.44×10^{-1}	400

Table S6: Areas under the ROC curves (A_{ROC}) for various criteria applied to the miRNA entries from SomamiR for which $N_{\mathrm{mut\ seed}} \geq 1$ and $N_{\mathrm{mut\ non-seed}} \geq 1$, and p-values based on the two-sided Mann-Whitney test. p-values of less than 0.05, which indicate criteria that perform significantly better than the random predictor (with $A_{\mathrm{ROC}} = 0.5$), in **bold**.

Criterion	A_{ROC}	p-value	Number of unique mature miRNAs in the sample
$\Delta p_{\mathrm{unfolded}}$	0.579	$\mathbf{2.88 \times 10^{-2}}$	40
$\Delta p_{\mathrm{unfolded\ seed}}$	0.540	2.70×10^{-1}	40
$\Delta S^{(\mathrm{i})}$	0.514	7.04×10^{-1}	40
$\langle S_{\mathrm{mut}} \rangle$	0.531	3.93×10^{-1}	40
$\langle \Delta S \rangle$, all cases	0.521	5.64×10^{-1}	40
$\langle \Delta S \rangle$, folded mutant and WT	0.553	2.16×10^{-1}	31
$\langle \Delta S \rangle$, unfolded mutant and WT	0.296	6.59×10^{-2}	9
$\langle d_{\mathrm{Hamming}} \rangle L^{-1}$	0.481	5.96×10^{-1}	40
$\langle d_{\mathrm{Hamming}} \rangle$ percentile	0.497	9.36×10^{-1}	40
$d_{\mathrm{Boltzmann}}$	0.496	9.16×10^{-1}	40
$\langle \Delta p_{\mathrm{bp}} \rangle$	0.481	5.96×10^{-1}	40
d_{tree}	0.518	5.90×10^{-1}	40

Table S7: Results for various criteria applied to the entries from miRNASNP-v3 related to cancer for which $N_{\text{mut seed}} \geq 1$ and $N_{\text{mut non-seed}} \geq 1$. The table contains areas under the ROC curves (A_{ROC}), and p-values based on the two-sided Mann-Whitney test. p-values of less than 0.05, which indicate criteria that perform significantly better than the random predictor (with $A_{\text{ROC}} = 0.5$), in **bold**.

Criterion	A_{ROC}	p-value	Number of unique mature miRNAs in the sample
$\Delta p_{\text{unfolded}}$	0.533	7.94×10^{-2}	143
$\Delta p_{\text{unfolded seed}}$	0.540	$\mathbf{3.44 \times 10^{-2}}$	143
$\Delta S^{(\text{i})}$	0.534	7.24×10^{-2}	143
$\langle S_{\text{mut}} \rangle$	0.515	4.18×10^{-1}	143
$\langle \Delta S \rangle$, all cases	0.494	7.42×10^{-1}	143
$\langle \Delta S \rangle$, folded mutant and WT	0.497	8.85×10^{-1}	109
$\langle \Delta S \rangle$, unfolded mutant and WT	0.475	5.85×10^{-1}	34
$\langle d_{\text{Hamming}} \rangle L^{-1}$	0.505	8.09×10^{-1}	143
$\langle d_{\text{Hamming}} \rangle$ percentile	0.540	$\mathbf{3.27 \times 10^{-2}}$	143
$d_{\text{Boltzmann}}$	0.519	3.08×10^{-1}	143
$\langle \Delta p_{\text{bp}} \rangle$	0.509	6.31×10^{-1}	143
d_{tree}	0.491	6.20×10^{-1}	143

Table S8: Results for various criteria applied to the entries from miRNASNP-v3 related to diseases other than cancer for which $N_{\mathrm{mut\ seed}} \geq 1$ and $N_{\mathrm{mut\ non-seed}} \geq 1$. The table contains areas under the ROC curves (A_{ROC}), and p-values based on the two-sided Mann-Whitney test. p-values of less than 0.05, which indicate criteria that perform significantly better than the random predictor (with $A_{\mathrm{ROC}} = 0.5$), in **bold**.

Criterion	A_{ROC}	p-value	Number of unique mature miRNAs in the sample
$\Delta p_{\mathrm{unfolded}}$	0.583	$\mathbf{4.04 \times 10^{-3}}$	46
$\Delta p_{\mathrm{unfolded\ seed}}$	0.521	4.70×10^{-1}	46
$\Delta S^{(\mathrm{i})}$	0.552	7.16×10^{-2}	46
$\langle S_{\mathrm{mut}} \rangle$	0.540	1.63×10^{-2}	46
$\langle \Delta S \rangle$, all cases	0.523	4.27×10^{-1}	46
$\langle \Delta S \rangle$, folded mutant and WT	0.545	1.75×10^{-1}	32
$\langle \Delta S \rangle$, unfolded mutant and WT	0.386	8.31×10^{-2}	14
$\langle d_{\mathrm{Hamming}} \rangle L^{-1}$	0.448	7.17×10^{-2}	46
$\langle d_{\mathrm{Hamming}} \rangle$ percentile	0.498	9.35×10^{-1}	46
$d_{\mathrm{Boltzmann}}$	0.442	$\mathbf{4.48 \times 10^{-2}}$	46
$\langle \Delta p_{\mathrm{bp}} \rangle$	0.509	7.48×10^{-1}	46
d_{tree}	0.527	3.06×10^{-1}	46

Table S9: Results for various criteria applied to the entries from miRNASNP-v3 related to cancer for which $N_{\text{mut seed}} \geq 2$ and $N_{\text{mut non-seed}} \geq 2$. The table contains areas under the ROC curves (A_{ROC}), and p-values based on the two-sided Mann-Whitney test. p-values of less than 0.05, which indicate criteria that perform significantly better than the random predictor (with $A_{\text{ROC}} = 0.5$), in **bold**.

Criterion	A_{ROC}	p-value	Number of unique mature miRNAs in the sample
$\Delta p_{\text{unfolded}}$	0.557	9.26×10^{-2}	23
$\Delta p_{\text{unfolded seed}}$	0.521	5.29×10^{-1}	23
$\Delta S^{(\text{i})}$	0.519	5.71×10^{-1}	23
$\langle S_{\text{mut}} \rangle$	0.570	$\mathbf{4.04 \times 10^{-2}}$	23
$\langle \Delta S \rangle$, all cases	0.539	2.56×10^{-1}	23
$\langle \Delta S \rangle$, folded mutant and WT	0.580	$\mathbf{3.86 \times 10^{-2}}$	18
$\langle \Delta S \rangle$, unfolded mutant and WT	0.419	3.68×10^{-1}	5
$\langle d_{\text{Hamming}} \rangle L^{-1}$	0.487	7.06×10^{-1}	23
$\langle d_{\text{Hamming}} \rangle$ percentile	0.531	3.70×10^{-1}	23
$d_{\text{Boltzmann}}$	0.519	5.80×10^{-1}	23
$\langle \Delta p_{\text{bp}} \rangle$	0.572	$\mathbf{3.47 \times 10^{-2}}$	23
d_{tree}	0.501	9.69×10^{-1}	23

Table S10: Results for various criteria applied to the entries from miRNASNP-v3 related to diseases other than cancer for which $N_{\mathrm{mut\ seed}} \geq 2$ and $N_{\mathrm{mut\ non-seed}} \geq 2$. The table contains areas under the ROC curves (A_{ROC}), and p-values based on the two-sided Mann-Whitney test. p-values of less than 0.05, which indicate criteria that perform significantly better than the random predictor (with $A_{\mathrm{ROC}} = 0.5$), in **bold**.

Criterion	A_{ROC}	p-value	Number of unique mature miRNAs in the sample
$\Delta p_{\mathrm{unfolded}}$	0.552	1.95×10^{-1}	12
$\Delta p_{\mathrm{unfolded\ seed}}$	0.508	8.40×10^{-1}	12
$\Delta S^{(\mathrm{i})}$	0.491	8.19×10^{-1}	12
$\langle S_{\mathrm{mut}} \rangle$	0.496	9.22×10^{-1}	12
$\langle \Delta S \rangle$, all cases	0.585	$\mathbf{3.33 \times 10^{-2}}$	12
$\langle \Delta S \rangle$, folded mutant and WT	0.615	$\mathbf{8.76 \times 10^{-3}}$	9
$\langle \Delta S \rangle$, unfolded mutant and WT	0.263	5.38×10^{-2}	3
$\langle d_{\mathrm{Hamming}} \rangle L^{-1}$	0.489	7.88×10^{-1}	12
$\langle d_{\mathrm{Hamming}} \rangle$ percentile	0.478	5.81×10^{-1}	12
$d_{\mathrm{Boltzmann}}$	0.459	3.01×10^{-1}	12
$\langle \Delta p_{\mathrm{bp}} \rangle$	0.540	3.13×10^{-1}	12
d_{tree}	0.514	7.11×10^{-1}	12

		cancer-related mutants		mutants related to other traits and diseases	
criterion 1	criterion 2	R_{Spearman}	p-value	R_{Spearman}	p-value
$\Delta p_{\text{unfolded}}$	$\langle d_{\text{Hamming}} \rangle$%ile	-0.091	2.57×10^{-27}	-0.066	7.12×10^{-6}
$\Delta p_{\text{unfolded}}$	$\Delta S^{(\text{i})}$	-0.417	≈ 0	-0.415	2.89×10^{-189}
$\Delta p_{\text{unfolded}}$	$\langle \Delta p_{\text{bp}} \rangle$	-0.303	1.35×10^{-298}	-0.313	3.14×10^{-104}
$\Delta S^{(\text{i})}$	$\langle d_{\text{Hamming}} \rangle$%ile	0.330	≈ 0	0.332	1.38×10^{-117}

Table S11: Spearman rank correlation coefficients and associated p-values between the three main criteria and the pair $\Delta p_{\text{unfolded}}$-$\langle \Delta p_{\text{bp}} \rangle$ calculated for miRNASNP-v3 with no filtering based on the number of mutations per miRNA. Most pairs of criteria display weak but significant correlations. Correlations are particularly weak between $\Delta p_{\text{unfolded}}$ and $\langle d_{\text{Hamming}} \rangle$%ile, indicating that the two can be treated as approximately uncorrelated. $\Delta p_{\text{unfolded}}$ is negatively correlated with $\langle \Delta p_{\text{bp}} \rangle$ as one may expect since a greater $\Delta p_{\text{unfolded}}$ means that the unfolded structure is more common in the ensemble and bases throughout the miRNA are more commonly unpaired.

		miRNASNP-v3 mutants, selected miRNAs	
criterion 1	criterion 2	R_{Spearman}	p-value
$\Delta p_{\text{unfolded}}$	$\langle d_{\text{Hamming}} \rangle$%ile	-0.355	2.17×10^{-11}
$\Delta p_{\text{unfolded}}$	$\Delta S^{(\text{i})}$	-0.563	≈ 0
$\Delta p_{\text{unfolded}}$	$\langle \Delta p_{\text{bp}} \rangle$	-0.453	≈ 0
$\Delta S^{(\text{i})}$	$\langle d_{\text{Hamming}} \rangle$%ile	0.406	9.07×10^{-15}

Table S12: Rank correlation coefficient data for a subset of the miRNASNP-v3 database limited to mutations in hsa-miR-485-5p, hsa-miR-1908-3p, hsa-miR-1269b, hsa-miR-4537, hsa-miR-4477b, hsa-miR-4641, hsa-miR-6821-3p. The trends observed are similar to those in Table S11 above.

4 Rank correlations between selected criteria

Tables S11-S13 contain correlation coefficients and associated p-values for the various datasets discussed in the main text.

		SomamiR mutants	
criterion 1	criterion 2	R_{Spearman}	p-value
$\Delta p_{\text{unfolded}}$	$\langle d_{\text{Hamming}} \rangle$%ile	-0.124	8.82×10^{-48}
$\Delta p_{\text{unfolded}}$	$\Delta S^{(\text{i})}$	-0.435	≈ 0
$\Delta p_{\text{unfolded}}$	$\langle \Delta p_{\text{bp}} \rangle$	-0.301	2.70×10^{-283}
$\Delta S^{(\text{i})}$	$\langle d_{\text{Hamming}} \rangle$%ile	0.351	≈ 0

Table S13: Rank correlation coefficient data from the SomamiR database, no filtering according to the number of mutations per miRNA. The trends observed are similar to those in Table S11 above.

5 Tables of p-values and other data for individual miRNAs

Table S14: Results for individual miRNAs from SomamiR for which $\Delta p_{\mathrm{unfolded}}$ performs better than the random criterion ($p < 0.05$). The p-values are based on the two-sided Mann-Whitney test, and the SS of the WT is predicted with ViennaRNA. For each miRNA, we give lists of references connecting it to cancer and other traits and diseases.

miRNA	A_{ROC}	p-value	Predicted SS (WT)	Ref., cancer	Ref., other traits and diseases
hsa-miR-3689b-3p	0.076	3.55×10^{-2}	....		
hsa-miR-19a-3p	0.000	3.92×10^{-2}	..((((......)))).......	[6–8]	[9–11]
hsa-miR-548g-5p	0.082	3.92×10^{-2}	(((((......)))))).......	[12]	
hsa-miR-345-3p	1.000	4.17×10^{-2}	(((((......))))))......	[13]	[14]
hsa-miR-30e-5p	1.000	4.17×10^{-2}	........(((......)))..	[15–19]	[20–22]
hsa-miR-367-3p	1.000	4.17×10^{-2}	.(((((......)))))).....	[23]	

Table S15: Results for individual miRNAs from SomamiR for which $\langle d_{\mathrm{Hamming}}\rangle L^{-1}$ performs better than the random criterion ($p < 0.05$). The p-values are based on the two-sided Mann-Whitney test, and the SS of the WT is predicted with ViennaRNA. For each miRNA, we give lists of references connecting it to cancer and other traits and diseases.

miRNA	A_{ROC}	p-value	Predicted SS (WT)	Ref., cancer	Ref., other traits and diseases
hsa-miR-3939	0.142	1.52×10^{-2}	.....(((..(....)..))).		[24]
hsa-miR-520g-3p	1.000	3.70×10^{-2}	.......((((.......))))..	[25]	
hsa-miR-635	1.000	3.92×10^{-2}	....(((((.((...)).)))))	[26, 27]	
hsa-miR-1185-1-3p	1.000	4.17×10^{-2}	......(((((....)))))..	[28]	[29]
hsa-miR-208a-3p	1.000	4.17×10^{-2}	.....((((((.....))))))		[30, 31]
hsa-miR-887-3p	1.000	4.17×10^{-2}	.....((((......))))...	[32, 33]	[34]
hsa-miR-192-3p	1.000	4.17×10^{-2}	..(((.........)))).....	[35]	[36–38]
hsa-miR-1294	1.000	4.17×10^{-2}	.........((((....)))).	[39, 40]	[41]
hsa-miR-125b-2-3p	1.000	4.17×10^{-2}	((.((((.......)))).)).	[42, 43]	
hsa-miR-485-5p	0.913	4.43×10^{-2}	...((....))...........	[44–56]	[57]

Table S16: miRNAs for which the combined p-values from the $\Delta p_{\mathrm{unfolded}}$ and $\langle d_{\mathrm{Hamming}} \rangle L^{-1}$ criteria is below 0.05 based on data from SomamiR. miRNA identifiers in *italics* if the p-value for $\Delta p_{\mathrm{unfolded}}$ is also less than 0.05 and **bold** if the p-value for $\langle d_{\mathrm{Hamming}} \rangle L^{-1}$ is less than 0.05. As elsewhere, p-values are based on the two-sided Mann-Whitney test.

miRNA	combined p-value	Predicted SS (WT)	Ref., cancer	Ref., other traits and diseases
hsa-miR-3689b-3p	2.62×10^{-2}	....		
**hsa-miR-1185-1-3p**	3.26×10^{-2}	......(((((....)))))..	[28]	[29]
hsa-miR-558	3.29×10^{-2}	....	[58]	[59]
hsa-miR-129-1-3p	4.15×10^{-2}	....	[60]	[61]
hsa-miR-129-2-3p	4.15×10^{-2}	....	[62]	[61, 63–65]
**hsa-miR-3939**	4.16×10^{-2}	.....(((..(....)..))).		[24]
hsa-miR-543	4.29×10^{-2}	....	[66]	[66, 67]
**hsa-miR-208a-3p**	4.99×10^{-2}	.....((((((.....))))))		[30, 31]
**hsa-miR-887-3p**	4.99×10^{-2}	.....((((......))))...	[32, 33]	[34]

Table S17: Results for individual miRNAs from miRNASNP-v3 for which $\Delta p_{\text{unfolded}}$ performs better than the random criterion ($p < 0.05$). The p-values are based on the two-sided Mann-Whitney test, and the SS of the WT is predicted with ViennaRNA. For each miRNA, we give lists of references connecting it to cancer and other traits and diseases. The names of the miRNAs for which the $\langle d_{\text{Hamming}} \rangle L^{-1}$ criterion also performs significantly better than the random one are in **bold**.

miRNA	A_{ROC}	p-value	Predicted SS (WT)	Ref., cancer	Ref., other traits and diseases
**hsa-miR-3150a-5p**	0.000	1.77×10^{-3}	....		[68]
hsa-miR-345-3p	1.000	1.77×10^{-3}	(((((......)))))......	[13]	[14]
hsa-miR-580-5p	0.000	1.77×10^{-3}	...(((.((....)).)))...	[69]	
**hsa-miR-4641**	0.990	2.80×10^{-3}	....	[70]	
hsa-miR-519a-3p	0.052	3.58×10^{-3}	.....((((.((...)).))))	[71]	[72, 73]
**hsa-miR-4537**	0.746	5.17×10^{-3}	..(((.((((...)))).))).	[74]	
**hsa-miR-539-5p**	0.933	6.13×10^{-3}	(((.......)))..........	[75–78]	
hsa-miR-4756-3p	0.022	7.09×10^{-3}	(((....)))..............	[14]	[79–81]
hsa-miR-6852-5p	0.023	8.08×10^{-3}	.(((.((...)).)))....	[82]	
**hsa-miR-1269b**	0.033	1.06×10^{-2}	.(((......)))..........	[83–85]	
hsa-miR-548i	0.864	1.33×10^{-2}	..........(((.....))).	[86]	[87]
hsa-miR-548l	0.957	1.60×10^{-2}	....	[88]	[89, 90]
hsa-miR-208a-3p	0.054	2.13×10^{-2}	.....((((((.....))))))		[30, 31]
hsa-miR-6888-3p	0.942	2.42×10^{-2}	....		
hsa-miR-4649-3p	0.942	2.42×10^{-2}	...((((....))))......		[91, 92]
**hsa-miR-6821-3p**	0.930	3.23×10^{-2}	....		[93]
hsa-miR-4518	0.860	3.36×10^{-2}	.(((((.(..(....)..).))))).	[94, 95]	[96]
**hsa-miR-19a-5p**	0.924	3.55×10^{-2}	.((..((((....))))..)).	[97]	[98, 99]
hsa-miR-190b-5p	0.076	3.55×10^{-2}	.(((((.....)))))......	[100]	[101]
hsa-miR-1307-3p	0.924	3.55×10^{-2}	..((((((...)))))).....		
hsa-miR-362-5p	1.000	3.70×10^{-2}	...(((........))).......	[102]	
hsa-miR-608	0.918	3.76×10^{-2}	.(((((.((......)).)))))..	[103–109]	[110, 111]
hsa-miR-323b-5p	0.854	3.90×10^{-2}	.((....))((((.....)))).		[112, 113]
hsa-miR-548g-5p	0.082	3.92×10^{-2}	(((((......))))).......	[12]	
hsa-miR-657	0.000	3.92×10^{-2}	((.(((.......))).))....	[114, 115]	
hsa-miR-19b-2-5p	1.000	4.17×10^{-2}	.....((((....))))....		[116]
hsa-miR-3116	1.000	4.17×10^{-2}	..((((........))))....	[117]	
hsa-miR-30e-5p	1.000	4.17×10^{-2}	........(((......)))..	[15–19]	[20–22]
hsa-miR-367-3p	1.000	4.17×10^{-2}	.(((((......)))))....	[23]	
hsa-miR-382-5p	1.000	4.17×10^{-2}	(((....)))..............	[118–122]	[123, 124]
**hsa-miR-558**	0.081	4.32×10^{-2}	....	[58]	[59]
hsa-miR-492	0.847	4.40×10^{-2}	.(..((...))..).........	[125–131]	
hsa-miR-518b	0.087	4.43×10^{-2}	....((.(((.......)))))		[132]
**hsa-miR-1307-5p**	1.000	4.44×10^{-2}	....((((.......))))..		
**hsa-miR-6893-5p**	1.000	4.44×10^{-2}	....	[133]	
**hsa-miR-892c-5p**	1.000	4.44×10^{-2}	....		
hsa-miR-520b-3p	1.000	4.44×10^{-2}	.........((((...)))).	[134]	
hsa-miR-8088	1.000	4.44×10^{-2}	((((...........))))..		[135]
hsa-miR-3120-5p	1.000	4.44×10^{-2}	......(((((.....)))))	[136]	
hsa-miR-3150b-3p	0.000	4.44×10^{-2}	(((.(....).))).......	[137]	[138–140]
hsa-miR-563	0.807	4.64×10^{-2}	.((..(((....))).)).		[141–143]
hsa-miR-3689b-3p	0.156	4.69×10^{-2}	....		
**hsa-miR-1227-3p**	1.000	4.76×10^{-2}	....		[144–146]
**hsa-miR-6794-3p**	0.000	4.76×10^{-2}	....		[147]

Table S18: Results for individual miRNAs from miRNASNP-v3 for which $\langle d_{\mathrm{Hamming}} \rangle L^{-1}$ performs better than the random criterion ($p < 0.05$). The p-values are based on the two-sided Mann-Whitney test, and the SS of the WT is predicted with ViennaRNA. For each miRNA, we give lists of references connecting it to cancer and other traits and diseases. The names of the miRNAs for which the $\Delta p_{\mathrm{unfolded}}$ criterion also performs significantly better than the random one are in **bold**.

miRNA	A_{ROC}	p-value	Predicted SS (WT)	Ref., cancer	Ref., other traits and diseases
hsa-miR-1908-3p	0.939	1.26×10^{-3}	((((...))))..........		
hsa-miR-4477b	1.000	1.77×10^{-3}	(((((..((....))..)))))	[148]	
hsa-miR-4722-3p	1.000	1.77×10^{-3}	..((((.((.......))))))	[149]	[150]
**hsa-miR-1269b**	0.000	1.77×10^{-3}	.(((......))).........	[83–85]	
hsa-miR-485-5p	0.926	1.99×10^{-3}	...((....))...........	[44–56]	[57]
**hsa-miR-4641**	0.981	5.59×10^{-3}	....	[70]	
**hsa-miR-3150a-5p**	0.022	7.09×10^{-3}	....		[68]
**hsa-miR-19a-5p**	0.043	1.60×10^{-2}	.((..((((....))))..)).	[97]	[98, 99]
**hsa-miR-4537**	0.289	1.76×10^{-2}	..(((.((((...)))).))).	[74]	
hsa-miR-6800-3p	0.047	1.82×10^{-2}	..........(((...)))..		
hsa-miR-99a-3p	0.946	2.13×10^{-2}	...(((((......)))))...		
**hsa-miR-558**	0.054	2.43×10^{-2}	....	[58]	[59]
hsa-miR-3939	0.195	2.49×10^{-2}	.....(((..(....)..))).		[24]
hsa-miR-513c-5p	0.935	2.84×10^{-2}	.((((...))))..........	[151, 152]	[153]
hsa-miR-148b-5p	0.065	2.84×10^{-2}	....((((.........)))).		[154, 155]
hsa-miR-4802-3p	0.071	3.14×10^{-2}	....((((.((...))))))...	[156]	
hsa-miR-1248	1.000	3.17×10^{-2}	.............((((...))))...	[26, 157]	[158]
hsa-miR-509-5p	0.930	3.23×10^{-2}	(((((....)))))).......	[159–161]	[162, 163]
hsa-miR-3130-3p	0.930	3.23×10^{-2}	..(((.((((...))))))).	[164]	[165]
**hsa-miR-6821-3p**	0.930	3.23×10^{-2}	....		[93]
hsa-miR-550a-5p	0.819	3.35×10^{-2}	.........(((........)))	[166]	[167]
hsa-miR-887-3p	0.924	3.55×10^{-2}	.....((((......))))...	[32, 33]	[34]
hsa-miR-624-3p	0.857	3.86×10^{-2}	....(((((.......)))))	[168]	
hsa-miR-103a-1-5p	1.000	3.92×10^{-2}	(((..(........)..)))...		
hsa-miR-302a-5p	1.000	3.92×10^{-2}	........(((....)))....		[169]
hsa-miR-635	1.000	3.92×10^{-2}	....(((((.((...)).)))))	[26, 27]	
**hsa-miR-539-5p**	0.148	4.14×10^{-2}	(((.......))).........	[75–78]	
hsa-miR-125b-2-3p	1.000	4.17×10^{-2}	((.((((.......)))).)).	[42, 43]	
hsa-miR-1185-1-3p	1.000	4.17×10^{-2}	......(((((....)))))..	[28]	[29]
hsa-miR-3915	1.000	4.17×10^{-2}	...((((........))))...	[170]	
hsa-miR-5195-5p	1.000	4.17×10^{-2}	..((....((....))....))		[171]
hsa-miR-5682	1.000	4.17×10^{-2}	.....((((((.....))))))	[172]	
hsa-miR-199a-3p	0.000	4.17×10^{-2}	.(((....)))...........	[173–176]	[177]
hsa-miR-381-5p	0.000	4.17×10^{-2}	.((((((.....))))))....	[178–180]	
hsa-miR-3689a-3p	0.193	4.30×10^{-2}	(((.((.........)).))).		
hsa-miR-328-5p	0.847	4.40×10^{-2}	....((((.......))))....	[181]	[36]
hsa-miR-500a-3p	0.913	4.43×10^{-2}	.....(((.....)))......	[182, 183]	
hsa-miR-4708-3p	0.913	4.43×10^{-2}	.((......))...........		[184]
hsa-miR-378i	1.000	4.44×10^{-2}	.(((.(((...))))))....	[185–187]	
**hsa-miR-892c-5p**	1.000	4.44×10^{-2}	....		
hsa-miR-3155a	1.000	4.44×10^{-2}	(((.((...))..)))....		[188]
**hsa-miR-6893-5p**	1.000	4.44×10^{-2}	....	[133]	
hsa-miR-376c-3p	0.000	4.44×10^{-2}	........(((...)))....	[189–191]	
hsa-miR-892a	0.000	4.44×10^{-2}	..((((((.......))))))	[192–195]	
**hsa-miR-1307-5p**	0.000	4.44×10^{-2}	....((((.......))))..		
hsa-miR-508-5p	0.803	4.49×10^{-2}	.......(((.......)))...	[196]	[197]
**hsa-miR-1227-3p**	1.000	4.76×10^{-2}	....		[144–146]
**hsa-miR-6794-3p**	0.000	4.76×10^{-2}	....		[147]

Table S19: miRNAs for which the combined p-values from the $\Delta p_{\mathrm{unfolded}}$ and $\langle d_{\mathrm{Hamming}} \rangle L^{-1}$ criteria is below 0.05 based on data from miRNASNP-v3. miRNA identifiers in *italics* if the p-value for $\Delta p_{\mathrm{unfolded}}$ is also less than 0.05, **bold** if the p-value for $\langle d_{\mathrm{Hamming}} \rangle L^{-1}$ is less than 0.05, and ***bold italics*** - if both are below 0.05. As elsewhere, p-values are based via the two-sided Mann-Whitney test.

miRNA	combined p-value	Predicted SS (WT)	Ref., cancer	Ref., other traits and diseases
hsa-miR-3150a-5p	1.54×10^{-4}	....		[68]
hsa-miR-4641	1.89×10^{-4}	....	[70]	
hsa-miR-1269b	2.24×10^{-4}	.(((......))).........	[83–85]	
hsa-miR-4537	9.36×10^{-4}	..(((.((((...)))).))).	[74]	
**hsa-miR-4477b**	1.14×10^{-3}	(((((..((....))..)))))	[148]	
**hsa-miR-1908-3p**	1.69×10^{-3}	((((...))))..........		
hsa-miR-345-3p	1.91×10^{-3}	(((((......)))))......	[13]	[14]
hsa-miR-539-5p	2.35×10^{-3}	(((.......))).........	[75–78]	
**hsa-miR-485-5p**	3.84×10^{-3}	...((....))..........	[44–56]	[57]
**hsa-miR-4722-3p**	3.94×10^{-3}	..((((.((.......))))))	[149]	[150]
hsa-miR-19a-5p	4.80×10^{-3}	.((..((((....))))..)).	[97]	[98, 99]
hsa-miR-548l	6.85×10^{-3}	....	[88]	[89, 90]
hsa-miR-6821-3p	8.22×10^{-3}	....		[93]
hsa-miR-558	8.25×10^{-3}	....	[58]	[59]
hsa-miR-4756-3p	8.81×10^{-3}	(((....)))...........	[14]	[79–81]
hsa-miR-6888-3p	1.11×10^{-2}	....		
hsa-miR-580-5p	1.14×10^{-2}	...(((.((....)).)))...	[69]	
hsa-miR-892c-5p	1.43×10^{-2}	....		
hsa-miR-1307-5p	1.43×10^{-2}	....((((.......))))..		
hsa-miR-6893-5p	1.43×10^{-2}	....	[133]	
hsa-miR-1227-3p	1.61×10^{-2}	....		[144–146]
hsa-miR-6794-3p	1.61×10^{-2}	....		[147]
**hsa-miR-6800-3p**	1.66×10^{-2}	..........(((...)))..		
hsa-miR-4434	2.09×10^{-2}	....		[198]
hsa-miR-519a-3p	2.12×10^{-2}	.....((((.((...)).))))	[71]	[72, 73]
hsa-miR-563	2.29×10^{-2}	.((..(((....))).)).		[141–143]
**hsa-miR-199a-3p**	2.31×10^{-2}	.(((....)))...........	[173–176]	[177]
**hsa-miR-5682**	2.31×10^{-2}	.....((((((.....))))))	[172]	
hsa-miR-4296	2.43×10^{-2}	...(((((....)))))	[199]	[200]
hsa-miR-548i	2.48×10^{-2}	..........(((.....))).	[86]	[87]
hsa-let-7f-1-3p	2.65×10^{-2}	....	[201]	[202–204]
hsa-miR-3689b-3p	2.75×10^{-2}	....		
hsa-miR-492	2.83×10^{-2}	.(..((...))..).........	[125–131]	
hsa-miR-4518	2.87×10^{-2}	.(((((.(..(....)..).))))).	[94, 95]	[96]
hsa-miR-4649-3p	3.09×10^{-2}	...((((....))))......		[91, 92]
**hsa-miR-1185-1-3p**	3.26×10^{-2}	......(((((....)))))..	[28]	[29]
**hsa-miR-4802-3p**	3.37×10^{-2}	....((((.((...))))))...	[156]	
**hsa-miR-513c-5p**	3.43×10^{-2}	.((((...)))).........	[151, 152]	[153]
hsa-miR-208a-3p	3.61×10^{-2}	.....((((((.....))))))		[30, 31]
hsa-miR-6852-5p	3.77×10^{-2}	.(((.((...)).)))....	[82]	
hsa-miR-19b-2-5p	4.15×10^{-2}	.....((((....))))....		[116]
hsa-miR-129-2-3p	4.15×10^{-2}	....	[62]	[61, 63–65]
**hsa-miR-3915**	4.15×10^{-2}	...((((........))))...	[170]	
hsa-miR-4441	4.34×10^{-2}	((((........)))).	[205]	
**hsa-miR-376c-3p**	4.61×10^{-2}	........(((...)))....	[189–191]	
hsa-miR-520b-3p	4.61×10^{-2}	.........((((...)))).	[134]	
**hsa-miR-3130-3p**	4.61×10^{-2}	..(((.((((...)))))))).	[164]	[165]
hsa-miR-6769b-3p	4.61×10^{-2}	....	[206, 207]	
hsa-miR-7151-3p	4.61×10^{-2}	.....((((......))))..		[208]

6 Table of disease-associated mutations that convert one WT miRNA to another

Table S20: Data on mutations that convert one miRNA to another and are associated with disease; data from SomamiR and miRNASNP-v3.

miRNA WT	mutated miRNA	WT sequence	mutated sequence	SS, WT	SS, mutant	$\langle d_{\mathrm{Hamming}} \rangle \times L^{-1}$	$\Delta p_{\mathrm{unfolded}}$
hsa-miR-1269a	hsa-miR-1269b	CUGGACUGAGCCAUGCUACUGG	CUGGACUGAGCCGUGCUACUGG	.(((......)))..........	.(((......)))..........	0.35	-0.0086
hsa-miR-3689a-3p	hsa-miR-3689c, hsa-miR-3689b-3p	CUGGGAGGUGUGAUAUUGUGGU	CUGGGAGGUGUGAUAUCGUGGU	(((.((.........)).))).	....	0.34	0.51
hsa-miR-3689b-3p	hsa-miR-3689a-3p	CUGGGAGGUGUGAUAUCGUGGU	CUGGGAGGUGUGAUAUUGUGGU	....	(((.((.........)).))).	0.34	-0.51
hsa-miR-3689c	has-miR-3689a-3p	CUGGGAGGUGUGAUAUCGUGGU	CUGGGAGGUGUGAUAUUGUGGU	....	(((.((.........)).))).	0.34	-0.51
hsa-miR-518e-5p	hsa-miR-526a-5p, hsa-miR-520c-5p, hsa-miR-518d-5p	CUCUAGAGGGAAGCACUUUCUG	CUCUAGAGGGAAGCGCUUUCUG	...(((((((.....)))))))	...(((((((.....)))))))	0.084	3.2×10^{-4}
hsa-miR-519a-5p	hsa-miR-526a-5p, hsa-miR-520c-5p, hsa-miR-518d-5p	CUCUAGAGGGAAGCACUUUCUG	CUCUAGAGGGAAGCGCUUUCUG	...(((((((.....)))))))	...(((((((.....)))))))	0.084	3.2×10^{-4}
hsa-miR-520c-5p	hsa-miR-519c-5p, hsa-miR-519b-5p, hsa-miR-523-5p, hsa-miR-518e-5p, hsa-miR-522-5p, hsa-miR-519a-5p	CUCUAGAGGGAAGCGCUUUCUG	CUCUAGAGGGAAGCACUUUCUG	...(((((((.....)))))))	...(((((((.....)))))))	0.084	-3.2×10^{-4}
hsa-miR-522-5p	has-miR-526a-5p, hsa-miR-520c-5p, hsa-miR-518d-5p	CUCUAGAGGGAAGCACUUUCUG	CUCUAGAGGGAAGCGCUUUCUG	...(((((((.....)))))))	...(((((((.....)))))))	0.084	3.2×10^{-4}
hsa-miR-548au-5p	has-miR-548ar-5p	AAAAGUAAUUGCAGUUUUUGC	AAAAGUAAUUGCGGUUUUUGC	....	..........(((.....)))	0.26	-0.15
hsa-miR-548au-5p	has-miR-548ay-5p	AAAAGUAAUUGUGGUUUUUGC	AAAAGUAAUUGCGGUUUUUGC	....	....	0.31	0.18
hsa-miR-519a-5p	has-miR-526a-5p, hsa-miR-520c-5p, hsa-miR-518d-5p	CUCUAGAGGGAAGCACUUUCUG	CUCUAGAGGGAAGCGCUUUCUG	...(((((((.....)))))))	...(((((((.....)))))))	0.084	3.2×10^{-4}

Table S21: Results for various criteria applied to the entries from miRNASNP-v3 that concern hsa-miR-4537. The table contains areas under the ROC curves (A_{ROC}), as well as p-values based on the two-sided Mann-Whitney test. p-values of less than 0.05, which indicate criteria that perform significantly better than the random predictor (with $A_{\mathrm{ROC}} = 0.5$), in **bold**.

Criterion	A_{ROC}	p-value
$\Delta p_{\mathrm{unfolded}}$	0.746	$\mathbf{5.17 \times 10^{-3}}$
$\Delta p_{\mathrm{unfolded\ seed}}$	0.509	9.27×10^{-1}
$\Delta S^{(\mathrm{i})}$	0.473	7.70×10^{-1}
$\langle S_{\mathrm{mut}} \rangle$	0.744	$\mathbf{5.57 \times 10^{-3}}$
$\langle \Delta S \rangle$, all cases	0.744	$\mathbf{5.57 \times 10^{-3}}$
$\langle d_{\mathrm{Hamming}} \rangle L^{-1}$	0.289	$\mathbf{1.76 \times 10^{-2}}$
$d_{\mathrm{Boltzmann}}$	0.309	$\mathbf{3.19 \times 10^{-2}}$
$\langle \Delta p_{\mathrm{bp}} \rangle$	0.309	$\mathbf{3.19 \times 10^{-2}}$
d_{tree}	0.665	5.59×10^{-2}

7 Case studies

7.1 hsa-miR-4537

We take the example of one of hsa-miR-4537, one of the individual miRNAs that exhibit a significant effect of secondary structure after adjusting for multiple hypothesis testing. The database contains a total of 20 entries for disease-associated mutations affecting this miRNA, out of which there are 16 unique point mutants with an unaffected seed region.

In Figure S17 we demostrate that all applicable criteria except for $\Delta p_{\mathrm{unfolded}}$ and $\Delta p_{\mathrm{unfolded\ seed}}$ perform significantly better than random for this individual microRNA. We do not show the results for the two criteria based on $\langle d_{\mathrm{Hamming}} \rangle$ separately because the percentile ranking is fully equivalent to the one by the normalized Hamming distance. This likely indicates that, while the effect of secondary structure is by no means the only factor that determines the association of miRNAs with disease, it plays a significant role in some cases. Moreover, hsa-mir-4537 is a tumour-suppressor miRNA relevant to gastric cancer [74], and, as one would expect if the secondary structure affected activity, the mutants that change the folding most tend to be associated with disease, leading to $A_{\mathrm{ROC}} > 0.5$ for $\Delta p_{\mathrm{unfolded}}$ and $A_{\mathrm{ROC}} < 0.5$ for $\langle d_{\mathrm{Hamming}} \rangle L^{-1}$. As one would expect if the secondary structure affected activity, the mutants that change the folding most tend to be associated with disease, leading to $A_{\mathrm{ROC}} > 0.5$ for $\Delta p_{\mathrm{unfolded}}$ and $A_{\mathrm{ROC}} < 0.5$ for $\langle d_{\mathrm{Hamming}} \rangle L^{-1}$. We provide additional data on this miRNA in Figure S18, which contains the ROC curves underlying Figure S17, and Table S21, which gives the values of A_{ROC} for the various criteria.

7.2 hsa-miR-485-5p

We also present the results of applying the various criteria to data for hsa-miR-485-5p, for which the p-value that quantifies the significance of the result for $\langle d_{\mathrm{Hamming}} \rangle / L$ is the lowest ($p = 5.45 \times 10^{-3}$). The miRNASNP-v3 database contains a total of 6 entries for disease-associated mutations affecting this miRNA, out of which there are 4 unique point mutants with an unaffected seed region.

As Figure S19 illustrates, all of our criteria except for $\Delta p_{\mathrm{unfolded}}$ and $\Delta p_{\mathrm{unfolded\ seed}}$ perform significantly better than random for this individual microRNA. This provides further support for the hypothesis that secondary structure impacts the function of some miRNAs. This miRNA is known to act as an inhibitor of breast cancer progression [51]; despite that, it is the mutants with secondary structure close to that of the WT that tend to be associated with disease, as indicated by the A_{ROC} value for $\langle d_{\mathrm{Hamming}} \rangle / L$ in the figure

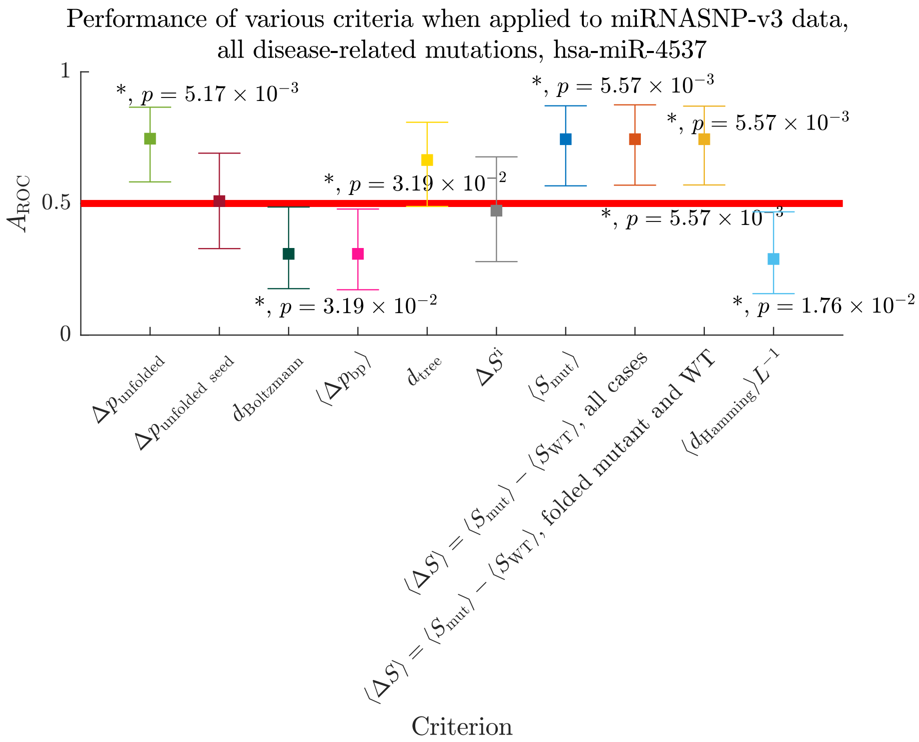

Figure S17: **Multiple independent SS-based criteria predict association of miRNA mutations with disease better than random for hsa-miR-4537.** Comparison of the performance of various criteria in terms of predicting disease-associated mutations when applied to mutations concerning hsa-miR-4537 from miRNASNP-v3. hsa-miR-4537 is known to play a tumour-suppressing role in gastric cancer [74]. Squares mark A_{ROC} values, error bars show 95% confidence intervals calculated with the bootstrapping method [209], and Mann-Whitney p-values are indicated wherever $p < 0.05$. The red horizontal line indicates the area under the curve for the random criterion, $A_{\mathrm{ROC}} = 0.5$. The only criteria that do not perform significantly better than the random one for this dataset are $\Delta p_{\mathrm{unfolded\ seed}}$, which measures the change in probability that the miRNA seed region is fully unfolded and ΔS^{i}, which measures the change in the positional entropy of the mutated site. The comparatively large values of A_{ROC} suggest that miRNA folding plays a role in disease and therefore miRNA activity.

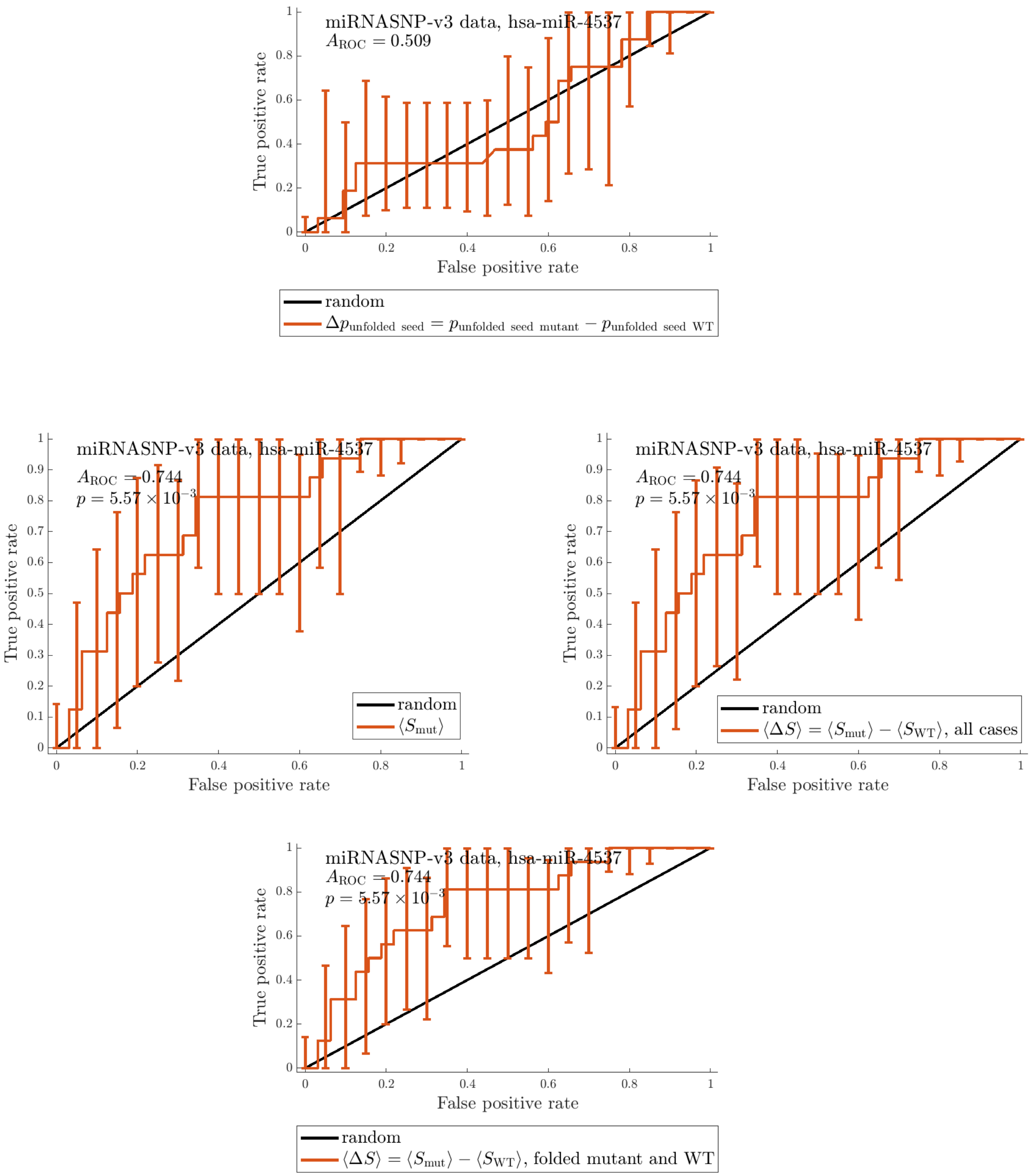

Figure S18: ROC curves characterizing the performance of all criteria discussed above for mutations affecting hsa-miR-4537 from miRNASNP-v3.

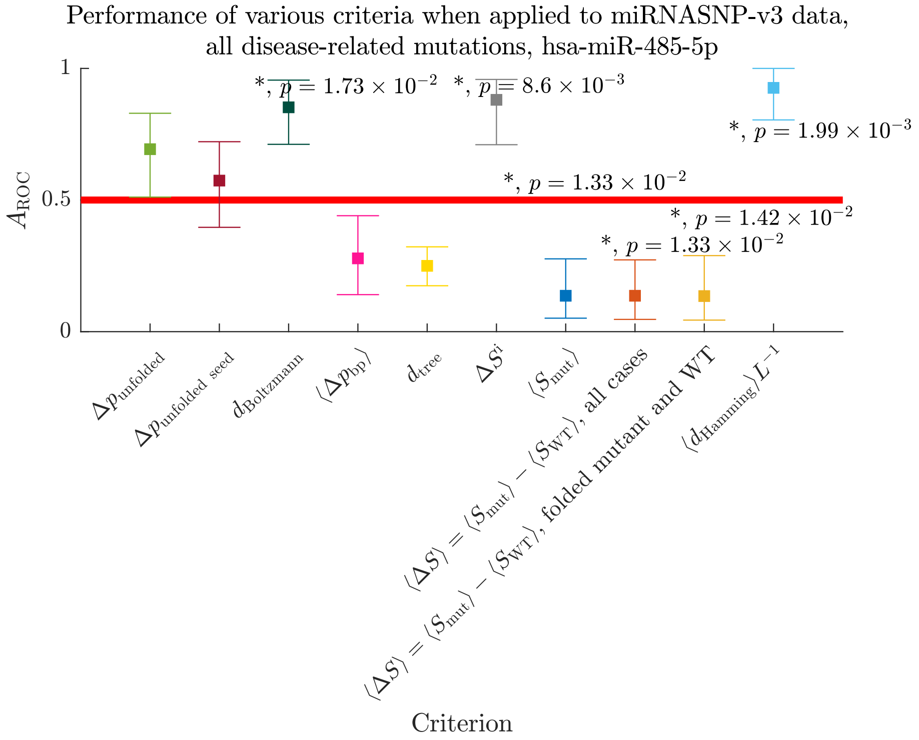

Figure S19: **Multiple independent SS-based criteria predict association of miRNA mutations with disease better than random for hsa-miR-485-5p.** Comparison of the performance of various criteria in terms of predicting disease-associated mutations when applied to mutations concerning hsa-miR-485-5p from miRNASNP-v3. hsa-miR-485-5p is associated with various types of cancer, including lung cancer, breast cancer and others [44–56] and cerebral ischemia [57]. Squares mark A_{ROC} values, error bars show 95% confidence intervals, and Mann-Whitney p-values are indicated wherever $p < 0.05$. The red horizontal lines indicate the area under the curve for the random criterion, $A_{\text{ROC}} = 0.5$. The only criteria that do not perform significantly better than the random one for this dataset are those based on the probability of folded states. The comparatively large values of A_{ROC} suggest that miRNA folding plays a role in disease and therefore miRNA activity.

($A_{\text{ROC}} = 0.926$). We provide additional data on this miRNA in Figure S20, which contains the ROC curves underlying Figure S19, and Table S22, which gives the relevant areas under the ROC curves.

8 Additional information on miRNAs with $q < 0.05$

When we apply the strictest level of filtering, $N_{\text{mut seed}} \geq 3$ and $N_{\text{mut non-seed}} \geq 3$, to the dataset with mutations associated with any traits and diseases, we find one miRNA with a q-value that passes our significance threshold for the criterion based on the mutant-WT SS Hamming distance $\langle d_{\text{Hamming}} \rangle / L$ criterion - $q = 3.72 \times 10^{-2}$ for **hsa-miR-4477b**.

Additionally, we calculate the Benjamini-Hochberg q-values for the combined p-values calculated via the Fisher method [210] for Δp_{folded} and $\langle d_{\text{Hamming}} \rangle / L$ of the individual miRNAs represented in SomamiR and miRNASNP-v3. When we analyse the mutations from miRNASNP-v3 that pertain to all diseases and traits with applied filtering by $N_{\text{mut seed}}$ and $N_{\text{mut non-seed}}$, we find q-values are below 0.05 for several miRNAs. At $N_{\text{mut seed}} \geq 1$ and $N_{\text{mut non-seed}} \geq 1$, $q = 2.67 \times 10^{-2}$ for **hsa-miR-4641** and **hsa-miR-1269b**. For the smaller subset with $N_{\text{mut seed}} \geq 2$ and $N_{\text{mut non-seed}} \geq 2$, $q = 1.86 \times 10^{-2}$ for **hsa-miR-4537** and

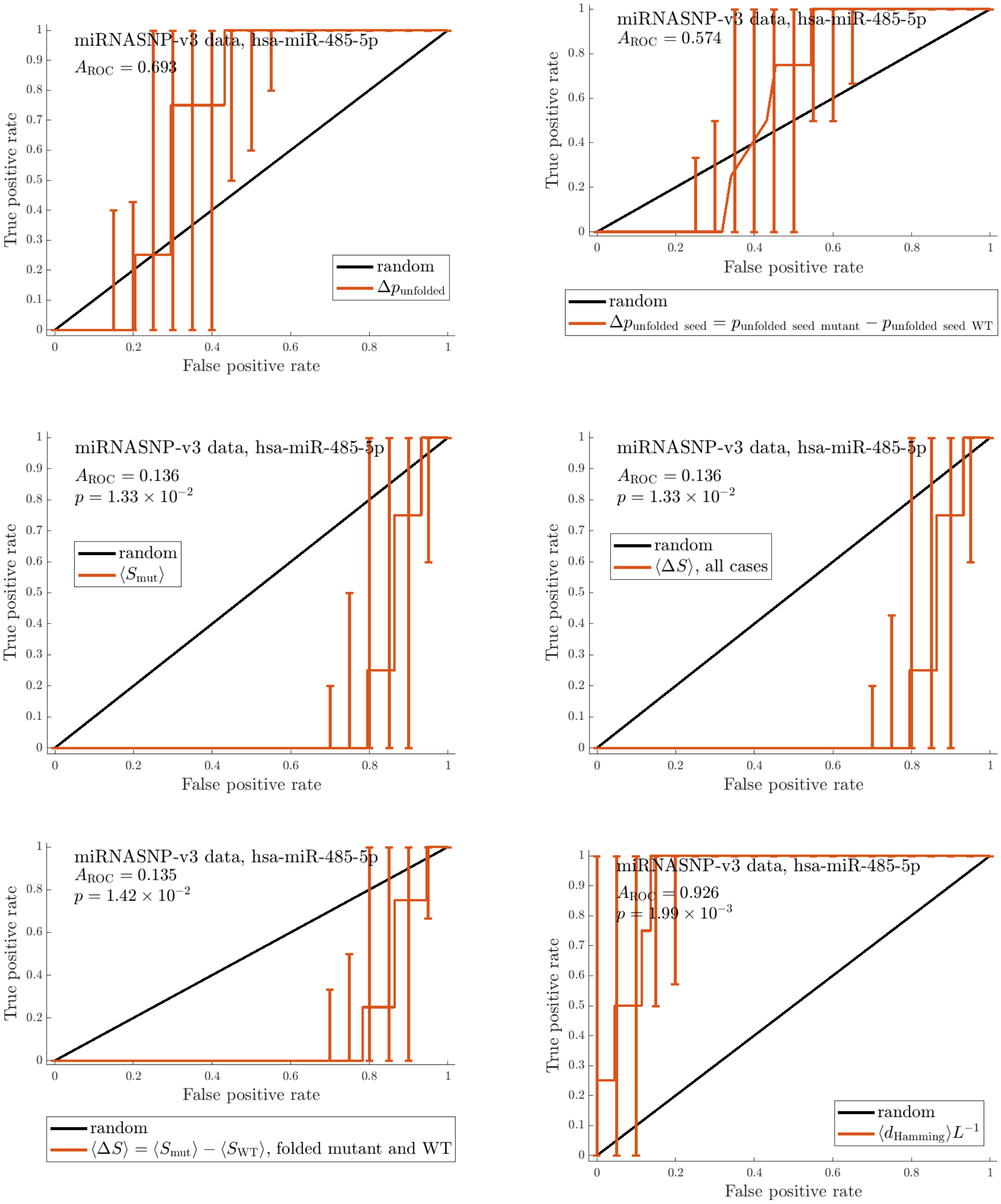

Figure S20: ROC curves characterizing the performance of all criteria discussed above for mutations affecting hsa-miR-485-5p from miRNASNP-v3.

Table S22: Results for various criteria applied to the entries from miRNASNP-v3 that concern hsa-miR-485-5p. The table contains areas under the ROC curves (A_{ROC}), and p-values based on the two-sided Mann-Whitney test. p-values of less than 0.05, which indicate criteria that perform significantly better than the random predictor (with $A_{\text{ROC}} = 0.5$), in **bold**.

Criterion	A_{ROC}	p-value
$\Delta p_{\text{unfolded}}$	0.693	2.21×10^{-1}
$\Delta p_{\text{unfolded seed}}$	0.574	6.47×10^{-1}
$\Delta S^{(\text{i})}$	0.881	$\mathbf{8.60 \times 10^{-3}}$
$\langle S_{\text{mut}} \rangle$	0.136	$\mathbf{1.33 \times 10^{-2}}$
$\langle \Delta S \rangle$, all cases	0.136	$\mathbf{1.33 \times 10^{-2}}$
$\langle d_{\text{Hamming}} \rangle L^{-1}$	0.926	$\mathbf{1.99 \times 10^{-3}}$
$d_{\text{Boltzmann}}$	0.852	$\mathbf{1.73 \times 10^{-2}}$
$\langle \Delta p_{\text{bp}} \rangle$	0.278	1.56×10^{-1}
d_{tree}	0.250	1.54×10^{-1}

hsa-miR-4477b, $q = 1.10 \times 10^{-2}$ for hsa-miR-1269b, and the q-value is further reduced to $q = 1.31 \times 10^{-2}$ for **hsa-miR-4537** and **hsa-miR-4477b** based on the set with $N_{\text{mut seed}} \geq 3$ and $N_{\text{mut non-seed}} \geq 3$.

9 Distributions of $\langle d_{\text{Hamming}} \rangle$ for disease-related mutations in mature miRNAs and their precursors

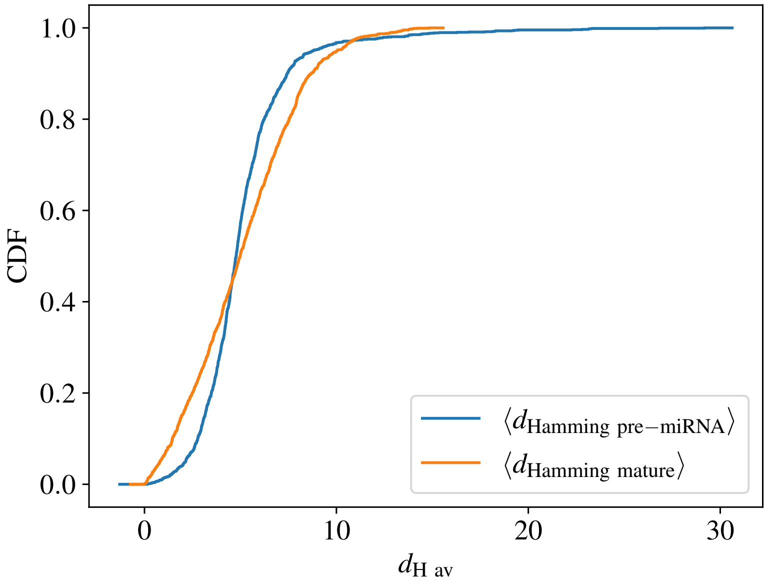

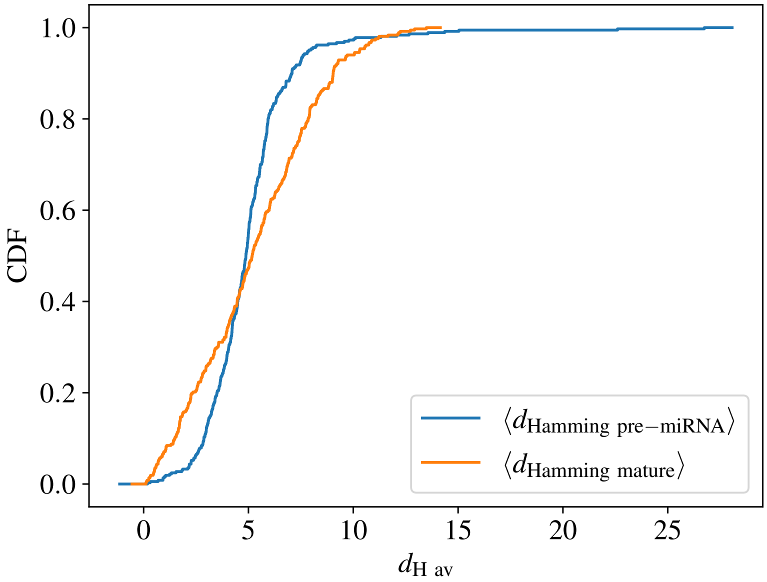

Figure S21: Cumulative distribution functions of $\langle d_{\text{Hamming}} \rangle$ for the disease-related mutations in miRNASNP-v3 (top) and SomamiR (bottom) in mature miRNAs and their precursors. The average Hamming distance is not significantly different between precursors and mature forms, meaning that secondary structure changes in mature forms are greater relative to sequence length.

10 Distributions of $\Delta_{\max}$ for disease-related mutations

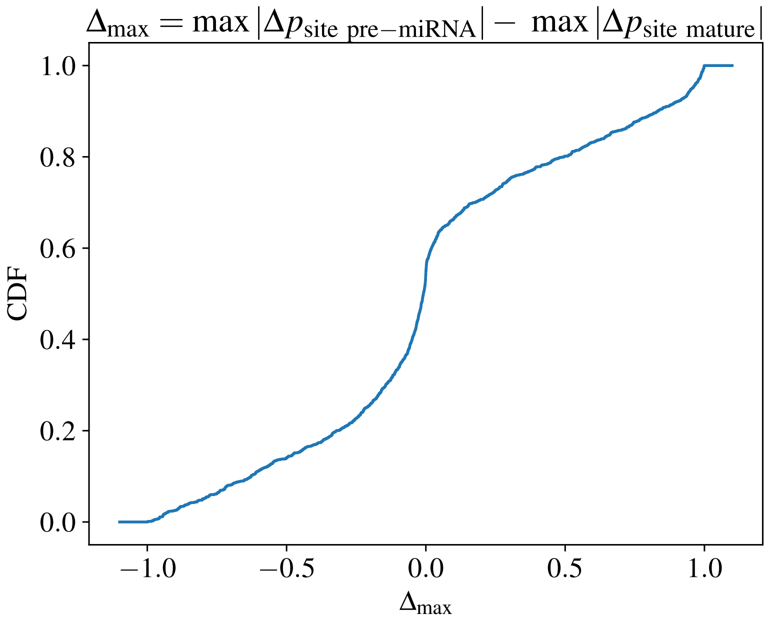

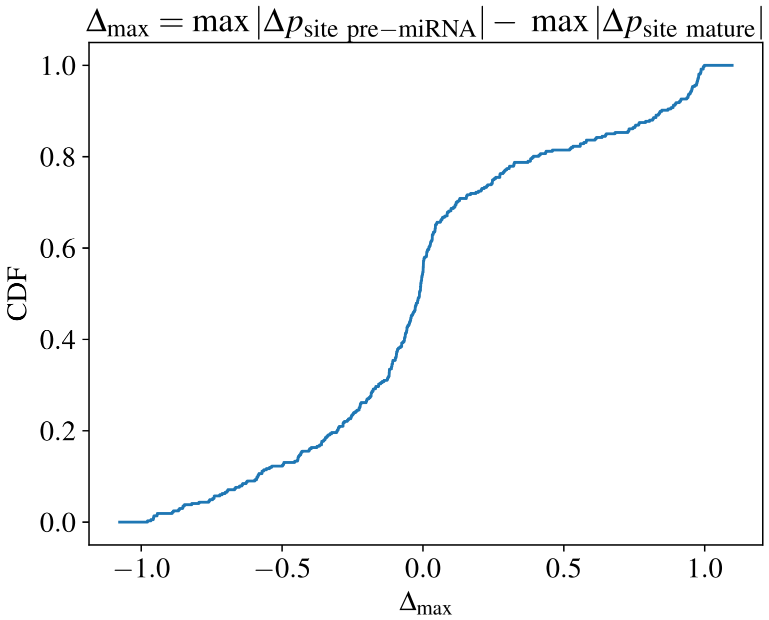

Figure S22: Cumulative distribution functions of $\Delta_{\max}$ for the disease-related mutations in miRNASNP-v3 (top) and SomamiR (bottom) in mature miRNAs and their precursors. Note that for some mutations, $\Delta_{\max} \sim -1$, meaning that the base-pairing at no sensitive site in the precursor is affected, but a site in the mature form is strongly affected.

Table S23: AlphaFold 3 predictions for the secondary structure of miRNA WTs and mutants in complex with Argonaute proteins. plDDT values are averaged over all nucleotides.

ID	sequence	SS	average plDDT
Argonaute 1, mutant hsa-miR-20a 16C>U	UAAAGUGCUUAUAGUGUAGGUAG	....	70.7
Argonaute 1, mutant hsa-miR-20a 8C>A	UAAAGUGAUUAUAGUGCAGGUAG	....	74.2
Argonaute 1, mutant hsa-miR-654-3p	UAUGUCUGCUUACCAUCACCUU	....	72.7
Argonaute 1, mutant hsa-miR-6869-3p	CGCCGCGCGCAGCGGCUCAGC	.(((((.....)))))).....	68.9
Argonaute 1, mutant hsa-miR-6869-3p with maximum $\langle d_{\text{Hamming mutant}} \rangle$	CGCCGCGAGCAUCGGCUCAGC	......((((....))))...	74.2
Argonaute 1, mutant hsa-miR-6869-3p with maximum $\Delta p_{\text{unfolded}}$	CGCCGCGCGCAUCGACUCAGC	....	81.7
Argonaute 2, mutant hsa-let-7c-3p	CUGUACAAGCUUCUAGCUUUCC	....	72.2
Argonaute 2, WT hsa-let-7c-3p	CUGUACAACCUUCUAGCUUUCC	....	88.6
Argonaute 2, mutant hsa-miR-211-5p	UUCCCUUUGGCAUCCUUCGCCU	....	74.3
Argonaute 2, WT hsa-miR-211-5p	UUCCCUUUGUCAUCCUUCGCCU	....	77.1
Argonaute 2, mutant hsa-miR-3144-3p	AUAUACCUGUUCGCUCUCUUUA	....	76.2
Argonaute 2, WT hsa-miR-3144-3p	AUAUACCUGUUCGGUCUCUUUA	....	70.4
Argonaute 2, mutant hsa-miR-574-3p	CACGCUCAUUCACACACCCACA	....	73.5
Argonaute 2, WT hsa-miR-574-3p	CACGCUCAUGCACACACCCACA	....	76.1
Argonaute 2, mutant hsa-miR-769-3p	CUGGGAUCCCCGGGGUCUUGGUU	....	64.7
Argonaute 2, mutant hsa-miR-6794-3p	CUCACUCUGAGUCCCUCCCU	....	73.2
Argonaute 2, mutant hsa-miR-6869-3p	CGCCGCGCGCAGCGGCUCAGC	.(((((.....)))))).....	68.0
Argonaute 2, mutant hsa-miR-6869-3p with maximum $\langle d_{\text{Hamming mutant}} \rangle$	CGCCGCGAGCAUCGGCUCAGC	....((((((....)))).))	76.4
Argonaute 2, mutant hsa-miR-6869-3p with maximum $\Delta p_{\text{unfolded}}$	CGCCGCGCGCAUCGACUCAGC	....	79.7
Argonaute 2, WT hsa-miR-6869-3p	CGCCGCGCGCAUCGGCUCAGC	....	74.2

11 AlphaFold3 studies of miRNA-Argonaute complexes

We used AlphaFold 3 [211] to study the secondary and tertiary structure of complexes between selected miRNAs and the Argonaute 1 and 2 proteins, whose sequences we took from the Protein DataBank entries 4KXT and 4F3T, respectively. We studied several WT miRNAs and point mutants in them, tabulated below. In each case, we took the top-scoring prediction as ranked by AlphaFold 3 and parsed its secondary structure with the DSSR software package [212]. We list the studied miRNA sequences and the predicted secondary structures for them in Table S23. We provide all five AlphaFold 3 predictions for the 3D-structure of the complexes in the form of CIF files with the online supplementary information alongside output from DSSR for the top-rated prediction.

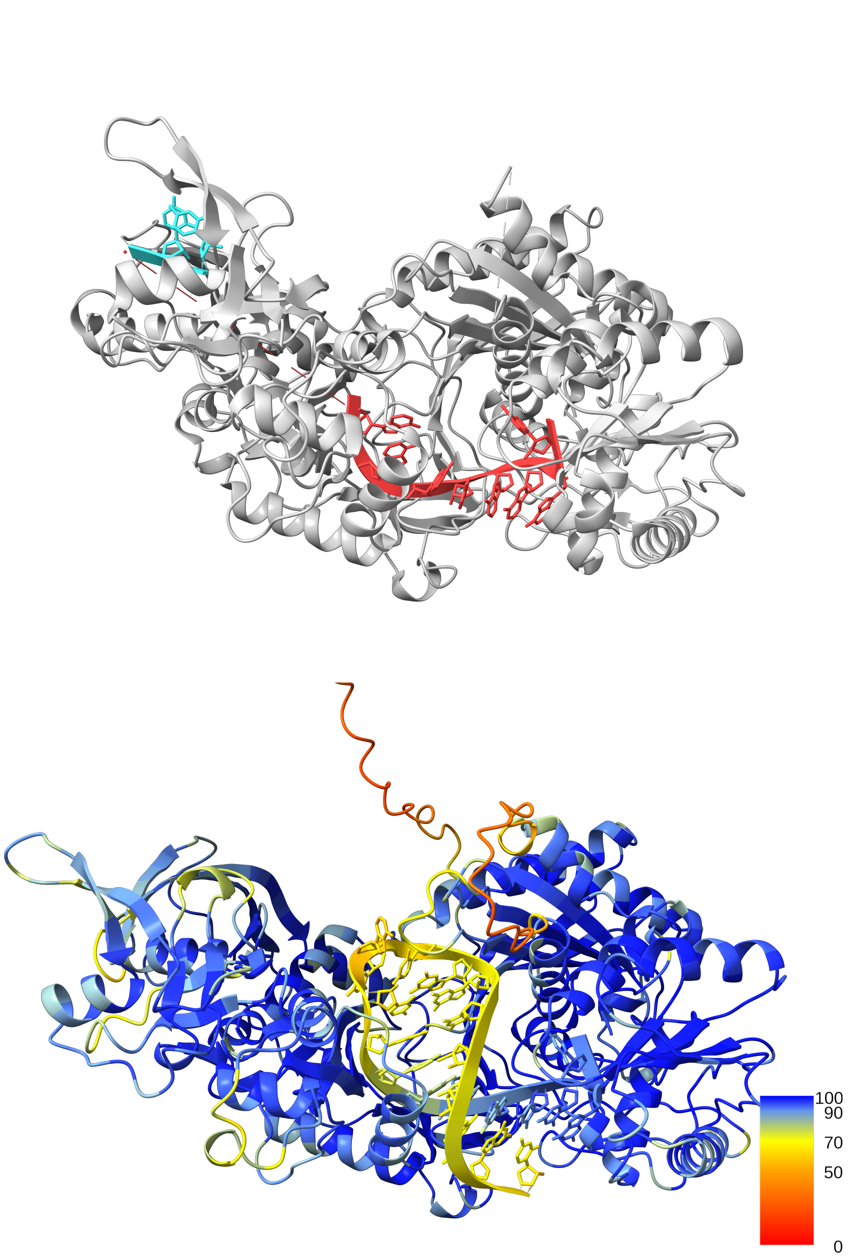

Figure S23: **AlphaFold 3 predicts non-trivial secondary structure in the complex of a point mutant of hsa-miR-6869-3p and Argonaute 1.** Crystal structure of the Argonaute 1 protein in complex with a guide RNA (PDB: 4KXT, top) and AlphaFold 3 predictions for the complex of the same protein non-seed point mutant of hsa-miR-6869-3p with the maximum $\langle d_{\mathrm{Hamming\ mutant}} \rangle$ (bottom). RNA depicted as a ribbons with bases indicated and all residues in the predicted structure coloured by plDDT, a measure of the confidence of the prediction, as indicated by the colour bars. Most of the miRNA structure is predicted with high confidence ($70 < \mathrm{plDDT} < 90$, average: 74.2), and the predictions show base-pairing within the miRNA. The crystal structure does not contain information about the positions of nucleotides 10 to 18 of the guide RNA. Images created with UCSF ChimeraX [213].

Table S24: Maximum-confidence (plDDT) AlphaFold 3 predictions for the complex between human Argonaute 2 protein taken from the PDB structure 4F3T and the miRNAs highlighted by our analysis of secondary structure and the point mutants that have the greatest value of $\Delta p_{\mathrm{unfolded}}$. plDDT values are averaged over all nucleotides.

ID	sequence	SS	average plDDT
WT hsa-miR-485-5p	AGAGGCUGGCCGUGAUGAAUUC	....	71.9
hsa-miR-485-5p mutant with maximum $\Delta p_{\mathrm{unfolded}}$	AGAGGCUAGCCGUGAUGAAUUC	....	56.2
WT hsa-miR-1269b-3p	CUGGACUGAGCCAUGCUACUGG	....	82.7
hsa-miR-1269b-3p mutant with maximum $\Delta p_{\mathrm{unfolded}}$	AUGGACUGAGCCAUGCUACUGG	....	82.0
WT hsa-miR-1908-3p-3p	CCGGCCGCCGGCUCCGCCCCG	....	73.9
hsa-miR-1908-3p-3p mutant with maximum $\Delta p_{\mathrm{unfolded}}$	ACGGCCGCCGGCUCCGCCCCG	....	72.5
WT hsa-miR-4477b-3p	AUUAAGGACAUUUGUGAUUGAU	....	74.1
hsa-miR-4477b-3p mutant with maximum $\Delta p_{\mathrm{unfolded}}$	AUUAAGGAAAUUUGUGAUUGAU	....	65.2
WT hsa-miR-4537-3p	UGAGCCGAGCUGAGCUUAGCUG	....	66.4
hsa-miR-4537-3p mutant with maximum $\Delta p_{\mathrm{unfolded}}$	AGAGCCGAGCUGAGCUUAGCUG	..(((.((((...)))).))).	65.3
WT hsa-miR-4641-3p	UGCCCAUGCCAUACUUUUGCCUCA	....	74.9
hsa-miR-4641-3p mutant with maximum $\Delta p_{\mathrm{unfolded}}$	AGCCCAUGCCAUACUUUUGCCUCA	....	74.1
WT hsa-miR-6821-3p-3p	UGACCUCUCCGCUCCGCACAG	....	85.9
hsa-miR-6821-3p mutant with maximum $\Delta p_{\mathrm{unfolded}}$	AGACCUCUCCGCUCCGCACAG	....	85.2

12 Code and data

We provide the set of Python, Bash and MATLAB scripts that we have used to generate all the results in the manuscript. We also provide the results from our computational predictions of the properties of secondary structure for all studied point mutants and tertiary structure predictions for selected miRNAs in complex with Argonaute proteins. Code and data available at https://doi.org/10.6084/m9.figshare.26490985.v1.

References

[1] A. Bhattacharya and Y. Cui, *Nucleic Acids Research*, 2016, **44**, D1005–D1010.

[2] R. Lorenz, S. H. Bernhart, C. H. zu Siederdissen, H. Tafer, C. Flamm, P. F. Stadler and I. L. Hofacker, *Algorithms for Molecular Biology*, 2011, **6**, 26.

[3] Mathworks, *Receiver operating characteristic (ROC) curve or other performance curve for classifier output - MATLAB perfcurve - MathWorks United Kingdom*, 2022, `https://uk.mathworks.com/help/stats/perfcurve.html`.

[4] Mathworks, *Wilcoxon rank sum test - MATLAB ranksum - MathWorks United Kingdom*, 2022, `https://uk.mathworks.com/help/stats/ranksum.html`.

[5] J. A. Garcia-Martin and P. Clote, *PLoS ONE*, 2015, **10**, 1–32.

[6] R. Bai, Z. Cui, Y. Ma, Y. Wu, N. Wang, L. Huang, Q. Yao and J. Sun, *Molecular Carcinogenesis*, 2019, **58**, 2254–2265.

[7] Y. Xiang, H. Liu, H. Hu, L. W. Li, Q. B. Zong, T. W. Wu, X. Y. Li, S. Q. Fang, Y. W. Liu, Y. Zhan, H. Wang and Z. X. Lu, *Aging*, 2022, **14**, 4755–4768.

[8] A. F. Christopher, M. Gupta and P. Bansal, *Gene*, 2016, **594**, 30–40.

[9] M. Zou, F. Wang, R. Gao, J. Wu, Y. Ou, X. Chen, T. Wang, X. Zhou, W. Zhu, P. Li, L. W. Qi, T. Jiang, W. Wang, C. Li, J. Chen, Q. He and Y. Chen, *Scientific Reports*, 2016, **6**, 1–15.

[10] G. Caruso, L. Falzone, G. Palermo, D. Ricci, G. Mazza, M. Libra, S. Caruso and G. Gattuso, *The Journal of Sexual Medicine*, 2023, **20**, 935–944.

[11] N. Coban, A. F. Erkan, A. S. Ozuynuk-Ertugrul and B. Ekici, *Acta Cardiologica*, 2023, **0**, 1–12.

[12] S. R. Zaker and K. Ghaedi, *Cell Journal*, 2021, **23**, 414–420.

[13] Q. Zeng, F. Jin, H. Qian, H. Chen, Y. Wang, D. Zhang, Y. Wei, T. Chen, B. Guo and C. Chai, *Carcinogenesis*, 2022, **43**, 150–159.

[14] Y. Gu, W. Wang, X. Wang, H. Xie, X. Ye and P. Shu, *Scientific Reports*, 2019, **9**, 1–8.

[15] K. Liu, F. Xie, A. Gao, R. Zhang, L. Zhang, Z. Xiao, Q. Hu, W. Huang, Q. Huang, B. Lin, J. Zhu, H. Wang, J. Que and X. Lan, *Molecular Cancer*, 2017, **16**, 1–14.

[16] S. Laudato, N. Patil, M. L. Abba, J. H. Leupold, A. Benner, T. Gaiser, A. Marx and H. Allgayer, *International Journal of Cancer*, 2017, **141**, 1879–1890.

[17] G. Xu, J. Cai, L. Wang, L. Jiang, J. Huang, R. Hu and F. Ding, *Experimental Cell Research*, 2018, **362**, 268–278.

[18] Z. Ma, F. Chao, S. Wang, Z. Song, Z. Zhuo, J. Zhang, G. Xu and G. Chen, *Biochemical and Biophysical Research Communications*, 2020, **525**, 418–424.

[19] H. Dai, J. Wang, Z. Huang, H. Zhang, X. Wang, Q. Li and W. Feng, *Technology in Cancer Research and Treatment*, 2021, **20**, 1–11.

[20] L. Sabre, P. Maddison, G. Sadalage, P. A. Ambrose and A. R. Punga, *Journal of Neuroimmunology*, 2018, **321**, 164–170.

[21] R. Mishra, S. Bhattacharya, B. S. Rawat, A. Kumar, A. Kumar, K. Niraj, A. Chande, P. Gandhi, D. Khetan, A. Aggarwal, S. Sato, P. Tailor, A. Takaoka and H. Kumar, *iScience*, 2020, **23**, 101322.

[22] B.-S. Kim, J.-Y. Jung, J.-Y. Jeon, H.-A. Kim and C.-H. Suh, *HLA*, 2016, **88**, 187–193.

[23] J. Huang, C. Deng, T. Guo, X. Chen, P. Chen, S. Du and M. Lu, *Anti-Cancer Agents in Medicinal Chemistry*, 2023, **23**, 717–725.

[24] R. M. Busch, L. Yehia, P. Bazeley, M. Seyfi, I. Blümcke, B. P. Hermann, I. M. Najm and C. Eng, *Epilepsia*, 2020, **61**, 2203–2213.

[25] Y. Zhang, L. Geng, G. Talmon and J. Wang, *Journal of Biological Chemistry*, 2015, **290**, 6215–6225.

[26] C. Wang, B. Wang, W. Liang, C. Zhou, W. Lin, Z. Meng, W. Wu, M. Wu, Y. Liao, X. Li, J. Zhao and Y. He, *BMC Cancer*, 2022, **22**, 1–11.

[27] G. Tai, M. Zhang and F. Liu, *Experimental Lung Research*, 2021, **47**, 136–148.

[28] W. Usuba, F. Urabe, Y. Yamamoto, J. Matsuzaki, H. Sasaki, M. Ichikawa, S. Takizawa, Y. Aoki, S. Niida, K. Kato, S. Egawa, T. Chikaraishi, H. Fujimoto and T. Ochiya, *Cancer Science*, 2019, **110**, 408–419.

[29] Y. Zhang, L. Cheng, Y. Chen, G. Y. Yang, J. Liu and L. Zeng, *Journal of Affective Disorders*, 2016, **193**, 51–58.

[30] F. Ren, W. C. Gao, Z. P. Ke, Y. Xu and Y. Liu, *Journal of Cellular Biochemistry*, 2019, **120**, 1932–1942.

[31] H. Cao, A. Baranova, W. Yue, H. Yu, Z. Zhu, F. Zhang and D. Liu, *Frontiers in Genetics*, 2020, **11**, 1–8.

[32] X. Xu and S. Zheng, *Cancer Management and Research*, 2020, **12**, 6137–6147.

[33] H. Ravishankar, A. S. Mangani, G. L. P. Moses, S. P. Mani, S. Parameswaran, V. Khetan, S. Ganesan and S. Krishnakumar, *Experimental Eye Research*, 2020, **199**, 108184.

[34] E. Findeiss, S. C. Schwarz, V. Evsyukov, T. W. Rösler, M. Höllerhage, T. Chakroun, N.-P. Nykänen, Y. Shen, W. Wurst, M. Kohl, J. Tost and G. U. Höglinger, *Frontiers in Cell and Developmental Biology*, 2021, **9**, 561086.

[35] R. Krattinger, A. Boström, H. B. Schiöth, W. E. Thasler, J. Mwinyi and G. A. Kullak-Ublick, *American Journal of Physiology - Gastrointestinal and Liver Physiology*, 2016, **310**, G1044–G1051.

[36] G. Zhou, X. Zhang, W. Wang, W. Zhang, H. Wang and G. Xin, *Medical Science Monitor*, 2019, **25**, 1903–1916.

[37] R. Mysore, Y. Zhou, S. Sädevirta, H. Savolainen-Peltonen, P. A. N. Haridas, J. Soronen, M. Leivonen, A. P. Sarin, P. Fischer-Posovszky, M. Wabitsch, H. Yki-Järvinen and V. M. Olkkonen, *Biochimica et Biophysica Acta - Molecular and Cell Biology of Lipids*, 2016, **1861**, 342–351.

[38] Z. Wang, K. K. Miu, X. Zhang, A. T. Y. Wan, G. Lu, H. H. Cheung, H. M. Lee, A. P. S. Kong, J. C. N. Chan and W. Y. Chan, *JHEP Reports*, 2020, **2**, 100179.

[39] J. Fang, J. Huang and X. Zhang, *Applied Biological Chemistry*, 2022, **65**, 46.

[40] T. Yang, S. Li, J. Liu, D. Yin, X. Yang, Q. Tang and S. Wang, *American Journal of Translational Research*, 2020, **12**, 2939–2955.

[41] P. L. Wander, D. A. Enquobahrie, T. K. Bammler, J. W. MacDonald, S. Srinouanprachanh, T. Kaleru, D. Khakpour and S. Trikudanathan, *Molecular and Cellular Endocrinology*, 2022, **554**, 111723.

[42] Z. lei Zeng, J. huan Lu, Y. Wang, H. Sheng, Y. nan Wang, Z. hong Chen, Q. nian Wu, J. B. Zheng, Y. xing Chen, D. dong Yang, K. Yu, H. yu Mo, J. jia Hu, P. shan Hu, Z. xian Liu, H. qiang Ju and R. H. Xu, *Cancer Medicine*, 2021, **10**, 2423–2441.

[43] X. guo Zhou, X. liang Huang, S. yuan Liang, S. mei Tang, S. kao Wu, T. tong Huang, Z. nan Mo and Q. yan Wang, *OncoTargets and Therapy*, 2018, **Volume 11**, 2815–2830.

[44] G. X. Guo, Q. Y. Li, W. L. Ma, Z. H. Shi and X. Q. Ren, *International Journal of Clinical and Experimental Pathology*, 2015, **8**, 12292–12299.

[45] X. J. Lin, C. L. He, T. Sun, X. J. Duan, Y. Sun and S. J. Xiong, *International Journal of Molecular Medicine*, 2017, **40**, 83–89.

[46] R. sheng Huang, Y. liang Zheng, C. Li, C. Ding, C. Xu and J. Zhao, *Life Sciences*, 2018, **199**, 104–111.

[47] X. X. Hu, X. N. Xu, B. S. He, H. L. Sun, T. Xu, X. X. Liu, X. X. Chen, K. X. Zeng, S. K. Wang and Y. Q. Pan, *Journal of Cancer*, 2018, **9**, 2603–2611.

[48] P. Jiang, C. Xu, L. Chen, A. Chen, X. Wu, M. Zhou, I. U. Haq, Z. Mariyam and Q. Feng, *Journal of Cellular Biochemistry*, 2018, **119**, 8623–8635.

[49] M. Wang, W. R. Cai, R. Meng, J. R. Chi, Y. R. Li, A. X. Chen, Y. Yu and X. C. Cao, *Biochemical and Biophysical Research Communications*, 2018, **501**, 48–54.

[50] D. L. Han, L. L. Wang, G. F. Zhang, W. F. Yang, J. Chai, H. M. Lin, Z. Fu and J. M. Yu, *European Review for Medical and Pharmacological Sciences*, 2019, **23**, 2809–2816.

[51] X. Wang, X. Zhou, F. Zeng, X. Wu and H. Li, *Breast Cancer*, 2020, **27**, 765–775.

[52] D. Wang, X. Zhou, J. Yin and Y. Zhou, *Open Life Sciences*, 2020, **15**, 488–500.

[53] L. Cheng, R. Peng, P. Guo, H. Zhang, D. Liu, X. Liao, Y. Liu, X. Mo and Y. Liao, *Experimental Cell Research*, 2021, **402**, 112547.

[54] Y. Rahmati, M. Alivand and H. Mollanoori, *Computational Biology and Chemistry*, 2021, **92**, 107458.

[55] Y. Chen, L. Wu and M. Bao, *Molecular Biotechnology*, 2022.

[56] J. Chen, S. Wu, J. Wang, Y. Sha and Y. Ji, *Reproductive Sciences*, 2022, **29**, 2236–2250.

[57] X. Chen, S. Zhang, P. Shi, Y. Su, D. Zhang and N. Li, *Current Neurovascular Research*, 2020, **17**, 259–266.

[58] C. Shen, Z. Wu, Y. Wang, S. Gao, L. Da, L. Xie, Y. Qie, D. Tian and H. Hu, *Cancer Medicine*, 2020, **9**, 3885–3903.

[59] S. Park, E. Cheon and H. Kim, *Osteoarthritis and Cartilage*, 2013, **21**, 981–989.

[60] X. Xu, H. Yuan, J. Pan, W. Chen, C. Chen, Y. Li and F. Li, *BMC Cancer*, 2022, **22**, 1–12.

[61] E. Raitoharju, I. Seppälä, N. Oksala, L. P. Lyytikäinen, O. Raitakari, J. Viikari, M. Ala-Korpela, P. Soininen, A. J. Kangas, M. Waldenberger, N. Klopp, T. Illig, J. Leiviskä, B. M. Loo, N. Hutri-Kähönen, M. Kähönen, R. Laaksonen and T. Lehtimäki, *Molecular and Cellular Endocrinology*, 2014, **391**, 41–49.

[62] X. Yu, H. Song, T. Xia, S. Han, B. Xiao, L. Luo, Y. Xi and J. Guo, *Gene*, 2013, **532**, 87–93.

[63] C. Eyileten, Z. Wicik, D. Keshwani, F. Aziz, F. Aberer, P. N. Pferschy, N. J. Tripolt, C. Sourij, B. Prietl, F. Prüller, D. von Lewinski, S. D. Rosa, J. M. Siller-Matula, M. Postula and H. Sourij, *Cardiovascular Diabetology*, 2022, **21**, 1–12.

[64] A. Gholaminejad, A. Roointan and Y. Gheisari, *BMC Immunology*, 2021, **22**, 1–17.

[65] Y. Sun, X. Wang, Z. Wang, Y. Zhang, N. Che, X. Luo, Z. Tan, X. Sun, X. Li, K. Yang, G. Wang, L. Luan, Y. Liu, X. Zheng, M. Wei, H. Cheng and J. Yin, *Epilepsy Research*, 2016, **127**, 276–283.

[66] C. Zhou, X. Zhao and S. Duan, *Journal of Cellular Physiology*, 2021, **236**, 15–26.

[67] M. Scheper, A. Iyer, J. J. Anink, L. Mesarosova, J. D. Mills and E. Aronica, *Neuropathology and Applied Neurobiology*, 2023, **49**, 1–13.

[68] R. N. Quiroz, A. L. Scott, E. A. Philot, L. Atencio, C. F. Ponce, G. A. Martinez, A. C. Bonfanti, L. G. Escorcia and E. N. Quiroz, *bioRxiv*, 2020.

[69] T. T. N. Nguyen, T. H. N. Nguyen, L. H. Huynh, H. N. Phan and H. T. Nguyen, *Recent Advances in Noncoding RNAs*, 2022, 1–18.

[70] L. Zhong, Y. Wang, Y. Cheng, W. Wang, B. Lu, L. Zhu and Y. Ma, *Biochemical and Biophysical Research Communications*, 2018, **499**, 1044–1049.

[71] K. Tu, Z. Liu, B. Yao, S. Han and W. Yang, *International Journal of Oncology*, 2016, **48**, 965–974.

[72] A. V. Timofeeva, V. A. Gusar, N. E. Kan, K. N. Prozorovskaya, A. O. Karapetyan, O. R. Bayev, V. V. Chagovets, S. F. Kliver, D. Y. Iakovishina, V. E. Frankevich and G. T. Sukhikh, *Placenta*, 2018, **61**, 61–71.

[73] E. Tolosa, T. Botta-Orfila, X. Morató, C. Calatayud, R. Ferrer-Lorente, M. J. Martí, M. Fernández, C. Gaig, Ángel Raya, A. Consiglio, M. Ezquerra and R. Fernández-Santiago, *Neurobiology of Aging*, 2018, **69**, 283–291.

[74] J. Liu, S. Yan, J. Hu, D. Ding, Y. Liu, X. Li, H. S. Pan, G. Liu, B. Wu and Y. Liu, *Bioengineered*, 2021, **12**, 8457–8467.

[75] A. Du, S. Zhao, L. Wan, T. Liu, Z. Peng, Z. Zhou, Z. Liao and H. Fang, *Journal of Cellular and Molecular Medicine*, 2016, **20**, 1329–1338.

[76] A. Romero-Ruiz, B. Pineda, D. Ovelleiro, C. Perdices-Lopez, E. Torres, M. J. Vazquez, I. Guler, Álvaro Jiménez, R. Pineda, M. Persano, C. Romero-Baldonado, J. E. Arjona, J. Lorente, C. Muñoz, E. Paz, F.-I. Garcia-Maceira, Álvaro Arjona-Sánchez and M. Tena-Sempere, *European Journal of Endocrinology*, 2021, **185**, 637–652.

[77] E. C. Willner, H. L. Galan, B. F. Cuneo, H. A. Hoffman, B. Neltner, E. L. Schuchardt, A. Karimpour-Fard, S. D. Miyamoto and C. C. Sucharov, *American Journal of Obstetrics and Gynecology*, 2021, **225**, 439.e1–439.e10.

[78] K. D. Silva, R. T. Demmer, D. Jönsson, A. Mousa, A. Forbes and J. Enticott, *Heliyon*, 2022, **8**, e08886.

[79] D. Anatolou, N. Dovrolis, G. Ragia, G. Kolios and V. G. Manolopoulos, *OMICS: A Journal of Integrative Biology*, 2022, **26**, 608–621.

[80] J. M. Modak, M. Roy-O'Reilly, L. Zhu, I. Staff and L. D. McCullough, *Journal of Stroke and Cerebrovascular Diseases*, 2019, **28**, 121–124.

[81] S. Zhou, Q. Meng, L. Li, L. Hai, Z. Wang, Z. Li and Y. Sun, *Frontiers in Genetics*, 2021, **12**, 1–9.

[82] D. Poudyal, A. Herman, J. W. Adelsberger, J. Yang, X. Hu, Q. Chen, M. Bosche, B. T. Sherman and T. Imamichi, *Scientific Reports*, 2018, **8**, 1–13.

[83] X. Wang, J. Gao, B. Zhou, J. Xie, G. Zhou and Y. Chen, *Life Sciences*, 2019, **232**, 116596.

[84] W. Yang, W. Xiao, Z. Cai, S. Jin and T. Li, *OncoTargets and Therapy*, 2020, **13**, 109–118.

[85] Z. Xie, C. Zhong and S. Duan, *Frontiers in Cell and Developmental Biology*, 2022, **10**, 1–11.

[86] C. Jia, Z. Yao, Z. Lin, L. Zhao, X. Cai, S. Chen, M. Deng and Q. Zhang, *Journal of Cellular Physiology*, 2021, **236**, 1252–1269.

[87] G. C. Genc, A. Dursun, S. K. Celik, M. Calik, F. Kokturk and I. E. Piskin, *Gene*, 2018, **678**, 73–78.

[88] A. Mariam, G. Miller-Atkins, A. Moro, A. I. Rodarte, S. Siddiqi, L.-A. Acevedo-Moreno, J. M. Brown, D. S. Allende, F. Aucejo and D. M. Rotroff, *PeerJ*, 2022, **10**, e12715.

[89] J. B. de Carvalho, G. L. de Morais, T. C. dos Santos Vieira, N. C. Rabelo, J. C. Llerena, S. M. de Carvalho Gonzalez and A. T. R. de Vasconcelos, *Frontiers in Genetics*, 2019, **10**, 1–11.

[90] C. Medina-Trillo, J.-D. Aroca-Aguilar, J.-J. Ferre-Fernández, C.-D. Méndez-Hernández, L. Morales, J. García-Feijoo and J. Escribano, *MicroRNA*, 2015, **4**, 50–56.

[91] Y. Wang, L. Zou, T. Wu, L. Xiong, T. Zhang, L. Kong, Y. Xue and M. Tang, *Ecotoxicology and Environmental Safety*, 2019, **169**, 863–873.

[92] Y. Yan, D. Song, X. Zhang, G. Hui and J. Wang, *Frontiers in Pharmacology*, 2020, **11**, 1–7.

[93] E. Budd, G. Nalesso and A. Mobasheri, *Expert Review of Molecular Diagnostics*, 2018, **18**, 55–74.

[94] B. Gao, Q. Shao, H. Choudhry, V. Marcus, K. Dong, J. Ragoussis and Z. H. Gao, *International Journal of Oncology*, 2016, **49**, 1108–1118.

[95] S. Nomiri, R. Hoshyar, E. Chamani, Z. Rezaei, F. Salmani, P. Larki, T. Tavakoli, F. gholipour, N. J. Tabrizi, A. Derakhshani, M. Santarpia, T. Franchina, O. Brunetti, N. Silvestris and H. Safarpour, *Biomedicine and Pharmacotherapy*, 2022, **147**, 112691.

[96] S. Orsten, I. Baysal, S. Yabanoglu-Ciftci, T. Ciftci, A. Azizova, D. Akinci, Y. Akyon and O. Akhan, *Journal of Helminthology*, 2021, **95**, e1.

[97] P. C. Sanchez-Diaz, T.-H. Hsiao, J. C. Chang, D. Yue, M. C. Tan, H.-I. H. Chen, G. E. Tomlinson, Y. Huang, Y. Chen and J. Y. Hung, *PLoS ONE*, 2013, **8**, e61622.

[98] B. Chen, S. She, D. Li, Z. Liu, X. Yang, Z. Zeng and F. Liu, *Scandinavian Journal of Gastroenterology*, 2013, **48**, 815–824.

[99] K. Kochan-Jamrozy, J. Króliczewski, A. Moszyńska, J. F. Collawn and R. Bartoszewski, *Cellular Signalling*, 2019, **54**, 150–160.

[100] P. Yan, P. Pang, X. Hu, A. Wang, H. Zhang, Y. Ma, K. Zhang, Y. Ye, B. Zhou and J. Mao, *Journal of Cancer*, 2021, **12**, 1–9.

[101] M. Chen, X. Wang, H. Wang, M. Zhang, L. Chen and H. Chen, *ResearchSquare Pre-print*, 2021, 1–12.

[102] F. Ni, Z. Gui, Q. Guo, Z. Hu, X. Wang, D. Chen and S. Wang, *Oncology Letters*, 2016, **11**, 1155–1160.

[103] B. M. Ryan, A. C. McClary, N. Valeri, D. Robinson, A. Paone, E. D. Bowman, A. I. Robles, C. Croce and C. C. Harris, *PLoS ONE*, 2012, **7**, 3–8.

[104] N. Othman, L. L. In, J. A. Harikrishna and N. Hasima, *PLoS ONE*, 2013, **8**, 1–14.

[105] J. Zheng, J. Deng, M. Xiao, L. Yang, L. Zhang, Y. You, M. Hu, N. Li, H. Wu, W. Li, J. Lu and Y. Zhou, *Cancer Research*, 2013, **73**, 5151–5162.

[106] F. Qiu, L. Yang, L. Zhang, X. Yang, R. Yang, W. Fang, D. Wu, J. Chen, C. Xie, D. Huang, Y. Zhou and J. Lu, *Gene*, 2015, **565**, 180–186.

[107] T. Xu, H. Q. Xie, Y. Li, Y. Xia, Y. Chen, L. Xu, L. Wang and B. Zhao, *Scientific Reports*, 2017, **7**, 1–10.

[108] H. Yan, S. Xin, J. Ma, H. Wang, H. Zhang and J. Liu, *Journal of Cellular Biochemistry*, 2019, **120**, 8723–8730.

[109] N. Zhang, Y. Li, Y. Zheng, L. Zhang, Y. Pan, J. Yu and M. Yang, *Laboratory Investigation*, 2019, **99**, 568–576.

[110] W. Gu, D. Wen, H. Lu, A. Zhang, H. Wang, J. Du, L. Zeng and J. Jiang, *Journal of Clinical Immunology*, 2020, **40**, 147–157.

[111] Y. Abrahams, M.-J. Laguette, S. Prince and M. Collins, *Annals of Human Genetics*, 2013, **77**, 204–214.

[112] B. Cheng, J. yi Li, X. chao Li, X. fang Wang, Z. jing Wang, J. Liu and A. ping Deng, *Scientific Reports*, 2018, **8**, 1–9.

[113] C. Sun, M. Liu, W. An, J. Liu, F. Yang, F. Wang, J. Jiang, Q. Zhou, Y. Jia, Y. Wang, J. Yuan, L. Ma, X. Sun, L. Wang, Z. Liao and Z. Li, *The Journal of Gene Medicine*, 2022, 1–10.

[114] X. He and Y. Feng, *Anti-Cancer Drugs*, 2022, **33**, 478–488.

[115] Y. Wang, M. Chen, Z. Tao, Q. Hua, S. Chen and B. Xiao, *Cancer Genetics*, 2013, **206**, 340–346.

[116] S. K. Saini, P. Kalaiarasan, R. K. Singh, S. Manvati and R. N. Bamezai, *Mitochondrion*, 2018, **43**, 30–36.

[117] S. Kong, Y. Cao, X. Li, Z. Li, Y. Xin and Y. Meng, *Journal of Cellular and Molecular Medicine*, 2020, **24**, 4677–4686.

[118] D. Liu, L. Zhong, Z. Yuan, J. Yao, P. Zhong, J. Liu, S. Yao, Y. Zhao, L. Liu, M. Chen, L. Li and B. Liu, *Cellular Signalling*, 2019, **54**, 1–9.

[119] J. Du, F. Bai, P. Zhao, X. Li, X. Li, L. Gao, C. Ma and X. Liang, *Biochimica et Biophysica Acta - Molecular Cell Research*, 2018, **1865**, 1–11.

[120] Z. Derakhshan, G. Khamisipour, F. H. Soleimani and N. Motamed, *Gene Reports*, 2022, **27**, 101582.

[121] X. Nie, H. Liu, X. Wei, L. Li, L. Lan, L. Fan, H. Ma, L. Liu, Y. Zhou, R. Hou and W.-D. Chen, *Cancer Management and Research*, 2021, **Volume 13**, 8025–8035.

[122] R. Fang, Y. Zhu, L. Hu, V. S. Khadka, J. Ai, H. Zou, D. Ju, B. Jiang, Y. Deng and X. Hu, *Frontiers in Physiology*, 2019, **10**, 1–12.

[123] Y. W. Hu, J. Y. Zhao, S. F. Li, J. L. Huang, Y. R. Qiu, X. Ma, S. G. Wu, Z. P. Chen, Y. R. Hu, J. Y. Yang, Y. C. Wang, J. J. Gao, Y. H. Sha, L. Zheng and Q. Wang, *Arteriosclerosis, Thrombosis, and Vascular Biology*, 2015, **35**, 87–101.

[124] G. Wojciechowska, L. Szczerbinski, M. Kretowski, M. Niemira, H. R. Hady and A. Kretowski, *Obesity*, 2022, **30**, 435–446.

[125] J. J. Zhao, J. Yang, J. Lin, N. Yao, Y. Zhu, J. Zheng, J. Xu, J. Q. Cheng, J. Y. Lin and X. Ma, *Child's Nervous System*, 2009, **25**, 13–20.

[126] J. von Frowein, P. Pagel, R. Kappler, D. von Schweinitz, A. Roscher and I. Schmid, *Hepatology*, 2011, **53**, 833–842.

[127] X. Song, Y. Xie, Y. Liu, M. Shao and W. Yang, *International Journal of Molecular Medicine*, 2017, **40**, 891–897.

[128] E. Taghizadeh, F. Taheri, D. Rostami, P. G. Renani, G. A. Ferns, A. Pasdar and M. G. Mobarhan, *Current Cancer Therapy Reviews*, 2020, **16**, 269–275.

[129] K. Wang, H. Lü, H. Qu, Q. Xie, T. Sun, O. Gan and B. Hu, *OncoTargets and Therapy*, 2019, **12**, 11453–11464.

[130] S. Zhao, L. Tang, W. Chen, J. Su, F. Li, X. Chen and L. Wu, *Naunyn-Schmiedeberg's Archives of Pharmacology*, 2021, **394**, 797–807.

[131] N. N. A. Deen, N. A. Lanman, S. Chittiboyina, S. Fostok, R. Nasr, S. Lelièvre and R. Talhouk, *Scientific reports*, 2022, **12**, 21974.

[132] D. S. Jairajpuri, Z. H. Malalla, N. Mahmood and W. Y. Almawi, *Gene*, 2017, **627**, 543–548.

[133] R. Shams, S. Saberi, M. Zali, A. Sadeghi, S. Ghafouri-Fard and H. A. Aghdaei, *Scientific Reports*, 2020, **10**, 1–15.

[134] K. A. Gaither, C. J. Watson, B. Madarampalli and P. Lazarus, *PLoS ONE*, 2020, **15**, 1–19.

[135] J. D. Galley, P. Mar, Y. Wang, R. Han, A. Rajab and G. E. Besner, *Journal of Pediatric Surgery*, 2021, **56**, 1966–1975.

[136] L. Hongdan and L. Feng, *Biochemical and Biophysical Research Communications*, 2018, **496**, 302–308.

[137] H. Li, Q. Zhao and Z. Tang, *Pathology Research and Practice*, 2021, **226**, 153566.

[138] K. J. Capistrano, J. Richner, J. Schwartz, S. K. Mukherjee, D. Shukla and A. R. Naqvi, *Biochimica et Biophysica Acta - Molecular Basis of Disease*, 2023, **1869**, 166612.

[139] A. Fernández-Pato, A. Virseda-Berdices, S. Resino, P. Ryan, O. Martínez-González, F. Pérez-García, M. Martin-Vicente, D. Valle-Millares, O. Brochado-Kith, R. Blancas, A. Martínez, F. C. Ceballos, S. Bartolome-Sánchez, E. J. Vidal-Alcántara, D. Alonso, N. Blanca-López, I. R. Martinez-Acitores, L. Martin-Pedraza, M. Ángeles Jiménez-Sousa and A. Fernández-Rodríguez, *Emerging Microbes and Infections*, 2022, **11**, 676–688.

[140] E. Chang, G. Fishbein, M. Bakir, G. Bondar, N. Jackson, D. Liem, S. Litovsky, J. Tallaj, C. Starling, P. Ping, E. Reed, M. Deng, E. Tabak and M. Cadeiras, *Circulation*, 2015, **132**,.

[141] J. M. Moreno, M. J. Núñez, A. Quiñonero, S. Martínez, M. D. L. Orden, C. Simón, A. Pellicer, C. Díaz-García and F. Domínguez, *Fertility and Sterility*, 2015, **104**, 1037–1046.e1.

[142] X. Zhuang, Z. Li, H. Lin, L. Gu, Q. Lin, Z. Lu and C. M. Tzeng, *Scientific Reports*, 2015, **5**, 1–9.

[143] Y. Liang, G. Zhao, L. Tang, J. Zhang, T. Li and Z. Liu, *Experimental Cell Research*, 2016, **347**, 312–321.

[144] L. Ou, W. Huang, T. Zhang, D. Xu, D. Kong and Y. Meng, *Transplant Immunology*, 2023, **77**, 101747.

[145] A. K. Singh, S. B. Rooge, A. Varshney, M. Vasudevan, A. Bhardwaj, S. K. Venugopal, N. Trehanpati, M. Kumar, R. Geffers, V. Kumar and S. K. Sarin, *Hepatology*, 2018, **67**, 1695–1709.

[146] J. Cui, X. Kang, Y. Shan, M. Zhang, Y. Gao, W. Wu and L. Chen, *Scientific Reports*, 2022, **12**, 1–10.

[147] N. Coban, A. S. Ozuynuk, A. F. Erkan, F. Guclu-Geyik and B. Ekici, *Molecular Biology Reports*, 2021, **48**, 7719–7732.

[148] X. He, G. Cheng, F. Xiao, L. Zhang, G. Jin, X. Zhao, Y. Liu, J. Liang, Y. Li, Z. Liu, Q. Yuan, H. Ren, Q. Wu, J. Wu, L. Xue, J. Feng, Z. Wang, Y. Xing, W. Wu, Z. Li, D. Wei and X. Song, *Journal of Gastrointestinal Oncology*, 2021, **12**, 69–78.

[149] H. Yang, Q. Li, Y. Wu, J. Dong, Y. Lao, Z. Ding, C. Xiao, J. Fu and S. Bai, *Oncology Reports*, 2020, **44**, 2045–2055.

[150] T. Nishimura, E. Tamizu, S. Uno, Y. Uwamino, H. Fujiwara, K. Nishio, Y. Nakano, H. Shiono, H. Namkoong, Y. Hoshino, S. Iwata and N. Hasegawa, *Journal of Infection and Chemotherapy*, 2017, **23**, 703–708.

[151] P. Muti, S. Donzelli, A. Sacconi, A. Hossain, F. Ganci, T. Frixa, S. Sieri, V. Krogh, F. Berrino, F. Biagioni, S. Strano, J. Beyene, Y. Yarden and G. Blandino, *Carcinogenesis*, 2018, **39**, 98–108.

[152] L. Falzone, G. Romano, R. Salemi, C. Bucolo, B. Tomasello, G. Lupo, C. Anfuso, D. Spandidos, M. Libra and S. Candido, *Molecular Medicine Reports*, 2019, **19**, 2599–2610.

[153] Q. Zhou, H. Li, Y. Zhang, W. Peng, H. Hou, M. Gu, F. Zhang, X. Wang, X. Gu and L. Li, *BMC Pregnancy and Childbirth*, 2021, **21**, 837.

[154] J. Ma, S. Shang, J. Wang, T. Zhang, F. Nie, X. Song, H. Zhao, C. Zhu, R. Zhang and D. Hao, *Psychiatry Research*, 2018, **265**, 70–76.

[155] G. N. López-Sánchez, E. Montalvo-Javé, M. Domínguez-Perez, B. Antuna-Puente, F. O. Beltrán-Anaya, A. Hidalgo-Miranda, N. C. Chávez-Tapia, M. Uribe and N. Nuño-Lámbarri, *Annals of Hepatology*, 2022, **27**, 100756.

[156] P. Kuang, P. Chen, L. Wang, W. Li, B. Chen, Y. Liu, Y. Xu, H. Wang, S. Zhao, L. Ye, F. Yu, H. Ji and Y. He, *Annals of Translational Medicine*, 2020, **8**, 121–121.

[157] M. Tanic, K. Yanowski, G. Gómez-López, M. S. Rodriguez-Pinilla, I. Marquez-Rodas, A. Osorio, D. G. Pisano, B. Martinez-Delgado and J. Benítez, *International Journal of Cancer*, 2015, **136**, 593–602.

[158] S. I. Jang, M. Tandon, L. Teos, C. Y. Zheng, B. M. Warner and I. Alevizos, *EBioMedicine*, 2019, **48**, 526–538.

[159] H. Hidaka, N. Seki, H. Yoshino, T. Yamasaki, Y. Yamada, N. Nohata, M. Fuse, M. Nakagawa and H. Enokida, *Oncotarget*, 2012, **3**, 44–57.

[160] A. Oroujalian, M. Peymani and K. Ghaedi, *Nucleosides, Nucleotides and Nucleic Acids*, 2021, **40**, 779–789.

[161] J. Guo, X. Fang, J. Zhou, L. Zeng and B. Yu, *Medicine (United States)*, 2022, **101**, E29705.

[162] A. Keller, P. Leidinger, J. Lange, A. Borries, H. Schroers, M. Scheffler, H.-P. Lenhof, K. Ruprecht and E. Meese, *PLoS ONE*, 2009, **4**, e7440.

[163] F. Finocchi, M. Pelloni, G. Balercia, F. Pallotti, A. F. Radicioni, A. Lenzi, F. Lombardo and D. Paoli, *Molecular Biology Reports*, 2020, **47**, 4373–4382.

[164] X. Jiang, M. Jiang, M. Xu, J. Xu and Y. Li, *Pathology - Research and Practice*, 2019, **215**, 900–904.

[165] Y. hao Wang, Y. wang Chen, W. li Xiao, X. lian Li, L. Feng, Y. lin Liu and X. xia Duan, *Current Medical Science*, 2022, **42**, 871–884.

[166] L. Meng, S. Chang, Y. Sang, P. Ding, L. Wang, X. Nan, R. Xu, F. Liu, L. Gu, Y. Zheng, Z. Li and M. Sang, *Breast Cancer Research*, 2022, **24**, 1–13.

[167] U. Heilmeier, M. Hackl, S. Skalicky, S. Weilner, F. Schroeder, K. Vierlinger, J. M. Patsch, T. Baum, E. Oberbauer, I. Lobach, A. J. Burghardt, A. V. Schwartz, J. Grillari and T. M. Link, *Journal of Bone and Mineral Research*, 2016, **31**, 2173–2192.

[168] H. Jia, H. Wang, F. Xia, Y. Sun, H. Liu, L. Yan, S. Li, D. Jiang and M. Xu, *DNA and Cell Biology*, 2020, **39**, 2257–2264.

[169] C. Tang, H. Wang, H. Wu, S. Yan, Z. Han, Z. Jiang, M. Na, M. Guo, D. Lu and Z. Lin, *Cellular and Molecular Neurobiology*, 2019, **39**, 461–470.

[170] J. Li, X. Yang, H. Guan, A. Mizokami, E. T. Keller, X. Xu, X. Liu, J. Tan, L. Hu, Y. Lu and J. Zhang, *International Journal of Oncology*, 2016, **49**, 838–846.

[171] Y. Hao, C. Lu, B. Zhang, Z. Xu, H. Guo and G. Zhang, *Clinical Interventions in Aging*, 2021, **16**, 187–202.

[172] L. Qi, C. Gao, F. Feng, T. Zhang, Y. Yao, X. Wang, C. Liu, J. Li, J. Li and C. Sun, *Journal of Cellular Biochemistry*, 2019, **120**, 18956–18966.

[173] Y. Qu, X. Huang, Z. Li, J. Liu, J. Wu, D. Chen, F. Zhao and D. Mu, *American Journal of Pathology*, 2014, **184**, 1541–1549.

[174] B. F. Chen, S. Gu, Y. K. Suen, L. Li and W. Y. Chan, *Epigenetics*, 2014, **9**, 119–128.

[175] L. Li, Y. P. Mou, Y. Y. Wang, H. J. Wang and X. Z. Mou, *Pathology Research and Practice*, 2019, **215**, 152511.

[176] R. Zhu, K. Nasu, N. Hijiya, M. Yoshihashi, T. Hirakawa, Y. Aoyagi and H. Narahara, *Reproductive Sciences*, 2021, **28**, 3498–3507.

[177] P. Bardin, E. Marchal-Duval, F. Sonneville, S. Blouquit-Laye, N. Rousselet, P. L. Rouzic, H. Corvol and O. Tabary, *Journal of Pathology*, 2018, **245**, 410–420.

[178] A. Shang, C. Zhou, G. Bian, W. Chen, W. Lu, W. Wang and D. Li, *Journal of Cellular Biochemistry*, 2019, **120**, 778–789.

[179] Y. Z. Yu, Q. Mu, Q. Ren, L. J. Xie, Q. T. Wang and C. P. Wang, *World Journal of Surgical Oncology*, 2021, **19**, 1–11.

[180] L. Bi, C. Zhang, Y. Yao and Z. He, *Journal of Biosciences*, 2021, **46**, 20.

[181] Z. Liu, Y. Yu, Z. Huang, Y. Kong, X. Hu, W. Xiao, J. Quan and X. Fan, *Cell Death & Disease*, 2019, **10**, 900.

[182] V. N. Aushev, D. D. Esposti, E. Lee, H. Vargas, Z. Herceg, J. Zhu and J. Chen, *Cancer Research*, 2016, **76**, 1896–1896.

[183] Z. Yang, S. Lu, Y. Wang, H. Tang, B. Wang, X. Sun, J. Qu and B. Rao, *International Journal of General Medicine*, 2022, **15**, 555–565.

[184] M. Asakage, Y. Usui, N. Nezu, H. Shimizu, K. Tsubota, N. Yamakawa, M. Takanashi, M. Kuroda and H. Goto, *Investigative Opthalmology & Visual Science*, 2020, **61**, 4.

[185] D. F. Pellatt, J. R. Stevens, R. K. Wolff, L. E. Mullany, J. S. Herrick, W. Samowitz and M. L. Slattery, *Clinical and Translational Gastroenterology*, 2016, **7**, e152.

[186] L. Liu, S. Han, X. Xiao, X. An, J. Gladkich, U. Hinz, S. Hillmer, T. Hoppe-Tichy, Y. Xu, M. Schaefer, O. Strobel and I. Herr, *Cell Death & Disease*, 2022, **13**, 1052.

[187] C. Gungormez, H. G. Aktas, N. Dilsiz and E. Borazan, *Molecular Biology Reports*, 2019, **46**, 4175–4183.

[188] Z. H. Huang, H. Wang, D. M. Wang, X. Y. Zhao, W. W. Liu, X. Zhong, D. M. He, B. R. Mu and M. H. Lu, *Journal of Cellular and Molecular Medicine*, 2022, **26**, 5779–5793.

[189] Y. Ma, X. Cong, Y. Zhang, X. Yin, Z. Zhu and Y. Xue, *Cancer Cell International*, 2020, **20**, 1–12.

[190] L. Zhang, F. Liu, Z. Meng, Q. Luo, D. Pan and Y. Qian, *Genomics*, 2021, **113**, 3512–3522.

[191] K. Wang, J. Jin, T. Ma and H. Zhai, *Biomedicine and Pharmacotherapy*, 2017, **91**, 517–525.

[192] M. J. Hsieh, C. W. Lin, S. C. Su, R. J. Reiter, A. W. G. Chen, M. K. Chen and S. F. Yang, *Molecular Therapy - Nucleic Acids*, 2020, **19**, 877–889.

[193] W. long Liang, J. Cao, B. Xu, P. Yang, F. Shen, Z. Sun, W. lin Li, Q. Wang and F. Liu, *Biomedicine & Pharmacotherapy*, 2015, **72**, 119–124.

[194] S. Li, Y. Liu, G. Qiu, Y. Luo, L. Luan, T. Xu, Y. Wang and S. Xia, *Cancer Management and Research*, 2021, **Volume 13**, 1967–1979.

[195] J. Dong, J. Wang, C. Shan, H. Zhang and O. Xu, *Experimental Biology and Medicine*, 2020, **245**, 1222–1232.

[196] Y. Shang, Z. Zhang, Z. Liu, B. Feng, G. Ren, K. Li, L. Zhou, Y. Sun, M. Li, J. Zhou, Y. An, K. Wu, Y. Nie and D. Fan, *Oncogene*, 2014, **33**, 3267–3276.

[197] Q. Liu, H. Li, N. Wang, H. Chen and J. Wang, *International Journal of Cardiology*, 2013, **168**, 2082–2088.

[198] B. Yang, X. Huang, S. Xu, L. Li, W. Wu, Y. Dai, M. X. Ge, L. Yuan, W. Cao, M. Yang, Y. Wu and D. Deng, *Frontiers in Immunology*, 2021, **12**, 1–14.

[199] H. Mutlu, S. Mutlu and M. Bostancıklıoğlu, *Anti-Cancer Agents in Medicinal Chemistry*, 2021, **21**, 1732–1737.

[200] Y. Dai, L. Huang, H. Zhang, G. Hong, Y. He, J. Hu and Y. Liu, *Injury*, 2021, **52**, 11–18.

[201] Y. Yang, Y. Liu, N. Xie, L. Shao, H. Sun, Y. Wei, Y. Sun, P. Wang, Y. Yan, S. Xie and Y. Li, *Experimental and Therapeutic Medicine*, 2021, **22**, 1305.

[202] H. Wang, M. Chen, S. Xu, Y. Pan, Y. Zhang, H. Huang and L. Xu, *Journal of Clinical Laboratory Analysis*, 2021, **35**, e23945.

[203] L. Mei, Y. Zheng, X. Gao, T. Ma, B. Xia, Y. Hao, B. Wei, Y. Wei, Z. Luo and J. Huang, *Pharmacological Research*, 2022, **186**, 106537.

[204] A. Bogucka-Kocka, D. P. Zalewski, K. P. Ruszel, A. Stępniewski, D. Gałkowski, J. Bogucki, Łukasz Komsta, P. Kołodziej, T. Zubilewicz, M. Feldo and J. Kocki, *Frontiers in Genetics*, 2019, **10**, 1–14.

[205] H. Hozhabri, M. M. Moghaddam, M. M. Moghaddam and A. Mohammadian, *Scientific Reports*, 2022, **12**, 1–19.

[206] S. M. Wang, P. W. Yang, X. J. Feng, Y. W. Zhu, F. J. Qiu, X. D. Hu and S. H. Zhang, *Frontiers in Oncology*, 2021, **11**, 1–12.

[207] Y. Hou, X. Zhang, H. Yao, L. Hou, Q. Zhang, E. Tao, X. Zhu, S. Jiang, Y. Ren, X. Hong, S. Lu, X. Leng, Y. Xie, Y. Gao, Y. Liang, T. Zhong, B. Long, J. Fang and X. Meng, *EMBO reports*, 2023, **24**, 1–21.

[208] Y. Yang and Q. Wang, *Scientific Reports*, 2023, **13**, 1–15.

[209] Mathworks, *Bootstrap confidence interval - MATLAB bootci - MathWorks United Kingdom*, `https://uk.mathworks.com/help/stats/bootci.html`.

[210] M. B. Brown, *Biometrics*, 1975, **31**, 987.

[211] J. Abramson, J. Adler, J. Dunger, R. Evans, T. Green, A. Pritzel, O. Ronneberger, L. Willmore, A. J. Ballard, J. Bambrick, S. W. Bodenstein, D. A. Evans, C. C. Hung, M. O'Neill, D. Reiman, K. Tunyasuvunakool, Z. Wu, A. Žemgulytė, E. Arvaniti, C. Beattie, O. Bertolli, A. Bridgland, A. Cherepanov, M. Congreve, A. I. Cowen-Rivers, A. Cowie, M. Figurnov, F. B. Fuchs, H. Gladman, R. Jain, Y. A. Khan, C. M. Low, K. Perlin, A. Potapenko, P. Savy, S. Singh, A. Stecula, A. Thillaisundaram, C. Tong, S. Yakneen, E. D. Zhong, M. Zielinski, A. Žídek, V. Bapst, P. Kohli, M. Jaderberg, D. Hassabis and J. M. Jumper, *Nature*, 2024, **630**, 493–500.

[212] X.-J. Lu, H. J. Bussemaker and W. K. Olson, *Nucleic Acids Research*, 2015, **43**, e142.

[213] E. C. Meng, T. D. Goddard, E. F. Pettersen, G. S. Couch, Z. J. Pearson, J. H. Morris and T. E. Ferrin, *Protein Science*, 2023, **32**, e4792.
